# Caribbean Corals in Crisis: Record Thermal Stress, Bleaching, and Mortality in 2005

**DOI:** 10.1371/journal.pone.0013969

**Published:** 2010-11-15

**Authors:** C. Mark Eakin, Jessica A. Morgan, Scott F. Heron, Tyler B. Smith, Gang Liu, Lorenzo Alvarez-Filip, Bart Baca, Erich Bartels, Carolina Bastidas, Claude Bouchon, Marilyn Brandt, Andrew W. Bruckner, Lucy Bunkley-Williams, Andrew Cameron, Billy D. Causey, Mark Chiappone, Tyler R. L. Christensen, M. James C Crabbe, Owen Day, Elena de la Guardia, Guillermo Díaz-Pulido, Daniel DiResta, Diego L. Gil-Agudelo, David S. Gilliam, Robert N. Ginsburg, Shannon Gore, Héctor M. Guzmán, James C. Hendee, Edwin A. Hernández-Delgado, Ellen Husain, Christopher F. G. Jeffrey, Ross J. Jones, Eric Jordán-Dahlgren, Les S. Kaufman, David I. Kline, Philip A. Kramer, Judith C. Lang, Diego Lirman, Jennie Mallela, Carrie Manfrino, Jean-Philippe Maréchal, Ken Marks, Jennifer Mihaly, W. Jeff Miller, Erich M. Mueller, Erinn M. Muller, Carlos A. Orozco Toro, Hazel A. Oxenford, Daniel Ponce-Taylor, Norman Quinn, Kim B. Ritchie, Sebastián Rodríguez, Alberto Rodríguez Ramírez, Sandra Romano, Jameal F. Samhouri, Juan A. Sánchez, George P. Schmahl, Burton V. Shank, William J. Skirving, Sascha C. C. Steiner, Estrella Villamizar, Sheila M. Walsh, Cory Walter, Ernesto Weil, Ernest H. Williams, Kimberly Woody Roberson, Yusri Yusuf

**Affiliations:** 1 Coral Reef Watch, National Oceanic and Atmospheric Administration, Silver Spring, Maryland, United States of America; 2 NOAA Coral Reef Watch, IM Systems Group, Silver Spring, Maryland, United States of America; 3 NOAA Coral Reef Watch, ReefSense Pty. Ltd., Townsville, Queensland, Australia; 4 School of Engineering and Physical Sciences, James Cook University, Townsville, Queensland, Australia; 5 Center for Marine and Environmental Studies, University of the Virgin Islands, St. Thomas, United States Virgin Islands, United States of America; 6 Parque Nacional Arrecifes de Cozumel, Cozumel, México; 7 School of Environmental Sciences, University of East Anglia, Norwich, United Kingdom; 8 CSA South, Inc., Dania Beach, Florida, United States of America; 9 Center for Coral Reef Research, Mote Marine Laboratory, Summerland Key, Florida, United States of America; 10 Instituto de Tecnología y Ciencias Marinas, Universidad Simón Bolívar, Caracas, Venezuela; 11 Laboratoire de Biologie Marine, Université des Antilles et de la Guyane, Pointe-à-Pitre, Guadeloupe, France; 12 Khaled bin Sultan Living Oceans Foundation, Landover, Maryland, United States of America; 13 Department of Biology, University of Puerto Rico, Mayagüez, Puerto Rico, United States of America; 14 Global Vision International and Amigos de Sian Ka'an Asociación Civil, Playa del Carmen, Quintana Roo, México; 15 Office of National Marine Sanctuaries, National Oceanic and Atmospheric Administration, Key West, Florida, United States of America; 16 Center for Marine Science, University of North Carolina at Wilmington, Key Largo, Florida, United States of America; 17 Luton Institute for Research in the Applied Natural Sciences, University of Bedfordshire, Luton, United Kingdom; 18 Buccoo Reef Trust, Carnbee, Trinidad and Tobago; 19 Centro de Investigaciones Marinas, Universidad de la Habana, Habana, Cuba; 20 Universidad del Magdalena, Santa Marta, Colombia; 21 Griffith School of Environment and Australian Rivers Institute, Griffith University, Nathan, Queensland, Australia; 22 Marine and Atmospheric Science Program, University of Miami, Coral Gables, Florida, United States of America; 23 Insituto de Investigaciones Marinas y Costeras (INVEMAR), Santa Marta, Colombia; 24 National Coral Reef Institute, Nova Southeastern University, Dania Beach, Florida, United States of America; 25 Rosenstiel School of Marine and Atmospheric Science, University of Miami, Virginia Key, Florida, United States of America; 26 Conservation and Fisheries Department, Road Town, Tortola, British Virgin Islands, United Kingdom; 27 Smithsonian Tropical Research Institute, Balboa, Panamá; 28 Atlantic Oceanographic and Meteorological Laboratory, National Oceanic and Atmospheric Administration, Miami, Florida, United States of America; 29 Center for Applied Tropical Ecology and Conservation, University of Puerto Rico, San Juan, Puerto Rico; 30 Marine Spatial Ecology Lab, University of Exeter, Exeter, United Kingdom; 31 Center for Coastal Monitoring and Assessment, National Oceanic and Atmospheric Administration, Silver Spring, Maryland, United States of America; 32 Bermuda Institute of Ocean Sciences, St George's, Bermuda; 33 Instituto de Ciencias del Mar y Limnología, Universidad Nacional Autónoma de México, Cancún, Quintana Roo, México; 34 Biology Department, Boston University, Boston, Massachusetts, United States of America; 35 Global Change Institute, University of Queensland, Brisbane, Queensland, Australia; 36 The Nature Conservancy, Sugarloaf Key, Florida, United States of America; 37 Ocean Research and Education Foundation Inc., Coral Gables, Florida, United States of America; 38 Department of Life Sciences, University of the West Indies, St. Augustine, Trinidad and Tobago; 39 Research School of Earth Science, Australian National University, Canberra, Australian Capital Territory, Australia; 40 Central Caribbean Marine Institute and Kean University, Union, New Jersey, United States of America; 41 Observatoire du Milieu Marin Martiniquais, Fort de France, Martinique, France; 42 Reef Check, Pacific Palisades, California, United States of America; 43 South Florida/Caribbean Network, Virgin Islands National Park, St. John, United States Virgin Islands, United States of America; 44 Perry Institute for Marine Science, Jupiter, Florida, United States of America; 45 Biological Sciences Department, Florida Institute of Technology, Melbourne, Florida, United States of America; 46 Corporación para el Desarrollo Sostenible del Archipiélago de San Andrés, Providencia y Santa Catalina (CORALINA), San Andrés Isla, Colombia; 47 Centre for Resource Management and Environmental Studies, University of the West Indies, Cave Hill, Barbados; 48 St. Croix East End Marine Park, Department of Planning and Natural Resources, Christiansted, United States Virgin Islands, United States of America; 49 Department of Ecology and Evolutionary Biology, University of California Los Angeles, Los Angeles, California, United States of America; 50 Departamento Ciencias Biologicas, Universidad de los Andes, Bogotá, Colombia; 51 Flower Garden Banks National Marine Sanctuary, National Oceanic and Atmospheric Administration, Galveston, Texas, United States of America; 52 Northeast Fisheries Science Center, National Oceanic and Atmospheric Administration, Woods Hole, Massachusetts, United States of America; 53 Institute for Tropical Marine Ecology Inc., Roseau, Dominica; 54 Instituto de Zoología Tropical, Universidad Central de Venezuela, Caracas, Venezuela; 55 Environmental Change Initiative, Brown University, Providence, Rhode Island, United States of America; 56 ReefBase and Institute of Oceanography, Universiti Malaysia Terengganu, Kuala Terengganu, Malaysia; Dalhousie University, Canada

## Abstract

**Background:**

The rising temperature of the world's oceans has become a major threat to coral reefs globally as the severity and frequency of mass coral bleaching and mortality events increase. In 2005, high ocean temperatures in the tropical Atlantic and Caribbean resulted in the most severe bleaching event ever recorded in the basin.

**Methodology/Principal Findings:**

Satellite-based tools provided warnings for coral reef managers and scientists, guiding both the timing and location of researchers' field observations as anomalously warm conditions developed and spread across the greater Caribbean region from June to October 2005. Field surveys of bleaching and mortality exceeded prior efforts in detail and extent, and provided a new standard for documenting the effects of bleaching and for testing nowcast and forecast products. Collaborators from 22 countries undertook the most comprehensive documentation of basin-scale bleaching to date and found that over 80% of corals bleached and over 40% died at many sites. The most severe bleaching coincided with waters nearest a western Atlantic warm pool that was centered off the northern end of the Lesser Antilles.

**Conclusions/Significance:**

Thermal stress during the 2005 event exceeded any observed from the Caribbean in the prior 20 years, and regionally-averaged temperatures were the warmest in over 150 years. Comparison of satellite data against field surveys demonstrated a significant predictive relationship between accumulated heat stress (measured using NOAA Coral Reef Watch's Degree Heating Weeks) and bleaching intensity. This severe, widespread bleaching and mortality will undoubtedly have long-term consequences for reef ecosystems and suggests a troubled future for tropical marine ecosystems under a warming climate.

## Introduction

Coral bleaching has become a major threat to coral reef ecosystems worldwide [1]. Bleaching occurs when stress to the coral-algal symbiosis causes corals to expel their endosymbiotic algae (zooxanthellae) and, if prolonged or particularly severe, may result in partial or complete coral mortality [2]. While many sources of stress have caused corals to bleach, “mass” coral bleaching (at scales of 100 km or more) has only occurred when anomalously warm ocean temperatures, typically coupled with high subsurface light levels, exceeded corals' physiological tolerances. This was observed during recent major El Niño-Southern Oscillation events (*e.g.*, 1982–83 [3], 1997–98 [4], and 2002 [5]) and verified by laboratory experiments [6], [7]. These bleaching events caused coral death at numerous sites around the world, with impacts on reef habitats, structures, and biodiversity that lasted a decade or more [8], [9].

From June to October 2005, a warm-water anomaly developed across the tropical Atlantic Ocean and greater Caribbean Sea region. Satellite-based sea surface temperature (SST) observations from the U.S. National Oceanic and Atmospheric Administration (NOAA) [10] detected a large region of warming ocean temperatures that reached a maximum anomaly of +1.2°C vs. the long-term mean when averaged across all Caribbean reef sites. Elevated temperatures persisted for many weeks and helped fuel the most active Atlantic hurricane season on record [11] and the most severe and extensive mass coral bleaching event observed in the Caribbean.

NOAA's Coral Reef Watch (CRW) developed and maintains a suite of operational satellite sea surface temperature (SST)-based products that provide coral bleaching nowcasts and alerts [10]. HotSpots are positive SST anomalies beyond coral's tolerance level that reflect instantaneous thermal stress and Degree Heating Weeks (DHWs) providing a a measure of sustained thermal stress during a 12-week period. In 2005, NOAA warned coral reef managers and scientists of anomalously warm conditions as they developed and spread across the greater Caribbean region. The maps of sustained thermal stress indicated levels that could cause mass coral bleaching and significant mortality, and guided both the timing and location of researchers' field observations. As a result, collaborators from 22 countries undertook the most comprehensive documentation of basin-scale bleaching to date.

## Results

**Figure 1:**
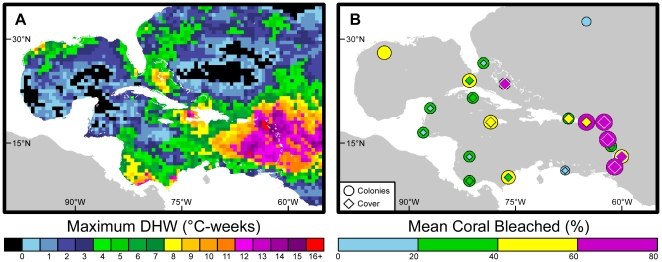
Thermal stress and bleaching during the 2005 Caribbean bleaching event. (A) Maximum NOAA Coral Reef Watch Degree Heating Week (DHW) values showing the highest thermal stress recorded at each 0.5-degree pixel during 2005. Values ≥4°C-weeks typically resulted in significant bleaching; ≥8°C-weeks typically resulted in widespread bleaching and significant mortality. (B) Jurisdictional means of coral bleached; marker color and size denote the severity measured as either percent live coral colonies (circles) or cover (diamonds).

**Figure 2:**
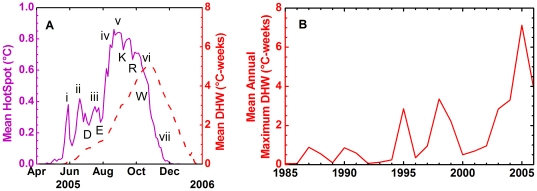
Temporal patterns of thermal stress in the Caribbean. Average of satellite-derived anomaly and thermal stress indices from the 0.5-degree pixels containing or nearest to reefs in the Caribbean (bounded by 35°N, 55°W, and the coast of the Americas). (A) NOAA coral bleaching HotSpots (purple) and DHW (red) in 2005. See results for explanations of (i)–(vii). Letters D–W refer to the major hurricanes of 2005: **D**ennis, **E**mily, **K**atrina, **R**ita and **W**ilma. (B) Average of annual maximum thermal stress (DHW) values during 1985–2006. Significant coral bleaching was reported during periods with average thermal stress above 0.5°C-weeks, and was especially widespread in 1995, 1998, and 2005.

NOAA measured sustained thermal stress in 2005 that exceeded 16°C-weeks in some regions, far greater than the thresholds that have usually been associated with the onset of mass coral bleaching (DHW  = 4°C-weeks) and mortality (DHW  = 8°C-weeks) [10] (Figure 1A). As the event developed, water temperatures rose across the basin to levels well above normal (i.e., long-term average condition, Figure 2A) and remained above normal for more than 7 months, resulting in especially severe thermal stress at the northern end of the Lesser Antilles (Figures 1A, S1, S2). Analysis of retrospective satellite data showed that the sustained thermal stress in the Caribbean during 2005 was more intense than any of the previous 20 years (Figure 2B).

The timeline for the geographic spread of the 2005 Caribbean thermal stress was decomposed into seven major phases as identified in Figure 2A: in late-May (i), thermal stress was observed off South America; by mid-June (ii), the Caribbean coast from Colombia to Nicaragua experienced elevated temperatures. In July (iii), the western Caribbean warm anomalies persisted from Panama to Nicaragua and the extreme western Atlantic east of the Lesser Antilles began to warm. Through August (iv), reefs in the Gulf of Mexico, Florida, the Bahamas, and the Lesser Antilles experienced high levels of stress, while low-level stress was present across most of the Caribbean. In September (v), the center of warming progressed along Cuba, Hispaniola, and Puerto Rico to the Leeward and Windward Islands while low-level stress persisted throughout the Caribbean. By October (vi), thermal stress subsided in the Gulf of Mexico; however, warm anomalies intensified in the Windward Islands and expanded into the southern Caribbean. As the region of maximum warming moved southward during November (vii), waters around the northern Antilles cooled; low-level heat stress affected the northern coast of South America until it mostly dissipated around the end of December 2005 [12].

After initial reports of bleaching in Colombia in June, CRW distributed alerts via the Internet as the thermal stress spread and intensified. Teams (represented by the many co-authors on this paper) deployed throughout the region to monitor the bleaching event as it developed, and subsequently to monitor coral mortality. Coral bleaching, other disease conditions, and mortality extended across the entire Caribbean – bleaching was especially intense along the Antilles (Figure 1B), and was observed in most Caribbean coral species in depths to 40 m. Over 3600 field surveys were recorded from 28 jurisdictions (*i.e.*, states, territories) in 22 countries (Figure S3). After quality control, data from 2575 field surveys were used in the bleaching analysis and 1077 were used in mortality studies. Surveys were grouped by 0.5-degree pixel at twice-weekly time intervals to allow satellite data and field surveys to be analyzed at comparable scales.

Several species and sites were reported to bleach for the first time, including: the first known bleaching at Saba; the first documented mass bleaching of the Flower Garden Banks, including at least partial bleaching of all *Millepora alcicornis* and *Montastraea cavernosa* colonies; and the first reported mass bleaching of *Acropora palmata* in Virgin Islands National Park (VINP), a species listed as threatened under the US Endangered Species Act (ESA) since 2006 [13].

Surveys conducted from the peak of thermal stress through January 2007 were analyzed to assess coral mortality. Detailed and repeated monitoring revealed that a combination of bleaching and other disease outbreaks killed coral colonies stressed by high temperatures [12], [14], [15]. Some researchers identified continued mortality as late as October 2007 [15], beyond which it was difficult to attribute further mortalities to this bleaching event with sufficient certainty. In parts of the Caribbean, temperatures remained anomalously high during the boreal winter-spring and into mid-2006, although remaining below the bleaching threshold. Many corals remained bleached, and disease and mortality continued through much of 2006. Mortality exceeded 50% in several locations and made this the worst case of thermal stress-related mortality documented in the Caribbean to date, and one of the worst cases globally [16]. The pattern of high thermal stress followed by subsequent mortality across much of the Caribbean was consistent with the pattern seen since the 1980s and 1990s in the Florida Keys, where outbreaks of other diseases have frequently been seen in years that followed thermal stress and bleaching [12].

In the Florida Keys in 2005, bleaching was less severe than in the Caribbean proper. However, increased temperatures were quickly followed by a loss of resistance to pathogenic disease and an increased abundance of microbial pathogens in *A. palmata* [17], perhaps explaining the high incidence of disease following the thermal stress by either contagious or opportunistic pathogens [18]. A longitudinal study of cohorts of corals in this region also revealed that more extensively bleached corals were more susceptible to disease outbreaks [19]. In VINP, video surveys of permanent transects revealed that mortality occurred in colonies due to bleaching, and in colonies that showed disease symptoms either during bleaching, after recovery from bleaching, or even without visible bleaching [14]. Frequent monitoring of *A. palmata* also revealed that bleached corals suffered greater disease-associated mortality than unbleached colonies, indicating that disease severity was dependent on host susceptibility [13]. In Barbados, corals remained bleached for 8 months or longer before dying [20], [21]; even a year after temperatures dropped below bleaching thresholds, some corals remained bleached or pale at many sites, particularly within the important reef-builders of the *Montastraea annularis* species complex, which are now under consideration for ESA protection [21]. Fortunately, thermal stress was lower off Venezuela (including Los Roques, Aruba, Bonaire, and Curaçao) and bleaching, disease, and mortality were limited with no long-term community decline [22].

**Figure 3:**
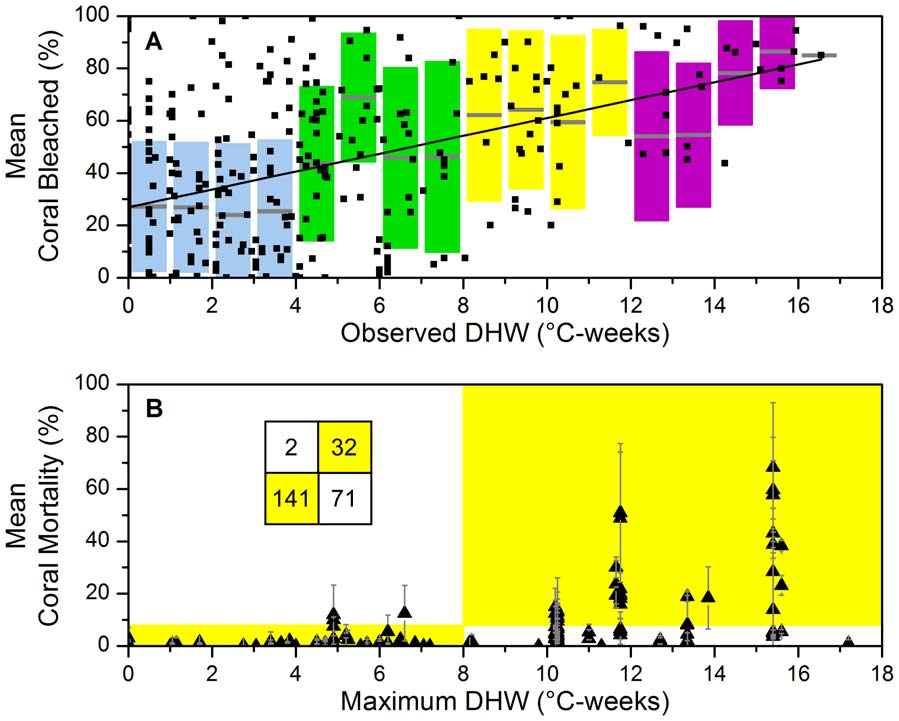
Mean coral bleaching and mortality versus thermal stress. (A) Small squares represent mean percent coral bleached (by area or colony) for each 0.5-degree pixel and twice-weekly time period plotted against observed DHW value. Solid line indicates significant linear regression (*slope*  = 3.41, *intercept*  = 26.94, *DF*  = 359, *p*<0.0001, *r^2^* = 0.24). Colored bars indicate mean (gray bar) and standard deviation of all surveys binned at 1°C-week intervals; colors correspond to low bleaching risk (DHW <4, blue), moderate risk (DHW ≥4, green), high bleaching and mortality risk (DHW ≥8, yellow), and very high risk (DHW ≥12, purple). (B) Triangles represent mean percent coral mortality (± standard deviation) reported during 25-Jul-2005 to 20-Jan-2007, plotted against the 2005 maximum DHW value recorded for each 0.5-degree pixel. Yellow and white areas correspond to the inset box where values indicate number of data points in each quadrant (quadrants defined as 0≤ DHW <8 and 0≤ mortality <8%; 0≤ DHW <8 and 8%≤ mortality; 8≤ DHW and 0≤ mortality <8%; 8≤ DHW and 8%≤ mortality).

Comparison of satellite data with field surveys demonstrated a strong coherence between thermal stress (Figure 1A) and widespread bleaching (Figure 1B, 3A) and mortality (Figure 3B). However, significant variability was seen in the severity of coral bleaching among reefs within each 0.5-degree satellite pixel, presumably due to variations in local conditions (*e.g.*, hydrodynamics, light, community composition). Consistent with CRW's previously established bleaching levels, significant coral bleaching began near 4°C-weeks (Alert Level 1, Figure 3A), with widespread mass bleaching and significant mortality occurring above 8°C-weeks (Alert Level 2, Figure 3B) [10]. However, bleaching also occurred at sites experiencing maximum stress levels below 4°C-weeks, indicating that either the 4°C-weeks threshold may have been conservative or the 0.5-degree spatial resolution failed to detect localized high temperatures. Bleaching has been reported to depend on numerous local factors, including light level, temperature variability, and past thermal stress history [23]. These could have influenced bleaching variability within and among reefs in each 0.5-degree pixel as well. Coral mortality in 2005 was highest in jurisdictions in the northern and central Lesser Antilles where stress exceeded 10°C-weeks (Figure 1A).

In the areas where thermal stress levels were less than 8°C-weeks, significant mortality was rare (2 of 143 surveys, <1.5%; Figure 3B). Above this threshold, significant mortality was observed in 31% of events. It was likely that local conditions at scales finer than those detected by satellite observations increased or decreased the effect of the thermal stress within and among reefs at the sub-pixel scale (*e.g.*, coral community structure, small-scale hydrodynamics, past bleaching; the analysis of which were beyond the scope of this study). Despite local variability, thermal stress values exceeding approximately 8°C-weeks successfully predicted significant mortality. Thermal stress of this magnitude should be weighed carefully by reef managers. In 2005, little mortality was seen below 8°C-weeks of thermal stress while above it there was an ecologically important 1-in-3 risk of mortality. The slow rate of recovery seen in Caribbean reefs [16], [24], [25] suggests that such high levels of mortality may determine the fate of coral reef ecosystems in this region for decades to come.

## Discussion

**Figure 4:**
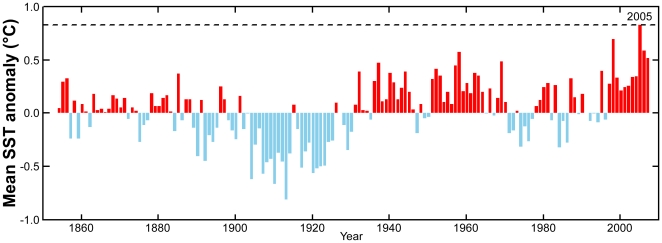
Long-term temperature record in the Caribbean. Temperature anomalies for 2.0-degree reef pixels in the tropical Caribbean computed using the NOAA Extended Reconstructed Sea Surface Temperature (ERSST) dataset. Anomalies were plotted relative to 1901–2000. The dashed line indicates the 2005 value.

Unlike many past Caribbean bleaching years, strong tropical climate forcing was only a minor driver of Caribbean SSTs in 2005. In their analysis of temperature anomalies across the tropical North Atlantic in 2005, Trenberth and Shea [26] indicated that half of the warming (0.45°C of the 0.9°C anomaly *vs*. a 1901–1970 baseline) was attributable to monotonic climate change, while only 0.2°C was attributable to the weak 2004–05 El Niño, and even less to the Atlantic Multi-decadal Oscillation (<0.1°C). Despite the lack of strong tropical forcing, 2005 fell among the warmest years on record [11]. NOAA’s Extended Reconstructed SST product [27], [28] showed that average ocean temperatures during the July-October period for the Caribbean exceeded temperatures seen at any time during the prior 150 years (Figure 4). Anticipated future warming of ocean waters [29] is expected to increase the likelihood of future Caribbean bleaching events [30].

**Figure 5:**
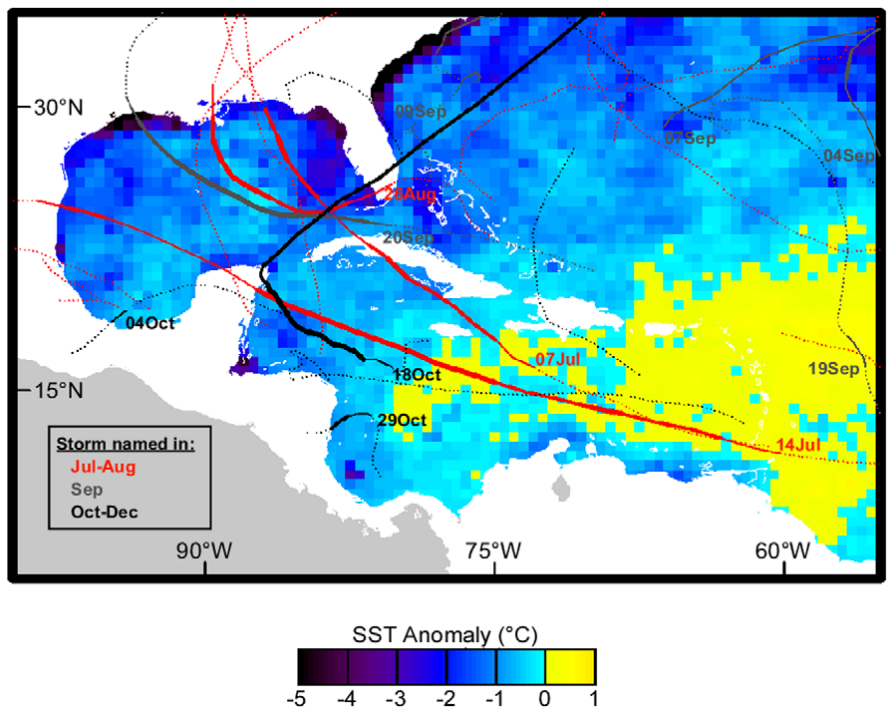
Thermal stress and hurricanes during the 2005 Caribbean bleaching event. Minimum observed SST anomaly for May-December 2005, overlaid with storm tracks (solid: hurricane, thickness denotes strength category; dotted: tropical storm; red: June-August; gray: September; black: October-December). Dates indicate initial date of hurricane formation. The large yellow region in the eastern Caribbean remained warmer than usual throughout this period.

High ocean temperature also contributed to the record 2005 hurricane season [26] that damaged coral reefs in Jamaica, Cuba, the Yucatan, Flower Garden Banks, and the Florida Keys [12] as well as causing major damage to communities and loss of human life. Hurricanes have been observed to cause mechanical damage to coral reefs, including damaging coral tissue and dislodging colonies, weakening corals in ways than could slow recovery following bleaching, and contributing to long-term ecosystem decline [12]. However, hurricanes that pass within several hundred kilometers of coral reefs have been shown to cool anomalously warm SSTs below bleaching thresholds, and were probably significant in reducing thermal stress and preventing more severe bleaching in the Florida Keys in 2005 [12], [31]. The absence of such cooling by tropical cyclones in the Leeward Islands (Figure 5) most likely contributed to the extreme warming, bleaching, and mortality seen there. The major hurricanes that cooled waters around the Florida Keys in 2005 (**D**ennis, **E**mily, **K**atrina, **R**ita, **W**ilma) were strong enough to reduce the Caribbean-average HotSpots (Figure 2A).

Many Caribbean reefs have changed dramatically since the early 20^th^ century as a result of a wide array of human disturbances [32], [33]. It is unlikely that natural climate variability was the cause of declines in Caribbean reefs during recent decades, as coral reef community composition had remained remarkably stable for the prior 220,000 years [34]. While bleaching is far from the only cause of reef decline in the Caribbean, the repeated coral bleaching events since the 1980s have been strongly attributed to anthropogenic climate change [1]. The mass bleaching and mortality from the 2005 warming further disturbed Caribbean ecosystems that were already under assault [12], [33]. Coral bleaching is expected to be an even greater threat to coral reefs in the future [30], [35].

Mass coral bleaching from thermal stress, followed by outbreaks of contagious or opportunistic diseases [18], [36], [37], have become a threat common to coral reefs globally. Bleaching and mortality such as that seen in the Caribbean in 2005 will undoubtedly have long-term consequences for Caribbean coral reefs, as these corals have shown very slow rates of recovery to mortality from mass bleaching [16]. This means that any future bleaching is likely to add to the damage caused in 2005, just as the 2005 event continued the decline of reefs that have suffered past mortality from bleaching, disease, and local stressors. As this paper went to press in 2010, major bleaching was again striking reefs in the Caribbean, in some places worse than in 2005. Major bleaching events have returned to the Caribbean every five years or less, and with growing intensity (Figure 2B). With no real sign of recovery after bleaching in Caribbean reefs [16], these repeated events are likely to have caused reef decline that will extend beyond our lifetimes.

The data presented here will aid researchers and resource managers as they develop actions to protect reefs against the thermal stress anticipated in coming decades [38], especially as new studies identify ways in which reductions of other sources of stress can increase reef resilience to climate change [24], [39], [40], [41]. As global ocean temperatures continue to rise, policy makers will need to address anthropogenic climate change, and managers will have to take concerted efforts to enhance the resilience of coral reefs for us to have hope of preventing dramatic losses of valuable coral reef resources.

## Materials and Methods

NOAA Coral Reef Watch (CRW) thermal stress products used in this study were based on nighttime-only Advanced Very High Resolution Radiometer (AVHRR) sea surface temperature (SST) data from sensors aboard operational NOAA Polar-Orbiting Environmental Satellites (POES), produced in near-real-time at 0.5-degree (50-km) spatial resolution. SST anomalies compared the measured temperature with the expected value at that time of year for each pixel. HotSpots were computed as positive anomalies above the mean temperature of the climatologically warmest month at each satellite data pixel, based on the NOAA operational climatology from years 1985–1990 and 1993. Degree Heating Weeks (DHWs) for any given time accumulated HotSpot values ≥1°C over the preceding 12-week period [10]. The satellite-derived quantities calculated for this paper (Table S1) at each reef pixel surveyed included: the date of first issuance of Bleaching Watch alert (HotSpot >0°C); the value of maximum DHW (°C-weeks) experienced during the event; and the date when temperatures dropped below stressful levels (HotSpot  = 0).

The DHW map (Figure 1A) included values in coastal regions that were masked as land in the operational CRW products. For the purpose of this figure only, the coastal values were inferred using kriging, a common statistical technique [42]. However, all data used for the subsequent analyses (Figure 2A) and comparison with field data (Figure 3) were retrieved from NOAA operational products. Spatial averaging of satellite metrics (Figure 2A, S1) was performed using the original operational data from the greater Caribbean pixels containing, or nearest to, coral reef locations within the region [100W-55W, 5N-35N].

CRW operational products were first made available on 12-Sep-2000. The 22-year time series of annual maximum DHW (Figure 2B) was produced from a retrospective suite of products that emulated the CRW near-real-time operational product [1] for the period 1985–2006 using data from the Pathfinder Version 5.0 SST dataset [43]. Spatial averaging was undertaken using the same pixels used for the operational data.

Field surveys of coral bleaching and mortality included at least the following quantitative data: 1) measures of coral bleaching as coral cover bleached (%), number of coral colonies bleached (*n*) and total number of colonies surveyed (*N*), or both; and/or 2) measures of coral mortality as coral cover dead (%), number of coral colonies dead (*n*) and total number of colonies surveyed (*N*), or both; 3) average observation depth (m); 4) observation date; and 5) observation location, including latitude, longitude, and reef site name. Data were quality controlled to exclude observations that met any of the following criteria: 1) bleaching observations taken before the onset of thermal stress (first issuance of Bleaching Watch alert); 2) bleaching observations taken after subsidence of thermal stress, defined as the 90^th^ day following the date of the last No Stress alert in 2005; and 3) mortality observations taken before the maximum DHW value occurred in 2005. Multiple observations (quadrats or transects) taken at any reef site on the same date and depth (±5 m) were combined into a single survey of either means of percent cover data or proportion of the number of colonies surveyed. The 2575 bleaching surveys used in this analysis (Table S1, Figures 1b, 3a) spanned the period 3-Jun-2005 through 13-Feb-2006. The 1077 mortality surveys used to estimate mortality associated with the thermal stress event (Table S1, Figure 3b) were conducted during 25-Jul-2005 to 20-Jan-2007. In some cases, multiple surveys from within 0.5-degree pixels were conducted on multiple dates during the period between the onset of thermal stress and 90 days after thermal stress subsided. However, these were almost always either new surveys at different sites or different, random sets of observations within transects. There were insufficient cases of repeated surveys of the same transect to analyze how bleaching changed through time either during the warming or cooling phases of the event. However, a few resurveyed sites did show some degree of recovery after the peak of bleaching. Reports detailing change through time at individual sites have been published and continue to be published elsewhere [15], [20], [21], [22].

As the multiple researchers who took part in this paper used a variety of methods, the work presented here was a meta-analysis of surveys conducted by numerous research institutions during the 2005 bleaching event. The techniques used were all highly comparable, well-accepted field methods. The authors assumed that differences among techniques were randomly distributed with respect to thermal stress. Past comparisons among coral reef survey methods have demonstrated that while there are some biases among methods, most provide comparable results when comparing among similar types of observation such as percent coral cover or disturbance [44], [45]. It is important to note that the percentage of colonies bleached was often higher than the percentage of cover bleached because (1) small colonies bleached more often than large colonies; and/or (2) both partially- and wholly-bleached colonies were counted as bleached in some survey methodologies. However, a statistical comparison of the linear regressions of percent cover bleached and percent colonies bleached vs. thermal stress (Figure S4) found no significant differences between the slopes of the two parameters (cover  = 3.91±0.89 vs. colonies  = 3.43±0.70, expressed as slope ±95% confidence interval). This supported the assumption that the different observation methods provided comparable results for this meta-analysis. Also, because any visible bleaching probably indicated a loss of most of the zooxanthellae originally present [46], it was appropriate to include any degree of bleaching, from pale and partially bleached to fully bleached colonies, as an indicator of significant stress in the corals. The same applies to partial and complete mortality as either indicated a thermal stress response resulting in mortality due to bleaching or various other diseases. Therefore, partial and complete bleaching and partial and complete mortality of corals were combined as observations of bleaching and mortality, respectively.

Mortality data included only corals that expert observers determined had recently died; however, the actual cause of mortality typically was not identifiable. An analysis of reefs in the region showed that 4% recent mortality normally existed as a background level during surveys in years lacking any major disturbance [47]. This 4% background level of mortality was then considered in establishing the level of mortality considered significant in Figure 3B. As was expected for an accumulated variable such as mortality, total percent coral mortality did rise slowly with time after the thermal stress. The observers did not feel that they could accurately separate mortality due to the 2005 bleaching event from other causes beyond 20-Jan-2007, thus determining the end date of the data used. Finally, the data density and non-random distribution of data submissions did not permit the standardization of mortality as a function of time since observation.

Operational satellite products from the co-located (or next-nearest) satellite pixel were compared with all field observations (Figure S3). A linear regression was used to compare mean coral bleaching (combined cover and colonies datasets) with thermal stress (observed DHW at the time of the survey). For surveys that occurred after the peak of thermal stress, the observed DHW may have declined from the maximum thermal stress experienced at that location. This could have resulted in a level of bleaching greater than that expected from the observed DHW against which it was compared. Each data point represented the average of all surveys for a given 0.5-degree pixel conducted during the twice-weekly time period (temporal resolution) of the satellite data, plotted against the DHW value observed for that pixel and time period. The relationship between observed DHW and percent coral bleached was highly significant (*slope*  = 3.41, *intercept*  = 26.94, *DF*  = 359, *p*<0.0001, *r^2^* = 0.24). Given the variability of monitoring techniques employed, sampling errors within each technique, and local factors at individual reef sites (*e.g.*, shading, ponding), the explicative power of the satellite metric (*r^2^* = 0.24 for percent coral bleached) supported the predictive relationship between the thermal stress monitored by CRW satellite products and the observed bleaching during this event. However, it was clear that inclusion of other information, including higher spatial resolution SST-based products, may further refine bleaching predictions in the future.

For consistency, mortality data were considered only for observations after the peak of the thermal stress event (*i.e.*, the maximum DHW) within a pixel and were analyzed against the maximum thermal stress (Figure 3B). For this study, the threshold for significant mortality was defined where the observed value was twice the regional baseline mortality; *i.e.*, 8%. The nature of this analysis was very broad, combining field datasets across time, space, and survey methodology. No attempt was made to separate mortality induced by bleaching from that resulting by other diseases as both were related to thermal stress [14], [48]. The results showed strong predictive power. However, thermal stress was far from a perfect predictor of mortality as local variability in the response of corals at and within individual reef sites likely played a critical role due to differences in circulation, shading, past thermal stress, and other factors that may have conferred local resilience.

Hurricanes extract heat from the upper ocean and induce vertical mixing. Both mechanisms have been shown to reduce the high temperatures of surface waters that cause coral bleaching [12], [31]. While 2005 was a record hurricane season, none passed near the Lesser Antilles where some of the highest bleaching and mortality were observed. This can be seen in hurricane tracks (Figure 5) acquired from the National Hurricane Center (www.nhc.noaa.gov). Surface temperatures in this region remained above climatological values throughout the May-December period, with no respite from thermal stress (Figures 1A, S2).

The NOAA Extended Reconstructed SST data [27], [28] used in Figure 4 were averaged across reef-containing pixels (2-degree resolution) within the region [91W-55W, 5N-35N] and are presented as anomalies relative to the 1901-2000 mean.

## Supporting Information

Figure S1 Sea surface temperature during the 2005 Caribbean bleaching event. Sea surface temperature (SST) averaged across the 0.5-degree pixels that contained or were nearest Caribbean reef locations (bounded by 35°N, 55°W, 5°N and the coast of the Americas). The ‘+’ symbols indicate the average climatological temperature during each month and the dashed line shows the maximum of these, an indication of the expected warmest (usually summer) temperature. The SST trace shows that, on average, temperatures around Caribbean reefs exceeded climatological values by close to 1°C for a period of more than four months. The magnitude and extended duration of the basin-wide thermal anomaly resulted in widespread coral bleaching and lowered the ability of corals to resist other disease conditions.
(0.69 MB ZIP)

Figure S2 Animation of the development of thermal stress during the 2005 Caribbean bleaching event, measured using NOAA Coral Reef Watch Degree Heating Week product from 4 June 2005 to 14 February 2006 with a pause during the peak of the event at 28 October 2005.
(5.54 MB TIF)

Figure S3 Locations of 2575 bleaching surveys submitted from sites across the greater Caribbean region. Colors denote number of surveys at each of the 1212 sites. See Table S1 for location details.
(0.18 MB TIF)

Figure S4 Comparison of bleaching survey methods. All observations of percent coral colonies (gray circles) and cover (black diamonds) are plotted versus observed Degree Heating Week (DHW). Linear regressions for colonies (gray line) and cover (black line) were highly significant (cover slope  = 3.91, intercept  = 19.99, df  = 212, p<0.0001, r^2^ = 0.26; colonies slope  = 3.43, intercept  = 29.46, df  = 304, p<0.0001, r^2^ = 0.24) and indicated no difference in slopes, suggesting comparable results.
(0.08 MB TIF)

Table S1 Complete data record for all survey data used in the analyses. Multiple observations from the same reef site, date and depth (±5 m) were combined as either means of percent cover data or proportion of the number of colonies surveyed to provide 2575 bleaching surveys and 1077 mortality surveys.
(0.17 MB PDF)
