# Supplementary figures and images for: Caribbean Corals in Crisis: Record Thermal Stress, Bleaching, and Mortality in 2005
Source: PLoS One. 2010 Nov 15;5(11):e13969. doi: 10.1371/journal.pone.0013969 (PMC2981599; doi:10.1371/journal.pone.0013969)

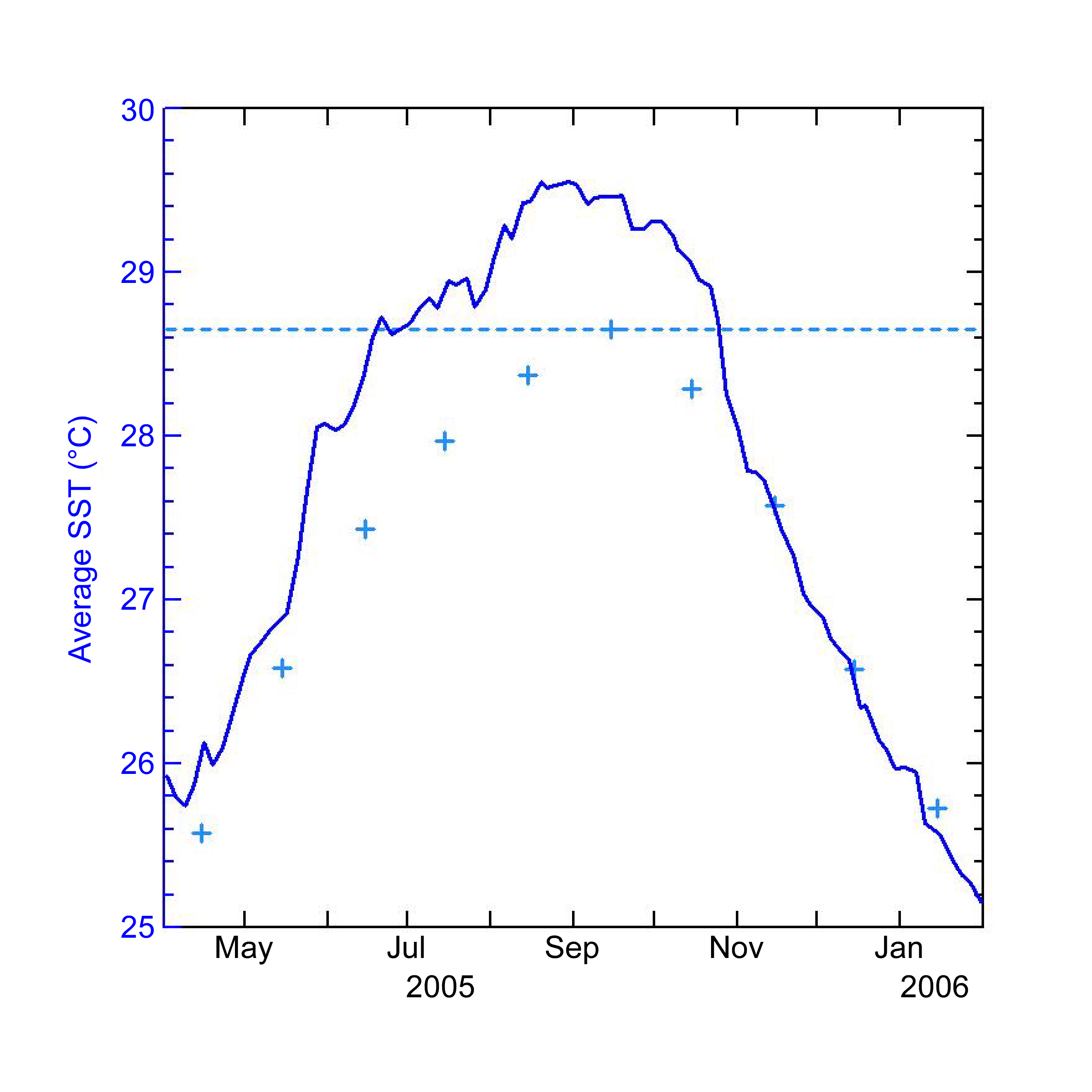

Supplement: Figure S2 — Animation of the development of thermal stress during the 2005 Caribbean bleaching event, measured using NOAA Coral Reef Watch Degree Heating Week product from 4 June 2005 to 14 February 2006 with a pause during the peak of the event at 28 October 2005. (5.54 MB TIF) [file pone.0013969.s002.tif]

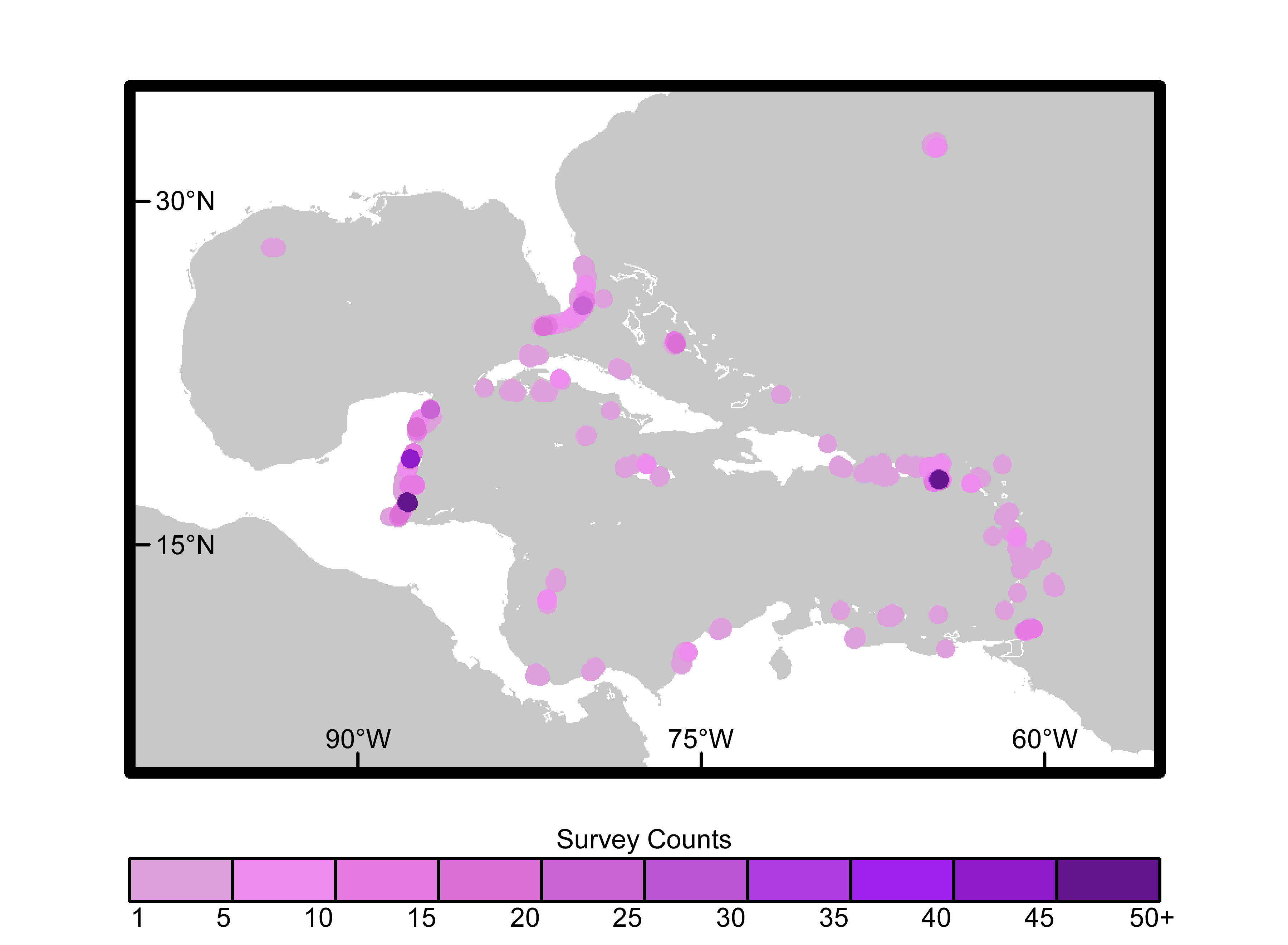

Supplement: Figure S3 — Locations of 2575 bleaching surveys submitted from sites across the greater Caribbean region. Colors denote number of surveys at each of the 1212 sites. See Table S1 for location details. (0.18 MB TIF) [file pone.0013969.s003.tif]
