# Supplementary material for: Caribbean Corals in Crisis: Record Thermal Stress, Bleaching, and Mortality in 2005
Source: PLoS One. 2010 Nov 15;5(11):e13969. doi: 10.1371/journal.pone.0013969 (PMC2981599; doi:10.1371/journal.pone.0013969)
Supplement: Table S1 — Complete data record for all survey data used in the analyses. Multiple observations from the same reef site, date and depth (±5 m) were combined as either means of percent cover data or proportion of the number of colonies surveyed to provide 2575 bleaching surveys and 1077 mortality surveys. (0.17 MB PDF) [file pone.0013969.s005.pdf]

Table S1. Survey data used for analyses. Multiple observations from the same reef site, date and depth (± 5 m) were combined as either means of percent cover data or proportion of the number of colonies surveyed to provide 2575 bleaching surveys and 1077 mortality surveys.

Observation Number	Reef or Site Name	Location	Country	Date (DD-MMM-YY)	Latitude (decimal degrees)	Longitude (decimal degrees)	Primary Contributor	Depth (m)	Cover bleached (%)	Colonies bleached (%)	Cover dead (%)	Colonies dead (%)	Observed DHW (°C-weeks)	Maximum DHW (°C-weeks)
2058	Turtle Rocks	Bimini	Bahamas	17-Aug-05	25.663	-79.309	Reef Check	0.9	17.0%	31.0%			2.85	5.7
2101	Southeast Barracuda Rocks, Gre	Southeast Barracuda Rocks, Gre	Bahamas	29-Aug-05	23.673	-76.202	ReefBase	3	75.0%				3.3	5.25
2102	Northwest Barracuda Rocks, Gr	Northwest Barracuda Rocks, Gr	Bahamas	31-Aug-05	23.718	-76.254	ReefBase	3	75.0%				3.3	5.25
2106	North Norman’s Pond Cay patch	North Norman’s Pond Cay patch	Bahamas	6-Sep-05	23.790	-76.138	ReefBase	6	75.0%				3.85	5.25
1751	Jane's Reef	Exumas	Bahamas	22-Oct-05	23.768	-76.090	Mueller, Erich	2		42.4%			4.4	5.25
1752	Palmata Reef	Exumas	Bahamas	22-Oct-05	23.797	-76.146	Mueller, Erich	4.5		50.0%			4.4	5.25
1753	Palmata Reef	Exumas	Bahamas	22-Oct-05	23.783	-76.099	Mueller, Erich	2		27.6%	5.0%		4.4	5.25
1754	South Perry Reef	Exumas	Bahamas	22-Oct-05	23.775	-76.090	Mueller, Erich	12.5		82.3%			4.4	5.25
1755	North Norman's Pond Cay Patch	Exumas	Bahamas	23-Oct-05	23.790	-76.138	Mueller, Erich	4.5		92.2%		0.0%	4.4	5.25
1756	North Perry Reef	Exumas	Bahamas	23-Oct-05	23.783	-76.101	Mueller, Erich	13.5		79.3%			4.4	5.25
1757	Rainbow Gardens	Exumas	Bahamas	23-Oct-05	23.797	-76.146	Mueller, Erich	4.5		48.8%			4.4	5.25
2329	G-spot	Exuma Cays	Bahamas	28-Oct-05	23.831	-76.198	Samhouri, Jameal	3.65		0.0%			3.9	5.25
2330	G-spot	Exuma Cays	Bahamas	28-Oct-05	23.831	-76.198	Samhouri, Jameal	3.65		50.0%			3.9	5.25
2331	G-spot	Exuma Cays	Bahamas	28-Oct-05	23.831	-76.198	Samhouri, Jameal	3.65		0.0%			3.9	5.25
2332	G-spot	Exuma Cays	Bahamas	28-Oct-05	23.831	-76.198	Samhouri, Jameal	3.65		0.0%			3.9	5.25
2333	G-spot	Exuma Cays	Bahamas	28-Oct-05	23.831	-76.198	Samhouri, Jameal	3.65		0.0%			3.9	5.25
2334	G-spot	Exuma Cays	Bahamas	28-Oct-05	23.831	-76.198	Samhouri, Jameal	3.65		62.5%			3.9	5.25
2335	G-spot	Exuma Cays	Bahamas	28-Oct-05	23.831	-76.198	Samhouri, Jameal	3.65		75.0%			3.9	5.25
2336	G-spot	Exuma Cays	Bahamas	28-Oct-05	23.831	-76.198	Samhouri, Jameal	3.65		0.0%			3.9	5.25
2337	G-spot	Exuma Cays	Bahamas	28-Oct-05	23.831	-76.198	Samhouri, Jameal	3.65		60.0%			3.9	5.25
2338	G-spot	Exuma Cays	Bahamas	28-Oct-05	23.831	-76.198	Samhouri, Jameal	3.65		41.7%			3.9	5.25
2339	G-spot	Exuma Cays	Bahamas	28-Oct-05	23.831	-76.198	Samhouri, Jameal	3.65		0.0%			3.9	5.25
2340	G-spot	Exuma Cays	Bahamas	28-Oct-05	23.831	-76.198	Samhouri, Jameal	3.65		57.1%			3.9	5.25
2341	G-spot	Exuma Cays	Bahamas	28-Oct-05	23.831	-76.198	Samhouri, Jameal	3.65		58.8%			3.9	5.25
2342	Tug & Barge	Exuma Cays	Bahamas	28-Oct-05	23.752	-76.105	Samhouri, Jameal	2.74		0.0%			3.9	5.25
2343	Tug & Barge	Exuma Cays	Bahamas	28-Oct-05	23.752	-76.105	Samhouri, Jameal	2.74		14.3%			3.9	5.25
2344	Tug & Barge	Exuma Cays	Bahamas	28-Oct-05	23.752	-76.105	Samhouri, Jameal	2.74		33.3%			3.9	5.25
2345	Tug & Barge	Exuma Cays	Bahamas	28-Oct-05	23.752	-76.105	Samhouri, Jameal	2.74		5.9%			3.9	5.25
2346	Tug & Barge	Exuma Cays	Bahamas	28-Oct-05	23.752	-76.105	Samhouri, Jameal	2.74		0.0%			3.9	5.25
2347	Tug & Barge	Exuma Cays	Bahamas	28-Oct-05	23.752	-76.105	Samhouri, Jameal	2.74		42.9%			3.9	5.25
2348	Tug & Barge	Exuma Cays	Bahamas	28-Oct-05	23.752	-76.105	Samhouri, Jameal	2.74		13.6%			3.9	5.25
2349	Tug & Barge	Exuma Cays	Bahamas	28-Oct-05	23.752	-76.105	Samhouri, Jameal	2.74		11.5%			3.9	5.25
2350	Tug & Barge	Exuma Cays	Bahamas	28-Oct-05	23.752	-76.105	Samhouri, Jameal	2.74		40.7%			3.9	5.25
2351	Tug & Barge	Exuma Cays	Bahamas	28-Oct-05	23.752	-76.105	Samhouri, Jameal	2.74		7.4%			3.9	5.25
2352	Tug & Barge	Exuma Cays	Bahamas	28-Oct-05	23.752	-76.105	Samhouri, Jameal	2.74		22.6%			3.9	5.25
2353	Tug & Barge	Exuma Cays	Bahamas	28-Oct-05	23.752	-76.105	Samhouri, Jameal	2.74		40.6%			3.9	5.25
2354	Tug & Barge	Exuma Cays	Bahamas	28-Oct-05	23.752	-76.105	Samhouri, Jameal	2.74		18.9%			3.9	5.25
2355	Tug & Barge	Exuma Cays	Bahamas	28-Oct-05	23.752	-76.105	Samhouri, Jameal	2.74		22.5%			3.9	5.25
2356	Tug & Barge	Exuma Cays	Bahamas	28-Oct-05	23.752	-76.105	Samhouri, Jameal	2.74		14.9%			3.9	5.25
2357	Tug & Barge	Exuma Cays	Bahamas	28-Oct-05	23.752	-76.105	Samhouri, Jameal	2.74		33.3%			3.9	5.25
2358	Windsock	Exuma Cays	Bahamas	29-Oct-05	23.743	-76.076	Samhouri, Jameal	3.96		0.0%			3.9	5.25
2359	Windsock	Exuma Cays	Bahamas	29-Oct-05	23.743	-76.076	Samhouri, Jameal	3.96		0.0%			3.9	5.25
2360	Windsock	Exuma Cays	Bahamas	29-Oct-05	23.743	-76.076	Samhouri, Jameal	3.96		50.0%			3.9	5.25
2361	Windsock	Exuma Cays	Bahamas	29-Oct-05	23.743	-76.076	Samhouri, Jameal	3.96		0.0%			3.9	5.25
2362	Windsock	Exuma Cays	Bahamas	29-Oct-05	23.743	-76.076	Samhouri, Jameal	3.96		33.3%			3.9	5.25
2363	Windsock	Exuma Cays	Bahamas	29-Oct-05	23.743	-76.076	Samhouri, Jameal	3.96		0.0%			3.9	5.25
2364	Windsock	Exuma Cays	Bahamas	29-Oct-05	23.743	-76.076	Samhouri, Jameal	3.96		66.7%			3.9	5.25
2365	Windsock	Exuma Cays	Bahamas	29-Oct-05	23.743	-76.076	Samhouri, Jameal	3.96		50.0%			3.9	5.25
2366	Windsock	Exuma Cays	Bahamas	29-Oct-05	23.743	-76.076	Samhouri, Jameal	3.96		83.3%			3.9	5.25
2367	Windsock	Exuma Cays	Bahamas	29-Oct-05	23.743	-76.076	Samhouri, Jameal	3.96		71.4%			3.9	5.25
2368	Windsock	Exuma Cays	Bahamas	29-Oct-05	23.743	-76.076	Samhouri, Jameal	3.96		0.0%			3.9	5.25
2369	Windsock	Exuma Cays	Bahamas	29-Oct-05	23.743	-76.076	Samhouri, Jameal	3.96		8.3%			3.9	5.25
2370	Windsock	Exuma Cays	Bahamas	29-Oct-05	23.743	-76.076	Samhouri, Jameal	3.96		0.0%			3.9	5.25
2371	Windsock	Exuma Cays	Bahamas	29-Oct-05	23.743	-76.076	Samhouri, Jameal	3.96		47.6%			3.9	5.25
2372	Rainbow	Exuma Cays	Bahamas	30-Oct-05	23.797	-76.146	Samhouri, Jameal	7.77		0.0%			3.9	5.25

Observation Number	Reef or Site Name	Location	Country	Date (DD-MMM-YY)	Latitude (decimal degrees)	Longitude (decimal degrees)	Primary Contributor	Depth (m)	Cover bleached (%)	Colonies bleached (%)	Cover dead (%)	Colonies dead (%)	Observed DHW (°C-weeks)	Maximum DHW (°C-weeks)
2373	Rainbow	Exuma Cays	Bahamas	30-Oct-05	23.797	-76.146	Samhouri, Jameal	7.77		50.0%			3.9	5.25
2374	Rainbow	Exuma Cays	Bahamas	30-Oct-05	23.797	-76.146	Samhouri, Jameal	7.77		0.0%			3.9	5.25
2375	Rainbow	Exuma Cays	Bahamas	30-Oct-05	23.797	-76.146	Samhouri, Jameal	7.77		0.0%			3.9	5.25
2376	Rainbow	Exuma Cays	Bahamas	30-Oct-05	23.797	-76.146	Samhouri, Jameal	7.77		25.0%			3.9	5.25
2377	Rainbow	Exuma Cays	Bahamas	30-Oct-05	23.797	-76.146	Samhouri, Jameal	7.77		0.0%			3.9	5.25
2378	Rainbow	Exuma Cays	Bahamas	30-Oct-05	23.797	-76.146	Samhouri, Jameal	7.77		0.0%			3.9	5.25
2379	Rainbow	Exuma Cays	Bahamas	30-Oct-05	23.797	-76.146	Samhouri, Jameal	7.77		33.3%			3.9	5.25
2380	Rainbow	Exuma Cays	Bahamas	30-Oct-05	23.797	-76.146	Samhouri, Jameal	7.77		14.3%			3.9	5.25
2381	Rainbow	Exuma Cays	Bahamas	30-Oct-05	23.797	-76.146	Samhouri, Jameal	7.77		0.0%			3.9	5.25
2382	Rainbow	Exuma Cays	Bahamas	30-Oct-05	23.797	-76.146	Samhouri, Jameal	7.77		0.0%			3.9	5.25
2383	Rainbow	Exuma Cays	Bahamas	30-Oct-05	23.797	-76.146	Samhouri, Jameal	7.77		11.1%			3.9	5.25
2384	Rainbow	Exuma Cays	Bahamas	30-Oct-05	23.797	-76.146	Samhouri, Jameal	7.77		44.4%			3.9	5.25
2385	Rainbow	Exuma Cays	Bahamas	30-Oct-05	23.797	-76.146	Samhouri, Jameal	7.77		21.4%			3.9	5.25
2386	Rainbow	Exuma Cays	Bahamas	30-Oct-05	23.797	-76.146	Samhouri, Jameal	7.77		26.7%			3.9	5.25
2387	Rainbow	Exuma Cays	Bahamas	30-Oct-05	23.797	-76.146	Samhouri, Jameal	7.77		11.1%			3.9	5.25
2388	Square Rock	Exuma Cays	Bahamas	31-Oct-05	23.710	-76.076	Samhouri, Jameal	3.12		0.0%			3	5.25
2389	Square Rock	Exuma Cays	Bahamas	31-Oct-05	23.710	-76.076	Samhouri, Jameal	3.12		0.0%			3	5.25
2390	Square Rock	Exuma Cays	Bahamas	31-Oct-05	23.710	-76.076	Samhouri, Jameal	3.12		0.0%			3	5.25
2391	Square Rock	Exuma Cays	Bahamas	31-Oct-05	23.710	-76.076	Samhouri, Jameal	3.12		20.0%			3	5.25
2392	Square Rock	Exuma Cays	Bahamas	31-Oct-05	23.710	-76.076	Samhouri, Jameal	3.12		45.5%			3	5.25
2393	Square Rock	Exuma Cays	Bahamas	31-Oct-05	23.710	-76.076	Samhouri, Jameal	3.12		30.8%			3	5.25
2394	Square Rock	Exuma Cays	Bahamas	31-Oct-05	23.710	-76.076	Samhouri, Jameal	3.12		71.4%			3	5.25
2395	Square Rock	Exuma Cays	Bahamas	31-Oct-05	23.710	-76.076	Samhouri, Jameal	3.12		31.3%			3	5.25
2396	Square Rock	Exuma Cays	Bahamas	31-Oct-05	23.710	-76.076	Samhouri, Jameal	3.12		66.7%			3	5.25
2397	Square Rock	Exuma Cays	Bahamas	31-Oct-05	23.710	-76.076	Samhouri, Jameal	3.12		56.5%			3	5.25
2398	Square Rock	Exuma Cays	Bahamas	31-Oct-05	23.710	-76.076	Samhouri, Jameal	3.12		62.5%			3	5.25
2399	Square Rock	Exuma Cays	Bahamas	31-Oct-05	23.710	-76.076	Samhouri, Jameal	3.12		67.9%			3	5.25
2400	Square Rock	Exuma Cays	Bahamas	31-Oct-05	23.710	-76.076	Samhouri, Jameal	3.12		75.8%			3	5.25
2401	Square Rock	Exuma Cays	Bahamas	31-Oct-05	23.710	-76.076	Samhouri, Jameal	3.12		18.2%			3	5.25
2402	Square Rock	Exuma Cays	Bahamas	31-Oct-05	23.710	-76.076	Samhouri, Jameal	3.12		66.7%			3	5.25
2403	Tug & Barge	Exuma Cays	Bahamas	8-Jun-06	23.752	-76.105	Samhouri, Jameal	2.74		0.0%			0	5.25
2404	G-spot	Exuma Cays	Bahamas	9-Jun-06	23.831	-76.198	Samhouri, Jameal	3.65		0.0%			0	5.25
2405	Rainbow	Exuma Cays	Bahamas	9-Jun-06	23.797	-76.146	Samhouri, Jameal	7.77		0.0%			0	5.25
2406	Square Rock	Exuma Cays	Bahamas	10-Jun-06	23.710	-76.076	Samhouri, Jameal	3.12		0.0%			0	5.25
2407	Windsock	Exuma Cays	Bahamas	10-Jun-06	23.743	-76.076	Samhouri, Jameal	3.96		0.0%			0	5.25
1762	Site 1	west coast	Barbados	15-Sep-05	13.136	-59.638	Oxenford, Hazel	11.5		71.6%			5.1	11
1763	Site 2	west coast	Barbados	23-Sep-05	13.292	-59.663	Oxenford, Hazel	11.5		58.4%			6.6	11
1764	Site 3	west coast	Barbados	30-Sep-05	13.122	-59.649	Oxenford, Hazel	11.5		62.7%			7.85	11
1765	Site 4	west coast	Barbados	4-Oct-05	13.188	-59.642	Oxenford, Hazel	11.5		82.7%			8.5	11
1766	Site 5	southwest coast	Barbados	5-Oct-05	13.060	-59.557	Oxenford, Hazel	11.5		62.5%			8.5	11
1767	Site 6	southwest coast	Barbados	6-Oct-05	13.073	-59.603	Oxenford, Hazel	11.5		85.1%			8.5	11
1010	BAR1-1	Bellairs	Barbados	8-Jan-06	13.188	-59.642	Ginsburg, Robert	3.5		36.7%	8.6%	6.7%	0.65	11
1011	BAR1-2	N of Lone Star	Barbados	8-Jan-06	13.212	-59.645	Ginsburg, Robert	13.5		55.0%	4.5%	1.7%	0.65	11
1012	BAR2-1	Asta Sand Channel	Barbados	9-Jan-06	13.073	-59.605	Ginsburg, Robert	10		36.5%	8.7%	0.0%	1.2	11
1013	BAR2-2	Needham Point	Barbados	9-Jan-06	13.078	-59.615	Ginsburg, Robert	10		21.3%	0.3%	0.0%	1.2	11
1014	BAR2-3	Coconut Court Buoy	Barbados	9-Jan-06	13.073	-59.603	Ginsburg, Robert	6.5		26.7%	1.0%	0.0%	1.2	11
1015	BAR3-1	Atlantis Bank Reef	Barbados	10-Jan-06	13.122	-59.649	Ginsburg, Robert	20		65.9%	6.7%	0.0%	1.2	11
1016	BAR3-2	Pile Bay (Oil Cans)	Barbados	10-Jan-06	13.123	-59.638	Ginsburg, Robert	5.5		45.7%	3.4%	1.2%	1.2	11
1017	BAR3-3	Brighton Stadium	Barbados	10-Jan-06	13.116	-59.634	Ginsburg, Robert	3.5		18.1%	10.0%	1.2%	1.2	11
1018	BAR4-1	Chefette	Barbados	11-Jan-06	13.183	-59.647	Ginsburg, Robert	15		70.9%	4.4%	0.0%	1.2	11
1019	BAR4-2	Bachelors Hall	Barbados	11-Jan-06	13.200	-59.644	Ginsburg, Robert	3		31.4%	7.2%	0.0%	1.2	11
1701	BAR2-2	Needham Point	Barbados	26-May-06	13.078	-59.615	Lang, Judith	10		17.3%			0	11
1702	BAR2-1	Asta Sand Channel	Barbados	27-May-06	13.073	-59.605	Lang, Judith	9.6		28.7%			0	11
1703	BAR2-3	Coconut Court Buoy	Barbados	27-May-06	13.073	-59.603	Lang, Judith	5.6		12.0%			0	11
1704	BAR3-1	Atlantis Bank Reef	Barbados	27-May-06	13.122	-59.649	Lang, Judith	18.7		39.4%			0	11
1705	BAR1-2	N of Lone Star	Barbados	28-May-06	13.212	-59.645	Lang, Judith	10.9		28.0%			0	11
1706	BAR4-1	Chefette	Barbados	28-May-06	13.183	-59.647	Lang, Judith	14.5		39.3%			0	11
1707	BAR4-2	Bachelors Hall	Barbados	28-May-06	13.200	-59.644	Lang, Judith	4.1		7.5%			0	11

Observation Number	Reef or Site Name	Location	Country	Date (DD-MMM-YY)	Latitude (decimal degrees)	Longitude (decimal degrees)	Primary Contributor	Depth (m)	Cover bleached (%)	Colonies bleached (%)	Cover dead (%)	Colonies dead (%)	Observed DHW (°C-weeks)	Maximum DHW (°C-weeks)
1708	BAR1-1	Bellairs	Barbados	29-May-06	13.188	-59.642	Lang, Judith	4.6		12.3%			0	11
2059	Lime Caye Wall	Sapodilla Cayes	Belize	18-Aug-05	16.095	-88.271	Reef Check	10	5.0%	18.0%			1.7	3.8
2060	Ragged Caye	Sapodilla Cayes	Belize	18-Aug-05	16.094	-88.292	Reef Check	10	6.3%	26.3%			1.7	3.8
2065	Basil Jones	Ambergris Caye	Belize	13-Sep-05	18.086	-87.866	Reef Check	1.5	5.0%	11.3%			0.5	0.5
2066	Basil Jones	Ambergris Caye	Belize	13-Sep-05	18.086	-87.866	Reef Check	8.75	45.0%	80.0%			0.5	0.5
2067	Mexico Rocks	Ambergris Caye	Belize	14-Sep-05	17.994	-87.901	Reef Check	12.75	62.5%	65.0%			0.5	0.5
2068	Coral Gardens	Ambergris Caye	Belize	19-Sep-05	17.826	-87.985	Reef Check	11.3	50.0%	50.0%			0.5	0.5
2069	Coral Gardens	Ambergris Caye	Belize	19-Sep-05	17.824	-87.994	Reef Check	2.6	40.0%	42.5%			0.5	0.5
2070	Caye Caulker	Ambergris Caye	Belize	20-Sep-05	17.784	-88.001	Reef Check	3.2	80.0%	90.0%			0.5	0.5
2071	Caye Caulker	Ambergris Caye	Belize	20-Sep-05	17.784	-87.992	Reef Check	11.5	26.3%	35.0%			0.5	0.5
2072	Caye Chapel	Ambergris Caye	Belize	20-Sep-05	17.684	-88.011	Reef Check	13.75	45.0%	50.0%			0	0
2073	Caye Chapel	Ambergris Caye	Belize	20-Sep-05	17.704	-88.016	Reef Check	4.4	80.0%	30.0%			0	0
2074	Gallows Point	Ambergris Caye	Belize	21-Sep-05	17.507	-88.047	Reef Check	2.65	6.8%	20.0%			0	0
2075	Gallows Point	Ambergris Caye	Belize	21-Sep-05	17.507	-88.047	Reef Check	5.8	45.0%	40.0%			0	0
2076	Goff's Caye	Ambergris Caye	Belize	21-Sep-05	17.340	-88.020	Reef Check	10.75	10.0%	50.0%			0	0
2077	Goff's Caye	Ambergris Caye	Belize	21-Sep-05	17.343	-88.032	Reef Check	2.15	3.0%	7.5%			0	0
2078	Rendevouz Caye		Belize	22-Sep-05	17.248	-88.056	Reef Check	10	8.8%	20.0%			0	0
2079	Rendevouz Caye		Belize	22-Sep-05	17.248	-88.056	Reef Check	5	6.3%	27.5%			0	0
2678	(5) Gladden Spit	Gladden Spit Seascape	Belize	4-Oct-05	16.533	-87.979	Walsh, Sheila	9	10.0%	28.2%	2.0%	1.4%	3.8	3.8
2679	(5) Gladden Spit	Gladden Spit Seascape	Belize	4-Oct-05	16.533	-87.979	Walsh, Sheila	9	25.5%		0.0%		3.8	3.8
2680	(5) Gladden Spit	Gladden Spit Seascape	Belize	4-Oct-05	16.533	-87.979	Walsh, Sheila	9	3.0%		5.0%		3.8	3.8
2681	(5) Gladden Spit	Gladden Spit Seascape	Belize	4-Oct-05	16.533	-87.979	Walsh, Sheila	9	0.0%		0.0%		3.8	3.8
2682	(5) Gladden Spit	Gladden Spit Seascape	Belize	4-Oct-05	16.533	-87.979	Walsh, Sheila	9	4.4%		0.0%		3.8	3.8
2683	(5) Gladden Spit	Gladden Spit Seascape	Belize	4-Oct-05	16.533	-87.979	Walsh, Sheila	9	77.0%		0.0%		3.8	3.8
2684	(5) Gladden Spit	Gladden Spit Seascape	Belize	4-Oct-05	16.533	-87.979	Walsh, Sheila	9	0.0%		0.0%		3.8	3.8
2685	(5) Gladden Spit	Gladden Spit Seascape	Belize	4-Oct-05	16.533	-87.979	Walsh, Sheila	9	1.5%		0.0%		3.8	3.8
2686	(5) Gladden Spit	Gladden Spit Seascape	Belize	4-Oct-05	16.533	-87.979	Walsh, Sheila	9	18.8%		0.0%		3.8	3.8
2687	(5) Gladden Spit	Gladden Spit Seascape	Belize	4-Oct-05	16.533	-87.979	Walsh, Sheila	9	51.8%		0.0%		3.8	3.8
2688	(5) Gladden Spit	Gladden Spit Seascape	Belize	4-Oct-05	16.533	-87.979	Walsh, Sheila	9	0.0%		0.0%		3.8	3.8
2689	(6) Pompion Caye	Gladden Spit Seascape	Belize	5-Oct-05	16.393	-88.070	Walsh, Sheila	11.7	15.4%	9.8%	0.0%	0.0%	3.8	3.8
2690	(6) Pompion Caye	Gladden Spit Seascape	Belize	5-Oct-05	16.393	-88.070	Walsh, Sheila	11.7	0.0%		0.0%		3.8	3.8
2691	(6) Pompion Caye	Gladden Spit Seascape	Belize	5-Oct-05	16.393	-88.070	Walsh, Sheila	11.7	0.0%		0.0%		3.8	3.8
2692	(6) Pompion Caye	Gladden Spit Seascape	Belize	5-Oct-05	16.393	-88.070	Walsh, Sheila	11.7	20.0%		0.0%		3.8	3.8
2693	(6) Pompion Caye	Gladden Spit Seascape	Belize	5-Oct-05	16.393	-88.070	Walsh, Sheila	11.7	0.0%		0.0%		3.8	3.8
2694	(6) Pompion Caye	Gladden Spit Seascape	Belize	5-Oct-05	16.393	-88.070	Walsh, Sheila	11.7	0.0%		0.0%		3.8	3.8
2695	(6) Pompion Caye	Gladden Spit Seascape	Belize	5-Oct-05	16.393	-88.070	Walsh, Sheila	11.7	16.0%		0.0%		3.8	3.8
2696	(6) Pompion Caye	Gladden Spit Seascape	Belize	5-Oct-05	16.393	-88.070	Walsh, Sheila	11.7	0.0%		0.0%		3.8	3.8
2697	(7) Ranguana Caye Canyons	Sapodilla Cayes Region	Belize	5-Oct-05	16.330	-88.126	Walsh, Sheila	12.3	3.9%	22.5%	0.0%	2.0%	3.8	3.8
2698	(7) Ranguana Caye Canyons	Sapodilla Cayes Region	Belize	5-Oct-05	16.330	-88.126	Walsh, Sheila	12.3	0.0%		0.0%		3.8	3.8
2699	(7) Ranguana Caye Canyons	Sapodilla Cayes Region	Belize	5-Oct-05	16.330	-88.126	Walsh, Sheila	12.3	0.0%		1.4%		3.8	3.8
2700	(7) Ranguana Caye Canyons	Sapodilla Cayes Region	Belize	5-Oct-05	16.330	-88.126	Walsh, Sheila	12.3	26.0%		0.0%		3.8	3.8
2701	(7) Ranguana Caye Canyons	Sapodilla Cayes Region	Belize	5-Oct-05	16.330	-88.126	Walsh, Sheila	12.3	0.0%		0.0%		3.8	3.8
2702	(7) Ranguana Caye Canyons	Sapodilla Cayes Region	Belize	5-Oct-05	16.330	-88.126	Walsh, Sheila	12.3	0.0%		0.0%		3.8	3.8
2703	(7) Ranguana Caye Canyons	Sapodilla Cayes Region	Belize	5-Oct-05	16.330	-88.126	Walsh, Sheila	12.3	26.7%		0.0%		3.8	3.8
2704	(7) Ranguana Caye Canyons	Sapodilla Cayes Region	Belize	5-Oct-05	16.330	-88.126	Walsh, Sheila	12.3	0.8%		0.0%		3.8	3.8
2705	(7) Ranguana Caye Canyons	Sapodilla Cayes Region	Belize	5-Oct-05	16.330	-88.126	Walsh, Sheila	12.3	2.5%		0.0%		3.8	3.8
2706	(7) Ranguana Caye Canyons	Sapodilla Cayes Region	Belize	5-Oct-05	16.330	-88.126	Walsh, Sheila	12.3	27.2%		1.4%		3.8	3.8
2707	(8) Ranguana Caye South Canyo	Sapodilla Cayes Region	Belize	5-Oct-05	16.313	-88.137	Walsh, Sheila	11.4	14.2%	38.6%	0.0%	0.0%	3.8	3.8
2708	(8) Ranguana Caye South Canyo	Sapodilla Cayes Region	Belize	5-Oct-05	16.313	-88.137	Walsh, Sheila	11.4	2.0%		0.0%		3.8	3.8
2709	(8) Ranguana Caye South Canyo	Sapodilla Cayes Region	Belize	5-Oct-05	16.313	-88.137	Walsh, Sheila	11.4	10.7%		0.0%		3.8	3.8
2710	(8) Ranguana Caye South Canyo	Sapodilla Cayes Region	Belize	5-Oct-05	16.313	-88.137	Walsh, Sheila	11.4	6.9%		0.0%		3.8	3.8
2711	(8) Ranguana Caye South Canyo	Sapodilla Cayes Region	Belize	5-Oct-05	16.313	-88.137	Walsh, Sheila	11.4	15.9%		0.0%		3.8	3.8
2712	(8) Ranguana Caye South Canyo	Sapodilla Cayes Region	Belize	5-Oct-05	16.313	-88.137	Walsh, Sheila	11.4	0.0%		0.0%		3.8	3.8
2713	(8) Ranguana Caye South Canyo	Sapodilla Cayes Region	Belize	5-Oct-05	16.313	-88.137	Walsh, Sheila	11.4	16.7%		0.0%		3.8	3.8
2714	(11) Tom Owen's Caye	Sapodilla Cayes	Belize	6-Oct-05	16.218	-88.191	Walsh, Sheila	13.35	12.0%	23.5%	0.0%	0.0%	3.8	3.8
2715	(11) Tom Owen's Caye	Sapodilla Cayes	Belize	6-Oct-05	16.218	-88.191	Walsh, Sheila	13.35	0.0%		0.0%		3.8	3.8
2716	(11) Tom Owen's Caye	Sapodilla Cayes	Belize	6-Oct-05	16.218	-88.191	Walsh, Sheila	13.35	6.0%		0.0%		3.8	3.8
2717	(11) Tom Owen's Caye	Sapodilla Cayes	Belize	6-Oct-05	16.218	-88.191	Walsh, Sheila	13.35	0.0%		0.0%		3.8	3.8

Observation Number	Reef or Site Name	Location	Country	Date (DD-MMM-YY)	Latitude (decimal degrees)	Longitude (decimal degrees)	Primary Contributor	Depth (m)	Cover bleached (%)	Colonies bleached (%)	Cover dead (%)	Colonies dead (%)	Observed DHW (°C-weeks)	Maximum DHW (°C-weeks)
2718	(11) Tom Owen's Caye	Sapodilla Cayes	Belize	6-Oct-05	16.218	-88.191	Walsh, Sheila	13.35	0.0%		0.0%		3.8	3.8
2719	(11) Tom Owen's Caye	Sapodilla Cayes	Belize	6-Oct-05	16.218	-88.191	Walsh, Sheila	13.35	25.5%		0.0%		3.8	3.8
2720	(11) Tom Owen's Caye	Sapodilla Cayes	Belize	6-Oct-05	16.218	-88.191	Walsh, Sheila	13.35	19.0%		0.0%		3.8	3.8
2721	(11) Tom Owen's Caye	Sapodilla Cayes	Belize	6-Oct-05	16.218	-88.191	Walsh, Sheila	13.35	12.0%		0.0%		3.8	3.8
2722	(11) Tom Owen's Caye	Sapodilla Cayes	Belize	6-Oct-05	16.218	-88.191	Walsh, Sheila	13.35	0.0%		0.0%		3.8	3.8
2723	(11) Tom Owen's Caye	Sapodilla Cayes	Belize	6-Oct-05	16.218	-88.191	Walsh, Sheila	13.35	10.8%		0.0%		3.8	3.8
2724	(11) Tom Owen's Caye	Sapodilla Cayes	Belize	6-Oct-05	16.218	-88.191	Walsh, Sheila	13.35	0.0%		0.0%		3.8	3.8
2725	(11) Tom Owen's Caye	Sapodilla Cayes	Belize	6-Oct-05	16.218	-88.191	Walsh, Sheila	13.35	28.6%		0.0%		3.8	3.8
2726	(12) North East Sapodilla Caye	Sapodilla Cayes	Belize	6-Oct-05	16.154	-88.237	Walsh, Sheila	12	0.0%	13.0%	0.0%	0.7%	3.8	3.8
2727	(12) North East Sapodilla Caye	Sapodilla Cayes	Belize	6-Oct-05	16.154	-88.237	Walsh, Sheila	12	0.0%		0.0%		3.8	3.8
2728	(12) North East Sapodilla Caye	Sapodilla Cayes	Belize	6-Oct-05	16.154	-88.237	Walsh, Sheila	12	0.7%		0.0%		3.8	3.8
2729	(12) North East Sapodilla Caye	Sapodilla Cayes	Belize	6-Oct-05	16.154	-88.237	Walsh, Sheila	12	0.0%		0.0%		3.8	3.8
2730	(12) North East Sapodilla Caye	Sapodilla Cayes	Belize	6-Oct-05	16.154	-88.237	Walsh, Sheila	12	8.0%		0.0%		3.8	3.8
2731	(12) North East Sapodilla Caye	Sapodilla Cayes	Belize	6-Oct-05	16.154	-88.237	Walsh, Sheila	12	0.0%		3.3%		3.8	3.8
2732	(12) North East Sapodilla Caye	Sapodilla Cayes	Belize	6-Oct-05	16.154	-88.237	Walsh, Sheila	12	25.0%		0.0%		3.8	3.8
2733	(12) North East Sapodilla Caye	Sapodilla Cayes	Belize	6-Oct-05	16.154	-88.237	Walsh, Sheila	12	0.0%		0.0%		3.8	3.8
2734	(12) North East Sapodilla Caye	Sapodilla Cayes	Belize	6-Oct-05	16.154	-88.237	Walsh, Sheila	12	17.1%		0.0%		3.8	3.8
2735	(12) North East Sapodilla Caye	Sapodilla Cayes	Belize	6-Oct-05	16.154	-88.237	Walsh, Sheila	12	0.0%		0.0%		3.8	3.8
2736	(12) North East Sapodilla Caye	Sapodilla Cayes	Belize	6-Oct-05	16.154	-88.237	Walsh, Sheila	12	0.0%		0.0%		3.8	3.8
2737	(12) North East Sapodilla Caye	Sapodilla Cayes	Belize	6-Oct-05	16.154	-88.237	Walsh, Sheila	12	8.6%		0.0%		3.8	3.8
2738	(12) North East Sapodilla Caye	Sapodilla Cayes	Belize	6-Oct-05	16.154	-88.237	Walsh, Sheila	12	1.0%		0.0%		3.8	3.8
2739	(12) North East Sapodilla Caye	Sapodilla Cayes	Belize	6-Oct-05	16.154	-88.237	Walsh, Sheila	12	30.0%		0.0%		3.8	3.8
2740	(12) North East Sapodilla Caye	Sapodilla Cayes	Belize	6-Oct-05	16.154	-88.237	Walsh, Sheila	12	4.2%		0.0%		3.8	3.8
2741	(10) North Spot	Sapodilla Cayes	Belize	7-Oct-05	16.176	-88.225	Walsh, Sheila	12	13.8%	45.8%	0.0%	3.5%	3.2	3.8
2742	(10) North Spot	Sapodilla Cayes	Belize	7-Oct-05	16.176	-88.225	Walsh, Sheila	12	13.3%		0.0%		3.2	3.8
2743	(10) North Spot	Sapodilla Cayes	Belize	7-Oct-05	16.176	-88.225	Walsh, Sheila	12	27.5%		0.0%		3.2	3.8
2744	(10) North Spot	Sapodilla Cayes	Belize	7-Oct-05	16.176	-88.225	Walsh, Sheila	12	11.6%		0.0%		3.2	3.8
2745	(10) North Spot	Sapodilla Cayes	Belize	7-Oct-05	16.176	-88.225	Walsh, Sheila	12	18.0%		0.0%		3.2	3.8
2746	(10) North Spot	Sapodilla Cayes	Belize	7-Oct-05	16.176	-88.225	Walsh, Sheila	12	7.9%		2.5%		3.2	3.8
2747	(10) North Spot	Sapodilla Cayes	Belize	7-Oct-05	16.176	-88.225	Walsh, Sheila	12	20.0%		0.0%		3.2	3.8
2748	(10) North Spot	Sapodilla Cayes	Belize	7-Oct-05	16.176	-88.225	Walsh, Sheila	12	0.3%		0.8%		3.2	3.8
2749	(10) North Spot	Sapodilla Cayes	Belize	7-Oct-05	16.176	-88.225	Walsh, Sheila	12	26.6%		1.4%		3.2	3.8
2750	(10) North Spot	Sapodilla Cayes	Belize	7-Oct-05	16.176	-88.225	Walsh, Sheila	12	2.5%		0.0%		3.2	3.8
2751	(10) North Spot	Sapodilla Cayes	Belize	7-Oct-05	16.176	-88.225	Walsh, Sheila	12	10.3%		0.0%		3.2	3.8
2752	(10) North Spot	Sapodilla Cayes	Belize	7-Oct-05	16.176	-88.225	Walsh, Sheila	12	4.9%		0.0%		3.2	3.8
2753	(10) North Spot	Sapodilla Cayes	Belize	7-Oct-05	16.176	-88.225	Walsh, Sheila	12	46.7%		0.0%		3.2	3.8
2754	(9) No Name Site	Sapodilla Cayes Region	Belize	7-Oct-05	16.251	-88.176	Walsh, Sheila	10.2	5.8%	13.9%	0.0%	1.9%	3.2	3.8
2755	(9) No Name Site	Sapodilla Cayes Region	Belize	7-Oct-05	16.251	-88.176	Walsh, Sheila	10.2	25.0%		0.0%		3.2	3.8
2756	(9) No Name Site	Sapodilla Cayes Region	Belize	7-Oct-05	16.251	-88.176	Walsh, Sheila	10.2	10.4%		0.4%		3.2	3.8
2757	(9) No Name Site	Sapodilla Cayes Region	Belize	7-Oct-05	16.251	-88.176	Walsh, Sheila	10.2	0.0%		0.0%		3.2	3.8
2758	(9) No Name Site	Sapodilla Cayes Region	Belize	7-Oct-05	16.251	-88.176	Walsh, Sheila	10.2	0.0%		0.0%		3.2	3.8
2759	(9) No Name Site	Sapodilla Cayes Region	Belize	7-Oct-05	16.251	-88.176	Walsh, Sheila	10.2	0.0%		0.0%		3.2	3.8
2760	(9) No Name Site	Sapodilla Cayes Region	Belize	7-Oct-05	16.251	-88.176	Walsh, Sheila	10.2	0.0%		0.0%		3.2	3.8
2761	(9) No Name Site	Sapodilla Cayes Region	Belize	7-Oct-05	16.251	-88.176	Walsh, Sheila	10.2	0.0%		0.0%		3.2	3.8
2762	(9) No Name Site	Sapodilla Cayes Region	Belize	7-Oct-05	16.251	-88.176	Walsh, Sheila	10.2	21.6%		3.2%		3.2	3.8
2763	(9) No Name Site	Sapodilla Cayes Region	Belize	7-Oct-05	16.251	-88.176	Walsh, Sheila	10.2	0.0%		0.0%		3.2	3.8
2764	(9) No Name Site	Sapodilla Cayes Region	Belize	7-Oct-05	16.251	-88.176	Walsh, Sheila	10.2	0.0%		0.0%		3.2	3.8
2765	(9) No Name Site	Sapodilla Cayes Region	Belize	7-Oct-05	16.251	-88.176	Walsh, Sheila	10.2	0.0%		0.0%		3.2	3.8
2766	(9) No Name Site	Sapodilla Cayes Region	Belize	7-Oct-05	16.251	-88.176	Walsh, Sheila	10.2	3.6%		1.4%		3.2	3.8
2767	(9) No Name Site	Sapodilla Cayes Region	Belize	7-Oct-05	16.251	-88.176	Walsh, Sheila	10.2	1.4%		0.0%		3.2	3.8
2768	(9) No Name Site	Sapodilla Cayes Region	Belize	7-Oct-05	16.251	-88.176	Walsh, Sheila	10.2	0.0%		0.0%		3.2	3.8
2769	(9) No Name Site	Sapodilla Cayes Region	Belize	7-Oct-05	16.251	-88.176	Walsh, Sheila	10.2	12.5%		2.9%		3.2	3.8
2770	(2) Turneffe Atoll	Turneffe Atoll	Belize	9-Oct-05	17.538	-87.744	Walsh, Sheila	11.85	8.0%	18.3%	0.0%	1.5%	0	0
2771	(2) Turneffe Atoll	Turneffe Atoll	Belize	9-Oct-05	17.538	-87.744	Walsh, Sheila	11.85	17.7%		0.0%		0	0
2772	(2) Turneffe Atoll	Turneffe Atoll	Belize	9-Oct-05	17.538	-87.744	Walsh, Sheila	11.85	2.4%		0.0%		0	0
2773	(2) Turneffe Atoll	Turneffe Atoll	Belize	9-Oct-05	17.538	-87.744	Walsh, Sheila	11.85	7.7%		0.0%		0	0
2774	(2) Turneffe Atoll	Turneffe Atoll	Belize	9-Oct-05	17.538	-87.744	Walsh, Sheila	11.85	65.0%		1.9%		0	0
2775	(2) Turneffe Atoll	Turneffe Atoll	Belize	9-Oct-05	17.538	-87.744	Walsh, Sheila	11.85	31.7%		5.0%		0	0

Observation Number	Reef or Site Name	Location	Country	Date (DD-MMM-YY)	Latitude (decimal degrees)	Longitude (decimal degrees)	Primary Contributor	Depth (m)	Cover bleached (%)	Colonies bleached (%)	Cover dead (%)	Colonies dead (%)	Observed DHW (°C-weeks)	Maximum DHW (°C-weeks)
2776	(2) Turneffe Atoll	Turneffe Atoll	Belize	9-Oct-05	17.538	-87.744	Walsh, Sheila	11.85	14.3%		0.0%		0	0
2777	(2) Turneffe Atoll	Turneffe Atoll	Belize	9-Oct-05	17.538	-87.744	Walsh, Sheila	11.85	0.0%		0.0%		0	0
2778	(2) Turneffe Atoll	Turneffe Atoll	Belize	9-Oct-05	17.538	-87.744	Walsh, Sheila	11.85	0.0%		0.0%		0	0
2779	(2) Turneffe Atoll	Turneffe Atoll	Belize	9-Oct-05	17.538	-87.744	Walsh, Sheila	11.85	62.5%		0.0%		0	0
2780	(2) Turneffe Atoll	Turneffe Atoll	Belize	9-Oct-05	17.538	-87.744	Walsh, Sheila	11.85	0.0%		0.0%		0	0
2781	(3) Turneffe Atoll	Turneffe Atoll	Belize	9-Oct-05	17.500	-87.758	Walsh, Sheila	11.25	56.9%	18.0%	0.0%	1.4%	0	0
2782	(3) Turneffe Atoll	Turneffe Atoll	Belize	9-Oct-05	17.500	-87.758	Walsh, Sheila	11.25	4.0%		0.3%		0	0
2783	(3) Turneffe Atoll	Turneffe Atoll	Belize	9-Oct-05	17.500	-87.758	Walsh, Sheila	11.25	4.3%		0.0%		0	0
2784	(3) Turneffe Atoll	Turneffe Atoll	Belize	9-Oct-05	17.500	-87.758	Walsh, Sheila	11.25	13.8%		0.0%		0	0
2785	(3) Turneffe Atoll	Turneffe Atoll	Belize	9-Oct-05	17.500	-87.758	Walsh, Sheila	11.25	0.0%		0.0%		0	0
2786	(3) Turneffe Atoll	Turneffe Atoll	Belize	9-Oct-05	17.500	-87.758	Walsh, Sheila	11.25	60.0%		0.0%		0	0
2787	(3) Turneffe Atoll	Turneffe Atoll	Belize	9-Oct-05	17.500	-87.758	Walsh, Sheila	11.25	8.9%		0.0%		0	0
2788	(3) Turneffe Atoll	Turneffe Atoll	Belize	9-Oct-05	17.500	-87.758	Walsh, Sheila	11.25	0.0%		0.0%		0	0
2789	(3) Turneffe Atoll	Turneffe Atoll	Belize	9-Oct-05	17.500	-87.758	Walsh, Sheila	11.25	0.0%		0.0%		0	0
2790	(3) Turneffe Atoll	Turneffe Atoll	Belize	9-Oct-05	17.500	-87.758	Walsh, Sheila	11.25	0.0%		0.0%		0	0
2791	(3) Turneffe Atoll	Turneffe Atoll	Belize	9-Oct-05	17.500	-87.758	Walsh, Sheila	11.25	49.2%		0.1%		0	0
2792	(1) Turneffe Atoll	Turneffe Atoll	Belize	10-Oct-05	17.563	-87.475	Walsh, Sheila	11	11.8%	29.9%	3.6%	2.1%	0	0
2793	(1) Turneffe Atoll	Turneffe Atoll	Belize	10-Oct-05	17.563	-87.475	Walsh, Sheila	11	16.5%		0.0%		0	0
2794	(1) Turneffe Atoll	Turneffe Atoll	Belize	10-Oct-05	17.563	-87.475	Walsh, Sheila	11	19.6%		0.4%		0	0
2795	(1) Turneffe Atoll	Turneffe Atoll	Belize	10-Oct-05	17.563	-87.475	Walsh, Sheila	11	0.0%		0.0%		0	0
2796	(1) Turneffe Atoll	Turneffe Atoll	Belize	10-Oct-05	17.563	-87.475	Walsh, Sheila	11	17.5%		0.0%		0	0
2797	(1) Turneffe Atoll	Turneffe Atoll	Belize	10-Oct-05	17.563	-87.475	Walsh, Sheila	11	55.0%		4.2%		0	0
2798	(1) Turneffe Atoll	Turneffe Atoll	Belize	10-Oct-05	17.563	-87.475	Walsh, Sheila	11	0.4%		0.0%		0	0
2799	(1) Turneffe Atoll	Turneffe Atoll	Belize	10-Oct-05	17.563	-87.475	Walsh, Sheila	11	0.0%		0.0%		0	0
2800	(1) Turneffe Atoll	Turneffe Atoll	Belize	10-Oct-05	17.563	-87.475	Walsh, Sheila	11	0.0%		0.0%		0	0
2801	(1) Turneffe Atoll	Turneffe Atoll	Belize	10-Oct-05	17.563	-87.475	Walsh, Sheila	11	0.0%		0.0%		0	0
2802	(1) Turneffe Atoll	Turneffe Atoll	Belize	10-Oct-05	17.563	-87.475	Walsh, Sheila	11	54.0%		0.0%		0	0
2803	(1) Turneffe Atoll	Turneffe Atoll	Belize	10-Oct-05	17.563	-87.475	Walsh, Sheila	11	0.0%		0.0%		0	0
2804	(4) Turneffe Atoll	Turneffe Atoll	Belize	10-Oct-05	17.484	-87.480	Walsh, Sheila	11.55	40.0%	21.4%	0.0%	1.2%	0	0
2805	(4) Turneffe Atoll	Turneffe Atoll	Belize	10-Oct-05	17.484	-87.480	Walsh, Sheila	11.55	21.3%		1.0%		0	0
2806	(4) Turneffe Atoll	Turneffe Atoll	Belize	10-Oct-05	17.484	-87.480	Walsh, Sheila	11.55	18.9%		0.0%		0	0
2807	(4) Turneffe Atoll	Turneffe Atoll	Belize	10-Oct-05	17.484	-87.480	Walsh, Sheila	11.55	5.0%		0.0%		0	0
2808	(4) Turneffe Atoll	Turneffe Atoll	Belize	10-Oct-05	17.484	-87.480	Walsh, Sheila	11.55	6.7%		0.0%		0	0
2809	(4) Turneffe Atoll	Turneffe Atoll	Belize	10-Oct-05	17.484	-87.480	Walsh, Sheila	11.55	0.0%		0.0%		0	0
2810	(4) Turneffe Atoll	Turneffe Atoll	Belize	10-Oct-05	17.484	-87.480	Walsh, Sheila	11.55	0.0%		0.0%		0	0
2811	(4) Turneffe Atoll	Turneffe Atoll	Belize	10-Oct-05	17.484	-87.480	Walsh, Sheila	11.55	5.6%		0.0%		0	0
2812	(4) Turneffe Atoll	Turneffe Atoll	Belize	10-Oct-05	17.484	-87.480	Walsh, Sheila	11.55	0.0%		0.0%		0	0
2813	(4) Turneffe Atoll	Turneffe Atoll	Belize	10-Oct-05	17.484	-87.480	Walsh, Sheila	11.55	70.0%		0.3%		0	0
1089	Long Caye site 1	Glovers Reef	Belize	14-Oct-05	16.749	-87.786	Husain, Ellen	7	0.0%	36.4%			2.1	3.8
1090	Long Caye site 1	Glovers Reef	Belize	14-Oct-05	16.749	-87.786	Husain, Ellen	7	0.0%				2.1	3.8
1091	Long Caye site 1	Glovers Reef	Belize	14-Oct-05	16.749	-87.786	Husain, Ellen	7	50.0%				2.1	3.8
1092	Long Caye site 1	Glovers Reef	Belize	14-Oct-05	16.749	-87.786	Husain, Ellen	7	100.0%				2.1	3.8
1093	Long Caye site 1	Glovers Reef	Belize	14-Oct-05	16.749	-87.786	Husain, Ellen	7	66.7%				2.1	3.8
1094	Long Caye site 1	Glovers Reef	Belize	14-Oct-05	16.749	-87.786	Husain, Ellen	7	75.0%				2.1	3.8
1095	Long Caye site 1	Glovers Reef	Belize	14-Oct-05	16.749	-87.786	Husain, Ellen	7	0.0%				2.1	3.8
1096	Long Caye site 1	Glovers Reef	Belize	14-Oct-05	16.749	-87.786	Husain, Ellen	7	14.3%				2.1	3.8
1097	Long Caye site 1	Glovers Reef	Belize	14-Oct-05	16.749	-87.786	Husain, Ellen	7	37.5%				2.1	3.8
1098	Long Caye site 1	Glovers Reef	Belize	14-Oct-05	16.749	-87.786	Husain, Ellen	7	0.0%				2.1	3.8
1099	Long Caye site 1	Glovers Reef	Belize	14-Oct-05	16.749	-87.786	Husain, Ellen	7	0.0%				2.1	3.8
1100	Long Caye site 1	Glovers Reef	Belize	14-Oct-05	16.749	-87.786	Husain, Ellen	7	15.4%				2.1	3.8
1101	Long Caye site 1	Glovers Reef	Belize	14-Oct-05	16.749	-87.786	Husain, Ellen	7	17.2%				2.1	3.8
1102	Long Caye site 1	Glovers Reef	Belize	14-Oct-05	16.749	-87.786	Husain, Ellen	7	16.7%				2.1	3.8
1103	Long Caye site 1	Glovers Reef	Belize	14-Oct-05	16.749	-87.786	Husain, Ellen	7	23.7%				2.1	3.8
1104	Long Caye site 3	Glovers Reef	Belize	14-Oct-05	16.749	-87.786	Husain, Ellen	9	0.0%	48.5%			2.1	3.8
1105	Long Caye site 3	Glovers Reef	Belize	14-Oct-05	16.749	-87.786	Husain, Ellen	9	2.0%				2.1	3.8
1106	Long Caye site 3	Glovers Reef	Belize	14-Oct-05	16.749	-87.786	Husain, Ellen	9	3.0%				2.1	3.8
1107	Long Caye site 3	Glovers Reef	Belize	14-Oct-05	16.749	-87.786	Husain, Ellen	9	1.0%				2.1	3.8
1108	Long Caye site 3	Glovers Reef	Belize	14-Oct-05	16.749	-87.786	Husain, Ellen	9	2.0%				2.1	3.8

Observation Number	Reef or Site Name	Location	Country	Date (DD-MMM-YY)	Latitude (decimal degrees)	Longitude (decimal degrees)	Primary Contributor	Depth (m)	Cover bleached (%)	Colonies bleached (%)	Cover dead (%)	Colonies dead (%)	Observed DHW (°C-weeks)	Maximum DHW (°C-weeks)
1109	Long Caye site 3	Glovers Reef	Belize	14-Oct-05	16.749	-87.786	Husain, Ellen	9	15.0%				2.1	3.8
1110	Long Caye site 3	Glovers Reef	Belize	14-Oct-05	16.749	-87.786	Husain, Ellen	9	0.0%				2.1	3.8
1111	Long Caye site 3	Glovers Reef	Belize	14-Oct-05	16.749	-87.786	Husain, Ellen	9	1.0%				2.1	3.8
1112	Long Caye site 3	Glovers Reef	Belize	14-Oct-05	16.749	-87.786	Husain, Ellen	9	0.0%				2.1	3.8
1113	Long Caye site 3	Glovers Reef	Belize	14-Oct-05	16.749	-87.786	Husain, Ellen	9	3.0%				2.1	3.8
1114	Long Caye site 3	Glovers Reef	Belize	14-Oct-05	16.749	-87.786	Husain, Ellen	9	9.0%				2.1	3.8
1115	Long Caye site 3	Glovers Reef	Belize	14-Oct-05	16.749	-87.786	Husain, Ellen	9	8.0%				2.1	3.8
1116	Long Caye site 3	Glovers Reef	Belize	14-Oct-05	16.749	-87.786	Husain, Ellen	9	3.0%				2.1	3.8
1117	Long Caye site 3	Glovers Reef	Belize	14-Oct-05	16.749	-87.786	Husain, Ellen	9	1.0%				2.1	3.8
1118	Long Caye site 3	Glovers Reef	Belize	14-Oct-05	16.749	-87.786	Husain, Ellen	9	0.0%				2.1	3.8
1119	Long Caye site 3	Glovers Reef	Belize	14-Oct-05	16.749	-87.786	Husain, Ellen	9	4.0%				2.1	3.8
1120	Long Caye site 3	Glovers Reef	Belize	14-Oct-05	16.749	-87.786	Husain, Ellen	9	10.0%				2.1	3.8
1121	Long Caye site 4	Glovers Reef	Belize	28-Oct-05	16.749	-87.786	Husain, Ellen	9	1.0%	31.1%			2.1	3.8
1122	Long Caye site 4	Glovers Reef	Belize	28-Oct-05	16.749	-87.786	Husain, Ellen	9	1.0%				2.1	3.8
1123	Long Caye site 4	Glovers Reef	Belize	28-Oct-05	16.749	-87.786	Husain, Ellen	9	1.0%				2.1	3.8
1124	Long Caye site 4	Glovers Reef	Belize	28-Oct-05	16.749	-87.786	Husain, Ellen	9	0.0%				2.1	3.8
1125	Long Caye site 4	Glovers Reef	Belize	28-Oct-05	16.749	-87.786	Husain, Ellen	9	3.0%				2.1	3.8
1126	Long Caye site 4	Glovers Reef	Belize	28-Oct-05	16.749	-87.786	Husain, Ellen	9	1.0%				2.1	3.8
1127	Long Caye site 4	Glovers Reef	Belize	28-Oct-05	16.749	-87.786	Husain, Ellen	9	3.0%				2.1	3.8
1128	Long Caye site 4	Glovers Reef	Belize	28-Oct-05	16.749	-87.786	Husain, Ellen	9	0.0%				2.1	3.8
1129	Long Caye site 4	Glovers Reef	Belize	28-Oct-05	16.749	-87.786	Husain, Ellen	9	0.0%				2.1	3.8
1130	Long Caye site 4	Glovers Reef	Belize	28-Oct-05	16.749	-87.786	Husain, Ellen	9	1.0%				2.1	3.8
1131	Long Caye site 4	Glovers Reef	Belize	28-Oct-05	16.749	-87.786	Husain, Ellen	9	2.0%				2.1	3.8
1132	Long Caye site 4	Glovers Reef	Belize	28-Oct-05	16.749	-87.786	Husain, Ellen	9	0.0%				2.1	3.8
1133	Long Caye site 4	Glovers Reef	Belize	28-Oct-05	16.749	-87.786	Husain, Ellen	9	2.0%				2.1	3.8
1134	Long Caye site 4	Glovers Reef	Belize	28-Oct-05	16.749	-87.786	Husain, Ellen	9	1.0%				2.1	3.8
1135	Long Caye site 4	Glovers Reef	Belize	28-Oct-05	16.749	-87.786	Husain, Ellen	9	6.0%				2.1	3.8
1136	Long Caye site 4	Glovers Reef	Belize	28-Oct-05	16.749	-87.786	Husain, Ellen	9	1.0%				2.1	3.8
1137	Long Caye site 4	Glovers Reef	Belize	28-Oct-05	16.749	-87.786	Husain, Ellen	9	0.0%				2.1	3.8
1138	Middle Caye site 2	Glovers Reef	Belize	28-Oct-05	16.749	-87.786	Husain, Ellen	9	0.0%	20.0%			2.1	3.8
1139	Middle Caye site 2	Glovers Reef	Belize	28-Oct-05	16.749	-87.786	Husain, Ellen	9	0.0%				2.1	3.8
1140	Middle Caye site 2	Glovers Reef	Belize	28-Oct-05	16.749	-87.786	Husain, Ellen	9	0.0%				2.1	3.8
1141	Middle Caye site 2	Glovers Reef	Belize	28-Oct-05	16.749	-87.786	Husain, Ellen	9	1.0%				2.1	3.8
1142	Middle Caye site 2	Glovers Reef	Belize	28-Oct-05	16.749	-87.786	Husain, Ellen	9	0.0%				2.1	3.8
1143	Middle Caye site 2	Glovers Reef	Belize	28-Oct-05	16.749	-87.786	Husain, Ellen	9	1.0%				2.1	3.8
1144	Middle Caye site 2	Glovers Reef	Belize	28-Oct-05	16.749	-87.786	Husain, Ellen	9	0.0%				2.1	3.8
1145	Middle Caye site 2	Glovers Reef	Belize	28-Oct-05	16.749	-87.786	Husain, Ellen	9	0.0%				2.1	3.8
1146	Middle Caye site 2	Glovers Reef	Belize	28-Oct-05	16.749	-87.786	Husain, Ellen	9	5.0%				2.1	3.8
1147	Middle Caye site 2	Glovers Reef	Belize	28-Oct-05	16.749	-87.786	Husain, Ellen	9	0.0%				2.1	3.8
1148	Middle Caye site 2	Glovers Reef	Belize	28-Oct-05	16.749	-87.786	Husain, Ellen	9	1.0%				2.1	3.8
1149	Middle Caye site 2	Glovers Reef	Belize	28-Oct-05	16.749	-87.786	Husain, Ellen	9	1.0%				2.1	3.8
1150	Middle Caye site 2	Glovers Reef	Belize	28-Oct-05	16.749	-87.786	Husain, Ellen	9	2.0%				2.1	3.8
1151	Middle Caye site 2	Glovers Reef	Belize	28-Oct-05	16.749	-87.786	Husain, Ellen	9	6.0%				2.1	3.8
1152	Middle Caye site 2	Glovers Reef	Belize	28-Oct-05	16.749	-87.786	Husain, Ellen	9	1.0%				2.1	3.8
1153	Middle Caye site 2	Glovers Reef	Belize	28-Oct-05	16.749	-87.786	Husain, Ellen	9	0.0%				2.1	3.8
1154	West Wall Site 1	Glovers Reef	Belize	21-Nov-05	16.802	-87.860	Husain, Ellen	7	2.0%	30.3%			2.1	3.8
1155	West Wall Site 1	Glovers Reef	Belize	21-Nov-05	16.802	-87.860	Husain, Ellen	7	3.0%				2.1	3.8
1156	West Wall Site 1	Glovers Reef	Belize	21-Nov-05	16.802	-87.860	Husain, Ellen	7	4.0%				2.1	3.8
1157	West Wall Site 1	Glovers Reef	Belize	21-Nov-05	16.802	-87.860	Husain, Ellen	7	0.0%				2.1	3.8
1158	West Wall Site 1	Glovers Reef	Belize	21-Nov-05	16.802	-87.860	Husain, Ellen	7	0.0%				2.1	3.8
1159	West Wall Site 1	Glovers Reef	Belize	21-Nov-05	16.802	-87.860	Husain, Ellen	7	1.0%				2.1	3.8
1160	West Wall Site 1	Glovers Reef	Belize	21-Nov-05	16.802	-87.860	Husain, Ellen	7	2.0%				2.1	3.8
1161	West Wall Site 1	Glovers Reef	Belize	21-Nov-05	16.802	-87.860	Husain, Ellen	7	7.0%				2.1	3.8
1162	West Wall Site 1	Glovers Reef	Belize	21-Nov-05	16.802	-87.860	Husain, Ellen	7	2.0%				2.1	3.8
1163	West Wall Site 1	Glovers Reef	Belize	21-Nov-05	16.802	-87.860	Husain, Ellen	7	4.0%				2.1	3.8
1164	West Wall Site 1	Glovers Reef	Belize	21-Nov-05	16.802	-87.860	Husain, Ellen	7	5.0%				2.1	3.8
1165	West Wall Site 1	Glovers Reef	Belize	21-Nov-05	16.802	-87.860	Husain, Ellen	7	0.0%				2.1	3.8
1166	West Wall Site 1	Glovers Reef	Belize	21-Nov-05	16.802	-87.860	Husain, Ellen	7	3.0%				2.1	3.8

Observation Number	Reef or Site Name	Location	Country	Date (DD-MMM-YY)	Latitude (decimal degrees)	Longitude (decimal degrees)	Primary Contributor	Depth (m)	Cover bleached (%)	Colonies bleached (%)	Cover dead (%)	Colonies dead (%)	Observed DHW (°C-weeks)	Maximum DHW (°C-weeks)
1167	West Wall Site 1	Glovers Reef	Belize	21-Nov-05	16.802	-87.860	Husain, Ellen	7	1.0%				2.1	3.8
1168	West Wall Site 1	Glovers Reef	Belize	21-Nov-05	16.802	-87.860	Husain, Ellen	7	2.0%				2.1	3.8
1169	West Wall Site 1	Glovers Reef	Belize	21-Nov-05	16.802	-87.860	Husain, Ellen	7	1.0%				2.1	3.8
1170	West Wall Site 1	Glovers Reef	Belize	21-Nov-05	16.802	-87.860	Husain, Ellen	7	3.0%				2.1	3.8
1171	West Wall Site 2	Glovers Reef	Belize	21-Nov-05	16.802	-87.860	Husain, Ellen	9	0.0%	32.8%			2.1	3.8
1172	West Wall Site 2	Glovers Reef	Belize	21-Nov-05	16.802	-87.860	Husain, Ellen	9	2.0%				2.1	3.8
1173	West Wall Site 2	Glovers Reef	Belize	21-Nov-05	16.802	-87.860	Husain, Ellen	9	1.0%				2.1	3.8
1174	West Wall Site 2	Glovers Reef	Belize	21-Nov-05	16.802	-87.860	Husain, Ellen	9	4.0%				2.1	3.8
1175	West Wall Site 2	Glovers Reef	Belize	21-Nov-05	16.802	-87.860	Husain, Ellen	9	2.0%				2.1	3.8
1176	West Wall Site 2	Glovers Reef	Belize	21-Nov-05	16.802	-87.860	Husain, Ellen	9	0.0%				2.1	3.8
1177	West Wall Site 2	Glovers Reef	Belize	21-Nov-05	16.802	-87.860	Husain, Ellen	9	4.0%				2.1	3.8
1178	West Wall Site 2	Glovers Reef	Belize	21-Nov-05	16.802	-87.860	Husain, Ellen	9	2.0%				2.1	3.8
1179	West Wall Site 2	Glovers Reef	Belize	21-Nov-05	16.802	-87.860	Husain, Ellen	9	3.0%				2.1	3.8
1180	West Wall Site 2	Glovers Reef	Belize	21-Nov-05	16.802	-87.860	Husain, Ellen	9	2.0%				2.1	3.8
1181	West Wall Site 2	Glovers Reef	Belize	21-Nov-05	16.802	-87.860	Husain, Ellen	9	0.0%				2.1	3.8
1182	West Wall Site 2	Glovers Reef	Belize	21-Nov-05	16.802	-87.860	Husain, Ellen	9	4.0%				2.1	3.8
1183	West Wall Site 2	Glovers Reef	Belize	21-Nov-05	16.802	-87.860	Husain, Ellen	9	4.0%				2.1	3.8
1184	West Wall Site 2	Glovers Reef	Belize	21-Nov-05	16.802	-87.860	Husain, Ellen	9	7.0%				2.1	3.8
1185	West Wall Site 2	Glovers Reef	Belize	21-Nov-05	16.802	-87.860	Husain, Ellen	9	5.0%				2.1	3.8
1186	West Wall Site 2	Glovers Reef	Belize	21-Nov-05	16.802	-87.860	Husain, Ellen	9	5.0%				2.1	3.8
1187	West Wall Site 2	Glovers Reef	Belize	21-Nov-05	16.802	-87.860	Husain, Ellen	9	15.0%				2.1	3.8
1188	West Wall Site 2	Glovers Reef	Belize	21-Nov-05	16.802	-87.860	Husain, Ellen	9	11.0%				2.1	3.8
1189	West Wall Site 3	Glovers Reef	Belize	21-Nov-05	16.802	-87.860	Husain, Ellen	7	0.0%	43.5%			2.1	3.8
1190	West Wall Site 3	Glovers Reef	Belize	21-Nov-05	16.802	-87.860	Husain, Ellen	7	0.0%				2.1	3.8
1191	West Wall Site 3	Glovers Reef	Belize	21-Nov-05	16.802	-87.860	Husain, Ellen	7	3.0%				2.1	3.8
1192	West Wall Site 3	Glovers Reef	Belize	21-Nov-05	16.802	-87.860	Husain, Ellen	7	5.0%				2.1	3.8
1193	West Wall Site 3	Glovers Reef	Belize	21-Nov-05	16.802	-87.860	Husain, Ellen	7	4.0%				2.1	3.8
1194	West Wall Site 3	Glovers Reef	Belize	21-Nov-05	16.802	-87.860	Husain, Ellen	7	0.0%				2.1	3.8
1195	West Wall Site 3	Glovers Reef	Belize	21-Nov-05	16.802	-87.860	Husain, Ellen	7	1.0%				2.1	3.8
1196	West Wall Site 3	Glovers Reef	Belize	21-Nov-05	16.802	-87.860	Husain, Ellen	7	0.0%				2.1	3.8
1197	West Wall Site 3	Glovers Reef	Belize	21-Nov-05	16.802	-87.860	Husain, Ellen	7	6.0%				2.1	3.8
1198	West Wall Site 3	Glovers Reef	Belize	21-Nov-05	16.802	-87.860	Husain, Ellen	7	0.0%				2.1	3.8
1199	West Wall Site 3	Glovers Reef	Belize	21-Nov-05	16.802	-87.860	Husain, Ellen	7	2.0%				2.1	3.8
1200	West Wall Site 3	Glovers Reef	Belize	21-Nov-05	16.802	-87.860	Husain, Ellen	7	13.0%				2.1	3.8
1201	West Wall Site 3	Glovers Reef	Belize	21-Nov-05	16.802	-87.860	Husain, Ellen	7	6.0%				2.1	3.8
1202	West Wall Site 3	Glovers Reef	Belize	21-Nov-05	16.802	-87.860	Husain, Ellen	7	8.0%				2.1	3.8
1203	West Wall Site 3	Glovers Reef	Belize	21-Nov-05	16.802	-87.860	Husain, Ellen	7	1.0%				2.1	3.8
1204	West Wall Site 3	Glovers Reef	Belize	21-Nov-05	16.802	-87.860	Husain, Ellen	7	1.0%				2.1	3.8
1205	West Wall Site 3	Glovers Reef	Belize	21-Nov-05	16.802	-87.860	Husain, Ellen	7	13.0%				2.1	3.8
1206	West Wall Site 3	Glovers Reef	Belize	21-Nov-05	16.802	-87.860	Husain, Ellen	7	5.0%				2.1	3.8
888	Frank's reef	Sapodilla Cayes	Belize	1-Aug-06	16.136	-88.335	Crabbe, James	15	0.0%	0.0%	0.0%	0.0%	0	3.8
889	Nicholas reef	Sapodilla Cayes	Belize	2-Aug-06	16.110	-88.256	Crabbe, James	18	0.0%	0.0%	0.0%	0.0%	0	3.8
890	Barracuda Bank	Port Honduras	Belize	6-Aug-06	16.161	-88.576	Crabbe, James	12	0.0%	0.0%	0.0%	0.0%	0	3.8
1298	CHDE	inshore	Bermuda	1-Jul-05	32.363	-64.695	Jones, Ross	2.7		0.7%	0.0%	0.0%	0	3.3
1299	CHDW	inshore	Bermuda	1-Jul-05	32.362	-64.696	Jones, Ross	2.4		0.6%	0.0%	0.0%	0	3.3
1300	WBBE	inner lagoon	Bermuda	1-Jul-05	32.371	-64.707	Jones, Ross	4		2.2%	0.0%	0.0%	0	3.3
1301	WBBW	inner lagoon	Bermuda	1-Jul-05	32.366	-64.715	Jones, Ross	3.7		0.3%	0.0%	0.0%	0	3.3
1302	THSE	outer lagoon	Bermuda	6-Jul-05	32.425	-64.705	Jones, Ross	7.3		0.2%	0.0%	0.0%	0	3.3
1303	THSW	outer lagoon	Bermuda	6-Jul-05	32.426	-64.726	Jones, Ross	7		0.3%	0.0%	0.0%	0	3.3
1304	CHME	inshore	Bermuda	7-Jul-05	32.353	-64.696	Jones, Ross	6		3.5%	0.0%	0.0%	0	3.3
1305	CHMW	inshore	Bermuda	7-Jul-05	32.352	-64.698	Jones, Ross	4		0.2%	0.0%	0.0%	0	3.3
1306	CHFE	inshore	Bermuda	8-Jul-05	32.354	-64.707	Jones, Ross	3		0.2%	0.0%	0.0%	0	3.3
1307	CHFW	inshore	Bermuda	8-Jul-05	32.350	-64.706	Jones, Ross	3		0.0%	0.0%	0.0%	0	3.3
1308	HBE	north rim	Bermuda	12-Jul-05	32.464	-64.816	Jones, Ross	7.3		0.0%	0.0%	0.0%	0	3.3
1309	HBW	north rim	Bermuda	12-Jul-05	32.459	-64.832	Jones, Ross	7.9		1.1%	0.0%	0.0%	0	3.3
1310	NEBE	north rim	Bermuda	13-Jul-05	32.481	-64.699	Jones, Ross	6.3		0.2%	0.0%	0.0%	0	3.3
1311	NEBW	north rim	Bermuda	13-Jul-05	32.480	-64.704	Jones, Ross	6		0.3%	0.0%	0.0%	0	3.3
1312	TBE	inner lagoon	Bermuda	14-Jul-05	32.313	-64.783	Jones, Ross	5.8		0.0%	0.0%	0.0%	0	3.3

Observation Number	Reef or Site Name	Location	Country	Date (DD-MMM-YY)	Latitude (decimal degrees)	Longitude (decimal degrees)	Primary Contributor	Depth (m)	Cover bleached (%)	Colonies bleached (%)	Cover dead (%)	Colonies dead (%)	Observed DHW (°C-weeks)	Maximum DHW (°C-weeks)
1313	TBW	inner lagoon	Bermuda	14-Jul-05	32.312	-64.790	Jones, Ross	5.8		0.4%	0.0%	0.0%	0	3.3
1314	JSBE	south terrace	Bermuda	19-Jul-05	32.315	-64.709	Jones, Ross	12.2		0.0%	0.0%	0.0%	0	3.3
1315	JSBW	south terrace	Bermuda	19-Jul-05	32.312	-64.710	Jones, Ross	13.7		0.3%	0.0%	0.0%	0	3.3
1316	CHPE	inshore	Bermuda	20-Jul-05	32.510	-64.772	Jones, Ross	4.6		0.3%	0.0%	0.0%	0	3.3
1317	CHPW	inshore	Bermuda	20-Jul-05	32.469	-64.768	Jones, Ross	6		1.6%	0.0%	0.0%	0	3.3
1318	CHGE	south shore	Bermuda	1-Aug-05	32.340	-64.667	Jones, Ross	7.6		0.0%	0.0%	0.0%	0	3.3
1319	CHGW	south shore	Bermuda	1-Aug-05	32.337	-64.672	Jones, Ross	7.6		0.3%	0.0%	0.0%	0	3.3
1320	CREE	outer lagoon	Bermuda	2-Aug-05	32.401	-64.798	Jones, Ross	4.9		0.1%	0.0%	0.0%	0	3.3
1321	CREW	outer lagoon	Bermuda	2-Aug-05	32.401	-64.806	Jones, Ross	4.3		0.1%	0.0%	0.0%	0	3.3
1322	TW2	south slope	Bermuda	8-Aug-05	32.276	-64.766	Jones, Ross	9		0.1%	0.0%	0.0%	0.5	3.3
1323	TW3	south slope	Bermuda	8-Aug-05	32.271	-64.772	Jones, Ross	7.8		0.1%	0.0%	0.0%	0.5	3.3
1324	TW4	south slope	Bermuda	8-Aug-05	32.267	-64.779	Jones, Ross	8.5		0.1%	0.0%	0.0%	0.5	3.3
1325	TW5	south slope	Bermuda	8-Aug-05	32.264	-64.787	Jones, Ross	8		0.0%	0.0%	0.0%	0.5	3.3
1326	CHTE	inshore	Bermuda	9-Aug-05	32.460	-64.885	Jones, Ross	5.8		0.0%	0.0%	0.0%	0.5	3.3
1327	CHTW	inshore	Bermuda	9-Aug-05	32.446	-64.929	Jones, Ross	5		0.0%	0.0%	0.0%	0.5	3.3
1328	SBE	south terrace	Bermuda	10-Aug-05	32.282	-64.756	Jones, Ross	14.6		0.3%	0.0%	0.0%	0.5	3.3
1329	SBW	south terrace	Bermuda	17-Aug-05	32.279	-64.761	Jones, Ross	14		0.1%	0.0%	0.0%	0.5	3.3
1330	TE1	south slope	Bermuda	18-Aug-05	32.286	-64.758	Jones, Ross	7.8		0.1%	0.0%	0.0%	0.5	3.3
1331	TE2	south slope	Bermuda	18-Aug-05	32.288	-64.754	Jones, Ross	8.8		0.3%	0.0%	0.0%	0.5	3.3
1332	TW1	south slope	Bermuda	18-Aug-05	32.280	-64.764	Jones, Ross	8		0.0%	0.0%	0.0%	0.5	3.3
1333	TE3	south slope	Bermuda	19-Aug-05	32.294	-64.746	Jones, Ross	9.8		0.0%	0.0%	0.0%	1.05	3.3
1334	TE4	south slope	Bermuda	19-Aug-05	32.297	-64.741	Jones, Ross	11		0.1%	0.0%	0.0%	1.05	3.3
1335	TE5	south slope	Bermuda	19-Aug-05	32.302	-64.734	Jones, Ross	11		0.3%	0.0%	0.0%	1.05	3.3
1336	WLBE	south terrace	Bermuda	24-Aug-05	32.254	-64.794	Jones, Ross	15.5		0.3%	0.0%	0.0%	1.65	3.3
1337	WLBW	south terrace	Bermuda	24-Aug-05	32.251	-64.804	Jones, Ross	16.5		0.6%	0.0%	0.0%	1.65	3.3
2817	Chub Cut	West Coast	Bermuda	18-Oct-05	32.347	-64.928	Weil, Ernesto	8		0.3%			3.3	3.3
1034	South side of West Dog	West Dog	British Virgin Islands	9-Sep-05	18.481	-64.473	Gore, Shannon	5	5.0%		5.0%		7.3	13.85
1035	Great Harbour	Peter Island	British Virgin Islands	29-Sep-05	18.357	-64.589	Gore, Shannon	15	90.0%				6.7	10.25
2113	Hans Creek, Beef Island	Hans Creek, Beef Island	British Virgin Islands	29-Sep-05	18.433	-61.850	ReefBase	5.5	97.5%				11.75	13.85
1036	Alice in Wonderland	Ginger Island	British Virgin Islands	30-Sep-05	18.386	-64.485	Gore, Shannon	20	95.0%				11.75	13.85
1037	Hans Creek	Beef Island	British Virgin Islands	5-Oct-05	18.441	-64.548	Gore, Shannon	5	95.0%		5.0%		12.65	13.85
2118	The British Virgin Islands	The British Virgin Islands	British Virgin Islands	5-Oct-05	18.453	-64.626	ReefBase	30	90.0%				12.65	13.85
1038	Alice in Wonderland	Ginger Island	British Virgin Islands	7-Oct-05	18.386	-64.485	Gore, Shannon	20	95.0%				13.35	13.85
27	Trellis Bay	Beef Island	British Virgin Islands	18-Oct-05	18.452	-64.530	Baca, Bart	1		36.8%			13.35	13.85
28	Trellis Bay	Beef Island	British Virgin Islands	18-Oct-05	18.452	-64.530	Baca, Bart	2		58.1%			13.35	13.85
29	Trellis Bay	Beef Island	British Virgin Islands	18-Oct-05	18.451	-64.533	Baca, Bart	3		73.5%			13.35	13.85
30	Wells Bay	Beef Island	British Virgin Islands	19-Oct-05	18.441	-64.548	Baca, Bart	3		12.1%			13.35	13.85
1039	Great Harbour	Peter Island	British Virgin Islands	27-Oct-05	18.357	-64.589	Gore, Shannon	15	95.0%				10.25	10.25
1040	Great Harbour	Peter Island	British Virgin Islands	12-Dec-05	18.357	-64.589	Gore, Shannon	15	60.0%				5.6	10.25
2088	Diamond Reef	Great Camanoe	British Virgin Islands	13-Dec-05	18.463	-64.527	Reef Check	10	47.5%	73.8%			4.65	13.85
2089	Diamond Reef	Great Camanoe	British Virgin Islands	13-Dec-05	18.463	-64.527	Reef Check	3	48.8%	66.3%			4.65	13.85
1041	Alice in Wonderland	Ginger Island	British Virgin Islands	14-Dec-05	18.386	-64.485	Gore, Shannon	20	60.0%				4.65	13.85
1042	Hans Creek	Beef Island	British Virgin Islands	14-Dec-05	18.441	-64.548	Gore, Shannon	5	60.0%				4.65	13.85
1043	Black Forest	Peter Island	British Virgin Islands	15-Dec-05	18.350	-64.610	Gore, Shannon	10	60.0%				5.6	10.25
1044	Southern Great Thatch	Great Thatch	British Virgin Islands	15-Dec-05	18.384	-64.735	Gore, Shannon	5	60.0%				5.6	10.25
2090	Bronco Billy	George Dog	British Virgin Islands	15-Dec-05	18.489	-64.490	Reef Check	3	73.8%	83.8%			4.65	13.85
2091	Bronco Billy	George Dog	British Virgin Islands	15-Dec-05	18.489	-64.490	Reef Check	10	48.8%	71.3%			4.65	13.85
2094	Spyglass	Norman Island	British Virgin Islands	31-Dec-05	18.321	-64.615	Reef Check	3	63.8%	83.8%			2.1	10.25
2095	Spyglass	Norman Island	British Virgin Islands	31-Dec-05	18.321	-64.615	Reef Check	10	63.8%	81.3%			2.1	10.25
1045	East side of West Dog	West Dog	British Virgin Islands	7-Jan-06	18.483	-64.471	Gore, Shannon	5	75.0%				0.5	13.85
1027	BVI1-1	Great Thatch Island	British Virgin Islands	17-Jan-06	18.384	-64.735	Ginsburg, Robert	4		70.4%	2.0%	0.0%	0	10.25
1028	BVI1-2	SE Point, Little Thatch Is.	British Virgin Islands	17-Jan-06	18.380	-64.711	Ginsburg, Robert	5		59.4%	1.8%	0.0%	0	10.25
1046	SE Little Thatch	Little Thatch	British Virgin Islands	17-Jan-06	18.380	-64.711	Gore, Shannon	7	20.0%		8.0%		0	10.25
1047	Southern Great Thatch	Great Thatch	British Virgin Islands	17-Jan-06	18.384	-64.735	Gore, Shannon	5	40.0%		13.0%		0	10.25
1029	BVI2-1	Alice in Wonderland buoy, Ging	British Virgin Islands	18-Jan-06	18.386	-64.485	Ginsburg, Robert	20		59.5%	24.1%	4.8%	0	13.85
1030	BVI2-2	Dead Chest North	British Virgin Islands	18-Jan-06	18.369	-64.564	Ginsburg, Robert	7		50.9%	7.5%	0.0%	0	10.25
1031	BVI2-3	Black Forest, Peter Is.	British Virgin Islands	18-Jan-06	18.350	-64.610	Ginsburg, Robert	8.5		71.6%	12.7%	3.0%	0	10.25
1048	Alice in Wonderland	Ginger Island	British Virgin Islands	18-Jan-06	18.386	-64.485	Gore, Shannon	20	40.0%		26.4%		0	13.85

Observation Number	Reef or Site Name	Location	Country	Date (DD-MMM-YY)	Latitude (decimal degrees)	Longitude (decimal degrees)	Primary Contributor	Depth (m)	Cover bleached (%)	Colonies bleached (%)	Cover dead (%)	Colonies dead (%)	Observed DHW (°C-weeks)	Maximum DHW (°C-weeks)
1049	Black Forest	Peter Island	British Virgin Islands	18-Jan-06	18.350	-64.610	Gore, Shannon	10	40.0%		27.1%		0	10.25
1050	Coral Gardens	Dead Chest	British Virgin Islands	18-Jan-06	18.369	-64.564	Gore, Shannon	5	40.0%		26.0%		0	10.25
1032	BVI3-1	Soldier Bay Pt. - Norman Is.	British Virgin Islands	19-Jan-06	18.323	-64.612	Ginsburg, Robert	10.5		67.1%	11.4%	9.6%	0	10.25
1033	BVI3-2	Pelican Island	British Virgin Islands	19-Jan-06	18.332	-64.626	Ginsburg, Robert	8		46.6%	12.7%	9.6%	0	10.25
1051	Pelican Reef	Pelican Island	British Virgin Islands	19-Jan-06	18.332	-64.626	Gore, Shannon	10	40.0%		33.0%		0	10.25
1052	Soldier Bay Point	Norman Island	British Virgin Islands	19-Jan-06	18.323	-64.612	Gore, Shannon	15	40.0%		17.0%		0	10.25
1721	Jigsaw Puzzle	Little Cayman North	Cayman Islands	10-Aug-05	19.672	-80.103	Manfrino, Carrie	4.8		9.0%			0	4.35
1722	West Point	Little Cayman South	Cayman Islands	10-Aug-05	19.654	-80.104	Manfrino, Carrie	12.4		21.6%			0	4.35
1723	Coral City	Little Cayman South	Cayman Islands	12-Aug-05	19.681	-80.023	Manfrino, Carrie	11		11.8%			0.5	4.35
1724	Grundy's Gardens	Little Cayman South	Cayman Islands	12-Aug-05	19.657	-80.090	Manfrino, Carrie	10		17.5%			0.5	4.35
1725	Paul's Anchor	Little Cayman North	Cayman Islands	13-Aug-05	19.694	-80.069	Manfrino, Carrie	11.2		12.6%			0.5	4.35
1726	Snapshot	Little Cayman North	Cayman Islands	13-Aug-05	19.701	-80.057	Manfrino, Carrie	9.4		13.0%			0.5	4.35
1727	Mixing Bowl/Three Fathom Wa	Little Cayman North	Cayman Islands	15-Aug-05	19.685	-80.078	Manfrino, Carrie	13		10.1%			0.5	4.35
1728	Richard's Reef	Little Cayman South	Cayman Islands	15-Aug-05	19.657	-80.097	Manfrino, Carrie	10		9.2%			0.5	4.35
2176	Wild life	San Andrés	Colombia - Antilles	12-Sep-05	12.513	-81.731	Rodriguez, Alberto	4.5	3.0%	6.8%	0.0%	0.0%	4.4	8.2
2177	Wild life	San Andrés	Colombia - Antilles	12-Sep-05	12.513	-81.732	Rodriguez, Alberto	10.5	5.0%	14.2%	0.0%	0.0%	4.4	8.2
2178	Bajo Bonito	San Andrés	Colombia - Antilles	17-Sep-05	12.300	-81.717	Rodriguez, Alberto	11.25	2.0%	11.9%	0.0%	0.0%	3.8	8.2
2179	Bajo Bonito	San Andrés	Colombia - Antilles	17-Sep-05	12.300	-81.717	Rodriguez, Alberto	15	1.5%	42.9%	0.0%	0.0%	3.8	8.2
2180	Barco Hundido	San Andrés	Colombia - Antilles	17-Sep-05	12.537	-81.736	Rodriguez, Alberto	22.5	4.0%	20.5%	0.0%	0.0%	3.8	8.2
2181	Barco Hundido	San Andrés	Colombia - Antilles	17-Sep-05	12.537	-81.736	Rodriguez, Alberto	10.5	4.0%	53.8%	0.0%	0.0%	3.8	8.2
2182	Reggae nest	San Andrés	Colombia - Antilles	17-Sep-05	12.590	-81.715	Rodriguez, Alberto	22.5	1.0%	29.3%	0.0%	0.0%	3.8	8.2
2183	Reggae nest	San Andrés	Colombia - Antilles	17-Sep-05	12.590	-81.715	Rodriguez, Alberto	11.25	2.5%	60.0%	0.0%	0.0%	3.8	8.2
2184	West Ponit	San Andrés	Colombia - Antilles	17-Sep-05	12.496	-81.738	Rodriguez, Alberto	19.5	8.0%	47.4%	0.0%	0.0%	3.8	8.2
2185	West Ponit	San Andrés	Colombia - Antilles	17-Sep-05	12.496	-81.738	Rodriguez, Alberto	12	3.0%	29.4%	0.0%	0.0%	3.8	8.2
2186	Blue Hole	San Andrés	Colombia - Antilles	18-Sep-05	12.498	-81.715	Rodriguez, Alberto	8.5	1.0%	15.0%	0.0%	0.0%	3.2	8.2
2187	Blue Hole	San Andrés	Colombia - Antilles	18-Sep-05	12.498	-81.715	Rodriguez, Alberto	19.5	3.0%	41.7%	0.0%	0.0%	3.2	8.2
2188	Little reef	San Andrés	Colombia - Antilles	18-Sep-05	12.585	-81.686	Rodriguez, Alberto	4.5	2.0%	8.2%	0.0%	0.0%	3.2	8.2
2189	Piramide	San Andrés	Colombia - Antilles	18-Sep-05	12.584	-81.698	Rodriguez, Alberto	6	1.0%	27.5%	0.0%	0.0%	3.2	8.2
2190	Trampa tortugas	San Andrés	Colombia - Antilles	18-Sep-05	12.538	-81.686	Rodriguez, Alberto	10	2.0%	6.9%	0.0%	0.0%	3.2	8.2
2191	Trampa tortugas	San Andrés	Colombia - Antilles	18-Sep-05	12.538	-81.686	Rodriguez, Alberto	18	1.0%	8.5%	0.0%	0.0%	3.2	8.2
2032	South West Reef	San Andreas Is	Colombia - Antilles	5-Nov-05	12.521	-81.734	Quinn, Norman	10	25.0%	25.0%	5.0%	2.5%	2.2	8.2
2033	Southern Wall	San Andreas Is	Colombia - Antilles	5-Nov-05	12.499	-81.714	Quinn, Norman	15	50.0%	50.0%	0.0%	0.0%	2.2	8.2
2034	Northern reef	San Andreas Is	Colombia - Antilles	9-Nov-05	12.601	-81.708	Quinn, Norman	20	20.0%	20.0%	0.0%	0.0%	2.2	8.2
2200	Bajo San Felipe	Providencia	Colombia - Antilles	13-Nov-05	13.364	-81.405	Rodriguez, Alberto	5	3.0%	12.7%	0.0%	0.0%	4.1	4.9
2201	Espiral	Providencia	Colombia - Antilles	13-Nov-05	13.389	-81.418	Rodriguez, Alberto	23	1.0%	3.3%	0.0%	0.0%	4.1	4.9
2202	Manta City	Providencia	Colombia - Antilles	13-Nov-05	13.315	-81.374	Rodriguez, Alberto	6.5	2.0%	13.3%	0.0%	0.0%	4.1	4.9
2203	Manzanillo-barrera	Providencia	Colombia - Antilles	13-Nov-05	13.310	-81.372	Rodriguez, Alberto	4	1.0%	0.6%	0.0%	0.0%	4.1	4.9
2204	Tete´s place	Providencia	Colombia - Antilles	13-Nov-05	13.328	-81.412	Rodriguez, Alberto	7	1.0%	4.0%	0.0%	0.0%	4.1	4.9
2205	Basalto y palmera	Providencia	Colombia - Antilles	14-Nov-05	13.400	-81.371	Rodriguez, Alberto	3	1.0%	3.7%	0.0%	0.0%	3.05	4.8
2206	Basalto y palmera	Providencia	Colombia - Antilles	14-Nov-05	13.402	-81.371	Rodriguez, Alberto	7	2.0%	5.8%	0.0%	0.0%	3.05	4.8
2207	Canal	Providencia	Colombia - Antilles	14-Nov-05	13.399	-81.397	Rodriguez, Alberto	3	1.0%	2.5%	0.0%	0.0%	3.05	4.8
2208	Canal	Providencia	Colombia - Antilles	14-Nov-05	13.389	-81.384	Rodriguez, Alberto	7	3.0%	16.7%	0.0%	0.0%	3.05	4.8
2209	Maracaibo	Providencia	Colombia - Antilles	14-Nov-05	13.378	-81.353	Rodriguez, Alberto	4	1.0%	5.4%	0.0%	0.0%	3.05	4.8
2210	Pinaculos	Providencia	Colombia - Antilles	14-Nov-05	13.412	-81.334	Rodriguez, Alberto	5	1.0%	2.6%	0.0%	0.0%	3.05	4.8
2216	Canal	Providencia	Colombia - Antilles	28-Nov-05	13.399	-81.397	Rodriguez, Alberto	11	1.0%	27.8%	0.0%	0.0%	3.05	4.8
2217	Canal	Providencia	Colombia - Antilles	28-Nov-05	13.399	-81.397	Rodriguez, Alberto	11	1.0%	34.8%	0.0%	0.0%	3.05	4.8
2218	Confusion	Providencia	Colombia - Antilles	28-Nov-05	13.397	-81.405	Rodriguez, Alberto	19	3.0%	27.8%	0.0%	0.0%	4.1	4.9
2219	Confusion	Providencia	Colombia - Antilles	28-Nov-05	13.397	-81.405	Rodriguez, Alberto	19	3.0%	35.0%	0.0%	0.0%	4.1	4.9
2220	The Bar	Providencia	Colombia - Antilles	28-Nov-05	13.372	-81.403	Rodriguez, Alberto	13.5	10.0%	23.5%	0.0%	0.0%	4.1	4.9
2221	The Bar	Providencia	Colombia - Antilles	28-Nov-05	13.372	-81.403	Rodriguez, Alberto	13.5	10.0%	40.9%	0.0%	0.0%	4.1	4.9
2222	Bight	Providencia	Colombia - Antilles	29-Nov-05	13.506	-81.367	Rodriguez, Alberto	19	5.0%	31.6%	0.0%	0.0%	3.05	4.8
2223	Bight	Providencia	Colombia - Antilles	29-Nov-05	13.506	-81.367	Rodriguez, Alberto	19	5.0%	54.2%	0.0%	0.0%	3.05	4.8
2224	Rock Deep Shoald	Providencia	Colombia - Antilles	29-Nov-05	13.516	-81.349	Rodriguez, Alberto	16	1.0%	9.1%	0.0%	0.0%	3.05	4.8
2225	Rock Deep Shoald	Providencia	Colombia - Antilles	29-Nov-05	13.516	-81.349	Rodriguez, Alberto	16	1.0%	8.3%	0.0%	0.0%	3.05	4.8
2226	Tapoo Point	Providencia	Colombia - Antilles	29-Nov-05	13.458	-81.364	Rodriguez, Alberto	7.5	1.0%	18.5%	0.0%	0.0%	3.05	4.8
2227	Tapoo Point	Providencia	Colombia - Antilles	29-Nov-05	13.458	-81.364	Rodriguez, Alberto	7.5	1.0%	30.0%	0.0%	0.0%	3.05	4.8
2228	Bajo de San Felipe	Providencia	Colombia - Antilles	30-Nov-05	13.365	-81.404	Rodriguez, Alberto	9.5	5.0%	8.0%	0.0%	0.0%	4.1	4.9
2229	Bajo de San Felipe	Providencia	Colombia - Antilles	30-Nov-05	13.365	-81.404	Rodriguez, Alberto	9.5	5.0%	45.0%	0.0%	0.0%	4.1	4.9

Observation Number	Reef or Site Name	Location	Country	Date (DD-MMM-YY)	Latitude (decimal degrees)	Longitude (decimal degrees)	Primary Contributor	Depth (m)	Cover bleached (%)	Colonies bleached (%)	Cover dead (%)	Colonies dead (%)	Observed DHW (°C-weeks)	Maximum DHW (°C-weeks)
2230	Manta's Place	Providencia	Colombia - Antilles	30-Nov-05	13.315	-81.377	Rodriguez, Alberto	11	1.0%	12.0%	0.0%	0.0%	4.1	4.9
2231	Manta's Place	Providencia	Colombia - Antilles	30-Nov-05	13.315	-81.377	Rodriguez, Alberto	11	1.0%	31.8%	0.0%	0.0%	4.1	4.9
2232	Margarita's Place	Providencia	Colombia - Antilles	30-Nov-05	13.326	-81.360	Rodriguez, Alberto	9.5	5.0%	11.1%	0.0%	0.0%	4.1	4.9
2233	Margarita's Place	Providencia	Colombia - Antilles	30-Nov-05	13.326	-81.360	Rodriguez, Alberto	9.5	5.0%	18.2%	0.0%	0.0%	4.1	4.9
2240	Emisario	San Andrés	Colombia - Antilles	16-Dec-05	12.569	-81.726	Rodriguez, Alberto	11	5.0%	25.0%	0.0%	0.0%	1.65	8.2
2241	Emisario	San Andrés	Colombia - Antilles	16-Dec-05	12.569	-81.726	Rodriguez, Alberto	11	5.0%	43.8%	0.0%	0.0%	1.65	8.2
2242	Piedras de Carlitos	San Andrés	Colombia - Antilles	16-Dec-05	12.502	-81.710	Rodriguez, Alberto	9.5	1.0%	18.5%	0.0%	0.0%	1.65	8.2
2243	Piedras de Carlitos	San Andrés	Colombia - Antilles	16-Dec-05	12.502	-81.710	Rodriguez, Alberto	9.5	1.0%	23.1%	0.0%	0.0%	1.65	8.2
2244	Cantil de Bajo Bonito	San Andrés	Colombia - Antilles	17-Dec-05	12.587	-81.717	Rodriguez, Alberto	19	2.0%	35.7%	0.0%	0.0%	1.65	8.2
2245	Cantil de Bajo Bonito	San Andrés	Colombia - Antilles	17-Dec-05	12.587	-81.717	Rodriguez, Alberto	19	2.0%	34.5%	0.0%	0.0%	1.65	8.2
2246	Montañitas	San Andrés	Colombia - Antilles	17-Dec-05	12.590	-81.714	Rodriguez, Alberto	16	15.0%	25.9%	0.0%	0.0%	1.65	8.2
2247	Montañitas	San Andrés	Colombia - Antilles	17-Dec-05	12.590	-81.714	Rodriguez, Alberto	16	15.0%	36.0%	0.0%	0.0%	1.65	8.2
2248	Trampa tortugas	San Andrés	Colombia - Antilles	17-Dec-05	12.542	-81.680	Rodriguez, Alberto	16	1.0%	29.2%	0.0%	0.0%	1.65	8.2
2249	Trampa tortugas	San Andrés	Colombia - Antilles	17-Dec-05	12.542	-81.680	Rodriguez, Alberto	16	1.0%	26.7%	0.0%	0.0%	1.65	8.2
2263	Iguana	San Andrés	Colombia - Antilles	13-Sep-06	12.500	-81.733	Rodriguez, Alberto	4.5	2.0%	7.4%	0.0%	0.0%	0	8.2
2264	Iguana	San Andrés	Colombia - Antilles	14-Sep-06	12.500	-81.733	Rodriguez, Alberto	10.5	5.0%	20.5%	0.0%	0.0%	0	8.2
2265	Iguana	San Andrés	Colombia - Antilles	14-Sep-06	12.501	-81.734	Rodriguez, Alberto	16.5	5.0%	19.9%	0.0%	0.0%	0	8.2
2266	Wild life	San Andrés	Colombia - Antilles	16-Sep-06	12.513	-81.733	Rodriguez, Alberto	16.5	5.0%	23.5%	0.0%	0.0%	0	8.2
2267	Wild life	San Andrés	Colombia - Antilles	18-Sep-06	12.513	-81.731	Rodriguez, Alberto	4.5	1.0%	3.9%	0.0%	0.0%	0	8.2
2268	Iguana	San Andrés	Colombia - Antilles	19-Sep-06	12.500	-81.733	Rodriguez, Alberto	10.5	1.0%	3.6%	0.0%	0.0%	0	8.2
2269	Wild life	San Andrés	Colombia - Antilles	19-Sep-06	12.513	-81.732	Rodriguez, Alberto	10.5	1.0%	3.7%	0.0%	0.0%	0	8.2
2270	Iguana	San Andrés	Colombia - Antilles	21-Sep-06	12.500	-81.733	Rodriguez, Alberto	4.5	1.0%	1.0%	0.0%	0.0%	0	8.2
2271	Little reef	San Andrés	Colombia - Antilles	21-Sep-06	12.585	-81.686	Rodriguez, Alberto	4.5	1.0%	1.8%	0.0%	0.0%	0	8.2
2272	The Bar	Providencia	Colombia - Antilles	26-Sep-06	13.372	-81.403	Rodriguez, Alberto	10.5	1.0%	1.0%	0.0%	0.0%	0	4.9
2273	Canal	Providencia	Colombia - Antilles	27-Sep-06	13.389	-81.384	Rodriguez, Alberto	7	1.0%	4.0%	0.0%	0.0%	0	4.8
2274	Canal	Providencia	Colombia - Antilles	29-Sep-06	13.399	-81.397	Rodriguez, Alberto	4.5	1.0%	0.3%	0.0%	0.0%	0	4.8
2275	The Bar	Providencia	Colombia - Antilles	29-Sep-06	13.372	-81.403	Rodriguez, Alberto	4.5	1.0%	0.8%	0.0%	0.0%	0	4.9
2131	Isla Ceycen	San Bernardo Islands	Colombia - Mainland	2-Aug-05	9.706	-75.866	Rodriguez, Alberto	14.5	0.5%	47.2%	0.0%	0.0%	9.6	12.7
2132	Isla Ceycen	San Bernardo Islands	Colombia - Mainland	2-Aug-05	9.706	-75.866	Rodriguez, Alberto	8.5	10.0%	81.5%	0.0%	0.0%	9.6	12.7
2133	Isla Ceycen	San Bernardo Islands	Colombia - Mainland	2-Aug-05	9.706	-75.866	Rodriguez, Alberto	4.5	50.0%	95.8%	5.0%	8.3%	9.6	12.7
2134	N Isla Mucura	San Bernardo Islands	Colombia - Mainland	2-Aug-05	9.795	-75.866	Rodriguez, Alberto	5	30.0%	96.7%	5.0%	13.3%	9.6	12.7
2135	N Isla Mucura	San Bernardo Islands	Colombia - Mainland	2-Aug-05	9.795	-75.866	Rodriguez, Alberto	7	40.0%	100.0%	5.0%	25.0%	9.6	12.7
2136	N Isla Mucura	San Bernardo Islands	Colombia - Mainland	2-Aug-05	9.795	-75.866	Rodriguez, Alberto	2	30.0%	87.2%	4.0%	5.1%	9.6	12.7
2137	Bajo Tio Solda	San Bernardo Islands	Colombia - Mainland	3-Aug-05	9.822	-75.891	Rodriguez, Alberto	11.5	51.0%	62.2%	0.0%	0.0%	9.6	12.7
2138	Bajo Tio Solda	San Bernardo Islands	Colombia - Mainland	3-Aug-05	9.822	-75.891	Rodriguez, Alberto	8	25.0%	73.8%	0.0%	2.4%	9.6	12.7
2139	Isla Mangle	San Bernardo Islands	Colombia - Mainland	3-Aug-05	9.779	-75.786	Rodriguez, Alberto	10	25.0%	63.2%	0.0%	0.0%	9.6	12.7
2140	Isla Mangle	San Bernardo Islands	Colombia - Mainland	3-Aug-05	9.779	-75.786	Rodriguez, Alberto	6.5	35.0%	64.3%	2.0%	0.0%	9.6	12.7
2141	Isla Mangle	San Bernardo Islands	Colombia - Mainland	3-Aug-05	9.775	-75.787	Rodriguez, Alberto	3.5	70.0%	96.2%	5.0%	17.0%	9.6	12.7
2142	W Isla Maravilla	San Bernardo Islands	Colombia - Mainland	3-Aug-05	9.761	-75.879	Rodriguez, Alberto	3.5	40.0%	79.2%	2.0%	10.4%	9.6	12.7
2143	W Isla Maravilla	San Bernardo Islands	Colombia - Mainland	3-Aug-05	9.761	-75.879	Rodriguez, Alberto	1.5	50.0%		5.0%		9.6	12.7
2144	NE Isla Tintipan	San Bernardo Islands	Colombia - Mainland	4-Aug-05	9.813	-75.836	Rodriguez, Alberto	11.5	5.0%	50.0%	1.0%	0.0%	9.6	12.7
2145	NE Isla Tintipan	San Bernardo Islands	Colombia - Mainland	4-Aug-05	9.813	-75.836	Rodriguez, Alberto	5	30.0%	86.4%	1.0%	6.8%	9.6	12.7
2146	NE Isla Tintipan	San Bernardo Islands	Colombia - Mainland	4-Aug-05	9.807	-75.833	Rodriguez, Alberto	1.5	60.0%	96.8%	5.0%	20.6%	9.6	12.7
2168	NW Isla Tintipan	San Bernardo Islands	Colombia - Mainland	4-Aug-05	9.813	-75.849	Rodriguez, Alberto	11.5	7.0%	69.8%	0.0%	0.0%	9.6	12.7
2147	Isla Tesoro	Rosario Islands	Colombia - Mainland	5-Aug-05	10.238	-75.737	Rodriguez, Alberto	5.5	20.0%	69.2%	0.2%	0.0%	10.3	12.7
2148	Isla Tesoro	Rosario Islands	Colombia - Mainland	5-Aug-05	10.238	-75.737	Rodriguez, Alberto	10.75	3.0%	70.7%	0.0%	0.0%	10.3	12.7
2149	N Isla Grande	Rosario Islands	Colombia - Mainland	5-Aug-05	10.186	-75.745	Rodriguez, Alberto	11.5	0.5%	28.9%	0.5%	0.0%	10.3	12.7
2150	N Isla Grande	Rosario Islands	Colombia - Mainland	5-Aug-05	10.186	-75.745	Rodriguez, Alberto	7	2.0%	64.3%	0.0%	0.0%	10.3	12.7
2151	N Isla Grande	Rosario Islands	Colombia - Mainland	5-Aug-05	10.184	-75.731	Rodriguez, Alberto	1.5	90.0%	100.0%	8.0%	17.6%	10.3	12.7
2152	N Isla Tesoro	Rosario Islands	Colombia - Mainland	5-Aug-05	10.238	-75.737	Rodriguez, Alberto	11.5	0.5%	52.1%	0.0%	0.0%	10.3	12.7
2153	N Isla Tesoro	Rosario Islands	Colombia - Mainland	5-Aug-05	10.238	-75.737	Rodriguez, Alberto	7.5	15.0%	76.9%	0.0%	0.0%	10.3	12.7
2154	N Isla Tesoro	Rosario Islands	Colombia - Mainland	5-Aug-05	10.238	-75.737	Rodriguez, Alberto	1.5	5.0%	79.5%	0.0%	0.0%	10.3	12.7
2169	NW Isla Tintipan	San Bernardo Islands	Colombia - Mainland	5-Aug-05	9.813	-75.849	Rodriguez, Alberto	7	30.0%	85.5%	1.0%	1.8%	10.3	12.7
2155	La Coca Reef, Isla Rosario	Rosario Islands	Colombia - Mainland	6-Aug-05	10.167	-75.786	Rodriguez, Alberto	10	3.0%	18.6%	0.5%	0.0%	10.3	12.7
2156	La Coca Reef, Isla Rosario	Rosario Islands	Colombia - Mainland	6-Aug-05	10.167	-75.786	Rodriguez, Alberto	7	40.0%	42.3%	1.0%	0.0%	10.3	12.7
2157	NW Isla Grande	Rosario Islands	Colombia - Mainland	6-Aug-05	10.186	-75.745	Rodriguez, Alberto	10.5	1.0%	30.9%	0.5%	3.6%	10.3	12.7
2158	SW Isla Rosario	Rosario Islands	Colombia - Mainland	6-Aug-05	10.155	-75.803	Rodriguez, Alberto	12	1.0%	34.0%	0.0%	0.0%	10.3	12.7
2159	SW Isla Rosario	Rosario Islands	Colombia - Mainland	6-Aug-05	10.155	-75.803	Rodriguez, Alberto	9.5	50.0%	54.2%	0.5%	2.1%	10.3	12.7

Observation Number	Reef or Site Name	Location	Country	Date (DD-MMM-YY)	Latitude (decimal degrees)	Longitude (decimal degrees)	Primary Contributor	Depth (m)	Cover bleached (%)	Colonies bleached (%)	Cover dead (%)	Colonies dead (%)	Observed DHW (°C-weeks)	Maximum DHW (°C-weeks)
2160	SW Isla Rosario	Rosario Islands	Colombia - Mainland	6-Aug-05	10.155	-75.788	Rodriguez, Alberto	1.5	80.0%	69.7%	2.0%	15.2%	10.3	12.7
2167	NW Isla Grande	Rosario Islands	Colombia - Mainland	6-Aug-05	10.186	-75.745	Rodriguez, Alberto	8	10.0%	57.7%	0.5%	0.0%	10.3	12.7
2161	Bajos del medio	Rosario Islands	Colombia - Mainland	7-Aug-05	10.160	-75.740	Rodriguez, Alberto	10.5	5.0%	55.8%	0.0%	0.0%	10.3	12.7
2162	Bajos del medio	Rosario Islands	Colombia - Mainland	7-Aug-05	10.160	-75.740	Rodriguez, Alberto	5	15.0%	53.0%	0.5%	0.0%	10.3	12.7
2163	Bajos del medio	Rosario Islands	Colombia - Mainland	7-Aug-05	10.160	-75.740	Rodriguez, Alberto	3	70.0%	92.5%	0.5%	22.5%	10.3	12.7
2164	Isla Pavitos	Rosario Islands	Colombia - Mainland	7-Aug-05	10.175	-75.771	Rodriguez, Alberto	10	7.0%	32.0%	0.0%	0.0%	10.3	12.7
2165	Isla Pavitos	Rosario Islands	Colombia - Mainland	7-Aug-05	10.175	-75.771	Rodriguez, Alberto	6.5	12.0%	69.8%	0.0%	0.0%	10.3	12.7
2166	Isla Pavitos	Rosario Islands	Colombia - Mainland	7-Aug-05	10.175	-75.771	Rodriguez, Alberto	2.5	85.0%	89.1%	0.5%	6.5%	10.3	12.7
2170	Chengue Bay	Santa Marta-Tayrona Park Area	Colombia - Mainland	12-Aug-05	11.328	-74.132	Rodriguez, Alberto	6	0.0%		0.0%		4.1	6.2
2171	Granate Cove	Santa Marta-Tayrona Park Area	Colombia - Mainland	12-Aug-05	11.300	-74.197	Rodriguez, Alberto	8	0.0%		0.0%		4.1	6.2
2172	Chengue Bay	Santa Marta-Tayrona Park Area	Colombia - Mainland	23-Aug-05	11.328	-74.132	Rodriguez, Alberto	6	0.0%		0.0%		2.95	6.2
2173	Granate Cove	Santa Marta-Tayrona Park Area	Colombia - Mainland	24-Aug-05	11.300	-74.197	Rodriguez, Alberto	8	0.0%		0.0%		2.95	6.2
2174	Chengue Bay	Santa Marta-Tayrona Park Area	Colombia - Mainland	5-Sep-05	11.328	-74.132	Rodriguez, Alberto	6	0.0%		0.0%		2.95	6.2
2175	Granate Cove	Santa Marta-Tayrona Park Area	Colombia - Mainland	5-Sep-05	11.300	-74.197	Rodriguez, Alberto	8	0.0%		0.0%		2.95	6.2
2408	Isla Baru	Punta Gigante	Colombia - Mainland	28-Sep-05	10.250	-75.630	Sánchez Muñoz, Juan Armando	12	25.0%				6.5	12.7
2409	Isla Baru	Punta Gigante	Colombia - Mainland	28-Sep-05	10.250	-75.630	Sánchez Muñoz, Juan Armando	12	100.0%				6.5	12.7
2410	Isla Baru	Punta Gigante	Colombia - Mainland	28-Sep-05	10.250	-75.630	Sánchez Muñoz, Juan Armando	12	100.0%				6.5	12.7
2411	Isla Baru	Punta Gigante	Colombia - Mainland	28-Sep-05	10.250	-75.630	Sánchez Muñoz, Juan Armando	12	25.0%				6.5	12.7
2192	Neguange Bay	Santa Marta-Tayrona Park Area	Colombia - Mainland	1-Oct-05	11.323	-74.081	Rodriguez, Alberto	5	0.0%		0.0%		0	6.2
2193	Taganga Bay	Santa Marta-Tayrona Park Area	Colombia - Mainland	2-Oct-05	11.272	-74.201	Rodriguez, Alberto	4.5	0.0%		0.0%		0	6.2
2412	Isla Baru	Pendales	Colombia - Mainland	7-Oct-05	10.240	-75.620	Sánchez Muñoz, Juan Armando	6	75.0%				6	12.7
2413	Isla Baru	Pendales	Colombia - Mainland	7-Oct-05	10.240	-75.620	Sánchez Muñoz, Juan Armando	6	50.0%				6	12.7
2414	Isla Baru	Pendales	Colombia - Mainland	7-Oct-05	10.240	-75.620	Sánchez Muñoz, Juan Armando	6	90.0%				6	12.7
2415	Isla Baru	Pendales	Colombia - Mainland	7-Oct-05	10.240	-75.620	Sánchez Muñoz, Juan Armando	6	75.0%				6	12.7
2416	Isla Baru	Pendales	Colombia - Mainland	7-Oct-05	10.240	-75.620	Sánchez Muñoz, Juan Armando	6	85.0%				6	12.7
2417	Isla Baru	Pendales	Colombia - Mainland	7-Oct-05	10.240	-75.620	Sánchez Muñoz, Juan Armando	6	90.0%				6	12.7
2418	Isla Baru	Pendales	Colombia - Mainland	7-Oct-05	10.240	-75.620	Sánchez Muñoz, Juan Armando	6	95.0%				6	12.7
2419	Isla Baru	Pendales	Colombia - Mainland	7-Oct-05	10.240	-75.620	Sánchez Muñoz, Juan Armando	6	80.0%				6	12.7
2420	Isla Baru	Pendales	Colombia - Mainland	7-Oct-05	10.240	-75.620	Sánchez Muñoz, Juan Armando	6	10.0%				6	12.7
2194	Gayraca	Santa Marta-Tayrona Park Area	Colombia - Mainland	19-Oct-05	11.339	-74.112	Rodriguez, Alberto	12	0.0%				2.35	6.2
2195	Gayraca	Santa Marta-Tayrona Park Area	Colombia - Mainland	19-Oct-05	11.331	-74.117	Rodriguez, Alberto	12	0.0%				2.35	6.2
2196	Neguange Bay	Santa Marta-Tayrona Park Area	Colombia - Mainland	21-Oct-05	11.323	-74.081	Rodriguez, Alberto	5	10.0%		0.0%		2.95	6.2
2197	Neguange Bay	Santa Marta-Tayrona Park Area	Colombia - Mainland	27-Oct-05	11.323	-74.081	Rodriguez, Alberto	5	15.0%		0.0%		4.6	6.2
2198	Neguange Bay	Santa Marta-Tayrona Park Area	Colombia - Mainland	11-Nov-05	11.323	-74.081	Rodriguez, Alberto	5	20.0%		0.0%		6.2	6.2
2199	Taganga Bay	Santa Marta-Tayrona Park Area	Colombia - Mainland	12-Nov-05	11.272	-74.201	Rodriguez, Alberto	4.5	30.0%		0.0%		6.2	6.2
2211	Chengue	Santa Marta-Tayrona Park Area	Colombia - Mainland	21-Nov-05	11.326	-74.128	Rodriguez, Alberto	4.5	2.0%	7.7%	0.0%	0.0%	6.2	6.2
2212	Chengue	Santa Marta-Tayrona Park Area	Colombia - Mainland	21-Nov-05	11.326	-74.128	Rodriguez, Alberto	10.5	2.0%	10.9%	0.0%	0.0%	6.2	6.2
2213	Chengue	Santa Marta-Tayrona Park Area	Colombia - Mainland	23-Nov-05	11.330	-74.129	Rodriguez, Alberto	4.5	1.0%	8.0%	0.0%	0.0%	6.2	6.2
2214	Chengue	Santa Marta-Tayrona Park Area	Colombia - Mainland	23-Nov-05	11.330	-74.129	Rodriguez, Alberto	10.5	1.0%	4.6%	0.0%	0.0%	6.2	6.2
2215	Chengue	Santa Marta-Tayrona Park Area	Colombia - Mainland	25-Nov-05	11.330	-74.129	Rodriguez, Alberto	13.5	1.0%	3.0%	0.0%	0.0%	6.2	6.2
2234	Calichán-Aguja	Santa Marta-Tayrona Park Area	Colombia - Mainland	1-Dec-05	11.312	-74.195	Rodriguez, Alberto	8	1.0%	15.6%	0.0%	0.0%	6.2	6.2
2235	Granate Cove	Santa Marta-Tayrona Park Area	Colombia - Mainland	1-Dec-05	11.300	-74.197	Rodriguez, Alberto	8	1.0%		0.0%		6.2	6.2
2236	Granate-Cantil	Santa Marta-Tayrona Park Area	Colombia - Mainland	1-Dec-05	11.302	-74.195	Rodriguez, Alberto	8	5.0%	15.6%	0.0%	0.0%	6.2	6.2
2237	Chengue	Santa Marta-Tayrona Park Area	Colombia - Mainland	7-Dec-05	11.326	-74.128	Rodriguez, Alberto	14	1.0%	5.4%	0.0%	0.0%	6.2	6.2
2238	Chengue Bay	Santa Marta-Tayrona Park Area	Colombia - Mainland	12-Dec-05	11.328	-74.132	Rodriguez, Alberto	6	20.0%		0.0%		6.2	6.2
2239	Gayraca	Santa Marta-Tayrona Park Area	Colombia - Mainland	14-Dec-05	11.327	-74.115	Rodriguez, Alberto	4.5	1.0%	15.0%	0.0%	0.0%	6.2	6.2
2250	Playa Blanca	Rodadero-Santa Marta	Colombia - Mainland	26-Dec-05	11.219	-74.241	Rodriguez, Alberto	8	5.0%	9.0%	1.0%	1.0%	6.2	6.2
2251	Taganga Bay	Santa Marta-Tayrona Park Area	Colombia - Mainland	30-Dec-05	11.272	-74.201	Rodriguez, Alberto	4.5	20.0%	41.2%	1.0%	10.0%	5.55	6.2
2252	Neguange Bay	Santa Marta-Tayrona Park Area	Colombia - Mainland	18-Jan-06	11.323	-74.081	Rodriguez, Alberto	5	5.0%	23.3%	1.0%	0.5%	2.4	6.2
2253	Isla Tesoro	Rosario Islands	Colombia - Mainland	6-Feb-06	10.234	-75.746	Rodriguez, Alberto	10.5	1.0%	11.6%	1.0%		0	12.7
2254	Isla Tesoro	Rosario Islands	Colombia - Mainland	6-Feb-06	10.234	-75.746	Rodriguez, Alberto	5.5	1.0%	19.7%	1.0%		0	12.7
2255	Isla Pavitos	Rosario Islands	Colombia - Mainland	7-Feb-06	10.175	-75.771	Rodriguez, Alberto	2.5	1.0%	8.7%	3.0%		0	12.7
2256	Isla Pavitos	Rosario Islands	Colombia - Mainland	8-Feb-06	10.175	-75.771	Rodriguez, Alberto	10	1.0%	1.0%	3.0%		0	12.7
2257	Bajo Tio Solda	San Bernardo Islands	Colombia - Mainland	11-Feb-06	9.822	-75.891	Rodriguez, Alberto	10.5	1.0%	5.8%	2.0%		0	12.7
2258	Isla Ceycen	San Bernardo Islands	Colombia - Mainland	11-Feb-06	9.706	-75.866	Rodriguez, Alberto	10.5	1.0%	1.0%	0.0%		0	12.7
2259	Isla Ceycen	San Bernardo Islands	Colombia - Mainland	11-Feb-06	9.706	-75.866	Rodriguez, Alberto	4.5	1.0%	3.8%	5.0%		0	12.7
2260	Bajo Minalta	San Bernardo Islands	Colombia - Mainland	12-Feb-06	9.791	-75.923	Rodriguez, Alberto	4.5	1.0%	0.3%	2.0%		0	12.7
2261	Isla Mangle	San Bernardo Islands	Colombia - Mainland	12-Feb-06	9.777	-75.785	Rodriguez, Alberto	3.5	1.0%	11.0%	2.0%		0	12.7

Observation Number	Reef or Site Name	Location	Country	Date (DD-MMM-YY)	Latitude (decimal degrees)	Longitude (decimal degrees)	Primary Contributor	Depth (m)	Cover bleached (%)	Colonies bleached (%)	Cover dead (%)	Colonies dead (%)	Observed DHW (°C-weeks)	Maximum DHW (°C-weeks)
2262	Isla Mangle	San Bernardo Islands	Colombia - Mainland	12-Feb-06	9.779	-75.786	Rodriguez, Alberto	7.5	1.0%	8.8%	3.0%		0	12.7
2276	Chengue	Santa Marta-Tayrona Park Area	Colombia - Mainland	18-Oct-06	11.326	-74.128	Rodriguez, Alberto	10.5	1.0%	1.2%	0.0%	0.0%	0.55	6.2
2277	Chengue	Santa Marta-Tayrona Park Area	Colombia - Mainland	18-Oct-06	11.326	-74.128	Rodriguez, Alberto	4.5	1.0%	2.3%	0.0%	0.0%	0.55	6.2
2278	Chengue	Santa Marta-Tayrona Park Area	Colombia - Mainland	25-Oct-06	11.330	-74.129	Rodriguez, Alberto	10.5	1.0%	0.9%	0.0%	0.0%	0.55	6.2
2279	Chengue	Santa Marta-Tayrona Park Area	Colombia - Mainland	27-Oct-06	11.330	-74.129	Rodriguez, Alberto	4.5	1.0%	5.6%	0.0%	0.0%	0.55	6.2
975	Calle 16	Miramar, Habana	Cuba - North	3-May-05	23.297	-82.556	de la Guardia, Elena	10	20.8%	36.5%	0.0%	0.0%	0	1.05
976	Copacabana	Miramar, Habana	Cuba - North	9-May-05	23.142	-82.491	de la Guardia, Elena	10	30.4%	49.1%	0.0%	0.0%	0	1.05
977	Puntilla	Miramar, Habana	Cuba - North	10-May-05	23.163	-82.526	de la Guardia, Elena	10	36.1%	53.1%	0.1%	0.6%	0	1.05
981	Rincon de Guanabo	Guanabo, Habana	Cuba - North	24-Jun-05	23.250	-82.217	de la Guardia, Elena	3	0.5%	2.1%	0.0%	0.0%	0	1
988	Calle 16	Miramar, Habana	Cuba - North	14-Sep-05	23.297	-82.556	de la Guardia, Elena	10	43.8%	63.6%	0.1%	0.6%	1.05	1.05
989	Rincon de Guanabo	Guanabo, Habana	Cuba - North	17-Sep-05	23.250	-82.217	de la Guardia, Elena	3	25.3%	41.2%	0.0%	0.0%	1	1
2112	Rincón de Guanabo (East of Hav	Rincón de Guanabo (East of Hav	Cuba - North	18-Sep-05	23.177	-82.098	ReefBase	2	62.5%				1	1
990	Puntilla	Miramar, Habana	Cuba - North	20-Sep-05	23.163	-82.526	de la Guardia, Elena	10	50.2%	72.4%	0.1%	0.0%	1.05	1.05
991	Copacabana	Miramar, Habana	Cuba - North	26-Sep-05	23.142	-82.491	de la Guardia, Elena	10	56.6%	70.7%	0.0%	0.0%	1.05	1.05
2117	Facing the Instituto de Oceanolo	Facing the Instituto de Oceanolo	Cuba - North	5-Oct-05	23.096	-82.472	ReefBase	12.5	70.0%				1.05	1.05
2121	Cayo Coco (Central Northern Cu	Cayo Coco (Central Northern Cu	Cuba - North	10-Oct-05	22.561	-78.463	ReefBase	7.5	50.0%				6.7	6.7
2122	Cayo Guillermo (central norther	Cayo Guillermo (central norther	Cuba - North	12-Oct-05	22.633	-78.646	ReefBase	8.5	60.0%				6.7	6.7
2123	Reparto Flores, Havana City	Reparto Flores, Havana City	Cuba - North	13-Oct-05	23.101	-82.465	ReefBase	30	40.0%				1.05	1.05
999	Calle 16	Miramar, Habana	Cuba - North	5-Jan-06	23.297	-82.556	de la Guardia, Elena	10	27.5%	33.7%	1.0%	0.5%	0	1.05
978	Boya 7	Punta Frances, Isla de la Juventu	Cuba - South	25-May-05	21.701	-83.198	de la Guardia, Elena	15	18.4%	24.2%	0.0%	0.0%	0	2.75
979	Boya RC	Punta Frances, Isla de la Juventu	Cuba - South	26-May-05	21.728	-83.189	de la Guardia, Elena	10	29.7%	33.8%	0.0%	0.0%	0	2.75
980	Boya 36	Punta Frances, Isla de la Juventu	Cuba - South	27-May-05	21.727	-83.348	de la Guardia, Elena	12	42.1%	50.0%	0.0%	0.0%	0	2.75
982	Ballenatos	Canarreos	Cuba - South	27-Jul-05	21.608	-81.789	de la Guardia, Elena	3	4.1%	9.6%	0.0%	0.0%	0	4.6
983	Estopa	Canarreos	Cuba - South	28-Jul-05	21.751	-82.007	de la Guardia, Elena	8	5.5%	18.1%	0.0%	0.0%	0	4.6
984	Rosario	Canarreos	Cuba - South	29-Jul-05	21.597	-82.067	de la Guardia, Elena	10	0.3%	1.4%	0.0%	0.0%	0	4.6
2100	Cayo Largo (SW Cuba)	Cayo Largo (SW Cuba)	Cuba - South	15-Aug-05	21.583	-81.633	ReefBase	5	35.0%				0.6	4.6
2103	East of Bahía de Cochinos	East of Bahía de Cochinos	Cuba - South	1-Sep-05	22.166	-81.138	ReefBase	14	37.5%				1.7	3.4
2105	Cayo Anclitas (Jardines de la Re	Cayo Anclitas (Jardines de la Re	Cuba - South	6-Sep-05	20.817	-78.972	ReefBase	6.5	10.0%				2.7	6.65
985	Punta Perdiz	Bahía de Cochinos	Cuba - South	7-Sep-05	22.213	-81.209	de la Guardia, Elena	5	42.9%	60.9%	0.0%	0.0%	1.7	3.4
986	Punta Perdiz	Bahía de Cochinos	Cuba - South	7-Sep-05	22.213	-81.209	de la Guardia, Elena	10	29.8%	47.8%	0.1%	0.0%	1.7	3.4
987	Punta Perdiz	Bahía de Cochinos	Cuba - South	7-Sep-05	22.213	-81.209	de la Guardia, Elena	20	24.4%	19.3%	0.3%	0.0%	1.7	3.4
2107	María la Gorda (West end of Cu	María la Gorda (West end of Cu	Cuba - South	7-Sep-05	21.799	-84.522	ReefBase	21.5	8.0%				1.5	2.5
2108	Punta Perdiz (Bahía de Cochino	Punta Perdiz (Bahía de Cochino	Cuba - South	7-Sep-05	22.114	-81.118	ReefBase	15	50.0%				1.7	3.4
2125	Cayo Anclitas (Jardines de la Re	Cayo Anclitas (Jardines de la Re	Cuba - South	17-Oct-05	20.818	-78.972	ReefBase	22.5	63.0%				6.65	6.65
2126	Punta Francés, Isla de la Juventu	Punta Francés, Isla de la Juventu	Cuba - South	1-Nov-05	21.610	-83.102	ReefBase	5	12.5%				2.75	2.75
2127	María la Gorda (West end of Cu	María la Gorda (West end of Cu	Cuba - South	9-Nov-05	21.799	-84.522	ReefBase	12.5	40.0%				2.5	2.5
992	Boya 7	Punta Frances, Isla de la Juventu	Cuba - South	25-Nov-05	21.701	-83.198	de la Guardia, Elena	15	17.4%	29.8%	0.2%	0.4%	1.2	2.75
993	Boya 36	Punta Frances, Isla de la Juventu	Cuba - South	26-Nov-05	21.727	-83.348	de la Guardia, Elena	12	14.6%	29.6%	0.0%	0.1%	1.2	2.75
994	Boya RC	Punta Frances, Isla de la Juventu	Cuba - South	27-Nov-05	21.728	-83.189	de la Guardia, Elena	12	18.1%	34.4%	0.0%	0.0%	1.2	2.75
995	Cresta de la pasa	Punta Frances, Isla de la Juventu	Cuba - South	28-Nov-05	21.656	-83.419	de la Guardia, Elena	3	10.6%	24.7%	0.0%	0.0%	1.2	2.75
996	Punta Perdiz	Bahía de Cochinos	Cuba - South	9-Dec-05	22.213	-81.209	de la Guardia, Elena	5	17.7%	26.5%	0.2%	0.5%	0.5	3.4
997	Punta Perdiz	Bahía de Cochinos	Cuba - South	9-Dec-05	22.213	-81.209	de la Guardia, Elena	10	19.9%	25.7%	0.5%	0.0%	0.5	3.4
998	Punta Perdiz	Bahía de Cochinos	Cuba - South	9-Dec-05	22.213	-81.209	de la Guardia, Elena	20	21.8%	33.9%	1.7%	0.6%	0.5	3.4
2609	Salisbury E	Grand Savane	Dominica	20-Oct-05	15.262	-61.264	Steiner, Sascha	3.5	96.0%	95.9%			14.5	15.6
2610	Salisbury W	Grand Savane	Dominica	20-Oct-05	15.262	-61.264	Steiner, Sascha	12.5	80.7%	71.9%			14.5	15.6
2611	Calibishi	Calibishi	Dominica	25-Oct-05	15.354	-61.205	Steiner, Sascha	3.5	94.2%	64.8%			15.1	15.6
2612	Batali	Batali	Dominica	27-Oct-05	15.266	-61.276	Steiner, Sascha	3.5	88.9%	76.6%			15.6	15.6
2613	Floral Gardens	Grand Savane	Dominica	27-Oct-05	15.265	-61.270	Steiner, Sascha	12.5	85.3%	65.9%			15.6	15.6
2614	Fond Cole	Fond Cole	Dominica	29-Oct-05	15.191	-61.234	Steiner, Sascha	3.5	78.2%	84.9%			15.6	15.6
2615	Rena's	Grand Savane	Dominica	1-Nov-05	15.263	-61.271	Steiner, Sascha	12.5	71.6%	68.8%			15.6	15.6
2616	Macoucherie	Macoucheri	Dominica	2-Nov-05	15.255	-61.261	Steiner, Sascha	3.5	61.8%	63.4%			15.6	15.6
2617	Rodneys Rock	Tarou	Dominica	2-Nov-05	15.225	-61.264	Steiner, Sascha	3.5	85.5%	88.7%			15.6	15.6
2618	Brain Reef	Grand Savane	Dominica	3-Nov-05	15.262	-61.271	Steiner, Sascha	12.5	82.0%	79.1%			15.6	15.6
2619	Bery's Dream	Mero	Dominica	7-Nov-05	15.251	-61.256	Steiner, Sascha	12.5	88.8%	83.9%			15	15.6
2620	Salisbury E	Grand Savane	Dominica	8-Nov-05	15.262	-61.264	Steiner, Sascha	3.5	93.0%	90.6%			15	15.6
2621	Salisbury W	Grand Savane	Dominica	8-Nov-05	15.262	-61.264	Steiner, Sascha	12.5	98.0%	81.3%			15	15.6
2622	Champagne E	Champagne	Dominica	12-Nov-05	15.144	-61.222	Steiner, Sascha	3.5	95.6%	86.4%			14.3	15.6
2623	Champagne W	Champagne	Dominica	12-Nov-05	15.144	-61.223	Steiner, Sascha	12.5	88.0%	80.6%			14.3	15.6

Observation Number	Reef or Site Name	Location	Country	Date (DD-MMM-YY)	Latitude (decimal degrees)	Longitude (decimal degrees)	Primary Contributor	Depth (m)	Cover bleached (%)	Colonies bleached (%)	Cover dead (%)	Colonies dead (%)	Observed DHW (°C-weeks)	Maximum DHW (°C-weeks)
2624	Cachacrou N	Scotts Head	Dominica	13-Nov-05	15.125	-61.221	Steiner, Sascha	12.5	87.8%	77.5%			13.7	15.6
2625	Cachacrou S	Scotts Head	Dominica	13-Nov-05	15.125	-61.221	Steiner, Sascha	3.5	66.8%	59.6%			13.7	15.6
2626	Colihaut	Colihaut	Dominica	18-Nov-05	15.285	-62.274	Steiner, Sascha	6	93.9%	85.5%			13.1	15.6
2096	Lauro Reef		Dominica	14-Jan-06	15.439	-61.446	Reef Check	9.5	2.0%	43.8%			1	15.6
2097	Rinas Hole		Dominica	14-Jan-06	15.442	-61.454	Reef Check	9.5	4.0%	17.5%			1	15.6
2098	Rose Garden		Dominica	21-Jan-06	15.591	-61.472	Reef Check	9.8	0.0%	0.0%			0	15.6
2099	Sunshine Reef		Dominica	21-Jan-06	15.582	-61.476	Reef Check	12	0.0%	0.0%			0	15.6
2627	Salisbury E	Grand Savane	Dominica	24-Oct-06	15.262	-61.264	Steiner, Sascha	3.5	12.0%	15.3%			4.2	15.6
2628	Champagne E	Champagne	Dominica	26-Oct-06	15.144	-61.222	Steiner, Sascha	3.5	35.0%	30.4%			4.2	15.6
2629	Salisbury W	Grand Savane	Dominica	27-Oct-06	15.262	-61.264	Steiner, Sascha	12.5	32.0%	32.5%			4.2	15.6
2630	Bery's Dream	Mero	Dominica	31-Oct-06	15.251	-61.256	Steiner, Sascha	12.5	37.0%	30.6%			4.2	15.6
2631	Batali	Batali	Dominica	1-Nov-06	15.266	-61.276	Steiner, Sascha	3.5	15.0%	20.7%			4.2	15.6
2632	Fond Cole	Fond Cole	Dominica	2-Nov-06	15.191	-61.234	Steiner, Sascha	3.5	37.0%	28.9%			4.2	15.6
2633	Rodneys Rock	Tarou	Dominica	3-Nov-06	15.225	-61.264	Steiner, Sascha	3.5	22.0%	18.8%			4.2	15.6
2634	Brain Reef	Grand Savane	Dominica	6-Nov-06	15.262	-61.271	Steiner, Sascha	12.5	56.0%	38.1%			4.2	15.6
2635	Cachacrou S	Scotts Head	Dominica	8-Nov-06	15.125	-61.221	Steiner, Sascha	3.5	21.0%	23.7%			4.2	15.6
2636	Rena's	Grand Savane	Dominica	11-Nov-06	15.263	-61.271	Steiner, Sascha	12.5	31.0%	27.6%			4.2	15.6
2637	Floral Gardens	Grand Savane	Dominica	13-Nov-06	15.265	-61.270	Steiner, Sascha	12.5	28.0%	31.1%			4.2	15.6
2638	Calibishi	Calibishi	Dominica	15-Nov-06	15.354	-61.205	Steiner, Sascha	3.5	19.0%	22.0%			4.2	15.6
2639	Cachacrou N	Scotts Head	Dominica	16-Nov-06	15.125	-61.221	Steiner, Sascha	12.5	39.0%	28.6%			4.2	15.6
2640	Champagne W	Champagne	Dominica	16-Nov-06	15.144	-61.223	Steiner, Sascha	12.5	18.0%	7.5%			4.2	15.6
2641	Colihaut	Colihaut	Dominica	18-Nov-06	15.285	-62.274	Steiner, Sascha	3.5	32.0%	30.0%			4.2	15.6
2642	Macoucherie	Macoucheri	Dominica	25-Nov-06	15.255	-61.261	Steiner, Sascha	3.5	30.0%	23.5%			4.2	15.6
2643	Salisbury W	Grand Savane	Dominica	25-Nov-06	15.262	-61.264	Steiner, Sascha	12.5	19.0%	18.2%			4.2	15.6
2056	Acuario (Catalina)	La Romana	Dominican Republic	14-Aug-05	18.398	-69.024	Reef Check	10	1.0%	3.0%			0	6.55
2057	Pared	Catalina	Dominican Republic	14-Aug-05	18.374	-69.006	Reef Check	5	3.0%	4.0%			0	6.55
2080	Plaza Helena	Las Terrenas	Dominican Republic	2-Oct-05	19.340	-69.480	Reef Check	6	40.0%	50.0%			4.1	5.65
2081	Plaza Ramon	Las Terrenas	Dominican Republic	2-Oct-05	19.340	-69.499	Reef Check	5	60.0%	65.0%			4.15	6.55
2082	Aquario	Bayahibe	Dominican Republic	8-Oct-05	18.267	-68.783	Reef Check	10	30.0%	75.0%			5.35	6.55
33	Reef flat Ilet Fajou	Grand Cul-de-Sac Marin	FWI - Guadeloupe	23-Sep-05	16.357	-61.587	Bouchon, Claude	1.5	34.0%	25.7%	0.6%	2.9%	9.25	15.4
34	Passe à Colas	Grand Cul-de-Sac Marin	FWI - Guadeloupe	23-Nov-05	16.360	-61.574	Bouchon, Claude	12	55.0%	47.9%	3.0%	4.2%	11.95	15.4
37	Pointe Gris-Gris	Port-Louis	FWI - Guadeloupe	7-Dec-05	16.398	-61.533	Bouchon, Claude	11	50.0%	44.8%	4.0%	6.9%	9.4	15.4
38	Pointe Barracuda	Ilet Pigeon	FWI - Guadeloupe	9-Dec-05	16.167	-61.791	Bouchon, Claude	10	49.0%	49.0%	5.0%	6.0%	9.35	15.6
39	Outer reef slope Ilet Fajou	Grand Cul-de-Sac Marin	FWI - Guadeloupe	15-Dec-05	16.361	-61.580	Bouchon, Claude	11	78.0%	86.5%	4.0%	5.8%	7.75	15.4
40	Passe à Colas	Grand Cul-de-Sac Marin	FWI - Guadeloupe	17-Apr-06	16.360	-61.574	Bouchon, Claude	12	22.0%	13.4%	51.8%	45.1%	0	15.4
41	Reef flat Ilet Fajou	Grand Cul-de-Sac Marin	FWI - Guadeloupe	17-Apr-06	16.357	-61.587	Bouchon, Claude	1.5	15.0%	4.4%	16.7%	0.0%	0	15.4
42	Pointe Barracuda	Ilet Pigeon	FWI - Guadeloupe	27-Apr-06	16.167	-61.791	Bouchon, Claude	10	10.0%	10.8%	25.8%	20.5%	0	15.6
45	Outer reef slope Ilet Fajou	Grand Cul-de-Sac Marin	FWI - Guadeloupe	18-May-06	16.361	-61.580	Bouchon, Claude	11	36.0%	26.7%	42.1%	73.3%	0	15.4
46	Pointe Gris-Gris	Port-Louis	FWI - Guadeloupe	27-May-06	16.398	-61.533	Bouchon, Claude	11	15.0%	10.1%	44.3%	42.0%	0	15.4
51	Passe à Colas	Grand Cul-de-Sac Marin	FWI - Guadeloupe	21-Nov-06	16.360	-61.574	Bouchon, Claude	12	12.0%	5.6%	51.8%	67.6%	3.95	15.4
54	Outer reef slope Ilet Fajou	Grand Cul-de-Sac Marin	FWI - Guadeloupe	12-Dec-06	16.361	-61.580	Bouchon, Claude	11	36.0%	21.4%	50.8%	85.7%	3.3	15.4
55	Pointe Gris-Gris	Port-Louis	FWI - Guadeloupe	19-Dec-06	16.398	-61.533	Bouchon, Claude	11	14.0%	12.7%	39.7%	38.0%	1.9	15.4
56	Pointe Barracuda	Ilet Pigeon	FWI - Guadeloupe	20-Dec-06	16.167	-61.791	Bouchon, Claude	10	9.0%	9.6%	39.7%	37.0%	2.25	15.6
57	Reef flat Ilet Fajou	Grand Cul-de-Sac Marin	FWI - Guadeloupe	31-Dec-06	16.357	-61.587	Bouchon, Claude	1.5	21.0%	4.9%	27.8%	0.0%	0	15.4
1729	Ilet à Rats	Robert	FWI - Martinique	25-Jun-05	14.680	-60.090	Marechal, Jean-Philippe	5	2.0%	1.3%			1.15	11.75
1730	Fond Boucher	Case Pilote	FWI - Martinique	9-Jul-05	14.390	-61.090	Marechal, Jean-Philippe	9	0.0%	0.0%			1.15	11.75
1731	Jardin Tropical	Sainte Luce	FWI - Martinique	10-Jul-05	14.270	-60.550	Marechal, Jean-Philippe	12	0.0%	0.0%			1.15	11.75
1732	Pointe Borgnesse	Sainte Luce	FWI - Martinique	11-Jul-05	14.260	-60.540	Marechal, Jean-Philippe	10	0.0%	0.0%			1.15	11.75
1733	Pointe Borgnesse	Sainte Luce	FWI - Martinique	28-Oct-05	14.464	-60.909	Marechal, Jean-Philippe	10	76.4%	76.4%	16.0%		11.25	11.75
1734	Jardin Tropical	Sainte Luce	FWI - Martinique	8-Nov-05	14.452	-60.930	Marechal, Jean-Philippe	12	74.0%	72.6%	20.0%		10.7	11.75
1735	Jardin Tropical	Sainte Luce	FWI - Martinique	27-Nov-05	14.270	-60.550	Marechal, Jean-Philippe	12	38.6%	38.6%	18.1%		7.55	11.75
35	Fond Boucher	Case Pilote	FWI - Martinique	3-Dec-05	14.658	-61.158	Bouchon, Claude	10	37.0%	31.5%	10.0%	9.4%	7.55	11.75
1736	Fond Boucher	Case Pilote	FWI - Martinique	3-Dec-05	14.390	-61.090	Marechal, Jean-Philippe	9	32.2%	32.2%			7.55	11.75
36	Pointe Borgnèse	Sainte-Luce	FWI - Martinique	4-Dec-05	14.447	-60.905	Bouchon, Claude	12	61.0%	41.4%	1.0%	1.7%	7.55	11.75
1737	Pointe Borgnesse	Sainte Luce	FWI - Martinique	4-Dec-05	14.260	-60.540	Marechal, Jean-Philippe	10	53.3%	53.3%			7.55	11.75
1738	Ilet à Rats	Robert	FWI - Martinique	18-Dec-05	14.680	-60.090	Marechal, Jean-Philippe	5	15.3%	15.3%	21.9%		4.65	11.75
1020	MAR1-1	Pte. Borgnesse	FWI - Martinique	13-Jan-06	14.447	-60.905	Ginsburg, Robert	12		59.2%	11.3%	4.2%	0	11.75
1021	MAR1-2	Corpo de Garde	FWI - Martinique	13-Jan-06	14.456	-60.939	Ginsburg, Robert	11		48.1%	2.4%	0.0%	0	11.75

Observation Number	Reef or Site Name	Location	Country	Date (DD-MMM-YY)	Latitude (decimal degrees)	Longitude (decimal degrees)	Primary Contributor	Depth (m)	Cover bleached (%)	Colonies bleached (%)	Cover dead (%)	Colonies dead (%)	Observed DHW (°C-weeks)	Maximum DHW (°C-weeks)
1022	MAR2-1	Fond Boucher	FWI - Martinique	14-Jan-06	14.658	-61.158	Ginsburg, Robert	6.5		22.5%	19.8%	6.7%	0	11.75
1023	MAR2-2	La Citadelle	FWI - Martinique	14-Jan-06	14.810	-61.230	Ginsburg, Robert	12.5		17.1%	1.2%	0.0%	0	11.75
1024	MAR2-3	Trou Bleu	FWI - Martinique	14-Jan-06	14.767	-61.199	Ginsburg, Robert	13.5		21.8%	13.4%	5.7%	0	11.75
1025	MAR3-1	Burgos	FWI - Martinique	15-Jan-06	14.493	-61.091	Ginsburg, Robert	12.5		43.6%	5.8%	1.8%	0	11.75
1026	MAR3-2	Jardin de Solomon	FWI - Martinique	15-Jan-06	14.506	-61.097	Ginsburg, Robert	13.5		40.0%	11.6%	7.1%	0	11.75
1709	MAR3-1	Burgos	FWI - Martinique	31-May-06	14.492	-61.089	Lang, Judith	11.9		11.8%			0	11.75
1710	MAR3-2	Jardin de Salomon	FWI - Martinique	31-May-06	14.506	-61.097	Lang, Judith	9.2		12.3%			0	11.75
1711	MAR2-1	Fond Boucher	FWI - Martinique	1-Jun-06	14.658	-61.158	Lang, Judith	11.4		10.7%			0	11.75
1712	MAR1-1	Pte. Borgnesse	FWI - Martinique	2-Jun-06	14.447	-60.905	Lang, Judith	10.8		21.4%			0	11.75
1713	MAR1-2	Corps de Garde	FWI - Martinique	2-Jun-06	14.456	-60.939	Lang, Judith	11.8		22.6%			0	11.75
1739	Fond Boucher	Case Pilote	FWI - Martinique	13-Jul-06	14.390	-61.090	Marechal, Jean-Philippe	9	0.0%	0.0%	48.8%		0	11.75
47	Fond Boucher	Case Pilote	FWI - Martinique	14-Jul-06	14.658	-61.158	Bouchon, Claude	10	5.0%	4.2%	21.1%	88.5%	0	11.75
48	Pointe Borgnèse	Sainte-Luce	FWI - Martinique	15-Jul-06	14.447	-60.905	Bouchon, Claude	12	26.0%	27.8%	52.9%	61.1%	0	11.75
1740	Pointe Borgnesse	Sainte Luce	FWI - Martinique	15-Jul-06	14.260	-60.540	Marechal, Jean-Philippe	10	0.0%	0.0%	30.9%		0	11.75
1741	Ilet à Rats	Robert	FWI - Martinique	20-Jul-06	14.680	-60.090	Marechal, Jean-Philippe	5	0.0%	0.0%	4.3%		0	11.75
1742	Jardin Tropical	Sainte Luce	FWI - Martinique	24-Jul-06	14.270	-60.550	Marechal, Jean-Philippe	12	0.0%	0.0%	19.3%		0	11.75
1743	Jardin Tropical	Sainte Luce	FWI - Martinique	18-Nov-06	14.270	-60.550	Marechal, Jean-Philippe	12	0.0%	0.0%			1.6	11.75
1744	Ilet à Rats	Robert	FWI - Martinique	26-Nov-06	14.680	-60.090	Marechal, Jean-Philippe	5	0.0%	0.0%			1.6	11.75
1745	Fond Boucher	Case Pilote	FWI - Martinique	1-Dec-06	14.390	-61.090	Marechal, Jean-Philippe	9	0.0%	0.0%			1.6	11.75
52	Fond Boucher	Case Pilote	FWI - Martinique	2-Dec-06	14.658	-61.158	Bouchon, Claude	10	0.0%	0.0%	21.1%	77.5%	1.6	11.75
53	Pointe Borgnèse	Sainte-Luce	FWI - Martinique	3-Dec-06	14.447	-60.905	Bouchon, Claude	12	48.0%	34.2%	52.9%	52.6%	1.6	11.75
1746	Pointe Borgnesse	Sainte Luce	FWI - Martinique	3-Dec-06	14.260	-60.540	Marechal, Jean-Philippe	10	0.0%	0.0%			1.6	11.75
31	Ilet Coco	Ilet Coco	FWI - Saint-Barthélemy	15-Aug-05	17.874	-62.814	Bouchon, Claude	12	46.0%	47.3%	0.0%	0.0%	2.35	11.65
32	Baleine du Pain de Sucre	Pain de Sucre	FWI - Saint-Barthélemy	17-Aug-05	17.899	-62.877	Bouchon, Claude	11	61.0%	58.2%	0.0%	0.0%	2.35	11.65
43	Baleine du Pain de Sucre	Pain de Sucre	FWI - Saint-Barthélemy	5-May-06	17.899	-62.877	Bouchon, Claude	11	23.0%	15.4%	30.1%	17.3%	0	11.65
44	Ilet Coco	Ilet Coco	FWI - Saint-Barthélemy	7-May-06	17.874	-62.814	Bouchon, Claude	12	11.0%	8.5%	21.7%	17.0%	0	11.65
49	Ilet Coco	Ilet Coco	FWI - Saint-Barthélemy	8-Nov-06	17.874	-62.814	Bouchon, Claude	12	18.0%	9.2%	28.2%	26.4%	2.75	11.65
50	Baleine du Pain de Sucre	Pain de Sucre	FWI - Saint-Barthélemy	9-Nov-06	17.899	-62.877	Bouchon, Claude	11	22.0%	17.8%	30.1%	35.6%	2.75	11.65
2816	Flamingo Bay	West Coast	Grenada	4-Oct-05	12.092	-61.759	Weil, Ernesto	11.5		49.1%			9.85	13.55
882	M1	Discovery Bay	Jamaica	10-Aug-05	18.469	-77.408	Crabbe, James	12	5.0%	6.7%	0.0%	0.0%	0	4.9
883	Dancing Ladies	Discovery Bay	Jamaica	11-Aug-05	18.474	-77.393	Crabbe, James	10	5.0%	5.0%	0.0%	0.0%	0	4.9
884	Rio Bueno	Discovery Bay	Jamaica	11-Aug-05	18.477	-77.451	Crabbe, James	8	5.0%	3.3%	0.0%	0.0%	0	4.9
885	Columbus Park	Discovery Bay	Jamaica	12-Aug-05	18.458	-77.406	Crabbe, James	8	20.0%	20.0%	0.0%	0.0%	0.5	4.9
886	Pear Tree Bottom	Discovery Bay	Jamaica	12-Aug-05	18.463	-77.391	Crabbe, James	10	5.0%	4.0%	0.0%	2.0%	0.5	4.9
887	Dairy Bull	Discovery Bay	Jamaica	13-Aug-05	18.468	-77.394	Crabbe, James	8	20.0%	20.0%	0.0%	8.9%	0.5	4.9
2061	Bloody Bay	Westmoreland	Jamaica	27-Aug-05	18.361	-78.343	Reef Check	10	0.3%	0.0%			1.15	6.6
2062	Bloody Bay	Westmoreland	Jamaica	27-Aug-05	18.361	-78.343	Reef Check	4	0.0%	0.0%			1.15	6.6
2063	El Punto Negrilo	Negril	Jamaica	28-Aug-05	18.254	-78.367	Reef Check	4	0.0%	0.0%			1.15	6.6
2064	El Punto Negrilo	Negril	Jamaica	28-Aug-05	18.254	-78.367	Reef Check	10	0.0%	0.0%			1.15	6.6
2027	East Rio Bueno	St Ann	Jamaica	9-Sep-05	18.479	-77.450	Quinn, Norman	10	0.0%	90.0%	0.0%	0.0%	1	4.9
2028	Caricomp Site	Discovery Bay	Jamaica	27-Sep-05	18.473	-77.414	Quinn, Norman	30	92.0%	90.0%	0.0%	0.0%	3.9	4.9
2029	Dairy Bull	Discovery Bay	Jamaica	29-Sep-05	18.468	-77.389	Quinn, Norman	10	95.0%	95.0%	0.0%	0.0%	4.4	4.9
2030	East Rio Bueno	St Ann	Jamaica	30-Sep-05	18.479	-77.450	Quinn, Norman	10	78.0%	70.0%	0.0%	0.0%	4.4	4.9
2083	Drunkenman's Cay	Port Royal	Jamaica	8-Nov-05	17.902	-76.846	Reef Check	7	82.5%	90.0%			4.25	4.25
2084	Drunkenman's Cay	Port Royal	Jamaica	8-Nov-05	17.902	-76.846	Reef Check	4	87.5%	60.0%			4.25	4.25
2086	Lime Cay	Port Royal	Jamaica	17-Nov-05	17.942	-76.819	Reef Check	5	65.0%	85.0%			4.25	4.25
2087	Lime Cay	Port Royal	Jamaica	17-Nov-05	17.942	-76.819	Reef Check	7	77.5%	68.8%			4.25	4.25
2035	Negril Reef	Negril	Jamaica	20-Nov-05	18.314	-78.356	Quinn, Norman	10	42.0%	42.0%	0.0%	0.0%	5.45	6.6
2036	M1	Discovery Bay	Jamaica	1-Dec-05	18.472	-77.409	Quinn, Norman	10	99.0%	99.0%	1.0%	1.0%	4.4	4.9
2037	Dairy Bull	Discovery Bay	Jamaica	11-Dec-05	18.468	-77.389	Quinn, Norman	10	100.0%	100.0%	4.0%	20.0%	2.8	4.9
2038	Pear Tree Bottom	Discovery Bay	Jamaica	12-Dec-05	18.465	-77.357	Quinn, Norman	10	98.0%	98.0%	1.0%	1.0%	2.25	4.9
2039	Chalet Caribe	Montrgo Bay	Jamaica	16-Dec-05	18.454	-77.972	Quinn, Norman	10	100.0%	100.0%	2.0%	2.0%	1.55	3.85
2040	East Rio Bueno	Rio Bueno Bay	Jamaica	28-Dec-05	18.479	-77.450	Quinn, Norman	10	100.0%	100.0%	10.0%	10.0%	0	4.9
2041	Pear Tree Bottom	Discovery Bay	Jamaica	30-Dec-05	18.465	-77.357	Quinn, Norman	10	98.0%	98.0%	6.0%	6.0%	0	4.9
2042	Lipman's Ledge	Discovery Bay	Jamaica	31-Dec-05	18.471	-77.383	Quinn, Norman	10	98.0%	98.0%	0.0%		0	4.9
2043	Dancing Lady	Discovery Bay	Jamaica	1-Jan-06	18.473	-77.412	Quinn, Norman	10	96.0%	96.0%	1.0%	1.0%	0	4.9
2044	M1	Discovery Bay	Jamaica	3-Jan-06	18.472	-77.409	Quinn, Norman	10	95.0%	95.0%	5.0%	10.0%	0	4.9
2047	M1	Discovery Bay	Jamaica	27-Feb-06	18.472	-77.409	Quinn, Norman	10	90.0%	90.0%	10.0%	10.0%	0	4.9

Observation Number	Reef or Site Name	Location	Country	Date (DD-MMM-YY)	Latitude (decimal degrees)	Longitude (decimal degrees)	Primary Contributor	Depth (m)	Cover bleached (%)	Colonies bleached (%)	Cover dead (%)	Colonies dead (%)	Observed DHW (°C-weeks)	Maximum DHW (°C-weeks)
2048	Negril Reef	Negril	Jamaica	7-Mar-06	18.316	-78.353	Quinn, Norman	10	10.0%	10.0%	5.0%	20.0%	0	6.6
2049	Dairy Bull	Discovery Bay	Jamaica	14-Mar-06	18.468	-77.389	Quinn, Norman	10	0.0%	0.0%			0	4.9
112	LL10	Mahahual	Mexico	9-May-05	18.707	-87.706	Cameron, Andy	9.75		36.4%			0	2.15
1768	LL10	Las Llamaradas	Mexico	9-May-05	18.704	-87.704	Ponce-Taylor, Daniel	9.75		50.0%			0	0.5
113	DB05	Mahahual	Mexico	10-May-05	18.724	-87.698	Cameron, Andy	5.5		30.8%			0	2.15
114	DB05	Mahahual	Mexico	10-May-05	18.724	-87.698	Cameron, Andy	4.95		18.8%			0	2.15
115	LL05	Mahahual	Mexico	10-May-05	18.707	-87.707	Cameron, Andy	5.65		16.7%			0	2.15
116	LL10	Mahahual	Mexico	10-May-05	18.707	-87.706	Cameron, Andy	9.1		19.2%			0	2.15
117	LPC10	Mahahual	Mexico	10-May-05	18.659	-87.717	Cameron, Andy	10.05		5.9%			0	0.5
118	PDC20	Mahahual	Mexico	10-May-05	18.667	-87.714	Cameron, Andy	16.05		9.3%			0	0.5
119	PJ10	Pez Maya, Reserva de la Biosfer	Mexico	10-May-05	20.017	-87.462	Cameron, Andy	8.8		40.0%			0	0
120	PX10	Pez Maya, Reserva de la Biosfer	Mexico	10-May-05	19.934	-87.434	Cameron, Andy	10.25		50.0%			0	0
1769	DB05	Dolphin Bay	Mexico	10-May-05	18.721	-87.692	Ponce-Taylor, Daniel	5.5		40.0%			0	2.15
1770	DB05	Dolphin Bay	Mexico	10-May-05	18.721	-87.692	Ponce-Taylor, Daniel	4.95		37.5%			0	2.15
1771	LL05	Las Llamaradas	Mexico	10-May-05	18.704	-87.704	Ponce-Taylor, Daniel	5.65		20.0%			0	0.5
1772	LL10	Las Llamaradas	Mexico	10-May-05	18.704	-87.704	Ponce-Taylor, Daniel	9.1		35.7%			0	0.5
1773	LPC10	Los Preciones Cañones	Mexico	10-May-05	18.655	-87.710	Ponce-Taylor, Daniel	10.05		16.7%			0	0.5
1774	PDC20	Pirates Del Caribe	Mexico	10-May-05	18.660	-87.709	Ponce-Taylor, Daniel	16.05		23.5%			0	0.5
121	BUC10	Mahahual	Mexico	11-May-05	18.687	-87.711	Cameron, Andy	11.9		31.8%			0	0.5
122	BUC10	Mahahual	Mexico	11-May-05	18.687	-87.711	Cameron, Andy	11.6		23.8%			0	0.5
123	DB10	Mahahual	Mexico	11-May-05	18.724	-87.697	Cameron, Andy	7.75		11.8%			0	2.15
124	LL05	Mahahual	Mexico	11-May-05	18.707	-87.707	Cameron, Andy	5.1		33.3%			0	2.15
125	RB10	Mahahual	Mexico	11-May-05	18.681	-87.713	Cameron, Andy	9.9		26.7%			0	0.5
126	SMDR10	Pez Maya, Reserva de la Biosfer	Mexico	11-May-05	19.967	-87.453	Cameron, Andy	8.8		12.5%			0	0
127	SMDR20	Pez Maya, Reserva de la Biosfer	Mexico	11-May-05	19.968	-87.452	Cameron, Andy	17.85		66.7%			0	0
1775	BUC10	Bucaneros	Mexico	11-May-05	18.685	-87.706	Ponce-Taylor, Daniel	11.9		42.9%			0	0.5
1776	BUC10	Bucaneros	Mexico	11-May-05	18.685	-87.706	Ponce-Taylor, Daniel	11.6		36.4%			0	0.5
1777	DB10	Dolphin Bay	Mexico	11-May-05	18.721	-87.691	Ponce-Taylor, Daniel	7.75		25.0%			0	2.15
1778	LL05	Las Llamaradas	Mexico	11-May-05	18.704	-87.704	Ponce-Taylor, Daniel	5.1		50.0%			0	0.5
1779	RB10	Rio Bermejo	Mexico	11-May-05	18.675	-87.708	Ponce-Taylor, Daniel	9.9		36.4%			0	0.5
128	PP10	Pez Maya, Reserva de la Biosfer	Mexico	12-May-05	20.038	-87.466	Cameron, Andy	8.2		50.0%			0	0
129	PY20	Pez Maya, Reserva de la Biosfer	Mexico	12-May-05	19.973	-87.453	Cameron, Andy	19.65		25.0%			0	0
130	RB05	Mahahual	Mexico	12-May-05	18.681	-87.714	Cameron, Andy	5.8		0.0%			0	0.5
131	RB05	Mahahual	Mexico	12-May-05	18.681	-87.714	Cameron, Andy	5.8		33.3%			0	0.5
132	RB10	Mahahual	Mexico	12-May-05	18.681	-87.713	Cameron, Andy	8.95		60.0%			0	0.5
133	SMDR10	Pez Maya, Reserva de la Biosfer	Mexico	12-May-05	19.967	-87.453	Cameron, Andy	9.3		27.3%			0	0
134	SMDR10	Pez Maya, Reserva de la Biosfer	Mexico	12-May-05	19.967	-87.453	Cameron, Andy	9.6		10.0%			0	0
1780	RB05	Rio Bermejo	Mexico	12-May-05	18.675	-87.708	Ponce-Taylor, Daniel	5.8		0.0%			0	0.5
1781	RB05	Rio Bermejo	Mexico	12-May-05	18.675	-87.708	Ponce-Taylor, Daniel	5.8		33.3%			0	0.5
1782	RB10	Rio Bermejo	Mexico	12-May-05	18.675	-87.708	Ponce-Taylor, Daniel	8.95		64.3%			0	0.5
135	FV20	Mahahual	Mexico	13-May-05	18.742	-87.674	Cameron, Andy	20.85		0.0%			0	2.15
136	LE10	Mahahual	Mexico	13-May-05	18.720	-87.699	Cameron, Andy	9.15		25.0%			0	2.15
137	LE10	Mahahual	Mexico	13-May-05	18.720	-87.699	Cameron, Andy	9.1		28.0%			0	2.15
138	LE10	Mahahual	Mexico	13-May-05	18.720	-87.699	Cameron, Andy	10.8		47.8%			0	2.15
139	PL05	Pez Maya, Reserva de la Biosfer	Mexico	13-May-05	20.050	-87.470	Cameron, Andy	3.7		0.0%			0	0
140	PP10	Pez Maya, Reserva de la Biosfer	Mexico	13-May-05	20.038	-87.466	Cameron, Andy	8.8		57.9%			0	0
141	PP10	Pez Maya, Reserva de la Biosfer	Mexico	13-May-05	20.038	-87.466	Cameron, Andy	9.1		66.7%			0	0
1783	FV20	Faro Viejo	Mexico	13-May-05	18.738	-87.671	Ponce-Taylor, Daniel	20.85		0.0%			0	2.15
1784	LE10	Los Escalones	Mexico	13-May-05	18.718	-87.693	Ponce-Taylor, Daniel	9.15		38.1%			0	2.15
1785	LE10	Los Escalones	Mexico	13-May-05	18.718	-87.693	Ponce-Taylor, Daniel	9.1		43.8%			0	2.15
1786	LE10	Los Escalones	Mexico	13-May-05	18.718	-87.693	Ponce-Taylor, Daniel	10.8		58.8%			0	2.15
142	FV20	Mahahual	Mexico	14-May-05	18.742	-87.674	Cameron, Andy	19.2		0.0%			0	2.15
143	FV20	Mahahual	Mexico	14-May-05	18.742	-87.674	Cameron, Andy	18.3		0.0%			0	2.15
1787	FV20	Faro Viejo	Mexico	14-May-05	18.738	-87.671	Ponce-Taylor, Daniel	19.2		0.0%			0	2.15
1788	FV20	Faro Viejo	Mexico	14-May-05	18.738	-87.671	Ponce-Taylor, Daniel	18.3		0.0%			0	2.15
144	RB05	Mahahual	Mexico	15-May-05	18.681	-87.714	Cameron, Andy	6.4		0.0%			0	0.5
145	RB05	Mahahual	Mexico	15-May-05	18.681	-87.714	Cameron, Andy	5.8		28.6%			0	0.5
1789	RB05	Rio Bermejo	Mexico	15-May-05	18.675	-87.708	Ponce-Taylor, Daniel	6.4		0.0%			0	0.5

Observation Number	Reef or Site Name	Location	Country	Date (DD-MMM-YY)	Latitude (decimal degrees)	Longitude (decimal degrees)	Primary Contributor	Depth (m)	Cover bleached (%)	Colonies bleached (%)	Cover dead (%)	Colonies dead (%)	Observed DHW (°C-weeks)	Maximum DHW (°C-weeks)
1790	RB05	Rio Bermejo	Mexico	15-May-05	18.675	-87.708	Ponce-Taylor, Daniel	5.8		28.6%			0	0.5
146	PDC10	Mahahual	Mexico	16-May-05	18.667	-87.716	Cameron, Andy	10.7		11.5%			0	0.5
147	PDC10	Mahahual	Mexico	16-May-05	18.667	-87.716	Cameron, Andy	10.25		41.7%			0	0.5
148	PSJ05	Pez Maya, Reserva de la Biosfer	Mexico	16-May-05	19.883	-87.426	Cameron, Andy	2.3		8.3%			0	0
149	RB10	Mahahual	Mexico	16-May-05	18.681	-87.713	Cameron, Andy	9.1		0.0%			0	0.5
150	RB10	Mahahual	Mexico	16-May-05	18.681	-87.713	Cameron, Andy	9.15		26.7%			0	0.5
1791	PDC10	Pirates Del Caribe	Mexico	16-May-05	18.660	-87.709	Ponce-Taylor, Daniel	10.7		27.3%			0	0.5
1792	PDC10	Pirates Del Caribe	Mexico	16-May-05	18.660	-87.709	Ponce-Taylor, Daniel	10.25		62.5%			0	0.5
1793	RB10	Rio Bermejo	Mexico	16-May-05	18.675	-87.708	Ponce-Taylor, Daniel	9.1		0.0%			0	0.5
1794	RB10	Rio Bermejo	Mexico	16-May-05	18.675	-87.708	Ponce-Taylor, Daniel	9.15		40.0%			0	0.5
151	DB05	Mahahual	Mexico	17-May-05	18.724	-87.698	Cameron, Andy	4.45		20.0%			0	2.15
152	DB05	Mahahual	Mexico	17-May-05	18.724	-87.698	Cameron, Andy	5.3		0.0%			0	2.15
153	DB05	Mahahual	Mexico	17-May-05	18.724	-87.698	Cameron, Andy	5.3		10.0%			0	2.15
154	LL20	Mahahual	Mexico	17-May-05	18.707	-87.704	Cameron, Andy	14.3		73.9%			0	2.15
155	LL20	Mahahual	Mexico	17-May-05	18.707	-87.704	Cameron, Andy	13.75		42.1%			0	2.15
156	PSJ10	Pez Maya, Reserva de la Biosfer	Mexico	17-May-05	19.883	-87.418	Cameron, Andy	9.1		75.0%			0	0
157	PSJ10	Pez Maya, Reserva de la Biosfer	Mexico	17-May-05	19.883	-87.418	Cameron, Andy	9.1		15.4%			0	0
1795	DB05	Dolphin Bay	Mexico	17-May-05	18.721	-87.692	Ponce-Taylor, Daniel	4.45		30.0%			0	2.15
1796	DB05	Dolphin Bay	Mexico	17-May-05	18.721	-87.692	Ponce-Taylor, Daniel	5.3		0.0%			0	2.15
1797	DB05	Dolphin Bay	Mexico	17-May-05	18.721	-87.692	Ponce-Taylor, Daniel	5.3		20.0%			0	2.15
1798	LL20	Las Llamaradas	Mexico	17-May-05	18.704	-87.702	Ponce-Taylor, Daniel	14.3		78.9%			0	0.5
1799	LL20	Las Llamaradas	Mexico	17-May-05	18.704	-87.702	Ponce-Taylor, Daniel	13.75		58.3%			0	0.5
158	PC10	Pez Maya, Reserva de la Biosfer	Mexico	18-May-05	20.099	-87.462	Cameron, Andy	9.1		3.8%			0	0
159	PC10	Pez Maya, Reserva de la Biosfer	Mexico	18-May-05	20.099	-87.462	Cameron, Andy	7.45		50.0%			0	0
160	PC10	Pez Maya, Reserva de la Biosfer	Mexico	18-May-05	20.099	-87.462	Cameron, Andy	7.6		45.5%			0	0
161	PC10	Pez Maya, Reserva de la Biosfer	Mexico	18-May-05	20.099	-87.462	Cameron, Andy	8.2		0.0%			0	0
162	PDC05	Mahahual	Mexico	18-May-05	18.667	-87.717	Cameron, Andy	5.8		0.0%			0	0.5
163	PDC05	Mahahual	Mexico	18-May-05	18.667	-87.717	Cameron, Andy	5.8		100.0%			0	0.5
164	PDC10	Mahahual	Mexico	18-May-05	18.667	-87.716	Cameron, Andy	10.5		15.8%			0	0.5
165	PDC10	Mahahual	Mexico	18-May-05	18.667	-87.716	Cameron, Andy	10.15		35.7%			0	0.5
166	PDC10	Mahahual	Mexico	18-May-05	18.667	-87.716	Cameron, Andy	11.15		20.0%			0	0.5
167	PJ05	Pez Maya, Reserva de la Biosfer	Mexico	18-May-05	20.015	-87.465	Cameron, Andy	4.6		44.4%			0	0
168	PJ05	Pez Maya, Reserva de la Biosfer	Mexico	18-May-05	20.015	-87.465	Cameron, Andy	4.6		37.5%			0	0
169	PJ05	Pez Maya, Reserva de la Biosfer	Mexico	18-May-05	20.015	-87.465	Cameron, Andy	5.35		37.5%			0	0
170	PJ05	Pez Maya, Reserva de la Biosfer	Mexico	18-May-05	20.015	-87.465	Cameron, Andy	4.9		25.0%			0	0
1800	PDC05	Pirates Del Caribe	Mexico	18-May-05	18.660	-87.717	Ponce-Taylor, Daniel	5.8		0.0%			0	0.5
1801	PDC05	Pirates Del Caribe	Mexico	18-May-05	18.660	-87.717	Ponce-Taylor, Daniel	5.8		100.0%			0	0.5
1802	PDC10	Pirates Del Caribe	Mexico	18-May-05	18.660	-87.709	Ponce-Taylor, Daniel	10.5		27.3%			0	0.5
1803	PDC10	Pirates Del Caribe	Mexico	18-May-05	18.660	-87.709	Ponce-Taylor, Daniel	10.15		50.0%			0	0.5
1804	PDC10	Pirates Del Caribe	Mexico	18-May-05	18.660	-87.709	Ponce-Taylor, Daniel	11.15		40.0%			0	0.5
171	LE05	Mahahual	Mexico	19-May-05	18.720	-87.701	Cameron, Andy	5		0.0%			0	2.15
172	LE10	Mahahual	Mexico	19-May-05	18.720	-87.699	Cameron, Andy	10.55		12.5%			0	2.15
173	LE10	Mahahual	Mexico	19-May-05	18.720	-87.699	Cameron, Andy	9.85		13.8%			0	2.15
174	LL20	Mahahual	Mexico	19-May-05	18.707	-87.704	Cameron, Andy	14.75		10.3%			0	2.15
175	LL20	Mahahual	Mexico	19-May-05	18.707	-87.704	Cameron, Andy	15.5		10.0%			0	2.15
176	PJ10	Pez Maya, Reserva de la Biosfer	Mexico	19-May-05	20.017	-87.462	Cameron, Andy	13.7		50.0%			0	0
177	PJ10	Pez Maya, Reserva de la Biosfer	Mexico	19-May-05	20.017	-87.462	Cameron, Andy	8.05		29.4%			0	0
178	PJ10	Pez Maya, Reserva de la Biosfer	Mexico	19-May-05	20.017	-87.462	Cameron, Andy	10.1		18.2%			0	0
179	PSJ10	Pez Maya, Reserva de la Biosfer	Mexico	19-May-05	19.883	-87.418	Cameron, Andy	9.1		69.2%			0	0
180	PSJ20	Pez Maya, Reserva de la Biosfer	Mexico	19-May-05	19.880	-87.412	Cameron, Andy	20.25		0.0%			0	0
1805	LE05	Los Escalones	Mexico	19-May-05	18.718	-87.701	Ponce-Taylor, Daniel	5		0.0%			0	2.15
1806	LE10	Los Escalones	Mexico	19-May-05	18.718	-87.693	Ponce-Taylor, Daniel	10.55		22.2%			0	2.15
1807	LE10	Los Escalones	Mexico	19-May-05	18.718	-87.693	Ponce-Taylor, Daniel	9.85		28.6%			0	2.15
1808	LL20	Las Llamaradas	Mexico	19-May-05	18.704	-87.702	Ponce-Taylor, Daniel	14.75		27.3%			0	0.5
1809	LL20	Las Llamaradas	Mexico	19-May-05	18.704	-87.702	Ponce-Taylor, Daniel	15.5		22.2%			0	0.5
181	DB10	Mahahual	Mexico	20-May-05	18.724	-87.697	Cameron, Andy	8.5		30.8%			0	2.15
182	DB10	Mahahual	Mexico	20-May-05	18.724	-87.697	Cameron, Andy	8.8		20.0%			0	2.15
183	PDC20	Mahahual	Mexico	20-May-05	18.667	-87.714	Cameron, Andy	14.15		33.3%			0	0.5

Observation Number	Reef or Site Name	Location	Country	Date (DD-MMM-YY)	Latitude (decimal degrees)	Longitude (decimal degrees)	Primary Contributor	Depth (m)	Cover bleached (%)	Colonies bleached (%)	Cover dead (%)	Colonies dead (%)	Observed DHW (°C-weeks)	Maximum DHW (°C-weeks)
184	PJ10	Pez Maya, Reserva de la Biosfer	Mexico	20-May-05	20.017	-87.462	Cameron, Andy	11.9		40.0%			0	0
185	PL05	Pez Maya, Reserva de la Biosfer	Mexico	20-May-05	20.050	-87.470	Cameron, Andy	2.55		23.1%			0	0
186	PL05	Pez Maya, Reserva de la Biosfer	Mexico	20-May-05	20.050	-87.470	Cameron, Andy	3.2		15.4%			0	0
187	PL10	Pez Maya, Reserva de la Biosfer	Mexico	20-May-05	20.052	-87.466	Cameron, Andy	6.1		55.9%			0	0
188	PP10	Pez Maya, Reserva de la Biosfer	Mexico	20-May-05	20.038	-87.466	Cameron, Andy	7.45		33.3%			0	0
189	PP10	Pez Maya, Reserva de la Biosfer	Mexico	20-May-05	20.038	-87.466	Cameron, Andy	7.45		14.3%			0	0
190	RB05	Mahahual	Mexico	20-May-05	18.681	-87.714	Cameron, Andy	6.55		0.0%			0	0.5
1810	DB10	Dolphin Bay	Mexico	20-May-05	18.721	-87.691	Ponce-Taylor, Daniel	8.5		33.3%			0	2.15
1811	DB10	Dolphin Bay	Mexico	20-May-05	18.721	-87.691	Ponce-Taylor, Daniel	8.8		28.6%			0	2.15
1812	PDC20	Pirates Del Caribe	Mexico	20-May-05	18.660	-87.709	Ponce-Taylor, Daniel	14.15		53.8%			0	0.5
1813	RB05	Rio Bermejo	Mexico	20-May-05	18.675	-87.708	Ponce-Taylor, Daniel	6.55		0.0%			0	0.5
191	LC10	Pez Maya, Reserva de la Biosfer	Mexico	21-May-05	19.787	-87.433	Cameron, Andy	10.7		20.0%			0	0
192	LC20	Pez Maya, Reserva de la Biosfer	Mexico	21-May-05	19.786	-87.426	Cameron, Andy	17.25		9.1%			0	0
193	PAY10	Mahahual	Mexico	21-May-05	18.634	-87.720	Cameron, Andy	9.45		13.6%			0	0.5
194	PAY10	Mahahual	Mexico	21-May-05	18.634	-87.720	Cameron, Andy	9.4		9.1%			0	0.5
195	PAY10	Mahahual	Mexico	21-May-05	18.634	-87.720	Cameron, Andy	9.45		0.0%			0	0.5
196	PAY10	Mahahual	Mexico	21-May-05	18.634	-87.720	Cameron, Andy	9.8		16.7%			0	0.5
197	PDC20	Mahahual	Mexico	21-May-05	18.667	-87.714	Cameron, Andy	16.1		33.3%			0	0.5
1814	PAY10	Paytocal	Mexico	21-May-05	18.634	-87.719	Ponce-Taylor, Daniel	9.45		30.0%			0	0.5
1815	PAY10	Paytocal	Mexico	21-May-05	18.634	-87.719	Ponce-Taylor, Daniel	9.4		10.0%			0	0.5
1816	PAY10	Paytocal	Mexico	21-May-05	18.634	-87.719	Ponce-Taylor, Daniel	9.45		0.0%			0	0.5
1817	PAY10	Paytocal	Mexico	21-May-05	18.634	-87.719	Ponce-Taylor, Daniel	9.8		33.3%			0	0.5
1818	PDC20	Pirates Del Caribe	Mexico	21-May-05	18.660	-87.709	Ponce-Taylor, Daniel	16.1		50.0%			0	0.5
198	LPC10	Mahahual	Mexico	23-May-05	18.659	-87.717	Cameron, Andy	11.3		33.3%			0	0.5
199	LPC10	Mahahual	Mexico	23-May-05	18.659	-87.717	Cameron, Andy	10.65		5.3%			0	0.5
200	PL10	Pez Maya, Reserva de la Biosfer	Mexico	23-May-05	20.052	-87.466	Cameron, Andy	7.6		50.0%			0	0
201	PL10	Pez Maya, Reserva de la Biosfer	Mexico	23-May-05	20.052	-87.466	Cameron, Andy	7.6		53.8%			0	0
202	PL10	Pez Maya, Reserva de la Biosfer	Mexico	23-May-05	20.052	-87.466	Cameron, Andy	6.1		37.5%			0	0
203	PL10	Pez Maya, Reserva de la Biosfer	Mexico	23-May-05	20.052	-87.466	Cameron, Andy	5.8		25.0%			0	0
204	PSJ10	Pez Maya, Reserva de la Biosfer	Mexico	23-May-05	19.883	-87.418	Cameron, Andy	8.05		38.5%			0	0
205	PSJ10	Pez Maya, Reserva de la Biosfer	Mexico	23-May-05	19.883	-87.418	Cameron, Andy	10.4		47.1%			0	0
206	PSJ20	Pez Maya, Reserva de la Biosfer	Mexico	23-May-05	19.880	-87.412	Cameron, Andy	19.35		37.5%			0	0
207	PSJ20	Pez Maya, Reserva de la Biosfer	Mexico	23-May-05	19.880	-87.412	Cameron, Andy	19.8		66.7%			0	0
208	RB20	Mahahual	Mexico	23-May-05	18.681	-87.711	Cameron, Andy	14.75		32.0%			0	0.5
1819	LPC10	Los Preciones Cañones	Mexico	23-May-05	18.655	-87.710	Ponce-Taylor, Daniel	11.3		50.0%			0	0.5
1820	LPC10	Los Preciones Cañones	Mexico	23-May-05	18.655	-87.710	Ponce-Taylor, Daniel	10.65		14.3%			0	0.5
1821	RB20	Rio Bermejo	Mexico	23-May-05	18.675	-87.707	Ponce-Taylor, Daniel	14.75		46.7%			0	0.5
209	LC10	Pez Maya, Reserva de la Biosfer	Mexico	24-May-05	19.787	-87.433	Cameron, Andy	10.05		26.7%			0	0
210	LC20	Pez Maya, Reserva de la Biosfer	Mexico	24-May-05	19.786	-87.426	Cameron, Andy	17.25		47.1%			0	0
211	LE05	Mahahual	Mexico	24-May-05	18.720	-87.701	Cameron, Andy	5.2		34.6%			0	2.15
212	LE05	Mahahual	Mexico	24-May-05	18.720	-87.701	Cameron, Andy	5.5		18.2%			0	2.15
213	LPC10	Mahahual	Mexico	24-May-05	18.659	-87.717	Cameron, Andy	10.1		0.0%			0	0.5
214	PJ05	Pez Maya, Reserva de la Biosfer	Mexico	24-May-05	20.015	-87.465	Cameron, Andy	6.4		28.6%			0	0
1822	LE05	Los Escalones	Mexico	24-May-05	18.718	-87.701	Ponce-Taylor, Daniel	5.2		41.2%			0	2.15
1823	LE05	Los Escalones	Mexico	24-May-05	18.718	-87.701	Ponce-Taylor, Daniel	5.5		20.0%			0	2.15
1824	LPC10	Los Preciones Cañones	Mexico	24-May-05	18.655	-87.710	Ponce-Taylor, Daniel	10.1		20.0%			0	0.5
215	LE20	Mahahual	Mexico	25-May-05	18.720	-87.698	Cameron, Andy	15.5		45.8%			0	2.15
216	LPC05	Mahahual	Mexico	25-May-05	18.660	-87.719	Cameron, Andy	4		0.0%			0	0.5
217	LPC05	Mahahual	Mexico	25-May-05	18.660	-87.719	Cameron, Andy	4.6		0.0%			0	0.5
218	LPC10	Mahahual	Mexico	25-May-05	18.659	-87.717	Cameron, Andy	11.3		0.0%			0	0.5
219	PL05	Pez Maya, Reserva de la Biosfer	Mexico	25-May-05	20.050	-87.470	Cameron, Andy	3.05		16.7%			0	0
220	PL05	Pez Maya, Reserva de la Biosfer	Mexico	25-May-05	20.050	-87.470	Cameron, Andy	2.7		41.7%			0	0
221	SMDR10	Pez Maya, Reserva de la Biosfer	Mexico	25-May-05	19.967	-87.453	Cameron, Andy	9.15		11.5%			0	0
222	SMDR20	Pez Maya, Reserva de la Biosfer	Mexico	25-May-05	19.968	-87.452	Cameron, Andy	18.3		75.0%			0	0
1825	LE20	Los Escalones	Mexico	25-May-05	18.718	-87.692	Ponce-Taylor, Daniel	15.5		66.7%			0	2.15
1826	LPC05	Los Preciones Cañones	Mexico	25-May-05	18.655	-87.718	Ponce-Taylor, Daniel	4		0.0%			0	0.5
1827	LPC05	Los Preciones Cañones	Mexico	25-May-05	18.655	-87.718	Ponce-Taylor, Daniel	4.6		0.0%			0	0.5
1828	LPC10	Los Preciones Cañones	Mexico	25-May-05	18.655	-87.710	Ponce-Taylor, Daniel	11.3		22.2%			0	0.5

Observation Number	Reef or Site Name	Location	Country	Date (DD-MMM-YY)	Latitude (decimal degrees)	Longitude (decimal degrees)	Primary Contributor	Depth (m)	Cover bleached (%)	Colonies bleached (%)	Cover dead (%)	Colonies dead (%)	Observed DHW (°C-weeks)	Maximum DHW (°C-weeks)
223	LE05	Mahahual	Mexico	26-May-05	18.720	-87.701	Cameron, Andy	5.15		18.2%			0	2.15
224	LE05	Mahahual	Mexico	26-May-05	18.720	-87.701	Cameron, Andy	5.3		30.8%			0	2.15
225	PP05	Pez Maya, Reserva de la Biosfer	Mexico	26-May-05	20.040	-87.470	Cameron, Andy	3.5		23.1%			0	0
226	PX10	Pez Maya, Reserva de la Biosfer	Mexico	26-May-05	19.934	-87.434	Cameron, Andy	11.15		16.7%			0	0
227	PY20	Pez Maya, Reserva de la Biosfer	Mexico	26-May-05	19.973	-87.453	Cameron, Andy	19.2		20.0%			0	0
228	RB10	Mahahual	Mexico	26-May-05	18.681	-87.713	Cameron, Andy	9.75		6.9%			0	0.5
229	RB20	Mahahual	Mexico	26-May-05	18.681	-87.711	Cameron, Andy	12.05		10.8%			0	0.5
230	RB20	Mahahual	Mexico	26-May-05	18.681	-87.711	Cameron, Andy	14.65		5.6%			0	0.5
231	SMDR20	Pez Maya, Reserva de la Biosfer	Mexico	26-May-05	19.968	-87.452	Cameron, Andy	18		33.3%			0	0
1829	LE05	Los Escalones	Mexico	26-May-05	18.718	-87.701	Ponce-Taylor, Daniel	5.15		25.0%			0	2.15
1830	LE05	Los Escalones	Mexico	26-May-05	18.718	-87.701	Ponce-Taylor, Daniel	5.3		44.4%			0	2.15
1831	RB10	Rio Bermejo	Mexico	26-May-05	18.675	-87.708	Ponce-Taylor, Daniel	9.75		15.4%			0	0.5
1832	RB20	Rio Bermejo	Mexico	26-May-05	18.675	-87.707	Ponce-Taylor, Daniel	12.05		26.7%			0	0.5
1833	RB20	Rio Bermejo	Mexico	26-May-05	18.675	-87.707	Ponce-Taylor, Daniel	14.65		10.0%			0	0.5
232	DB10	Mahahual	Mexico	27-May-05	18.724	-87.697	Cameron, Andy	8.9		30.0%			0	2.15
233	DB10	Mahahual	Mexico	27-May-05	18.724	-87.697	Cameron, Andy	8.35		10.0%			0	2.15
234	LL10	Mahahual	Mexico	27-May-05	18.707	-87.706	Cameron, Andy	12.5		64.0%			0	2.15
235	PDC20	Mahahual	Mexico	27-May-05	18.667	-87.714	Cameron, Andy	13.55		5.0%			0	0.5
236	PDC20	Mahahual	Mexico	27-May-05	18.667	-87.714	Cameron, Andy	15.5		3.7%			0	0.5
237	PX10	Pez Maya, Reserva de la Biosfer	Mexico	27-May-05	19.934	-87.434	Cameron, Andy	12.2		35.7%			0	0
238	PX10	Pez Maya, Reserva de la Biosfer	Mexico	27-May-05	19.934	-87.434	Cameron, Andy	12.5		25.0%			0	0
239	PY20	Pez Maya, Reserva de la Biosfer	Mexico	27-May-05	19.973	-87.453	Cameron, Andy	18.3		31.3%			0	0
240	PY20	Pez Maya, Reserva de la Biosfer	Mexico	27-May-05	19.973	-87.453	Cameron, Andy	18.9		22.2%			0	0
241	SMDR20	Pez Maya, Reserva de la Biosfer	Mexico	27-May-05	19.968	-87.452	Cameron, Andy	17.4		83.3%			0	0
242	SMDR20	Pez Maya, Reserva de la Biosfer	Mexico	27-May-05	19.968	-87.452	Cameron, Andy	18.6		50.0%			0	0
1834	DB10	Dolphin Bay	Mexico	27-May-05	18.721	-87.691	Ponce-Taylor, Daniel	8.9		47.4%			0	2.15
1835	DB10	Dolphin Bay	Mexico	27-May-05	18.721	-87.691	Ponce-Taylor, Daniel	8.35		13.3%			0	2.15
1836	LL10	Las Llamaradas	Mexico	27-May-05	18.704	-87.704	Ponce-Taylor, Daniel	12.5		60.0%			0	0.5
1837	PDC20	Pirates Del Caribe	Mexico	27-May-05	18.660	-87.709	Ponce-Taylor, Daniel	13.55		10.0%			0	0.5
1838	PDC20	Pirates Del Caribe	Mexico	27-May-05	18.660	-87.709	Ponce-Taylor, Daniel	15.5		14.3%			0	0.5
243	PDC05	Mahahual	Mexico	28-May-05	18.667	-87.717	Cameron, Andy	6.4		25.0%			0	0.5
244	PDC05	Mahahual	Mexico	28-May-05	18.667	-87.717	Cameron, Andy	6.7		33.3%			0	0.5
245	PY20	Pez Maya, Reserva de la Biosfer	Mexico	28-May-05	19.973	-87.453	Cameron, Andy	18.45		21.4%			0	0
246	RB20	Mahahual	Mexico	28-May-05	18.681	-87.711	Cameron, Andy	15.35		25.7%			0	0.5
247	RB20	Mahahual	Mexico	28-May-05	18.681	-87.711	Cameron, Andy	14.15		7.7%			0	0.5
1839	PDC05	Pirates Del Caribe	Mexico	28-May-05	18.660	-87.717	Ponce-Taylor, Daniel	6.4		25.0%			0	0.5
1840	PDC05	Pirates Del Caribe	Mexico	28-May-05	18.660	-87.717	Ponce-Taylor, Daniel	6.7		42.9%			0	0.5
1841	RB20	Rio Bermejo	Mexico	28-May-05	18.675	-87.707	Ponce-Taylor, Daniel	15.35		39.1%			0	0.5
1842	RB20	Rio Bermejo	Mexico	28-May-05	18.675	-87.707	Ponce-Taylor, Daniel	14.15		23.1%			0	0.5
248	LE20	Mahahual	Mexico	31-May-05	18.720	-87.698	Cameron, Andy	17.8		17.6%			0	2.15
249	LE20	Mahahual	Mexico	31-May-05	18.720	-87.698	Cameron, Andy	17.85		20.0%			0	2.15
250	LL10	Mahahual	Mexico	31-May-05	18.707	-87.706	Cameron, Andy	9.6		15.4%			0	2.15
251	LL10	Mahahual	Mexico	31-May-05	18.707	-87.706	Cameron, Andy	9.9		28.6%			0	2.15
1843	LE20	Los Escalones	Mexico	31-May-05	18.718	-87.692	Ponce-Taylor, Daniel	17.8		30.0%			0	2.15
1844	LE20	Los Escalones	Mexico	31-May-05	18.718	-87.692	Ponce-Taylor, Daniel	17.85		44.4%			0	2.15
1845	LL10	Las Llamaradas	Mexico	31-May-05	18.704	-87.704	Ponce-Taylor, Daniel	9.6		18.2%			0	0.5
1846	LL10	Las Llamaradas	Mexico	31-May-05	18.704	-87.704	Ponce-Taylor, Daniel	9.9		47.6%			0	0.5
252	PL20	Pez Maya, Reserva de la Biosfer	Mexico	1-Jun-05	20.051	-87.463	Cameron, Andy	11.45		52.4%			0	0
253	PL20	Pez Maya, Reserva de la Biosfer	Mexico	1-Jun-05	20.051	-87.463	Cameron, Andy	15.25		66.7%			0	0
254	PX10	Pez Maya, Reserva de la Biosfer	Mexico	1-Jun-05	19.934	-87.434	Cameron, Andy	10.35		35.3%			0	0
255	SMDR10	Pez Maya, Reserva de la Biosfer	Mexico	1-Jun-05	19.967	-87.453	Cameron, Andy	8.65		16.7%			0	0
256	LL05	Mahahual	Mexico	2-Jun-05	18.707	-87.707	Cameron, Andy	4.75		11.1%			0	2.15
257	LL05	Mahahual	Mexico	2-Jun-05	18.707	-87.707	Cameron, Andy	4.6		0.0%			0	2.15
258	LL05	Mahahual	Mexico	2-Jun-05	18.707	-87.707	Cameron, Andy	4.6		0.0%			0	2.15
259	LPC20	Mahahual	Mexico	2-Jun-05	18.660	-87.716	Cameron, Andy	15.75		26.1%			0	0.5
260	PL20	Pez Maya, Reserva de la Biosfer	Mexico	2-Jun-05	20.051	-87.463	Cameron, Andy	14.3		27.8%			0	0
261	PL20	Pez Maya, Reserva de la Biosfer	Mexico	2-Jun-05	20.051	-87.463	Cameron, Andy	11.75		29.4%			0	0
262	PX20	Pez Maya, Reserva de la Biosfer	Mexico	2-Jun-05	19.933	-87.432	Cameron, Andy	18		38.9%			0	0

Observation Number	Reef or Site Name	Location	Country	Date (DD-MMM-YY)	Latitude (decimal degrees)	Longitude (decimal degrees)	Primary Contributor	Depth (m)	Cover bleached (%)	Colonies bleached (%)	Cover dead (%)	Colonies dead (%)	Observed DHW (°C-weeks)	Maximum DHW (°C-weeks)
1847	LL05	Las Llamaradas	Mexico	2-Jun-05	18.704	-87.704	Ponce-Taylor, Daniel	4.75		14.3%			0	0.5
1848	LL05	Las Llamaradas	Mexico	2-Jun-05	18.704	-87.704	Ponce-Taylor, Daniel	4.6		0.0%			0	0.5
1849	LL05	Las Llamaradas	Mexico	2-Jun-05	18.704	-87.704	Ponce-Taylor, Daniel	4.6		0.0%			0	0.5
1850	LPC20	Los Preciones Cañones	Mexico	2-Jun-05	18.655	-87.709	Ponce-Taylor, Daniel	15.75		38.5%			0	0.5
263	LPC05	Mahahual	Mexico	3-Jun-05	18.660	-87.719	Cameron, Andy	5.2		0.0%			0	0.5
264	LPC05	Mahahual	Mexico	3-Jun-05	18.660	-87.719	Cameron, Andy	5.2		25.0%			0	0.5
265	LPC20	Mahahual	Mexico	3-Jun-05	18.660	-87.716	Cameron, Andy	15.5		7.3%			0	0.5
266	LPC20	Mahahual	Mexico	3-Jun-05	18.660	-87.716	Cameron, Andy	20.9		34.8%			0	0.5
267	LPC20	Mahahual	Mexico	3-Jun-05	18.660	-87.716	Cameron, Andy	14.6		0.0%			0	0.5
1851	LPC05	Los Preciones Cañones	Mexico	3-Jun-05	18.655	-87.718	Ponce-Taylor, Daniel	5.2		0.0%			0	0.5
1852	LPC05	Los Preciones Cañones	Mexico	3-Jun-05	18.655	-87.718	Ponce-Taylor, Daniel	5.2		33.3%			0	0.5
1853	LPC20	Los Preciones Cañones	Mexico	3-Jun-05	18.655	-87.709	Ponce-Taylor, Daniel	15.5		17.6%			0	0.5
1854	LPC20	Los Preciones Cañones	Mexico	3-Jun-05	18.655	-87.709	Ponce-Taylor, Daniel	20.9		53.3%			0	0.5
1855	LPC20	Los Preciones Cañones	Mexico	3-Jun-05	18.655	-87.709	Ponce-Taylor, Daniel	14.6		12.5%			0	0.5
268	BUC10	Mahahual	Mexico	4-Jun-05	18.687	-87.711	Cameron, Andy	10.25		10.3%			0	0.5
269	LE20	Mahahual	Mexico	4-Jun-05	18.720	-87.698	Cameron, Andy	14.6		20.7%			0	2.15
270	PAY05	Mahahual	Mexico	4-Jun-05	18.635	-87.722	Cameron, Andy	4.9		0.0%			0	0.5
271	PAY05	Mahahual	Mexico	4-Jun-05	18.635	-87.722	Cameron, Andy	4.9		0.0%			0	0.5
272	PAY20	Mahahual	Mexico	4-Jun-05	18.635	-87.719	Cameron, Andy	19.55		47.4%			0	0.5
1856	BUC10	Bucaneros	Mexico	4-Jun-05	18.685	-87.706	Ponce-Taylor, Daniel	10.25		20.0%			0	0.5
1857	LE20	Los Escalones	Mexico	4-Jun-05	18.718	-87.692	Ponce-Taylor, Daniel	14.6		31.3%			0	2.15
1858	PAY05	Paytocal	Mexico	4-Jun-05	18.634	-87.720	Ponce-Taylor, Daniel	4.9		0.0%			0	0.5
1859	PAY05	Paytocal	Mexico	4-Jun-05	18.634	-87.720	Ponce-Taylor, Daniel	4.9		0.0%			0	0.5
1860	PAY20	Paytocal	Mexico	4-Jun-05	18.634	-87.718	Ponce-Taylor, Daniel	19.55		64.3%			0	0.5
273	PAY05	Mahahual	Mexico	8-Jun-05	18.635	-87.722	Cameron, Andy	5.35		33.3%			0	0.5
274	PAY05	Mahahual	Mexico	8-Jun-05	18.635	-87.722	Cameron, Andy	5.5		50.0%			0	0.5
275	PAY05	Mahahual	Mexico	8-Jun-05	18.635	-87.722	Cameron, Andy	4.6		60.0%			0	0.5
276	PAY20	Mahahual	Mexico	8-Jun-05	18.635	-87.719	Cameron, Andy	19.8		44.4%			0	0.5
277	PAY20	Mahahual	Mexico	8-Jun-05	18.635	-87.719	Cameron, Andy	17.85		31.0%			0	0.5
278	PAY20	Mahahual	Mexico	8-Jun-05	18.635	-87.719	Cameron, Andy	17.7		41.2%			0	0.5
1861	PAY05	Paytocal	Mexico	8-Jun-05	18.634	-87.720	Ponce-Taylor, Daniel	5.35		33.3%			0	0.5
1862	PAY05	Paytocal	Mexico	8-Jun-05	18.634	-87.720	Ponce-Taylor, Daniel	5.5		50.0%			0	0.5
1863	PAY05	Paytocal	Mexico	8-Jun-05	18.634	-87.720	Ponce-Taylor, Daniel	4.6		60.0%			0	0.5
1864	PAY10	Paytocal	Mexico	8-Jun-05	18.634	-87.719	Ponce-Taylor, Daniel	9.1		61.5%			0	0.5
1865	PAY20	Paytocal	Mexico	8-Jun-05	18.634	-87.718	Ponce-Taylor, Daniel	19.8		46.7%			0	0.5
1866	PAY20	Paytocal	Mexico	8-Jun-05	18.634	-87.718	Ponce-Taylor, Daniel	17.85		58.3%			0	0.5
1867	PAY20	Paytocal	Mexico	8-Jun-05	18.634	-87.718	Ponce-Taylor, Daniel	17.7		53.8%			0	0.5
279	LE20	Mahahual	Mexico	9-Jun-05	18.720	-87.698	Cameron, Andy	17.7		23.8%			0	2.15
280	PAY20	Mahahual	Mexico	9-Jun-05	18.635	-87.719	Cameron, Andy	17.55		20.0%			0	0.5
1868	LE20	Los Escalones	Mexico	9-Jun-05	18.718	-87.692	Ponce-Taylor, Daniel	17.7		41.7%			0	2.15
1869	PAY20	Paytocal	Mexico	9-Jun-05	18.634	-87.718	Ponce-Taylor, Daniel	17.55		40.0%			0	0.5
281	BUC20	Mahahual	Mexico	10-Jun-05	18.687	-87.709	Cameron, Andy	17.5		11.5%			0	0.5
282	BUC20	Mahahual	Mexico	10-Jun-05	18.687	-87.709	Cameron, Andy	16		11.9%			0	0.5
283	LPC05	Mahahual	Mexico	10-Jun-05	18.660	-87.719	Cameron, Andy	3.85		0.0%			0	0.5
284	LPC20	Mahahual	Mexico	10-Jun-05	18.660	-87.716	Cameron, Andy	21.2		30.0%			0	0.5
285	PC05	Pez Maya, Reserva de la Biosfer	Mexico	10-Jun-05	20.100	-87.464	Cameron, Andy	5.8		66.7%			0	0
286	PC05	Pez Maya, Reserva de la Biosfer	Mexico	10-Jun-05	20.100	-87.464	Cameron, Andy	5.8		77.8%			0	0
287	PC10	Pez Maya, Reserva de la Biosfer	Mexico	10-Jun-05	20.099	-87.462	Cameron, Andy	9.1		45.5%			0	0
288	PDC05	Mahahual	Mexico	10-Jun-05	18.667	-87.717	Cameron, Andy	5.2		75.0%			0	0.5
289	PL20	Pez Maya, Reserva de la Biosfer	Mexico	10-Jun-05	20.051	-87.463	Cameron, Andy	14		9.1%			0	0
290	PP05	Pez Maya, Reserva de la Biosfer	Mexico	10-Jun-05	20.040	-87.470	Cameron, Andy	3.4		27.3%			0	0
291	PP05	Pez Maya, Reserva de la Biosfer	Mexico	10-Jun-05	20.040	-87.470	Cameron, Andy	4.15		41.7%			0	0
1870	BUC20	Bucaneros	Mexico	10-Jun-05	18.685	-87.706	Ponce-Taylor, Daniel	17.5		25.0%			0	0.5
1871	BUC20	Bucaneros	Mexico	10-Jun-05	18.685	-87.706	Ponce-Taylor, Daniel	16		29.4%			0	0.5
1872	LPC05	Los Preciones Cañones	Mexico	10-Jun-05	18.655	-87.718	Ponce-Taylor, Daniel	3.85		0.0%			0	0.5
1873	LPC20	Los Preciones Cañones	Mexico	10-Jun-05	18.655	-87.709	Ponce-Taylor, Daniel	21.2		50.0%			0	0.5
1874	PDC05	Pirates Del Caribe	Mexico	10-Jun-05	18.660	-87.717	Ponce-Taylor, Daniel	5.2		72.7%			0	0.5
292	BUC10	Mahahual	Mexico	11-Jun-05	18.687	-87.711	Cameron, Andy	9.25		3.7%			0	0.5

Observation Number	Reef or Site Name	Location	Country	Date (DD-MMM-YY)	Latitude (decimal degrees)	Longitude (decimal degrees)	Primary Contributor	Depth (m)	Cover bleached (%)	Colonies bleached (%)	Cover dead (%)	Colonies dead (%)	Observed DHW (°C-weeks)	Maximum DHW (°C-weeks)
293	BUC10	Mahahual	Mexico	11-Jun-05	18.687	-87.711	Cameron, Andy	10.4		12.5%			0	0.5
294	BUC20	Mahahual	Mexico	11-Jun-05	18.687	-87.709	Cameron, Andy	18.25		0.0%			0	0.5
295	BUC20	Mahahual	Mexico	11-Jun-05	18.687	-87.709	Cameron, Andy	14.5		6.5%			0	0.5
296	LC10	Pez Maya, Reserva de la Biosfer	Mexico	11-Jun-05	19.787	-87.433	Cameron, Andy	9.6		35.7%			0	0
297	LC10	Pez Maya, Reserva de la Biosfer	Mexico	11-Jun-05	19.787	-87.433	Cameron, Andy	10.4		22.2%			0	0
298	LC10	Pez Maya, Reserva de la Biosfer	Mexico	11-Jun-05	19.787	-87.433	Cameron, Andy	11.75		20.0%			0	0
299	LC20	Pez Maya, Reserva de la Biosfer	Mexico	11-Jun-05	19.786	-87.426	Cameron, Andy	18.3		38.5%			0	0
300	LC20	Pez Maya, Reserva de la Biosfer	Mexico	11-Jun-05	19.786	-87.426	Cameron, Andy	17.4		0.0%			0	0
301	LC20	Pez Maya, Reserva de la Biosfer	Mexico	11-Jun-05	19.786	-87.426	Cameron, Andy	16.8		31.6%			0	0
1875	BUC10	Bucaneros	Mexico	11-Jun-05	18.685	-87.706	Ponce-Taylor, Daniel	9.25		11.1%			0	0.5
1876	BUC10	Bucaneros	Mexico	11-Jun-05	18.685	-87.706	Ponce-Taylor, Daniel	10.4		18.2%			0	0.5
1877	BUC20	Bucaneros	Mexico	11-Jun-05	18.685	-87.706	Ponce-Taylor, Daniel	18.25		0.0%			0	0.5
1878	BUC20	Bucaneros	Mexico	11-Jun-05	18.685	-87.706	Ponce-Taylor, Daniel	14.5		14.3%			0	0.5
302	BUC05	Mahahual	Mexico	12-Jun-05	18.687	-87.712	Cameron, Andy	4.9		0.0%			0	0.5
303	BUC05	Mahahual	Mexico	12-Jun-05	18.687	-87.712	Cameron, Andy	5.8		0.0%			0	0.5
304	BUC20	Mahahual	Mexico	12-Jun-05	18.687	-87.709	Cameron, Andy	18.6		0.0%			0	0.5
1879	BUC05	Bucaneros	Mexico	12-Jun-05	18.685	-87.707	Ponce-Taylor, Daniel	4.9		0.0%			0	0.5
1880	BUC05	Bucaneros	Mexico	12-Jun-05	18.685	-87.707	Ponce-Taylor, Daniel	5.8		0.0%			0	0.5
1881	BUC20	Bucaneros	Mexico	12-Jun-05	18.685	-87.706	Ponce-Taylor, Daniel	18.6		0.0%			0	0.5
305	FV20	Mahahual	Mexico	13-Jun-05	18.742	-87.674	Cameron, Andy	21		0.0%			0	2.15
306	LL20	Mahahual	Mexico	13-Jun-05	18.707	-87.704	Cameron, Andy	14		23.5%			0	2.15
1882	FV20	Faro Viejo	Mexico	13-Jun-05	18.738	-87.671	Ponce-Taylor, Daniel	21		0.0%			0	2.15
1883	LL20	Las Llamaradas	Mexico	13-Jun-05	18.704	-87.702	Ponce-Taylor, Daniel	14		42.9%			0	0.5
307	BUC05	Mahahual	Mexico	14-Jun-05	18.687	-87.712	Cameron, Andy	6.85		33.3%			0	0.5
308	BUC05	Mahahual	Mexico	14-Jun-05	18.687	-87.712	Cameron, Andy	7.3		57.1%			0	0.5
309	BUC05	Mahahual	Mexico	14-Jun-05	18.687	-87.712	Cameron, Andy	6.4		30.0%			0	0.5
310	PP05	Pez Maya, Reserva de la Biosfer	Mexico	14-Jun-05	20.040	-87.470	Cameron, Andy	3.2		33.3%			0	0
311	PX20	Pez Maya, Reserva de la Biosfer	Mexico	14-Jun-05	19.933	-87.432	Cameron, Andy	18.3		22.2%			0	0
312	PX20	Pez Maya, Reserva de la Biosfer	Mexico	14-Jun-05	19.933	-87.432	Cameron, Andy	15.85		64.7%			0	0
313	PX20	Pez Maya, Reserva de la Biosfer	Mexico	14-Jun-05	19.933	-87.432	Cameron, Andy	17.05		0.0%			0	0
314	PX20	Pez Maya, Reserva de la Biosfer	Mexico	14-Jun-05	19.933	-87.432	Cameron, Andy	16.95		75.0%			0	0
1884	BUC05	Bucaneros	Mexico	14-Jun-05	18.685	-87.707	Ponce-Taylor, Daniel	6.85		33.3%			0	0.5
1885	BUC05	Bucaneros	Mexico	14-Jun-05	18.685	-87.707	Ponce-Taylor, Daniel	7.3		57.1%			0	0.5
1886	BUC05	Bucaneros	Mexico	14-Jun-05	18.685	-87.707	Ponce-Taylor, Daniel	6.4		33.3%			0	0.5
315	PC05	Pez Maya, Reserva de la Biosfer	Mexico	15-Jun-05	20.100	-87.464	Cameron, Andy	6.4		44.4%			0	0
316	PC05	Pez Maya, Reserva de la Biosfer	Mexico	15-Jun-05	20.100	-87.464	Cameron, Andy	6.4		83.3%			0	0
317	PC05	Pez Maya, Reserva de la Biosfer	Mexico	15-Jun-05	20.100	-87.464	Cameron, Andy	6.1		37.5%			0	0
318	PSJ20	Pez Maya, Reserva de la Biosfer	Mexico	15-Jun-05	19.880	-87.412	Cameron, Andy	20.1		81.8%			0	0
319	PSJ20	Pez Maya, Reserva de la Biosfer	Mexico	15-Jun-05	19.880	-87.412	Cameron, Andy	18.15		100.0%			0	0
320	PP05	Pez Maya, Reserva de la Biosfer	Mexico	16-Jun-05	20.040	-87.470	Cameron, Andy	2.85		26.7%			0	0
321	PSJ05	Pez Maya, Reserva de la Biosfer	Mexico	16-Jun-05	19.883	-87.426	Cameron, Andy	3.05		60.0%			0	0
322	PSJ05	Pez Maya, Reserva de la Biosfer	Mexico	16-Jun-05	19.883	-87.426	Cameron, Andy	2.6		17.6%			0	0
323	PSJ05	Pez Maya, Reserva de la Biosfer	Mexico	16-Jun-05	19.883	-87.426	Cameron, Andy	2.45		0.0%			0	0
1	Paso del Cedral	Cozumel	Mexico	25-Jul-05	20.374	-87.029	Alvarez, Lorenzo	13.94		7.9%		0.0%	0	0
2	Yucab	Cozumel	Mexico	26-Jul-05	20.421	-87.017	Alvarez, Lorenzo	12.6		1.8%		0.0%	0	0
3	Chancanaab	Cozumel	Mexico	27-Jul-05	20.441	-87.002	Alvarez, Lorenzo	11.89		3.6%		0.0%	0	0
324	LL10	Mahahual	Mexico	27-Jul-05	18.707	-87.706	Cameron, Andy	10.05		19.2%			0	2.15
1887	LL10	Las Llamaradas	Mexico	27-Jul-05	18.704	-87.704	Ponce-Taylor, Daniel	10.05		21.4%			0	0.5
325	LL10	Mahahual	Mexico	28-Jul-05	18.707	-87.706	Cameron, Andy	9		23.5%			0	2.15
1888	LL10	Las Llamaradas	Mexico	28-Jul-05	18.704	-87.704	Ponce-Taylor, Daniel	9		30.0%			0	0.5
4	Paraiso	Cozumel	Mexico	29-Jul-05	20.469	-86.983	Alvarez, Lorenzo	9.88		0.0%		0.0%	0	0
326	DB10	Mahahual	Mexico	29-Jul-05	18.724	-87.697	Cameron, Andy	10.15		5.9%			0	2.15
327	DB10	Mahahual	Mexico	29-Jul-05	18.724	-87.697	Cameron, Andy	10.1		10.0%			0	2.15
328	LE10	Mahahual	Mexico	29-Jul-05	18.720	-87.699	Cameron, Andy	10.05		31.8%			0	2.15
1889	DB10	Dolphin Bay	Mexico	29-Jul-05	18.721	-87.691	Ponce-Taylor, Daniel	10.15		11.1%			0	2.15
1890	DB10	Dolphin Bay	Mexico	29-Jul-05	18.721	-87.691	Ponce-Taylor, Daniel	10.1		25.0%			0	2.15
1891	LE10	Los Escalones	Mexico	29-Jul-05	18.718	-87.693	Ponce-Taylor, Daniel	10.05		50.0%			0	2.15
329	LE05	Mahahual	Mexico	31-Jul-05	18.720	-87.701	Cameron, Andy	4.7		30.0%			0	2.15

Observation Number	Reef or Site Name	Location	Country	Date (DD-MMM-YY)	Latitude (decimal degrees)	Longitude (decimal degrees)	Primary Contributor	Depth (m)	Cover bleached (%)	Colonies bleached (%)	Cover dead (%)	Colonies dead (%)	Observed DHW (°C-weeks)	Maximum DHW (°C-weeks)
330	LE10	Mahahual	Mexico	31-Jul-05	18.720	-87.699	Cameron, Andy	9.1		16.7%			0	2.15
1892	LE05	Los Escalones	Mexico	31-Jul-05	18.718	-87.701	Ponce-Taylor, Daniel	4.7		44.4%			0	2.15
1893	LE10	Los Escalones	Mexico	31-Jul-05	18.718	-87.693	Ponce-Taylor, Daniel	9.1		13.3%			0	2.15
331	LE10	Mahahual	Mexico	1-Aug-05	18.720	-87.699	Cameron, Andy	9.95		35.3%			0	2.15
332	RB10	Mahahual	Mexico	1-Aug-05	18.681	-87.713	Cameron, Andy	9.1		4.0%			0	0.5
333	RB10	Mahahual	Mexico	1-Aug-05	18.681	-87.713	Cameron, Andy	9.3		3.2%			0	0.5
1894	LE10	Los Escalones	Mexico	1-Aug-05	18.718	-87.693	Ponce-Taylor, Daniel	9.95		57.1%			0	2.15
1895	RB10	Rio Bermejo	Mexico	1-Aug-05	18.675	-87.708	Ponce-Taylor, Daniel	9.1		50.0%			0	0.5
1896	RB10	Rio Bermejo	Mexico	1-Aug-05	18.675	-87.708	Ponce-Taylor, Daniel	9.3		7.7%			0	0.5
334	LL05	Mahahual	Mexico	2-Aug-05	18.707	-87.707	Cameron, Andy	5.35		16.7%			0	2.15
335	LL05	Mahahual	Mexico	2-Aug-05	18.707	-87.707	Cameron, Andy	4.8		30.0%			0	2.15
336	PL10	Pez Maya, Reserva de la Biosfer	Mexico	2-Aug-05	20.052	-87.466	Cameron, Andy	6.1		35.3%			0	0
337	PL10	Pez Maya, Reserva de la Biosfer	Mexico	2-Aug-05	20.052	-87.466	Cameron, Andy	6.4		58.8%			0	0
338	RB10	Mahahual	Mexico	2-Aug-05	18.681	-87.713	Cameron, Andy	10.25		26.3%			0	0.5
339	RB10	Mahahual	Mexico	2-Aug-05	18.681	-87.713	Cameron, Andy	9.6		15.0%			0	0.5
1897	LL05	Las Llamaradas	Mexico	2-Aug-05	18.704	-87.704	Ponce-Taylor, Daniel	5.35		25.0%			0	0.5
1898	LL05	Las Llamaradas	Mexico	2-Aug-05	18.704	-87.704	Ponce-Taylor, Daniel	4.8		46.2%			0	0.5
1899	RB10	Rio Bermejo	Mexico	2-Aug-05	18.675	-87.708	Ponce-Taylor, Daniel	10.25		45.5%			0	0.5
1900	RB10	Rio Bermejo	Mexico	2-Aug-05	18.675	-87.708	Ponce-Taylor, Daniel	9.6		21.4%			0	0.5
5	Colombia	Cozumel	Mexico	3-Aug-05	20.325	-87.027	Alvarez, Lorenzo	10.59		1.4%		0.0%	0	0
340	LE05	Mahahual	Mexico	3-Aug-05	18.720	-87.701	Cameron, Andy	4.65		0.0%			0	2.15
341	LE05	Mahahual	Mexico	3-Aug-05	18.720	-87.701	Cameron, Andy	5.55		10.0%			0	2.15
342	LE20	Mahahual	Mexico	3-Aug-05	18.720	-87.698	Cameron, Andy	17.85		15.0%			0	2.15
343	LE20	Mahahual	Mexico	3-Aug-05	18.720	-87.698	Cameron, Andy	18.2		0.0%			0	2.15
344	PL10	Pez Maya, Reserva de la Biosfer	Mexico	3-Aug-05	20.052	-87.466	Cameron, Andy	6.4		26.3%			0	0
345	PL10	Pez Maya, Reserva de la Biosfer	Mexico	3-Aug-05	20.052	-87.466	Cameron, Andy	7.15		36.8%			0	0
346	SMDR10	Pez Maya, Reserva de la Biosfer	Mexico	3-Aug-05	19.967	-87.453	Cameron, Andy	10.5		0.0%			0	0
1901	LE05	Los Escalones	Mexico	3-Aug-05	18.718	-87.701	Ponce-Taylor, Daniel	4.65		0.0%			0	2.15
1902	LE05	Los Escalones	Mexico	3-Aug-05	18.718	-87.701	Ponce-Taylor, Daniel	5.55		14.3%			0	2.15
1903	LE20	Los Escalones	Mexico	3-Aug-05	18.718	-87.692	Ponce-Taylor, Daniel	17.85		37.5%			0	2.15
1904	LE20	Los Escalones	Mexico	3-Aug-05	18.718	-87.692	Ponce-Taylor, Daniel	18.2		0.0%			0	2.15
6	Dalila	Cozumel	Mexico	4-Aug-05	20.349	-87.029	Alvarez, Lorenzo	12.28		2.9%		0.0%	0	0
347	LE20	Mahahual	Mexico	4-Aug-05	18.720	-87.698	Cameron, Andy	19.05		10.0%			0	2.15
348	LL05	Mahahual	Mexico	4-Aug-05	18.707	-87.707	Cameron, Andy	5.95		60.0%			0	2.15
349	PL10	Pez Maya, Reserva de la Biosfer	Mexico	4-Aug-05	20.052	-87.466	Cameron, Andy	6.4		0.0%			0	0
350	RB10	Mahahual	Mexico	4-Aug-05	18.681	-87.713	Cameron, Andy	9.25		20.0%			0	0.5
1905	LE20	Los Escalones	Mexico	4-Aug-05	18.718	-87.692	Ponce-Taylor, Daniel	19.05		14.3%			0	2.15
1906	LL05	Las Llamaradas	Mexico	4-Aug-05	18.704	-87.704	Ponce-Taylor, Daniel	5.95		70.0%			0	0.5
1907	RB10	Rio Bermejo	Mexico	4-Aug-05	18.675	-87.708	Ponce-Taylor, Daniel	9.25		18.2%			0	0.5
351	RB20	Mahahual	Mexico	5-Aug-05	18.681	-87.711	Cameron, Andy	14.8		3.7%			0	0.5
352	RB20	Mahahual	Mexico	5-Aug-05	18.681	-87.711	Cameron, Andy	16.15		68.8%			0	0.5
1908	RB20	Rio Bermejo	Mexico	5-Aug-05	18.675	-87.707	Ponce-Taylor, Daniel	14.8		11.1%			0	0.5
1909	RB20	Rio Bermejo	Mexico	5-Aug-05	18.675	-87.707	Ponce-Taylor, Daniel	16.15		50.0%			0	0.5
353	DB05	Mahahual	Mexico	8-Aug-05	18.724	-87.698	Cameron, Andy	5.35		58.3%			0	2.15
1910	DB05	Dolphin Bay	Mexico	8-Aug-05	18.721	-87.692	Ponce-Taylor, Daniel	5.35		63.6%			0	2.15
354	DB05	Mahahual	Mexico	9-Aug-05	18.724	-87.698	Cameron, Andy	6.4		27.8%			0	2.15
1911	DB05	Dolphin Bay	Mexico	9-Aug-05	18.721	-87.692	Ponce-Taylor, Daniel	6.4		38.5%			0	2.15
355	RB05	Mahahual	Mexico	10-Aug-05	18.681	-87.714	Cameron, Andy	6.25		0.0%			0	0.5
356	RB20	Mahahual	Mexico	10-Aug-05	18.681	-87.711	Cameron, Andy	14.45		10.0%			0	0.5
1912	RB05	Rio Bermejo	Mexico	10-Aug-05	18.675	-87.708	Ponce-Taylor, Daniel	6.25		0.0%			0	0.5
1913	RB20	Rio Bermejo	Mexico	10-Aug-05	18.675	-87.707	Ponce-Taylor, Daniel	14.45		25.0%			0	0.5
357	DB05	Mahahual	Mexico	11-Aug-05	18.724	-87.698	Cameron, Andy	4.75		30.0%			0	2.15
1914	DB05	Dolphin Bay	Mexico	11-Aug-05	18.721	-87.692	Ponce-Taylor, Daniel	4.75		42.9%			0	2.15
358	DB10	Mahahual	Mexico	12-Aug-05	18.724	-87.697	Cameron, Andy	8.35		18.5%			0	2.15
359	LE20	Mahahual	Mexico	12-Aug-05	18.720	-87.698	Cameron, Andy	16.05		52.6%			0	2.15
360	PJ05	Pez Maya, Reserva de la Biosfer	Mexico	12-Aug-05	20.015	-87.465	Cameron, Andy	5.95		33.3%			0	0
361	PJ05	Pez Maya, Reserva de la Biosfer	Mexico	12-Aug-05	20.015	-87.465	Cameron, Andy	6.7		33.3%			0	0
362	PJ05	Pez Maya, Reserva de la Biosfer	Mexico	12-Aug-05	20.015	-87.465	Cameron, Andy	4.6		11.1%			0	0

Observation Number	Reef or Site Name	Location	Country	Date (DD-MMM-YY)	Latitude (decimal degrees)	Longitude (decimal degrees)	Primary Contributor	Depth (m)	Cover bleached (%)	Colonies bleached (%)	Cover dead (%)	Colonies dead (%)	Observed DHW (°C-weeks)	Maximum DHW (°C-weeks)
1915	DB10	Dolphin Bay	Mexico	12-Aug-05	18.721	-87.691	Ponce-Taylor, Daniel	8.35		41.7%			0	2.15
1916	LE20	Los Escalones	Mexico	12-Aug-05	18.718	-87.692	Ponce-Taylor, Daniel	16.05		50.0%			0	2.15
363	PAY05	Mahahual	Mexico	15-Aug-05	18.635	-87.722	Cameron, Andy	4.75		33.3%			0	0.5
364	PAY05	Mahahual	Mexico	15-Aug-05	18.635	-87.722	Cameron, Andy	5.05		33.3%			0	0.5
365	PAY05	Mahahual	Mexico	15-Aug-05	18.635	-87.722	Cameron, Andy	4		0.0%			0	0.5
366	PAY20	Mahahual	Mexico	15-Aug-05	18.635	-87.719	Cameron, Andy	19.35		6.7%			0	0.5
367	PJ10	Pez Maya, Reserva de la Biosfer	Mexico	15-Aug-05	20.017	-87.462	Cameron, Andy	8.8		50.0%			0	0
368	PJ10	Pez Maya, Reserva de la Biosfer	Mexico	15-Aug-05	20.017	-87.462	Cameron, Andy	8.95		50.0%			0	0
369	PJ10	Pez Maya, Reserva de la Biosfer	Mexico	15-Aug-05	20.017	-87.462	Cameron, Andy	8.65		50.0%			0	0
1917	PAY05	Paytocal	Mexico	15-Aug-05	18.634	-87.720	Ponce-Taylor, Daniel	4.75		33.3%			0	0.5
1918	PAY05	Paytocal	Mexico	15-Aug-05	18.634	-87.720	Ponce-Taylor, Daniel	5.05		33.3%			0	0.5
1919	PAY05	Paytocal	Mexico	15-Aug-05	18.634	-87.720	Ponce-Taylor, Daniel	4		0.0%			0	0.5
1920	PAY20	Paytocal	Mexico	15-Aug-05	18.634	-87.718	Ponce-Taylor, Daniel	19.35		14.3%			0	0.5
370	LE20	Mahahual	Mexico	16-Aug-05	18.720	-87.698	Cameron, Andy	18.75		13.0%			0	2.15
1921	LE20	Los Escalones	Mexico	16-Aug-05	18.718	-87.692	Ponce-Taylor, Daniel	18.75		23.1%			0	2.15
371	PDC10	Mahahual	Mexico	17-Aug-05	18.667	-87.716	Cameron, Andy	10.7		33.3%			0	0.5
372	PDC10	Mahahual	Mexico	17-Aug-05	18.667	-87.716	Cameron, Andy	10.1		11.1%			0	0.5
1922	PDC10	Pirates Del Caribe	Mexico	17-Aug-05	18.660	-87.709	Ponce-Taylor, Daniel	10.7		50.0%			0	0.5
1923	PDC10	Pirates Del Caribe	Mexico	17-Aug-05	18.660	-87.709	Ponce-Taylor, Daniel	10.1		22.2%			0	0.5
1338	Xc B 1	Xcalac	Mexico	19-Aug-05	18.236	-87.835	Jordan, Eric	2	1.1%	11.0%			0	0.5
1339	Xc B 2	Xcalac	Mexico	19-Aug-05	18.236	-87.835	Jordan, Eric	2	0.2%	3.2%			0	0.5
1340	Xc B 3	Xcalac	Mexico	19-Aug-05	18.236	-87.835	Jordan, Eric	1.5	1.6%	11.7%			0	0.5
1341	Xc B 4	Xcalac	Mexico	19-Aug-05	18.236	-87.835	Jordan, Eric	1.8	2.9%	15.3%			0	0.5
1342	Xc B 5	Xcalac	Mexico	19-Aug-05	18.236	-87.835	Jordan, Eric	1.6	0.5%	9.8%			0	0.5
1343	Xc B 6	Xcalac	Mexico	19-Aug-05	18.236	-87.835	Jordan, Eric	1.7	1.6%	28.8%			0	0.5
1344	Xc Fs 1	Xcalac	Mexico	19-Aug-05	18.251	-87.823	Jordan, Eric	11.5	0.5%	12.1%			0	0.5
1345	Xc Fs 2	Xcalac	Mexico	19-Aug-05	18.251	-87.823	Jordan, Eric	10	2.1%	34.2%			0	0.5
1346	Xc Fs 3	Xcalac	Mexico	19-Aug-05	18.251	-87.823	Jordan, Eric	10.6	3.0%	26.7%			0	0.5
1347	Xc Fs 4	Xcalac	Mexico	20-Aug-05	18.251	-87.823	Jordan, Eric	11.2	2.1%	20.3%			0	0.5
1348	Xc Fs 5	Xcalac	Mexico	20-Aug-05	18.251	-87.823	Jordan, Eric	10.6	1.6%	32.3%			0	0.5
1349	Xc Fs 6	Xcalac	Mexico	20-Aug-05	18.251	-87.823	Jordan, Eric	10	1.8%	16.9%			0	0.5
373	LL20	Mahahual	Mexico	22-Aug-05	18.707	-87.704	Cameron, Andy	14.95		33.3%			0	2.15
374	LL20	Mahahual	Mexico	22-Aug-05	18.707	-87.704	Cameron, Andy	14.6		11.8%			0	2.15
1350	Mh B 1	Mahahual	Mexico	22-Aug-05	18.688	-87.715	Jordan, Eric	2	1.4%	15.0%			0	0.5
1351	Mh B 2	Mahahual	Mexico	22-Aug-05	18.688	-87.715	Jordan, Eric	1.5	1.5%	28.7%			0	0.5
1352	Mh B 3	Mahahual	Mexico	22-Aug-05	18.688	-87.715	Jordan, Eric	1.5	1.6%	26.6%			0	0.5
1353	Mh B 4	Mahahual	Mexico	22-Aug-05	18.688	-87.715	Jordan, Eric	2	1.3%	48.4%			0	0.5
1354	Mh B 5	Mahahual	Mexico	22-Aug-05	18.688	-87.715	Jordan, Eric	1	2.1%	31.9%			0	0.5
1355	Mh B 6	Mahahual	Mexico	22-Aug-05	18.688	-87.715	Jordan, Eric	2	0.4%	38.1%			0	0.5
1924	LL20	Las Llamaradas	Mexico	22-Aug-05	18.704	-87.702	Ponce-Taylor, Daniel	14.95		50.0%			0	0.5
1925	LL20	Las Llamaradas	Mexico	22-Aug-05	18.704	-87.702	Ponce-Taylor, Daniel	14.6		37.5%			0	0.5
375	PDC20	Mahahual	Mexico	23-Aug-05	18.667	-87.714	Cameron, Andy	13.85		31.8%			0	0.5
376	SMDR10	Pez Maya, Reserva de la Biosfer	Mexico	23-Aug-05	19.967	-87.453	Cameron, Andy	9.6		33.3%			0	0
377	SMDR10	Pez Maya, Reserva de la Biosfer	Mexico	23-Aug-05	19.967	-87.453	Cameron, Andy	9.3		42.9%			0	0
378	SMDR10	Pez Maya, Reserva de la Biosfer	Mexico	23-Aug-05	19.967	-87.453	Cameron, Andy	8.8		55.6%			0	0
379	SMDR10	Pez Maya, Reserva de la Biosfer	Mexico	23-Aug-05	19.967	-87.453	Cameron, Andy	9		36.8%			0	0
1356	Mh Fd 1	Mahahual	Mexico	23-Aug-05	18.690	-87.709	Jordan, Eric	15	8.0%	32.0%			0	0.5
1357	Mh Fd 2	Mahahual	Mexico	23-Aug-05	18.690	-87.709	Jordan, Eric	15	16.6%	60.6%			0	0.5
1358	Mh Fd 3	Mahahual	Mexico	23-Aug-05	18.690	-87.709	Jordan, Eric	15	10.1%	34.2%			0	0.5
1359	Mh Fd 4	Mahahual	Mexico	23-Aug-05	18.690	-87.709	Jordan, Eric	16.5	7.8%	46.2%			0	0.5
1926	PDC20	Pirates Del Caribe	Mexico	23-Aug-05	18.660	-87.709	Ponce-Taylor, Daniel	13.85		46.7%			0	0.5
380	DB05	Mahahual	Mexico	24-Aug-05	18.724	-87.698	Cameron, Andy	4.6		9.1%			0	2.15
381	DB05	Mahahual	Mexico	24-Aug-05	18.724	-87.698	Cameron, Andy	4.15		8.3%			0	2.15
382	DB10	Mahahual	Mexico	24-Aug-05	18.724	-87.697	Cameron, Andy	8		4.5%			0	2.15
383	LL20	Mahahual	Mexico	24-Aug-05	18.707	-87.704	Cameron, Andy	16.2		28.6%			0	2.15
384	LL20	Mahahual	Mexico	24-Aug-05	18.707	-87.704	Cameron, Andy	15.85		42.4%			0	2.15
385	PJ05	Pez Maya, Reserva de la Biosfer	Mexico	24-Aug-05	20.015	-87.465	Cameron, Andy	6.1		27.3%			0	0
386	PJ05	Pez Maya, Reserva de la Biosfer	Mexico	24-Aug-05	20.015	-87.465	Cameron, Andy	6.1		100.0%			0	0

Observation Number	Reef or Site Name	Location	Country	Date (DD-MMM-YY)	Latitude (decimal degrees)	Longitude (decimal degrees)	Primary Contributor	Depth (m)	Cover bleached (%)	Colonies bleached (%)	Cover dead (%)	Colonies dead (%)	Observed DHW (°C-weeks)	Maximum DHW (°C-weeks)
387	SMDR20	Pez Maya, Reserva de la Biosfer	Mexico	24-Aug-05	19.968	-87.452	Cameron, Andy	18.9		75.0%			0	0
1360	Mh Fd 5	Mahahual	Mexico	24-Aug-05	18.690	-87.709	Jordan, Eric	14.2		34.3%			0	0.5
1361	Mh Fd 6	Mahahual	Mexico	24-Aug-05	18.690	-87.709	Jordan, Eric	16.1		53.8%			0	0.5
1362	Mh Fd 7	Mahahual	Mexico	24-Aug-05	18.690	-87.709	Jordan, Eric	14.8		46.0%			0	0.5
1363	Mh Fs 1	Mahahual	Mexico	24-Aug-05	18.690	-87.710	Jordan, Eric	10	2.9%	13.6%			0	0.5
1364	Mh Fs 2	Mahahual	Mexico	24-Aug-05	18.690	-87.710	Jordan, Eric	9.11	7.0%	35.4%			0	0.5
1365	Mh Fs 3	Mahahual	Mexico	24-Aug-05	18.690	-87.710	Jordan, Eric	9.22	3.4%	21.1%			0	0.5
1366	Mh Fs 4	Mahahual	Mexico	24-Aug-05	18.690	-87.710	Jordan, Eric	10	7.1%	29.9%			0	0.5
1367	Mh Fs 5	Mahahual	Mexico	24-Aug-05	18.690	-87.710	Jordan, Eric	9	8.3%	30.9%			0	0.5
1368	Mh Fs 6	Mahahual	Mexico	24-Aug-05	18.689	-87.710	Jordan, Eric	10	5.9%	27.6%			0	0.5
1927	DB05	Dolphin Bay	Mexico	24-Aug-05	18.721	-87.692	Ponce-Taylor, Daniel	4.6		14.3%			0	2.15
1928	DB05	Dolphin Bay	Mexico	24-Aug-05	18.721	-87.692	Ponce-Taylor, Daniel	4.15		10.0%			0	2.15
1929	DB10	Dolphin Bay	Mexico	24-Aug-05	18.721	-87.691	Ponce-Taylor, Daniel	8		10.0%			0	2.15
1930	LL20	Las Llamaradas	Mexico	24-Aug-05	18.704	-87.702	Ponce-Taylor, Daniel	16.2		36.4%			0	0.5
1931	LL20	Las Llamaradas	Mexico	24-Aug-05	18.704	-87.702	Ponce-Taylor, Daniel	15.85		61.9%			0	0.5
388	DB10	Mahahual	Mexico	25-Aug-05	18.724	-87.697	Cameron, Andy	9.9		25.0%			0	2.15
389	LE10	Mahahual	Mexico	25-Aug-05	18.720	-87.699	Cameron, Andy	8.65		25.0%			0	2.15
390	LE10	Mahahual	Mexico	25-Aug-05	18.720	-87.699	Cameron, Andy	9.8		29.2%			0	2.15
391	PJ10	Pez Maya, Reserva de la Biosfer	Mexico	25-Aug-05	20.017	-87.462	Cameron, Andy	9		60.0%			0	0
392	PJ10	Pez Maya, Reserva de la Biosfer	Mexico	25-Aug-05	20.017	-87.462	Cameron, Andy	9		75.0%			0	0
393	RB20	Mahahual	Mexico	25-Aug-05	18.681	-87.711	Cameron, Andy	18		10.3%			0	0.5
394	SMDR20	Pez Maya, Reserva de la Biosfer	Mexico	25-Aug-05	19.968	-87.452	Cameron, Andy	18.45		77.8%			0	0
395	SMDR20	Pez Maya, Reserva de la Biosfer	Mexico	25-Aug-05	19.968	-87.452	Cameron, Andy	17.85		100.0%			0	0
1369	Uv B 1	El Uvero	Mexico	25-Aug-05	18.946	-87.608	Jordan, Eric	2	2.0%	16.2%			0	0.5
1370	Uv B 2	El Uvero	Mexico	25-Aug-05	18.946	-87.608	Jordan, Eric	2	2.2%	17.8%			0	0.5
1371	Uv B 3	El Uvero	Mexico	25-Aug-05	18.946	-87.608	Jordan, Eric	2.5	1.7%	20.7%			0	0.5
1372	Uv Fs 1	El Uvero	Mexico	25-Aug-05	18.946	-87.604	Jordan, Eric	10	4.7%	59.4%			0	0.5
1373	Uv Fs 2	El Uvero	Mexico	25-Aug-05	18.946	-87.604	Jordan, Eric	10	8.1%	43.6%			0	0.5
1374	Uv Fs 3	El Uvero	Mexico	25-Aug-05	18.946	-87.604	Jordan, Eric	9.5	3.2%	40.4%			0	0.5
1932	DB10	Dolphin Bay	Mexico	25-Aug-05	18.721	-87.691	Ponce-Taylor, Daniel	9.9		47.1%			0	2.15
1933	LE10	Los Escalones	Mexico	25-Aug-05	18.718	-87.693	Ponce-Taylor, Daniel	8.65		42.1%			0	2.15
1934	LE10	Los Escalones	Mexico	25-Aug-05	18.718	-87.693	Ponce-Taylor, Daniel	9.8		43.8%			0	2.15
1935	RB20	Rio Bermejo	Mexico	25-Aug-05	18.675	-87.707	Ponce-Taylor, Daniel	18		22.2%			0	0.5
396	LL10	Mahahual	Mexico	26-Aug-05	18.707	-87.706	Cameron, Andy	10.1		37.0%			0	2.15
397	LL10	Mahahual	Mexico	26-Aug-05	18.707	-87.706	Cameron, Andy	9.45		20.0%			0	2.15
398	LL10	Mahahual	Mexico	26-Aug-05	18.707	-87.706	Cameron, Andy	10.4		42.1%			0	2.15
399	RB05	Mahahual	Mexico	26-Aug-05	18.681	-87.714	Cameron, Andy	5.8		33.3%			0	0.5
400	RB05	Mahahual	Mexico	26-Aug-05	18.681	-87.714	Cameron, Andy	5.35		25.0%			0	0.5
401	SMDR20	Pez Maya, Reserva de la Biosfer	Mexico	26-Aug-05	19.968	-87.452	Cameron, Andy	18.6		0.0%			0	0
402	SMDR20	Pez Maya, Reserva de la Biosfer	Mexico	26-Aug-05	19.968	-87.452	Cameron, Andy	17.85		100.0%			0	0
1375	Uv B 4	El Uvero	Mexico	26-Aug-05	18.946	-87.608	Jordan, Eric	2.5	1.4%	8.7%			0	0.5
1376	Uv B 5	El Uvero	Mexico	26-Aug-05	18.946	-87.608	Jordan, Eric	2	1.5%	10.3%			0	0.5
1377	Uv B 6	El Uvero	Mexico	26-Aug-05	18.946	-87.608	Jordan, Eric	2	1.2%	21.5%			0	0.5
1378	Uv Fs 4	El Uvero	Mexico	26-Aug-05	18.946	-87.604	Jordan, Eric	9.6	3.7%	42.5%			0	0.5
1379	Uv Fs 5	El Uvero	Mexico	26-Aug-05	18.946	-87.604	Jordan, Eric	9.9	4.5%	44.2%			0	0.5
1380	Uv Fs 6	El Uvero	Mexico	26-Aug-05	18.946	-87.604	Jordan, Eric	9.7	4.7%	35.3%			0	0.5
1381	Xc Fd 1	Xcalac	Mexico	26-Aug-05	18.288	-87.820	Jordan, Eric	15		52.0%			0	0.5
1382	Xc Fd 2	Xcalac	Mexico	26-Aug-05	18.287	-87.818	Jordan, Eric	15		53.6%			0	0.5
1383	Xc Fd 3	Xcalac	Mexico	26-Aug-05	18.287	-87.818	Jordan, Eric	5.4		46.6%			0	0.5
1936	LL10	Las Llamaradas	Mexico	26-Aug-05	18.704	-87.704	Ponce-Taylor, Daniel	10.1		62.5%			0	0.5
1937	LL10	Las Llamaradas	Mexico	26-Aug-05	18.704	-87.704	Ponce-Taylor, Daniel	9.45		28.6%			0	0.5
1938	LL10	Las Llamaradas	Mexico	26-Aug-05	18.704	-87.704	Ponce-Taylor, Daniel	10.4		58.3%			0	0.5
1939	RB05	Rio Bermejo	Mexico	26-Aug-05	18.675	-87.708	Ponce-Taylor, Daniel	5.8		40.0%			0	0.5
1940	RB05	Rio Bermejo	Mexico	26-Aug-05	18.675	-87.708	Ponce-Taylor, Daniel	5.35		33.3%			0	0.5
403	LE05	Mahahual	Mexico	27-Aug-05	18.720	-87.701	Cameron, Andy	5.05		31.6%			0	2.15
404	LE05	Mahahual	Mexico	27-Aug-05	18.720	-87.701	Cameron, Andy	4.45		21.4%			0	2.15
405	SMDR05	Pez Maya, Reserva de la Biosfer	Mexico	27-Aug-05	19.967	-87.461	Cameron, Andy	2.85		7.1%			0	0
406	SMDR05	Pez Maya, Reserva de la Biosfer	Mexico	27-Aug-05	19.967	-87.461	Cameron, Andy	2.25		16.7%			0	0

Observation Number	Reef or Site Name	Location	Country	Date (DD-MMM-YY)	Latitude (decimal degrees)	Longitude (decimal degrees)	Primary Contributor	Depth (m)	Cover bleached (%)	Colonies bleached (%)	Cover dead (%)	Colonies dead (%)	Observed DHW (°C-weeks)	Maximum DHW (°C-weeks)
1384	Xc Fd 4	Xcalac	Mexico	27-Aug-05	18.287	-87.818	Jordan, Eric	15		46.1%			0	0.5
1385	Xc Fd 5	Xcalac	Mexico	27-Aug-05	18.287	-87.818	Jordan, Eric	15.6		28.5%			0	0.5
1386	Xc Fd 6	Xcalac	Mexico	27-Aug-05	18.287	-87.818	Jordan, Eric	16.8		29.7%			0	0.5
1941	LE05	Los Escalones	Mexico	27-Aug-05	18.718	-87.701	Ponce-Taylor, Daniel	5.05		42.9%			0	2.15
1942	LE05	Los Escalones	Mexico	27-Aug-05	18.718	-87.701	Ponce-Taylor, Daniel	4.45		30.0%			0	2.15
407	PDC10	Mahahual	Mexico	28-Aug-05	18.667	-87.716	Cameron, Andy	10.4		16.7%			0	0.5
1943	PDC10	Pirates Del Caribe	Mexico	28-Aug-05	18.660	-87.709	Ponce-Taylor, Daniel	10.4		28.6%			0	0.5
408	LL05	Mahahual	Mexico	29-Aug-05	18.707	-87.707	Cameron, Andy	5.35		43.8%			0	2.15
409	PAY10	Mahahual	Mexico	29-Aug-05	18.634	-87.720	Cameron, Andy	10.25		16.7%			0	0.5
410	PAY10	Mahahual	Mexico	29-Aug-05	18.634	-87.720	Cameron, Andy	10.4		28.6%			0	0.5
411	PSJ20	Pez Maya, Reserva de la Biosfer	Mexico	29-Aug-05	19.880	-87.412	Cameron, Andy	20.1		55.6%			0	0
412	PX10	Pez Maya, Reserva de la Biosfer	Mexico	29-Aug-05	19.934	-87.434	Cameron, Andy	10.35		66.7%			0	0
413	PX10	Pez Maya, Reserva de la Biosfer	Mexico	29-Aug-05	19.934	-87.434	Cameron, Andy	10.35		66.7%			0	0
1944	LL05	Las Llamaradas	Mexico	29-Aug-05	18.704	-87.704	Ponce-Taylor, Daniel	5.35		53.8%			0	0.5
1945	PAY10	Paytocal	Mexico	29-Aug-05	18.634	-87.719	Ponce-Taylor, Daniel	10.25		25.0%			0	0.5
1946	PAY10	Paytocal	Mexico	29-Aug-05	18.634	-87.719	Ponce-Taylor, Daniel	10.4		40.0%			0	0.5
414	PDC05	Mahahual	Mexico	30-Aug-05	18.667	-87.717	Cameron, Andy	5.2		0.0%			0	0.5
415	PDC05	Mahahual	Mexico	30-Aug-05	18.667	-87.717	Cameron, Andy	5.05		28.6%			0	0.5
416	PDC05	Mahahual	Mexico	30-Aug-05	18.667	-87.717	Cameron, Andy	5.25		25.0%			0	0.5
417	PDC05	Mahahual	Mexico	30-Aug-05	18.667	-87.717	Cameron, Andy	5.25		100.0%			0	0.5
418	PSJ20	Pez Maya, Reserva de la Biosfer	Mexico	30-Aug-05	19.880	-87.412	Cameron, Andy	19.65		45.5%			0	0
419	PSJ20	Pez Maya, Reserva de la Biosfer	Mexico	30-Aug-05	19.880	-87.412	Cameron, Andy	19.65		14.3%			0	0
1947	PDC05	Pirates Del Caribe	Mexico	30-Aug-05	18.660	-87.717	Ponce-Taylor, Daniel	5.2		0.0%			0	0.5
1948	PDC05	Pirates Del Caribe	Mexico	30-Aug-05	18.660	-87.717	Ponce-Taylor, Daniel	5.05		28.6%			0	0.5
1949	PDC05	Pirates Del Caribe	Mexico	30-Aug-05	18.660	-87.717	Ponce-Taylor, Daniel	5.25		33.3%			0	0.5
1950	PDC05	Pirates Del Caribe	Mexico	30-Aug-05	18.660	-87.717	Ponce-Taylor, Daniel	5.25		100.0%			0	0.5
420	PAY05	Mahahual	Mexico	31-Aug-05	18.635	-87.722	Cameron, Andy	4.85		30.0%			0	0.5
421	PAY05	Mahahual	Mexico	31-Aug-05	18.635	-87.722	Cameron, Andy	4.8		40.0%			0	0.5
422	PAY10	Mahahual	Mexico	31-Aug-05	18.634	-87.720	Cameron, Andy	9.1		25.0%			0	0.5
423	PAY10	Mahahual	Mexico	31-Aug-05	18.634	-87.720	Cameron, Andy	9.1		42.9%			0	0.5
424	PAY20	Mahahual	Mexico	31-Aug-05	18.635	-87.719	Cameron, Andy	17.7		47.1%			0	0.5
425	PAY20	Mahahual	Mexico	31-Aug-05	18.635	-87.719	Cameron, Andy	19.6		25.0%			0	0.5
426	PC10	Pez Maya, Reserva de la Biosfer	Mexico	31-Aug-05	20.099	-87.462	Cameron, Andy	8.35		43.5%			0	0
427	PC10	Pez Maya, Reserva de la Biosfer	Mexico	31-Aug-05	20.099	-87.462	Cameron, Andy	8.2		22.2%			0	0
428	PC10	Pez Maya, Reserva de la Biosfer	Mexico	31-Aug-05	20.099	-87.462	Cameron, Andy	8.35		42.3%			0	0
429	PC10	Pez Maya, Reserva de la Biosfer	Mexico	31-Aug-05	20.099	-87.462	Cameron, Andy	8.5		63.2%			0	0
430	PSJ20	Pez Maya, Reserva de la Biosfer	Mexico	31-Aug-05	19.880	-87.412	Cameron, Andy	19.05		38.5%			0	0
431	RB05	Mahahual	Mexico	31-Aug-05	18.681	-87.714	Cameron, Andy	5.5		0.0%			0	0.5
432	RB05	Mahahual	Mexico	31-Aug-05	18.681	-87.714	Cameron, Andy	5.05		0.0%			0	0.5
1951	PAY05	Paytocal	Mexico	31-Aug-05	18.634	-87.720	Ponce-Taylor, Daniel	4.85		42.9%			0	0.5
1952	PAY05	Paytocal	Mexico	31-Aug-05	18.634	-87.720	Ponce-Taylor, Daniel	4.8		50.0%			0	0.5
1953	PAY10	Paytocal	Mexico	31-Aug-05	18.634	-87.719	Ponce-Taylor, Daniel	9.1		40.0%			0	0.5
1954	PAY10	Paytocal	Mexico	31-Aug-05	18.634	-87.719	Ponce-Taylor, Daniel	9.1		42.9%			0	0.5
1955	PAY20	Paytocal	Mexico	31-Aug-05	18.634	-87.718	Ponce-Taylor, Daniel	17.7		46.7%			0	0.5
1956	PAY20	Paytocal	Mexico	31-Aug-05	18.634	-87.718	Ponce-Taylor, Daniel	19.6		36.4%			0	0.5
1957	RB05	Rio Bermejo	Mexico	31-Aug-05	18.675	-87.708	Ponce-Taylor, Daniel	5.5		0.0%			0	0.5
1958	RB05	Rio Bermejo	Mexico	31-Aug-05	18.675	-87.708	Ponce-Taylor, Daniel	5.05		0.0%			0	0.5
433	PAY10	Mahahual	Mexico	1-Sep-05	18.634	-87.720	Cameron, Andy	9.85		50.0%			0.5	0.5
434	PAY20	Mahahual	Mexico	1-Sep-05	18.635	-87.719	Cameron, Andy	17.75		22.2%			0.5	0.5
435	PAY20	Mahahual	Mexico	1-Sep-05	18.635	-87.719	Cameron, Andy	17		5.3%			0.5	0.5
1959	PAY10	Paytocal	Mexico	1-Sep-05	18.634	-87.719	Ponce-Taylor, Daniel	9.85		57.1%			0.5	0.5
1960	PAY20	Paytocal	Mexico	1-Sep-05	18.634	-87.718	Ponce-Taylor, Daniel	17.75		33.3%			0.5	0.5
1961	PAY20	Paytocal	Mexico	1-Sep-05	18.634	-87.718	Ponce-Taylor, Daniel	17		12.5%			0.5	0.5
436	LL05	Mahahual	Mexico	2-Sep-05	18.707	-87.707	Cameron, Andy	5.2		23.5%			0	2.15
437	PDC05	Mahahual	Mexico	2-Sep-05	18.667	-87.717	Cameron, Andy	5.55		0.0%			0.5	0.5
438	PDC10	Mahahual	Mexico	2-Sep-05	18.667	-87.716	Cameron, Andy	10.2		8.3%			0.5	0.5
439	PDC10	Mahahual	Mexico	2-Sep-05	18.667	-87.716	Cameron, Andy	10		4.2%			0.5	0.5
440	PDC20	Mahahual	Mexico	2-Sep-05	18.667	-87.714	Cameron, Andy	14.15		2.9%			0.5	0.5

Observation Number	Reef or Site Name	Location	Country	Date (DD-MMM-YY)	Latitude (decimal degrees)	Longitude (decimal degrees)	Primary Contributor	Depth (m)	Cover bleached (%)	Colonies bleached (%)	Cover dead (%)	Colonies dead (%)	Observed DHW (°C-weeks)	Maximum DHW (°C-weeks)
441	PDC20	Mahahual	Mexico	2-Sep-05	18.667	-87.714	Cameron, Andy	15.15		25.0%			0.5	0.5
1962	LL05	Las Llamaradas	Mexico	2-Sep-05	18.704	-87.704	Ponce-Taylor, Daniel	5.2		33.3%			0.5	0.5
1963	PDC05	Pirates Del Caribe	Mexico	2-Sep-05	18.660	-87.717	Ponce-Taylor, Daniel	5.55		0.0%			0.5	0.5
1964	PDC10	Pirates Del Caribe	Mexico	2-Sep-05	18.660	-87.709	Ponce-Taylor, Daniel	10.2		37.5%			0.5	0.5
1965	PDC10	Pirates Del Caribe	Mexico	2-Sep-05	18.660	-87.709	Ponce-Taylor, Daniel	10		11.1%			0.5	0.5
1966	PDC20	Pirates Del Caribe	Mexico	2-Sep-05	18.660	-87.709	Ponce-Taylor, Daniel	14.15		10.0%			0.5	0.5
1967	PDC20	Pirates Del Caribe	Mexico	2-Sep-05	18.660	-87.709	Ponce-Taylor, Daniel	15.15		50.0%			0.5	0.5
442	PDC20	Mahahual	Mexico	4-Sep-05	18.667	-87.714	Cameron, Andy	16.65		33.3%			0.5	0.5
443	PDC20	Mahahual	Mexico	4-Sep-05	18.667	-87.714	Cameron, Andy	15.75		29.6%			0.5	0.5
444	PL05	Pez Maya, Reserva de la Biosfer	Mexico	4-Sep-05	20.050	-87.470	Cameron, Andy	3.7		23.1%			0	0
445	PL05	Pez Maya, Reserva de la Biosfer	Mexico	4-Sep-05	20.050	-87.470	Cameron, Andy	3.55		60.0%			0	0
446	PL05	Pez Maya, Reserva de la Biosfer	Mexico	4-Sep-05	20.050	-87.470	Cameron, Andy	3.4		52.4%			0	0
447	PL05	Pez Maya, Reserva de la Biosfer	Mexico	4-Sep-05	20.050	-87.470	Cameron, Andy	3.4		50.0%			0	0
448	PL05	Pez Maya, Reserva de la Biosfer	Mexico	4-Sep-05	20.050	-87.470	Cameron, Andy	3.85		42.9%			0	0
449	PX10	Pez Maya, Reserva de la Biosfer	Mexico	4-Sep-05	19.934	-87.434	Cameron, Andy	12.8		80.0%			0	0
450	PX20	Pez Maya, Reserva de la Biosfer	Mexico	4-Sep-05	19.933	-87.432	Cameron, Andy	18.6		60.0%			0	0
451	PX20	Pez Maya, Reserva de la Biosfer	Mexico	4-Sep-05	19.933	-87.432	Cameron, Andy	18.9		69.2%			0	0
452	RB20	Mahahual	Mexico	4-Sep-05	18.681	-87.711	Cameron, Andy	14.35		32.3%			0.5	0.5
1968	PDC20	Pirates Del Caribe	Mexico	4-Sep-05	18.660	-87.709	Ponce-Taylor, Daniel	16.65		40.0%			0.5	0.5
1969	PDC20	Pirates Del Caribe	Mexico	4-Sep-05	18.660	-87.709	Ponce-Taylor, Daniel	15.75		43.8%			0.5	0.5
1970	RB20	Rio Bermejo	Mexico	4-Sep-05	18.675	-87.707	Ponce-Taylor, Daniel	14.35		55.6%			0.5	0.5
453	LL20	Mahahual	Mexico	5-Sep-05	18.707	-87.704	Cameron, Andy	14.8		45.8%			0	2.15
454	PL20	Pez Maya, Reserva de la Biosfer	Mexico	5-Sep-05	20.051	-87.463	Cameron, Andy	15.25		11.5%			0	0
455	PX20	Pez Maya, Reserva de la Biosfer	Mexico	5-Sep-05	19.933	-87.432	Cameron, Andy	18.3		54.5%			0	0
456	PX20	Pez Maya, Reserva de la Biosfer	Mexico	5-Sep-05	19.933	-87.432	Cameron, Andy	18.75		30.0%			0	0
457	PX20	Pez Maya, Reserva de la Biosfer	Mexico	5-Sep-05	19.933	-87.432	Cameron, Andy	18.45		27.3%			0	0
1971	LL20	Las Llamaradas	Mexico	5-Sep-05	18.704	-87.702	Ponce-Taylor, Daniel	14.8		64.3%			0.5	0.5
458	PL20	Pez Maya, Reserva de la Biosfer	Mexico	6-Sep-05	20.051	-87.463	Cameron, Andy	13.1		45.5%			0	0
459	PC10	Pez Maya, Reserva de la Biosfer	Mexico	7-Sep-05	20.099	-87.462	Cameron, Andy	9.6		20.0%			0	0
460	PL20	Pez Maya, Reserva de la Biosfer	Mexico	7-Sep-05	20.051	-87.463	Cameron, Andy	14		24.3%			0	0
461	PL20	Pez Maya, Reserva de la Biosfer	Mexico	7-Sep-05	20.051	-87.463	Cameron, Andy	13.4		57.1%			0	0
462	PL20	Pez Maya, Reserva de la Biosfer	Mexico	7-Sep-05	20.051	-87.463	Cameron, Andy	13.4		100.0%			0	0
463	PX10	Pez Maya, Reserva de la Biosfer	Mexico	7-Sep-05	19.934	-87.434	Cameron, Andy	11.9		27.3%			0	0
464	PX10	Pez Maya, Reserva de la Biosfer	Mexico	7-Sep-05	19.934	-87.434	Cameron, Andy	12.5		35.7%			0	0
1387	Ya Fd 1	Akumal	Mexico	7-Sep-05	20.409	-87.298	Jordan, Eric	12.5	9.1%	59.1%			0	0
1388	Ya Fd 2	Akumal	Mexico	7-Sep-05	20.408	-87.298	Jordan, Eric	12.9	4.7%	49.2%			0	0
1389	Ya Fs 1	Akumal	Mexico	7-Sep-05	20.406	-87.302	Jordan, Eric	6	7.1%	63.4%			0	0
1390	Ya Fs 2	Akumal	Mexico	7-Sep-05	20.406	-87.302	Jordan, Eric	6	3.7%	53.1%			0	0
1391	Ya Fs 3	Akumal	Mexico	7-Sep-05	20.406	-87.302	Jordan, Eric	5.8	12.1%	75.6%			0	0
465	PSJ20	Pez Maya, Reserva de la Biosfer	Mexico	8-Sep-05	19.880	-87.412	Cameron, Andy	21		72.7%			0	0
466	SMDR05	Pez Maya, Reserva de la Biosfer	Mexico	8-Sep-05	19.967	-87.461	Cameron, Andy	2.25		0.0%			0	0
467	SMDR05	Pez Maya, Reserva de la Biosfer	Mexico	8-Sep-05	19.967	-87.461	Cameron, Andy	2.7		25.0%			0	0
468	SMDR05	Pez Maya, Reserva de la Biosfer	Mexico	8-Sep-05	19.967	-87.461	Cameron, Andy	2.25		50.0%			0	0
1392	Ya Fd 3	Akumal	Mexico	8-Sep-05	20.408	-87.298	Jordan, Eric	13	9.7%	60.9%			0	0
1393	Ya Fd 4	Akumal	Mexico	8-Sep-05	20.408	-87.298	Jordan, Eric	13.7	3.7%	46.6%			0	0
1394	Ya Fs 4	Akumal	Mexico	8-Sep-05	20.406	-87.302	Jordan, Eric	7.4	7.9%	48.1%			0	0
1395	Ya Fs 5	Akumal	Mexico	8-Sep-05	20.406	-87.302	Jordan, Eric	4.4	3.5%	51.7%			0	0
1396	Ya Fs 6	Akumal	Mexico	8-Sep-05	20.406	-87.302	Jordan, Eric	5.3	4.6%	37.8%			0	0
1397	Ya Fd 5	Akumal	Mexico	9-Sep-05	20.408	-87.298	Jordan, Eric	12.5	10.9%	57.4%			0	0
1398	Ya Fd 6	Akumal	Mexico	9-Sep-05	20.408	-87.298	Jordan, Eric	13	3.2%	55.0%			0	0
1399	PM Bo B 1	Puerto Morelos	Mexico	18-Sep-05	20.874	-86.851	Jordan, Eric	5	7.4%	80.0%	0.0%	0.0%	0	0
1400	PM Ja Fs 1	Puerto Morelos	Mexico	18-Sep-05	20.832	-86.874	Jordan, Eric	6	2.9%	73.4%	0.0%	0.0%	0	0
1401	PM Ja Fs 2	Puerto Morelos	Mexico	18-Sep-05	20.832	-86.874	Jordan, Eric	6.5	6.6%	70.5%	0.0%	0.0%	0	0
1402	PM CN B 1	Puerto Morelos	Mexico	19-Sep-05	20.863	-86.859	Jordan, Eric	2	0.1%	28.0%	0.0%	0.0%	0	0
1403	PM CN B 2	Puerto Morelos	Mexico	19-Sep-05	20.862	-86.860	Jordan, Eric	2	0.1%	33.1%	0.0%	0.0%	0	0
1404	PM CN B 3	Puerto Morelos	Mexico	19-Sep-05	20.862	-86.860	Jordan, Eric	2	0.1%	24.4%	0.0%	0.0%	0	0
1405	PM CN B 4	Puerto Morelos	Mexico	19-Sep-05	20.862	-86.859	Jordan, Eric	2	0.4%	33.3%	0.0%	0.0%	0	0
1406	PM CN B 5	Puerto Morelos	Mexico	19-Sep-05	20.862	-86.860	Jordan, Eric	2	0.1%	27.0%	0.0%	0.0%	0	0

Observation Number	Reef or Site Name	Location	Country	Date (DD-MMM-YY)	Latitude (decimal degrees)	Longitude (decimal degrees)	Primary Contributor	Depth (m)	Cover bleached (%)	Colonies bleached (%)	Cover dead (%)	Colonies dead (%)	Observed DHW (°C-weeks)	Maximum DHW (°C-weeks)
1407	PM CN B 6	Puerto Morelos	Mexico	19-Sep-05	20.862	-86.859	Jordan, Eric	2	0.3%	31.1%	0.0%	0.0%	0	0
1408	PM Ja Fs 3	Puerto Morelos	Mexico	20-Sep-05	20.832	-86.874	Jordan, Eric	6.5	6.9%	59.0%	0.0%	0.0%	0	0
1409	PM Ja Fs 4	Puerto Morelos	Mexico	20-Sep-05	20.832	-86.874	Jordan, Eric	6	20.0%	55.3%	0.0%	0.0%	0	0
1410	PM Ja Fs 5	Puerto Morelos	Mexico	26-Sep-05	20.832	-86.874	Jordan, Eric	6	4.9%	48.6%	0.0%	0.0%	0	0
1411	PM Ja Fs 6	Puerto Morelos	Mexico	26-Sep-05	20.832	-86.874	Jordan, Eric	6	10.1%	60.2%	0.0%	0.0%	0	0
1412	PM Ce B 1	Puerto Morelos	Mexico	27-Sep-05	20.856	-86.860	Jordan, Eric	2	3.7%	44.2%	0.0%	0.0%	0	0
1413	PM Ce B 2	Puerto Morelos	Mexico	27-Sep-05	20.856	-86.861	Jordan, Eric	2	3.6%	42.0%	0.0%	0.0%	0	0
1414	PM Ce B 3	Puerto Morelos	Mexico	27-Sep-05	20.856	-86.861	Jordan, Eric	2	5.4%	61.8%	0.0%	0.0%	0	0
1415	PM Ce B 4	Puerto Morelos	Mexico	28-Sep-05	20.856	-86.860	Jordan, Eric	2	4.7%	53.6%	0.0%	0.0%	0	0
1416	PM Ce B 5	Puerto Morelos	Mexico	28-Sep-05	20.856	-86.861	Jordan, Eric	2	5.0%	54.3%	0.0%	0.0%	0	0
1417	PM Ce B 6	Puerto Morelos	Mexico	28-Sep-05	20.856	-86.860	Jordan, Eric	2	9.0%	52.4%	0.0%	0.0%	0	0
1418	PM Bo B 2	Puerto Morelos	Mexico	29-Sep-05	20.874	-86.851	Jordan, Eric	5	2.5%	59.9%	0.0%	0.0%	0	0
1419	PM Bo B 3	Puerto Morelos	Mexico	29-Sep-05	20.873	-86.851	Jordan, Eric	5	2.2%	61.9%	0.0%	0.0%	0	0
1420	PM Bo B 4	Puerto Morelos	Mexico	29-Sep-05	20.873	-86.851	Jordan, Eric	5	5.2%	74.1%	0.0%	0.0%	0	0
1421	PM Bo B 5	Puerto Morelos	Mexico	3-Oct-05	20.873	-86.851	Jordan, Eric	5	2.8%	57.5%	0.0%	0.0%	0	0
1422	PM Bo B 6	Puerto Morelos	Mexico	3-Oct-05	20.873	-86.851	Jordan, Eric	5	4.7%	53.3%	0.0%	0.0%	0	0
1423	PM Pi B 1	Puerto Morelos	Mexico	5-Oct-05	20.882	-86.848	Jordan, Eric	5	7.3%	70.9%	0.0%	0.0%	0	0
1424	PM Pi B 2	Puerto Morelos	Mexico	5-Oct-05	20.882	-86.848	Jordan, Eric	5	2.5%	69.5%	0.0%	0.0%	0	0
1425	PM Pi B 3	Puerto Morelos	Mexico	5-Oct-05	20.882	-86.848	Jordan, Eric	5	2.3%	48.4%	0.0%	0.0%	0	0
1426	PM Pi B 4	Puerto Morelos	Mexico	6-Oct-05	20.882	-86.848	Jordan, Eric	5	2.3%	59.9%	0.0%	0.0%	0	0
1427	PM Pi B 5	Puerto Morelos	Mexico	6-Oct-05	20.882	-86.848	Jordan, Eric	5	3.6%	56.7%	0.0%	0.0%	0	0
1428	PM Pi B 6	Puerto Morelos	Mexico	6-Oct-05	20.882	-86.848	Jordan, Eric	5	3.1%	45.4%	0.0%	0.0%	0	0
7	Punta Sur bajo	Cozumel	Mexico	26-Oct-05	20.298	-87.020	Alvarez, Lorenzo	4.5	50.0%				0	0
469	LL10	Mahahual	Mexico	10-Nov-05	18.707	-87.706	Cameron, Andy	9.6		55.2%			2.15	2.15
470	LL10	Mahahual	Mexico	10-Nov-05	18.707	-87.706	Cameron, Andy	9.6		50.0%			2.15	2.15
1972	LL10	Las Llamaradas	Mexico	10-Nov-05	18.704	-87.704	Ponce-Taylor, Daniel	9.6		61.9%			0.5	0.5
1973	LL10	Las Llamaradas	Mexico	10-Nov-05	18.704	-87.704	Ponce-Taylor, Daniel	9.6		64.7%			0.5	0.5
471	LL10	Mahahual	Mexico	13-Nov-05	18.707	-87.706	Cameron, Andy	9.8		48.5%			2.15	2.15
472	LL10	Mahahual	Mexico	13-Nov-05	18.707	-87.706	Cameron, Andy	8.3		21.7%			2.15	2.15
473	LL10	Mahahual	Mexico	13-Nov-05	18.707	-87.706	Cameron, Andy	8.8		28.0%			2.15	2.15
1974	LL10	Las Llamaradas	Mexico	13-Nov-05	18.704	-87.704	Ponce-Taylor, Daniel	9.8		61.9%			0.5	0.5
1975	LL10	Las Llamaradas	Mexico	13-Nov-05	18.704	-87.704	Ponce-Taylor, Daniel	8.3		35.7%			0.5	0.5
1976	LL10	Las Llamaradas	Mexico	13-Nov-05	18.704	-87.704	Ponce-Taylor, Daniel	8.8		41.2%			0.5	0.5
8	Paso del Cedral	Cozumel	Mexico	14-Nov-05	20.374	-87.029	Alvarez, Lorenzo	13.5		16.1%		0.0%	0	0
474	RB10	Mahahual	Mexico	14-Nov-05	18.681	-87.713	Cameron, Andy	11.3		71.9%			0.5	0.5
475	RB10	Mahahual	Mexico	14-Nov-05	18.681	-87.713	Cameron, Andy	11.35		34.4%			0.5	0.5
1977	RB10	Rio Bermejo	Mexico	14-Nov-05	18.675	-87.708	Ponce-Taylor, Daniel	11.3		76.0%			0.5	0.5
1978	RB10	Rio Bermejo	Mexico	14-Nov-05	18.675	-87.708	Ponce-Taylor, Daniel	11.35		57.9%			0.5	0.5
9	Chancanaab	Cozumel	Mexico	15-Nov-05	20.441	-87.002	Alvarez, Lorenzo	11.36		21.6%		0.0%	0	0
10	Colombia	Cozumel	Mexico	16-Nov-05	20.325	-87.027	Alvarez, Lorenzo	9.82		23.6%		0.0%	0	0
476	RB10	Mahahual	Mexico	16-Nov-05	18.681	-87.713	Cameron, Andy	11.3		24.2%			0.5	0.5
477	RB10	Mahahual	Mexico	16-Nov-05	18.681	-87.713	Cameron, Andy	9.5		16.7%			0.5	0.5
478	RB10	Mahahual	Mexico	16-Nov-05	18.681	-87.713	Cameron, Andy	8.55		50.0%			0.5	0.5
1979	RB10	Rio Bermejo	Mexico	16-Nov-05	18.675	-87.708	Ponce-Taylor, Daniel	11.3		50.0%			0.5	0.5
1980	RB10	Rio Bermejo	Mexico	16-Nov-05	18.675	-87.708	Ponce-Taylor, Daniel	9.5		41.2%			0.5	0.5
1981	RB10	Rio Bermejo	Mexico	16-Nov-05	18.675	-87.708	Ponce-Taylor, Daniel	8.55		70.0%			0.5	0.5
479	LE10	Mahahual	Mexico	17-Nov-05	18.720	-87.699	Cameron, Andy	9.6		16.7%			2.15	2.15
1982	LE10	Los Escalones	Mexico	17-Nov-05	18.718	-87.693	Ponce-Taylor, Daniel	9.6		36.8%			2.15	2.15
11	Palancar Jardines	Cozumel	Mexico	18-Nov-05	20.337	-87.027	Alvarez, Lorenzo	10	15.0%				0	0
480	LL05	Mahahual	Mexico	20-Nov-05	18.707	-87.707	Cameron, Andy	5.2		66.7%			2.15	2.15
481	LL05	Mahahual	Mexico	20-Nov-05	18.707	-87.707	Cameron, Andy	5.55		70.0%			2.15	2.15
1983	LL05	Las Llamaradas	Mexico	20-Nov-05	18.704	-87.704	Ponce-Taylor, Daniel	5.2		66.7%			0.5	0.5
1984	LL05	Las Llamaradas	Mexico	20-Nov-05	18.704	-87.704	Ponce-Taylor, Daniel	5.55		70.0%			0.5	0.5
12	Dalila	Cozumel	Mexico	21-Nov-05	20.349	-87.029	Alvarez, Lorenzo	11.5		5.6%		0.0%	0	0
13	Yucab	Cozumel	Mexico	21-Nov-05	20.421	-87.017	Alvarez, Lorenzo	12.65		7.4%		0.0%	0	0
482	LE10	Mahahual	Mexico	21-Nov-05	18.720	-87.699	Cameron, Andy	9.45		58.3%			2.15	2.15
483	LL05	Mahahual	Mexico	21-Nov-05	18.707	-87.707	Cameron, Andy	6.55		41.2%			2.15	2.15
484	LL05	Mahahual	Mexico	21-Nov-05	18.707	-87.707	Cameron, Andy	7.45		37.5%			2.15	2.15

Observation Number	Reef or Site Name	Location	Country	Date (DD-MMM-YY)	Latitude (decimal degrees)	Longitude (decimal degrees)	Primary Contributor	Depth (m)	Cover bleached (%)	Colonies bleached (%)	Cover dead (%)	Colonies dead (%)	Observed DHW (°C-weeks)	Maximum DHW (°C-weeks)
485	LL05	Mahahual	Mexico	21-Nov-05	18.707	-87.707	Cameron, Andy	6.4		23.1%			2.15	2.15
1985	LE10	Los Escalones	Mexico	21-Nov-05	18.718	-87.693	Ponce-Taylor, Daniel	9.45		72.2%			2.15	2.15
1986	LL05	Las Llamaradas	Mexico	21-Nov-05	18.704	-87.704	Ponce-Taylor, Daniel	6.55		58.3%			0.5	0.5
1987	LL05	Las Llamaradas	Mexico	21-Nov-05	18.704	-87.704	Ponce-Taylor, Daniel	7.45		50.0%			0.5	0.5
1988	LL05	Las Llamaradas	Mexico	21-Nov-05	18.704	-87.704	Ponce-Taylor, Daniel	6.4		35.7%			0.5	0.5
486	LE10	Mahahual	Mexico	22-Nov-05	18.720	-87.699	Cameron, Andy	10.5		16.7%			2.15	2.15
487	LE10	Mahahual	Mexico	22-Nov-05	18.720	-87.699	Cameron, Andy	10.25		16.7%			2.15	2.15
1989	LE10	Los Escalones	Mexico	22-Nov-05	18.718	-87.693	Ponce-Taylor, Daniel	10.5		25.0%			2.15	2.15
1990	LE10	Los Escalones	Mexico	22-Nov-05	18.718	-87.693	Ponce-Taylor, Daniel	10.25		35.7%			2.15	2.15
14	Paraiso	Cozumel	Mexico	23-Nov-05	20.469	-86.983	Alvarez, Lorenzo	9.91		22.2%		0.0%	0	0
15	Villa Blanca	Cozumel	Mexico	23-Nov-05	20.488	-86.967	Alvarez, Lorenzo	4.8		25.6%		5.1%	0	0
488	DB10	Mahahual	Mexico	23-Nov-05	18.724	-87.697	Cameron, Andy	8.2		53.3%			2.15	2.15
489	DB10	Mahahual	Mexico	23-Nov-05	18.724	-87.697	Cameron, Andy	8.45		15.0%			2.15	2.15
490	PJ10	Pez Maya, Reserva de la Biosfer	Mexico	23-Nov-05	20.017	-87.462	Cameron, Andy	10.1		63.6%			0	0
1991	DB10	Dolphin Bay	Mexico	23-Nov-05	18.721	-87.691	Ponce-Taylor, Daniel	8.2		57.1%			2.15	2.15
1992	DB10	Dolphin Bay	Mexico	23-Nov-05	18.721	-87.691	Ponce-Taylor, Daniel	8.45		37.5%			2.15	2.15
16	Islote	Cozumel	Mexico	24-Nov-05	20.270	-86.975	Alvarez, Lorenzo	13.4		21.1%		3.5%	0	0
17	Punta Chiqueros	Cozumel	Mexico	24-Nov-05	20.352	-86.883	Alvarez, Lorenzo	21.98		25.0%		10.0%	0	0
491	DB10	Mahahual	Mexico	24-Nov-05	18.724	-87.697	Cameron, Andy	8.3		75.0%			2.15	2.15
492	DB10	Mahahual	Mexico	24-Nov-05	18.724	-87.697	Cameron, Andy	8.4		74.1%			2.15	2.15
493	PJ05	Pez Maya, Reserva de la Biosfer	Mexico	24-Nov-05	20.015	-87.465	Cameron, Andy	4.15		0.0%			0	0
494	PJ05	Pez Maya, Reserva de la Biosfer	Mexico	24-Nov-05	20.015	-87.465	Cameron, Andy	4.6		30.8%			0	0
495	PJ05	Pez Maya, Reserva de la Biosfer	Mexico	24-Nov-05	20.015	-87.465	Cameron, Andy	4.6		58.3%			0	0
496	PJ10	Pez Maya, Reserva de la Biosfer	Mexico	24-Nov-05	20.017	-87.462	Cameron, Andy	11.75		58.8%			0	0
497	PJ10	Pez Maya, Reserva de la Biosfer	Mexico	24-Nov-05	20.017	-87.462	Cameron, Andy	10.1		42.1%			0	0
1993	DB10	Dolphin Bay	Mexico	24-Nov-05	18.721	-87.691	Ponce-Taylor, Daniel	8.3		85.7%			2.15	2.15
1994	DB10	Dolphin Bay	Mexico	24-Nov-05	18.721	-87.691	Ponce-Taylor, Daniel	8.4		83.3%			2.15	2.15
498	PDC10	Mahahual	Mexico	29-Nov-05	18.667	-87.716	Cameron, Andy	10.1		35.5%			0	0.5
499	PDC10	Mahahual	Mexico	29-Nov-05	18.667	-87.716	Cameron, Andy	10.55		33.3%			0	0.5
500	SMDR10	Pez Maya, Reserva de la Biosfer	Mexico	29-Nov-05	19.967	-87.453	Cameron, Andy	10.4		52.9%			0	0
501	SMDR10	Pez Maya, Reserva de la Biosfer	Mexico	29-Nov-05	19.967	-87.453	Cameron, Andy	9.95		61.5%			0	0
1995	PDC10	Pirates Del Caribe	Mexico	29-Nov-05	18.660	-87.709	Ponce-Taylor, Daniel	10.1		56.3%			0	0.5
1996	PDC10	Pirates Del Caribe	Mexico	29-Nov-05	18.660	-87.709	Ponce-Taylor, Daniel	10.55		52.6%			0	0.5
502	DB05	Mahahual	Mexico	30-Nov-05	18.724	-87.698	Cameron, Andy	4.3		22.2%			2.15	2.15
503	DB05	Mahahual	Mexico	30-Nov-05	18.724	-87.698	Cameron, Andy	4		7.7%			2.15	2.15
504	DB05	Mahahual	Mexico	30-Nov-05	18.724	-87.698	Cameron, Andy	4.65		5.6%			2.15	2.15
505	DB05	Mahahual	Mexico	30-Nov-05	18.724	-87.698	Cameron, Andy	4.65		25.0%			2.15	2.15
506	PDC10	Mahahual	Mexico	30-Nov-05	18.667	-87.716	Cameron, Andy	10.4		88.5%			0	0.5
507	PDC10	Mahahual	Mexico	30-Nov-05	18.667	-87.716	Cameron, Andy	10.35		75.0%			0	0.5
508	PDC10	Mahahual	Mexico	30-Nov-05	18.667	-87.716	Cameron, Andy	10.2		63.0%			0	0.5
509	SMDR10	Pez Maya, Reserva de la Biosfer	Mexico	30-Nov-05	19.967	-87.453	Cameron, Andy	8.8		58.3%			0	0
1997	DB05	Dolphin Bay	Mexico	30-Nov-05	18.721	-87.692	Ponce-Taylor, Daniel	4.3		40.0%			2.15	2.15
1998	DB05	Dolphin Bay	Mexico	30-Nov-05	18.721	-87.692	Ponce-Taylor, Daniel	4		14.3%			2.15	2.15
1999	DB05	Dolphin Bay	Mexico	30-Nov-05	18.721	-87.692	Ponce-Taylor, Daniel	4.65		11.1%			2.15	2.15
2000	DB05	Dolphin Bay	Mexico	30-Nov-05	18.721	-87.692	Ponce-Taylor, Daniel	4.65		50.0%			2.15	2.15
2001	PDC10	Pirates Del Caribe	Mexico	30-Nov-05	18.660	-87.709	Ponce-Taylor, Daniel	10.4		87.0%			0	0.5
2002	PDC10	Pirates Del Caribe	Mexico	30-Nov-05	18.660	-87.709	Ponce-Taylor, Daniel	10.35		81.0%			0	0.5
2003	PDC10	Pirates Del Caribe	Mexico	30-Nov-05	18.660	-87.709	Ponce-Taylor, Daniel	10.2		68.4%			0	0.5
510	DB05	Mahahual	Mexico	1-Dec-05	18.724	-87.698	Cameron, Andy	4.4		76.5%			2.15	2.15
511	DB10	Mahahual	Mexico	1-Dec-05	18.724	-87.697	Cameron, Andy	8.35		15.2%			2.15	2.15
512	PL05	Pez Maya, Reserva de la Biosfer	Mexico	1-Dec-05	20.050	-87.470	Cameron, Andy	4.3		50.0%			0	0
513	PL10	Pez Maya, Reserva de la Biosfer	Mexico	1-Dec-05	20.052	-87.466	Cameron, Andy	6.1		20.0%			0	0
514	PL10	Pez Maya, Reserva de la Biosfer	Mexico	1-Dec-05	20.052	-87.466	Cameron, Andy	6.55		14.3%			0	0
515	PL10	Pez Maya, Reserva de la Biosfer	Mexico	1-Dec-05	20.052	-87.466	Cameron, Andy	6.55		75.0%			0	0
516	PL10	Pez Maya, Reserva de la Biosfer	Mexico	1-Dec-05	20.052	-87.466	Cameron, Andy	5.05		58.3%			0	0
2004	DB05	Dolphin Bay	Mexico	1-Dec-05	18.721	-87.692	Ponce-Taylor, Daniel	4.4		78.6%			2.15	2.15
2005	DB10	Dolphin Bay	Mexico	1-Dec-05	18.721	-87.691	Ponce-Taylor, Daniel	8.35		33.3%			2.15	2.15
517	LE10	Mahahual	Mexico	2-Dec-05	18.720	-87.699	Cameron, Andy	8.55		44.1%			2.15	2.15

Observation Number	Reef or Site Name	Location	Country	Date (DD-MMM-YY)	Latitude (decimal degrees)	Longitude (decimal degrees)	Primary Contributor	Depth (m)	Cover bleached (%)	Colonies bleached (%)	Cover dead (%)	Colonies dead (%)	Observed DHW (°C-weeks)	Maximum DHW (°C-weeks)
2006	LE10	Los Escalones	Mexico	2-Dec-05	18.718	-87.693	Ponce-Taylor, Daniel	8.55		60.9%			2.15	2.15
18	Castillo	Cozumel	Mexico	3-Dec-05	20.510	-86.762	Alvarez, Lorenzo	6.14		9.8%		2.0%	0	0
19	Microatolones	Cozumel	Mexico	3-Dec-05	20.505	-86.758	Alvarez, Lorenzo	8.67		14.0%		0.0%	0	0
518	LL20	Mahahual	Mexico	3-Dec-05	18.707	-87.704	Cameron, Andy	14.95		45.5%			2.15	2.15
519	LL20	Mahahual	Mexico	3-Dec-05	18.707	-87.704	Cameron, Andy	15.25		22.2%			2.15	2.15
520	PL10	Pez Maya, Reserva de la Biosfer	Mexico	3-Dec-05	20.052	-87.466	Cameron, Andy	6.55		41.7%			0	0
521	PL20	Pez Maya, Reserva de la Biosfer	Mexico	3-Dec-05	20.051	-87.463	Cameron, Andy	13.1		80.0%			0	0
2007	LL20	Las Llamaradas	Mexico	3-Dec-05	18.704	-87.702	Ponce-Taylor, Daniel	14.95		66.7%			0	0.5
2008	LL20	Las Llamaradas	Mexico	3-Dec-05	18.704	-87.702	Ponce-Taylor, Daniel	15.25		52.6%			0	0.5
20	Hanan	Cozumel	Mexico	5-Dec-05	20.499	-86.761	Alvarez, Lorenzo	8.2		24.2%		0.0%	0	0
522	LL20	Mahahual	Mexico	5-Dec-05	18.707	-87.704	Cameron, Andy	13.4		60.0%			2.15	2.15
523	LL20	Mahahual	Mexico	5-Dec-05	18.707	-87.704	Cameron, Andy	15.4		56.8%			2.15	2.15
524	LL20	Mahahual	Mexico	5-Dec-05	18.707	-87.704	Cameron, Andy	15.25		32.4%			2.15	2.15
525	PC10	Pez Maya, Reserva de la Biosfer	Mexico	5-Dec-05	20.099	-87.462	Cameron, Andy	9.75		20.0%			0	0
526	PC10	Pez Maya, Reserva de la Biosfer	Mexico	5-Dec-05	20.099	-87.462	Cameron, Andy	8.35		35.3%			0	0
527	PC10	Pez Maya, Reserva de la Biosfer	Mexico	5-Dec-05	20.099	-87.462	Cameron, Andy	8.05		63.6%			0	0
528	PC10	Pez Maya, Reserva de la Biosfer	Mexico	5-Dec-05	20.099	-87.462	Cameron, Andy	8.05		53.8%			0	0
529	PC10	Pez Maya, Reserva de la Biosfer	Mexico	5-Dec-05	20.099	-87.462	Cameron, Andy	8.05		85.7%			0	0
530	PL20	Pez Maya, Reserva de la Biosfer	Mexico	5-Dec-05	20.051	-87.463	Cameron, Andy	14.3		50.0%			0	0
531	RB05	Mahahual	Mexico	5-Dec-05	18.681	-87.714	Cameron, Andy	5.8		0.0%			0	0.5
532	RB05	Mahahual	Mexico	5-Dec-05	18.681	-87.714	Cameron, Andy	5.8		0.0%			0	0.5
533	RB05	Mahahual	Mexico	5-Dec-05	18.681	-87.714	Cameron, Andy	6.4		16.7%			0	0.5
2009	LL20	Las Llamaradas	Mexico	5-Dec-05	18.704	-87.702	Ponce-Taylor, Daniel	13.4		66.7%			0	0.5
2010	LL20	Las Llamaradas	Mexico	5-Dec-05	18.704	-87.702	Ponce-Taylor, Daniel	15.4		72.4%			0	0.5
2011	LL20	Las Llamaradas	Mexico	5-Dec-05	18.704	-87.702	Ponce-Taylor, Daniel	15.25		64.7%			0	0.5
2012	RB05	Rio Bermejo	Mexico	5-Dec-05	18.675	-87.708	Ponce-Taylor, Daniel	5.8		0.0%			0	0.5
2013	RB05	Rio Bermejo	Mexico	5-Dec-05	18.675	-87.708	Ponce-Taylor, Daniel	5.8		0.0%			0	0.5
2014	RB05	Rio Bermejo	Mexico	5-Dec-05	18.675	-87.708	Ponce-Taylor, Daniel	6.4		25.0%			0	0.5
534	PJ10	Pez Maya, Reserva de la Biosfer	Mexico	6-Dec-05	20.017	-87.462	Cameron, Andy	10.55		46.2%			0	0
535	PJ10	Pez Maya, Reserva de la Biosfer	Mexico	6-Dec-05	20.017	-87.462	Cameron, Andy	9.9		44.4%			0	0
536	PL05	Pez Maya, Reserva de la Biosfer	Mexico	6-Dec-05	20.050	-87.470	Cameron, Andy	3.7		14.3%			0	0
537	PL05	Pez Maya, Reserva de la Biosfer	Mexico	6-Dec-05	20.050	-87.470	Cameron, Andy	3.7		8.3%			0	0
538	PL05	Pez Maya, Reserva de la Biosfer	Mexico	6-Dec-05	20.050	-87.470	Cameron, Andy	3.55		0.0%			0	0
539	PL05	Pez Maya, Reserva de la Biosfer	Mexico	6-Dec-05	20.050	-87.470	Cameron, Andy	3.4		0.0%			0	0
540	PL20	Pez Maya, Reserva de la Biosfer	Mexico	6-Dec-05	20.051	-87.463	Cameron, Andy	14.35		25.0%			0	0
541	PL20	Pez Maya, Reserva de la Biosfer	Mexico	6-Dec-05	20.051	-87.463	Cameron, Andy	15.1		70.6%			0	0
542	RB20	Mahahual	Mexico	6-Dec-05	18.681	-87.711	Cameron, Andy	14.9		35.1%			0	0.5
543	RB20	Mahahual	Mexico	6-Dec-05	18.681	-87.711	Cameron, Andy	14.8		56.4%			0	0.5
544	RB20	Mahahual	Mexico	6-Dec-05	18.681	-87.711	Cameron, Andy	15.55		16.7%			0	0.5
545	RB20	Mahahual	Mexico	6-Dec-05	18.681	-87.711	Cameron, Andy	13.1		37.9%			0	0.5
2015	RB20	Rio Bermejo	Mexico	6-Dec-05	18.675	-87.707	Ponce-Taylor, Daniel	14.9		52.2%			0	0.5
2016	RB20	Rio Bermejo	Mexico	6-Dec-05	18.675	-87.707	Ponce-Taylor, Daniel	14.8		73.9%			0	0.5
2017	RB20	Rio Bermejo	Mexico	6-Dec-05	18.675	-87.707	Ponce-Taylor, Daniel	15.55		35.3%			0	0.5
2018	RB20	Rio Bermejo	Mexico	6-Dec-05	18.675	-87.707	Ponce-Taylor, Daniel	13.1		42.9%			0	0.5
546	LE20	Mahahual	Mexico	7-Dec-05	18.720	-87.698	Cameron, Andy	14.5		61.8%			2.15	2.15
547	LE20	Mahahual	Mexico	7-Dec-05	18.720	-87.698	Cameron, Andy	14.9		35.7%			2.15	2.15
548	LE20	Mahahual	Mexico	7-Dec-05	18.720	-87.698	Cameron, Andy	16.95		15.2%			2.15	2.15
549	PL20	Pez Maya, Reserva de la Biosfer	Mexico	7-Dec-05	20.051	-87.463	Cameron, Andy	12.2		26.3%			0	0
550	RB05	Mahahual	Mexico	7-Dec-05	18.681	-87.714	Cameron, Andy	5.35		0.0%			0	0.5
551	RB05	Mahahual	Mexico	7-Dec-05	18.681	-87.714	Cameron, Andy	5		0.0%			0	0.5
552	RB20	Mahahual	Mexico	7-Dec-05	18.681	-87.711	Cameron, Andy	12.5		36.7%			0	0.5
553	SMDR10	Pez Maya, Reserva de la Biosfer	Mexico	7-Dec-05	19.967	-87.453	Cameron, Andy	9.45		30.8%			0	0
554	SMDR10	Pez Maya, Reserva de la Biosfer	Mexico	7-Dec-05	19.967	-87.453	Cameron, Andy	9.45		56.3%			0	0
555	SMDR20	Pez Maya, Reserva de la Biosfer	Mexico	7-Dec-05	19.968	-87.452	Cameron, Andy	17.85		66.7%			0	0
556	SMDR20	Pez Maya, Reserva de la Biosfer	Mexico	7-Dec-05	19.968	-87.452	Cameron, Andy	18.15		100.0%			0	0
557	SMDR20	Pez Maya, Reserva de la Biosfer	Mexico	7-Dec-05	19.968	-87.452	Cameron, Andy	18.15		33.3%			0	0
2019	LE20	Los Escalones	Mexico	7-Dec-05	18.718	-87.692	Ponce-Taylor, Daniel	14.5		65.2%			2.15	2.15
2020	LE20	Los Escalones	Mexico	7-Dec-05	18.718	-87.692	Ponce-Taylor, Daniel	14.9		52.9%			2.15	2.15

Observation Number	Reef or Site Name	Location	Country	Date (DD-MMM-YY)	Latitude (decimal degrees)	Longitude (decimal degrees)	Primary Contributor	Depth (m)	Cover bleached (%)	Colonies bleached (%)	Cover dead (%)	Colonies dead (%)	Observed DHW (°C-weeks)	Maximum DHW (°C-weeks)
2021	LE20	Los Escalones	Mexico	7-Dec-05	18.718	-87.692	Ponce-Taylor, Daniel	16.95		46.2%			2.15	2.15
2022	RB05	Rio Bermejo	Mexico	7-Dec-05	18.675	-87.708	Ponce-Taylor, Daniel	5.35		0.0%			0	0.5
2023	RB05	Rio Bermejo	Mexico	7-Dec-05	18.675	-87.708	Ponce-Taylor, Daniel	5		0.0%			0	0.5
2024	RB20	Rio Bermejo	Mexico	7-Dec-05	18.675	-87.707	Ponce-Taylor, Daniel	12.5		47.4%			0	0.5
558	SMDR20	Pez Maya, Reserva de la Biosfer	Mexico	8-Dec-05	19.968	-87.452	Cameron, Andy	17.85		0.0%			0	0
559	SMDR20	Pez Maya, Reserva de la Biosfer	Mexico	8-Dec-05	19.968	-87.452	Cameron, Andy	18.3		33.3%			0	0
560	PJ05	Pez Maya, Reserva de la Biosfer	Mexico	24-Dec-05	20.015	-87.465	Cameron, Andy	4.6		25.0%			0	0
561	LL10	Mahahual	Mexico	11-Feb-06	18.707	-87.706	Cameron, Andy	9.75		33.3%			0	2.15
562	LL10	Mahahual	Mexico	13-Feb-06	18.707	-87.706	Cameron, Andy	10.5		40.0%			0	2.15
563	LL10	Mahahual	Mexico	13-Feb-06	18.707	-87.706	Cameron, Andy	10.05		60.0%			0	2.15
564	DB10	Mahahual	Mexico	16-Feb-06	18.724	-87.697	Cameron, Andy	8		36.0%			0	2.15
565	LL10	Mahahual	Mexico	16-Feb-06	18.707	-87.706	Cameron, Andy	9.8		42.9%			0	2.15
566	LL10	Mahahual	Mexico	16-Feb-06	18.707	-87.706	Cameron, Andy	9.2		57.9%			0	2.15
567	PJ10	Pez Maya, Reserva de la Biosfer	Mexico	16-Feb-06	20.017	-87.462	Cameron, Andy	9.3		75.0%			0	0
568	PJ10	Pez Maya, Reserva de la Biosfer	Mexico	16-Feb-06	20.017	-87.462	Cameron, Andy	9.3		16.7%			0	0
569	DB10	Mahahual	Mexico	21-Feb-06	18.724	-87.697	Cameron, Andy	7.8		20.0%			0	2.15
570	RB10	Mahahual	Mexico	21-Feb-06	18.681	-87.713	Cameron, Andy	10.05		35.7%			0	0.5
571	PL10	Pez Maya, Reserva de la Biosfer	Mexico	22-Feb-06	20.052	-87.466	Cameron, Andy	5.6		37.0%			0	0
572	PL20	Pez Maya, Reserva de la Biosfer	Mexico	22-Feb-06	20.051	-87.463	Cameron, Andy	13		72.2%			0	0
573	PJ05	Pez Maya, Reserva de la Biosfer	Mexico	24-Feb-06	20.015	-87.465	Cameron, Andy	3.2		0.0%			0	0
574	PL20	Pez Maya, Reserva de la Biosfer	Mexico	24-Feb-06	20.051	-87.463	Cameron, Andy	12.6		57.1%			0	0
575	PL10	Pez Maya, Reserva de la Biosfer	Mexico	25-Feb-06	20.052	-87.466	Cameron, Andy	6.8		13.3%			0	0
576	PL10	Pez Maya, Reserva de la Biosfer	Mexico	25-Feb-06	20.052	-87.466	Cameron, Andy	6.7		0.0%			0	0
577	PL10	Pez Maya, Reserva de la Biosfer	Mexico	25-Feb-06	20.052	-87.466	Cameron, Andy	6.35		77.8%			0	0
578	PL10	Pez Maya, Reserva de la Biosfer	Mexico	25-Feb-06	20.052	-87.466	Cameron, Andy	6.3		64.3%			0	0
579	RB10	Mahahual	Mexico	26-Feb-06	18.681	-87.713	Cameron, Andy	10		58.1%			0	0.5
580	RB10	Mahahual	Mexico	26-Feb-06	18.681	-87.713	Cameron, Andy	10.4		36.8%			0	0.5
581	RB10	Mahahual	Mexico	26-Feb-06	18.681	-87.713	Cameron, Andy	9.1		37.5%			0	0.5
582	RB10	Mahahual	Mexico	26-Feb-06	18.681	-87.713	Cameron, Andy	9.1		40.0%			0	0.5
583	LL20	Mahahual	Mexico	27-Feb-06	18.707	-87.704	Cameron, Andy	14.3		55.0%			0	2.15
584	LL20	Mahahual	Mexico	27-Feb-06	18.707	-87.704	Cameron, Andy	14.9		21.1%			0	2.15
585	DB10	Mahahual	Mexico	28-Feb-06	18.724	-87.697	Cameron, Andy	8.8		28.1%			0	2.15
586	DB10	Mahahual	Mexico	28-Feb-06	18.724	-87.697	Cameron, Andy	7.75		34.5%			0	2.15
587	LL20	Mahahual	Mexico	28-Feb-06	18.707	-87.704	Cameron, Andy	15.6		44.8%			0	2.15
588	PJ10	Pez Maya, Reserva de la Biosfer	Mexico	28-Feb-06	20.017	-87.462	Cameron, Andy	8.05		80.0%			0	0
589	PL20	Pez Maya, Reserva de la Biosfer	Mexico	28-Feb-06	20.051	-87.463	Cameron, Andy	13.65		0.0%			0	0
590	PL20	Pez Maya, Reserva de la Biosfer	Mexico	28-Feb-06	20.051	-87.463	Cameron, Andy	14.5		73.3%			0	0
591	PL20	Pez Maya, Reserva de la Biosfer	Mexico	28-Feb-06	20.051	-87.463	Cameron, Andy	13.4		39.1%			0	0
592	LL20	Mahahual	Mexico	1-Mar-06	18.707	-87.704	Cameron, Andy	15.8		71.4%			0	2.15
593	LL20	Mahahual	Mexico	1-Mar-06	18.707	-87.704	Cameron, Andy	15		56.0%			0	2.15
594	DB05	Mahahual	Mexico	2-Mar-06	18.724	-87.698	Cameron, Andy	4.75		20.0%			0	2.15
595	DB05	Mahahual	Mexico	2-Mar-06	18.724	-87.698	Cameron, Andy	4.75		38.5%			0	2.15
596	DB05	Mahahual	Mexico	2-Mar-06	18.724	-87.698	Cameron, Andy	4.85		33.3%			0	2.15
597	DB05	Mahahual	Mexico	2-Mar-06	18.724	-87.698	Cameron, Andy	5.65		25.0%			0	2.15
598	PJ10	Pez Maya, Reserva de la Biosfer	Mexico	2-Mar-06	20.017	-87.462	Cameron, Andy	8.65		25.0%			0	0
599	PJ10	Pez Maya, Reserva de la Biosfer	Mexico	2-Mar-06	20.017	-87.462	Cameron, Andy	9.9		71.4%			0	0
600	PX20	Pez Maya, Reserva de la Biosfer	Mexico	2-Mar-06	19.933	-87.432	Cameron, Andy	18.15		81.8%			0	0
601	PX20	Pez Maya, Reserva de la Biosfer	Mexico	2-Mar-06	19.933	-87.432	Cameron, Andy	17.8		7.7%			0	0
602	PAY20	Mahahual	Mexico	3-Mar-06	18.635	-87.719	Cameron, Andy	17.6		68.4%			0	0.5
603	PAY20	Mahahual	Mexico	3-Mar-06	18.635	-87.719	Cameron, Andy	18.2		31.3%			0	0.5
604	PJ05	Pez Maya, Reserva de la Biosfer	Mexico	3-Mar-06	20.015	-87.465	Cameron, Andy	4.7		100.0%			0	0
605	PJ05	Pez Maya, Reserva de la Biosfer	Mexico	3-Mar-06	20.015	-87.465	Cameron, Andy	4.8		33.3%			0	0
606	PJ05	Pez Maya, Reserva de la Biosfer	Mexico	3-Mar-06	20.015	-87.465	Cameron, Andy	5.05		0.0%			0	0
607	PJ05	Pez Maya, Reserva de la Biosfer	Mexico	3-Mar-06	20.015	-87.465	Cameron, Andy	5.2		0.0%			0	0
608	DB10	Mahahual	Mexico	4-Mar-06	18.724	-87.697	Cameron, Andy	9.1		34.5%			0	2.15
609	PX20	Pez Maya, Reserva de la Biosfer	Mexico	4-Mar-06	19.933	-87.432	Cameron, Andy	18.15		50.0%			0	0
610	PX20	Pez Maya, Reserva de la Biosfer	Mexico	4-Mar-06	19.933	-87.432	Cameron, Andy	17.9		25.0%			0	0
611	RB20	Mahahual	Mexico	4-Mar-06	18.681	-87.711	Cameron, Andy	14.35		85.7%			0	0.5

Observation Number	Reef or Site Name	Location	Country	Date (DD-MMM-YY)	Latitude (decimal degrees)	Longitude (decimal degrees)	Primary Contributor	Depth (m)	Cover bleached (%)	Colonies bleached (%)	Cover dead (%)	Colonies dead (%)	Observed DHW (°C-weeks)	Maximum DHW (°C-weeks)
612	RB20	Mahahual	Mexico	4-Mar-06	18.681	-87.711	Cameron, Andy	16.1		64.0%			0	0.5
613	LL05	Mahahual	Mexico	6-Mar-06	18.707	-87.707	Cameron, Andy	5.5		41.2%			0	2.15
614	LL05	Mahahual	Mexico	6-Mar-06	18.707	-87.707	Cameron, Andy	5.1		18.2%			0	2.15
615	LL05	Mahahual	Mexico	6-Mar-06	18.707	-87.707	Cameron, Andy	5.35		52.9%			0	2.15
616	LL05	Mahahual	Mexico	6-Mar-06	18.707	-87.707	Cameron, Andy	5.25		26.3%			0	2.15
617	PL05	Pez Maya, Reserva de la Biosfer	Mexico	6-Mar-06	20.050	-87.470	Cameron, Andy	3.2		20.0%			0	0
618	PL05	Pez Maya, Reserva de la Biosfer	Mexico	6-Mar-06	20.050	-87.470	Cameron, Andy	3.2		16.7%			0	0
619	PL05	Pez Maya, Reserva de la Biosfer	Mexico	6-Mar-06	20.050	-87.470	Cameron, Andy	2.55		30.8%			0	0
620	PL05	Pez Maya, Reserva de la Biosfer	Mexico	6-Mar-06	20.050	-87.470	Cameron, Andy	2.85		0.0%			0	0
621	PX10	Pez Maya, Reserva de la Biosfer	Mexico	6-Mar-06	19.934	-87.434	Cameron, Andy	12.75		76.9%			0	0
622	PX10	Pez Maya, Reserva de la Biosfer	Mexico	6-Mar-06	19.934	-87.434	Cameron, Andy	12.05		21.4%			0	0
623	PX20	Pez Maya, Reserva de la Biosfer	Mexico	6-Mar-06	19.933	-87.432	Cameron, Andy	17.85		14.3%			0	0
624	RB20	Mahahual	Mexico	6-Mar-06	18.681	-87.711	Cameron, Andy	14.2		66.7%			0	0.5
625	RB20	Mahahual	Mexico	6-Mar-06	18.681	-87.711	Cameron, Andy	13.35		65.4%			0	0.5
626	DB05	Mahahual	Mexico	7-Mar-06	18.724	-87.698	Cameron, Andy	4.25		16.7%			0	2.15
627	PDC20	Mahahual	Mexico	7-Mar-06	18.667	-87.714	Cameron, Andy	15.75		60.0%			0	0.5
628	PDC20	Mahahual	Mexico	7-Mar-06	18.667	-87.714	Cameron, Andy	15.45		77.3%			0	0.5
629	PX05	Pez Maya, Reserva de la Biosfer	Mexico	7-Mar-06	19.932	-87.434	Cameron, Andy	6.6		0.0%			0	0
630	PX05	Pez Maya, Reserva de la Biosfer	Mexico	7-Mar-06	19.932	-87.434	Cameron, Andy	7.1		62.5%			0	0
631	PX05	Pez Maya, Reserva de la Biosfer	Mexico	7-Mar-06	19.932	-87.434	Cameron, Andy	6.65		83.3%			0	0
632	PL05	Pez Maya, Reserva de la Biosfer	Mexico	8-Mar-06	20.050	-87.470	Cameron, Andy	3.4		42.9%			0	0
633	PX10	Pez Maya, Reserva de la Biosfer	Mexico	8-Mar-06	19.934	-87.434	Cameron, Andy	10.65		19.0%			0	0
634	PX10	Pez Maya, Reserva de la Biosfer	Mexico	8-Mar-06	19.934	-87.434	Cameron, Andy	10.25		11.1%			0	0
635	PX10	Pez Maya, Reserva de la Biosfer	Mexico	8-Mar-06	19.934	-87.434	Cameron, Andy	12.65		64.3%			0	0
636	PX05	Pez Maya, Reserva de la Biosfer	Mexico	14-Mar-06	19.932	-87.434	Cameron, Andy	7.45		100.0%			0	0
637	PDC10	Mahahual	Mexico	15-Mar-06	18.667	-87.716	Cameron, Andy	9.8		52.4%			0	0.5
638	PDC10	Mahahual	Mexico	15-Mar-06	18.667	-87.716	Cameron, Andy	10.45		23.5%			0	0.5
639	PDC20	Mahahual	Mexico	15-Mar-06	18.667	-87.714	Cameron, Andy	15.95		60.0%			0	0.5
640	PDC10	Mahahual	Mexico	16-Mar-06	18.667	-87.716	Cameron, Andy	10.25		25.0%			0	0.5
641	PDC10	Mahahual	Mexico	16-Mar-06	18.667	-87.716	Cameron, Andy	10.35		52.9%			0	0.5
642	RB20	Mahahual	Mexico	16-Mar-06	18.681	-87.711	Cameron, Andy	15.15		50.0%			0	0.5
643	PAY20	Mahahual	Mexico	17-Mar-06	18.635	-87.719	Cameron, Andy	16.5		40.0%			0	0.5
644	PAY20	Mahahual	Mexico	17-Mar-06	18.635	-87.719	Cameron, Andy	18.1		38.1%			0	0.5
645	PDC10	Mahahual	Mexico	17-Mar-06	18.667	-87.716	Cameron, Andy	10.55		33.3%			0	0.5
646	PDC20	Mahahual	Mexico	17-Mar-06	18.667	-87.714	Cameron, Andy	14.7		42.9%			0	0.5
647	RB05	Mahahual	Mexico	17-Mar-06	18.681	-87.714	Cameron, Andy	5.5		12.5%			0	0.5
648	RB05	Mahahual	Mexico	17-Mar-06	18.681	-87.714	Cameron, Andy	6.1		50.0%			0	0.5
649	PAY20	Mahahual	Mexico	18-Mar-06	18.635	-87.719	Cameron, Andy	16.75		25.9%			0	0.5
650	LE05	Mahahual	Mexico	20-Mar-06	18.720	-87.701	Cameron, Andy	3.9		11.1%			0	2.15
651	PDC05	Mahahual	Mexico	24-Mar-06	18.667	-87.717	Cameron, Andy	5.75		60.0%			0	0.5
652	PDC05	Mahahual	Mexico	24-Mar-06	18.667	-87.717	Cameron, Andy	6		66.7%			0	0.5
653	PDC05	Mahahual	Mexico	24-Mar-06	18.667	-87.717	Cameron, Andy	4.75		60.0%			0	0.5
654	PDC05	Mahahual	Mexico	24-Mar-06	18.667	-87.717	Cameron, Andy	4.75		55.6%			0	0.5
655	PDC20	Mahahual	Mexico	24-Mar-06	18.667	-87.714	Cameron, Andy	14.4		45.0%			0	0.5
656	PDC05	Mahahual	Mexico	25-Mar-06	18.667	-87.717	Cameron, Andy	5.2		50.0%			0	0.5
657	LE10	Mahahual	Mexico	4-May-06	18.720	-87.699	Cameron, Andy	9.7		12.9%			0	2.15
658	LE10	Mahahual	Mexico	4-May-06	18.720	-87.699	Cameron, Andy	9.55		20.0%			0	2.15
659	LE10	Mahahual	Mexico	4-May-06	18.720	-87.699	Cameron, Andy	9.35		46.2%			0	2.15
660	BUC10	Mahahual	Mexico	5-May-06	18.687	-87.711	Cameron, Andy	10.25		28.0%			0	0.5
661	PP10	Pez Maya, Reserva de la Biosfer	Mexico	8-May-06	20.038	-87.466	Cameron, Andy	9.45		33.3%			0	0
662	PY20	Pez Maya, Reserva de la Biosfer	Mexico	9-May-06	19.973	-87.453	Cameron, Andy	18.6		100.0%			0	0
663	PY20	Pez Maya, Reserva de la Biosfer	Mexico	9-May-06	19.973	-87.453	Cameron, Andy	18.05		33.3%			0	0
664	PP10	Pez Maya, Reserva de la Biosfer	Mexico	10-May-06	20.038	-87.466	Cameron, Andy	10.25		100.0%			0	0
665	PP10	Pez Maya, Reserva de la Biosfer	Mexico	10-May-06	20.038	-87.466	Cameron, Andy	10.5		37.5%			0	0
666	PY20	Pez Maya, Reserva de la Biosfer	Mexico	10-May-06	19.973	-87.453	Cameron, Andy	18.6		66.7%			0	0
667	PY20	Pez Maya, Reserva de la Biosfer	Mexico	10-May-06	19.973	-87.453	Cameron, Andy	17.6		61.5%			0	0
668	PSJ10	Pez Maya, Reserva de la Biosfer	Mexico	11-May-06	19.883	-87.418	Cameron, Andy	9		83.3%			0	0
669	PSJ10	Pez Maya, Reserva de la Biosfer	Mexico	11-May-06	19.883	-87.418	Cameron, Andy	8.5		42.9%			0	0

Observation Number	Reef or Site Name	Location	Country	Date (DD-MMM-YY)	Latitude (decimal degrees)	Longitude (decimal degrees)	Primary Contributor	Depth (m)	Cover bleached (%)	Colonies bleached (%)	Cover dead (%)	Colonies dead (%)	Observed DHW (°C-weeks)	Maximum DHW (°C-weeks)
670	BUC10	Mahahual	Mexico	12-May-06	18.687	-87.711	Cameron, Andy	11.15		33.3%			0	0.5
671	LPC10	Mahahual	Mexico	12-May-06	18.659	-87.717	Cameron, Andy	11.2		22.7%			0	0.5
672	SMDR20	Pez Maya, Reserva de la Biosfer	Mexico	13-May-06	19.968	-87.452	Cameron, Andy	18.1		44.4%			0	0
673	BUC10	Mahahual	Mexico	16-May-06	18.687	-87.711	Cameron, Andy	11.25		41.7%			0	0.5
674	BUC10	Mahahual	Mexico	16-May-06	18.687	-87.711	Cameron, Andy	11.3		45.2%			0	0.5
675	LPC10	Mahahual	Mexico	16-May-06	18.659	-87.717	Cameron, Andy	12.05		31.3%			0	0.5
676	LPC10	Mahahual	Mexico	16-May-06	18.659	-87.717	Cameron, Andy	11.25		22.2%			0	0.5
677	PSJ20	Pez Maya, Reserva de la Biosfer	Mexico	16-May-06	19.880	-87.412	Cameron, Andy	21.15		100.0%			0	0
678	PSJ20	Pez Maya, Reserva de la Biosfer	Mexico	16-May-06	19.880	-87.412	Cameron, Andy	20.8		87.5%			0	0
679	BUC05	Mahahual	Mexico	17-May-06	18.687	-87.712	Cameron, Andy	8.5		5.0%			0	0.5
680	BUC05	Mahahual	Mexico	17-May-06	18.687	-87.712	Cameron, Andy	5.75		38.5%			0	0.5
681	BUC05	Mahahual	Mexico	17-May-06	18.687	-87.712	Cameron, Andy	6.55		8.3%			0	0.5
682	BUC05	Mahahual	Mexico	17-May-06	18.687	-87.712	Cameron, Andy	7.45		22.2%			0	0.5
683	LPC20	Mahahual	Mexico	17-May-06	18.660	-87.716	Cameron, Andy	18.75		50.0%			0	0.5
684	SMDR10	Pez Maya, Reserva de la Biosfer	Mexico	17-May-06	19.967	-87.453	Cameron, Andy	10.4		20.0%			0	0
685	SMDR10	Pez Maya, Reserva de la Biosfer	Mexico	17-May-06	19.967	-87.453	Cameron, Andy	10.8		61.5%			0	0
686	PSJ20	Pez Maya, Reserva de la Biosfer	Mexico	18-May-06	19.880	-87.412	Cameron, Andy	20.55		33.3%			0	0
687	PP10	Pez Maya, Reserva de la Biosfer	Mexico	19-May-06	20.038	-87.466	Cameron, Andy	10.4		44.4%			0	0
688	PP10	Pez Maya, Reserva de la Biosfer	Mexico	19-May-06	20.038	-87.466	Cameron, Andy	10.25		60.0%			0	0
689	PY20	Pez Maya, Reserva de la Biosfer	Mexico	19-May-06	19.973	-87.453	Cameron, Andy	18.6		90.0%			0	0
690	SMDR10	Pez Maya, Reserva de la Biosfer	Mexico	19-May-06	19.967	-87.453	Cameron, Andy	9.75		30.8%			0	0
691	LE05	Mahahual	Mexico	20-May-06	18.720	-87.701	Cameron, Andy	5.3		30.8%			0	2.15
692	LE05	Mahahual	Mexico	20-May-06	18.720	-87.701	Cameron, Andy	6.2		22.2%			0	2.15
693	BUC20	Mahahual	Mexico	23-May-06	18.687	-87.709	Cameron, Andy	19.1		23.5%			0	0.5
694	LE20	Mahahual	Mexico	24-May-06	18.720	-87.698	Cameron, Andy	17.05		31.6%			0	2.15
695	LE20	Mahahual	Mexico	24-May-06	18.720	-87.698	Cameron, Andy	15.6		10.0%			0	2.15
696	PP05	Pez Maya, Reserva de la Biosfer	Mexico	25-May-06	20.040	-87.470	Cameron, Andy	3		66.7%			0	0
697	PP05	Pez Maya, Reserva de la Biosfer	Mexico	25-May-06	20.040	-87.470	Cameron, Andy	3.35		42.9%			0	0
698	SMDR20	Pez Maya, Reserva de la Biosfer	Mexico	25-May-06	19.968	-87.452	Cameron, Andy	18.75		100.0%			0	0
699	BUC20	Mahahual	Mexico	29-May-06	18.687	-87.709	Cameron, Andy	15.95		20.7%			0	0.5
700	BUC20	Mahahual	Mexico	29-May-06	18.687	-87.709	Cameron, Andy	16.65		40.0%			0	0.5
701	LE10	Mahahual	Mexico	29-May-06	18.720	-87.699	Cameron, Andy	8.95		11.1%			0	2.15
702	SMDR10	Pez Maya, Reserva de la Biosfer	Mexico	29-May-06	19.967	-87.453	Cameron, Andy	9.15		30.0%			0	0
703	SMDR10	Pez Maya, Reserva de la Biosfer	Mexico	29-May-06	19.967	-87.453	Cameron, Andy	9.15		82.4%			0	0
704	SMDR20	Pez Maya, Reserva de la Biosfer	Mexico	29-May-06	19.968	-87.452	Cameron, Andy	18.15		100.0%			0	0
705	SMDR20	Pez Maya, Reserva de la Biosfer	Mexico	29-May-06	19.968	-87.452	Cameron, Andy	17.7		100.0%			0	0
21	Yucab	Cozumel	Mexico	30-May-06	20.421	-87.017	Alvarez, Lorenzo	12.6		0.0%		0.0%	0	0
706	BUC10	Mahahual	Mexico	30-May-06	18.687	-87.711	Cameron, Andy	10		11.8%			0	0.5
707	BUC20	Mahahual	Mexico	30-May-06	18.687	-87.709	Cameron, Andy	16.3		20.0%			0	0.5
708	BUC20	Mahahual	Mexico	30-May-06	18.687	-87.709	Cameron, Andy	15.65		10.5%			0	0.5
709	PP05	Pez Maya, Reserva de la Biosfer	Mexico	30-May-06	20.040	-87.470	Cameron, Andy	1.35		22.2%			0	0
710	PSJ20	Pez Maya, Reserva de la Biosfer	Mexico	30-May-06	19.880	-87.412	Cameron, Andy	20.7		87.5%			0	0
22	Colombia	Cozumel	Mexico	31-May-06	20.325	-87.027	Alvarez, Lorenzo	10.59		1.3%		0.0%	0	0
23	Dalila	Cozumel	Mexico	31-May-06	20.349	-87.029	Alvarez, Lorenzo	12.28		4.2%		0.0%	0	0
24	Paraiso	Cozumel	Mexico	31-May-06	20.469	-86.983	Alvarez, Lorenzo	9.88		13.6%		0.0%	0	0
711	LPC20	Mahahual	Mexico	31-May-06	18.660	-87.716	Cameron, Andy	17		4.3%			0	0.5
712	PSJ10	Pez Maya, Reserva de la Biosfer	Mexico	31-May-06	19.883	-87.418	Cameron, Andy	9.1		44.4%			0	0
713	PSJ10	Pez Maya, Reserva de la Biosfer	Mexico	31-May-06	19.883	-87.418	Cameron, Andy	7.9		100.0%			0	0
714	PSJ10	Pez Maya, Reserva de la Biosfer	Mexico	31-May-06	19.883	-87.418	Cameron, Andy	9.45		81.8%			0	0
715	SMDR05	Pez Maya, Reserva de la Biosfer	Mexico	31-May-06	19.967	-87.461	Cameron, Andy	1.65		11.1%			0	0
716	SMDR05	Pez Maya, Reserva de la Biosfer	Mexico	31-May-06	19.967	-87.461	Cameron, Andy	2.4		6.3%			0	0
25	Paso del Cedral	Cozumel	Mexico	1-Jun-06	20.374	-87.029	Alvarez, Lorenzo	13.94		1.4%		0.0%	0	0
717	LE20	Mahahual	Mexico	1-Jun-06	18.720	-87.698	Cameron, Andy	15.5		23.1%			0	2.15
718	LE20	Mahahual	Mexico	1-Jun-06	18.720	-87.698	Cameron, Andy	15.35		27.3%			0	2.15
719	LPC20	Mahahual	Mexico	1-Jun-06	18.660	-87.716	Cameron, Andy	14.25		23.8%			0	0.5
26	Chancanaab	Cozumel	Mexico	2-Jun-06	20.441	-87.002	Alvarez, Lorenzo	11.89		2.9%		0.0%	0	0
720	BUC05	Mahahual	Mexico	2-Jun-06	18.687	-87.712	Cameron, Andy	6.3		35.7%			0	0.5
721	PP05	Pez Maya, Reserva de la Biosfer	Mexico	2-Jun-06	20.040	-87.470	Cameron, Andy	3.85		18.8%			0	0

Observation Number	Reef or Site Name	Location	Country	Date (DD-MMM-YY)	Latitude (decimal degrees)	Longitude (decimal degrees)	Primary Contributor	Depth (m)	Cover bleached (%)	Colonies bleached (%)	Cover dead (%)	Colonies dead (%)	Observed DHW (°C-weeks)	Maximum DHW (°C-weeks)
722	PSJ20	Pez Maya, Reserva de la Biosfer	Mexico	2-Jun-06	19.880	-87.412	Cameron, Andy	19.95		73.3%			0	0
723	SMDR05	Pez Maya, Reserva de la Biosfer	Mexico	2-Jun-06	19.967	-87.461	Cameron, Andy	2.85		14.3%			0	0
724	FV20	Mahahual	Mexico	3-Jun-06	18.742	-87.674	Cameron, Andy	20.85		0.0%			0	2.15
725	LE05	Mahahual	Mexico	3-Jun-06	18.720	-87.701	Cameron, Andy	6.6		20.0%			0	2.15
726	LE05	Mahahual	Mexico	3-Jun-06	18.720	-87.701	Cameron, Andy	6.15		63.6%			0	2.15
727	LPC05	Mahahual	Mexico	5-Jun-06	18.660	-87.719	Cameron, Andy	5.2		50.0%			0	0.5
728	LPC05	Mahahual	Mexico	5-Jun-06	18.660	-87.719	Cameron, Andy	4.9		25.0%			0	0.5
729	LPC05	Mahahual	Mexico	5-Jun-06	18.660	-87.719	Cameron, Andy	5.35		33.3%			0	0.5
730	LPC05	Mahahual	Mexico	5-Jun-06	18.660	-87.719	Cameron, Andy	4.6		33.3%			0	0.5
731	LPC10	Mahahual	Mexico	5-Jun-06	18.659	-87.717	Cameron, Andy	11		13.0%			0	0.5
732	PC10	Pez Maya, Reserva de la Biosfer	Mexico	5-Jun-06	20.099	-87.462	Cameron, Andy	12.8		66.7%			0	0
733	PC10	Pez Maya, Reserva de la Biosfer	Mexico	5-Jun-06	20.099	-87.462	Cameron, Andy	9.8		100.0%			0	0
734	PC10	Pez Maya, Reserva de la Biosfer	Mexico	5-Jun-06	20.099	-87.462	Cameron, Andy	9.1		88.2%			0	0
735	PC10	Pez Maya, Reserva de la Biosfer	Mexico	5-Jun-06	20.099	-87.462	Cameron, Andy	9.3		28.6%			0	0
736	LE20	Mahahual	Mexico	6-Jun-06	18.720	-87.698	Cameron, Andy	15.6		19.4%			0	2.15
737	PC05	Pez Maya, Reserva de la Biosfer	Mexico	6-Jun-06	20.100	-87.464	Cameron, Andy	7.45		40.0%			0	0
738	PC05	Pez Maya, Reserva de la Biosfer	Mexico	6-Jun-06	20.100	-87.464	Cameron, Andy	5.95		100.0%			0	0
739	PC05	Pez Maya, Reserva de la Biosfer	Mexico	6-Jun-06	20.100	-87.464	Cameron, Andy	7.9		100.0%			0	0
740	PC05	Pez Maya, Reserva de la Biosfer	Mexico	6-Jun-06	20.100	-87.464	Cameron, Andy	8.65		83.3%			0	0
741	PC05	Pez Maya, Reserva de la Biosfer	Mexico	6-Jun-06	20.100	-87.464	Cameron, Andy	6.4		88.9%			0	0
742	PSJ05	Pez Maya, Reserva de la Biosfer	Mexico	6-Jun-06	19.883	-87.426	Cameron, Andy	6.1		50.0%			0	0
743	PSJ05	Pez Maya, Reserva de la Biosfer	Mexico	6-Jun-06	19.883	-87.426	Cameron, Andy	6.1		63.6%			0	0
744	PSJ05	Pez Maya, Reserva de la Biosfer	Mexico	6-Jun-06	19.883	-87.426	Cameron, Andy	6.4		30.0%			0	0
745	PSJ05	Pez Maya, Reserva de la Biosfer	Mexico	6-Jun-06	19.883	-87.426	Cameron, Andy	5.95		50.0%			0	0
746	LPC20	Mahahual	Mexico	7-Jun-06	18.660	-87.716	Cameron, Andy	15.35		8.0%			0	0.5
747	PC10	Pez Maya, Reserva de la Biosfer	Mexico	7-Jun-06	20.099	-87.462	Cameron, Andy	9.45		20.0%			0	0
748	PSJ05	Pez Maya, Reserva de la Biosfer	Mexico	7-Jun-06	19.883	-87.426	Cameron, Andy	6.25		4.5%			0	0
749	LE10	Mahahual	Mexico	8-Jun-06	18.720	-87.699	Cameron, Andy	10.25		18.4%			0	2.15
750	PAY10	Mahahual	Mexico	8-Jun-06	18.634	-87.720	Cameron, Andy	9.1		57.1%			0	0.5
751	SMDR05	Pez Maya, Reserva de la Biosfer	Mexico	8-Jun-06	19.967	-87.461	Cameron, Andy	1.65		33.3%			0	0
752	LPC20	Mahahual	Mexico	9-Jun-06	18.660	-87.716	Cameron, Andy	15.05		8.0%			0	0.5
753	FV05	Mahahual	Mexico	14-Jun-06	18.742	-87.679	Cameron, Andy	7.3		41.7%			0	2.15
754	FV05	Mahahual	Mexico	14-Jun-06	18.742	-87.679	Cameron, Andy	6.8		33.3%			0	2.15
755	FV10	Mahahual	Mexico	14-Jun-06	18.742	-87.677	Cameron, Andy	11		6.3%			0	2.15
756	FV10	Mahahual	Mexico	14-Jun-06	18.742	-87.677	Cameron, Andy	10.7		25.0%			0	2.15
757	PP05	Pez Maya, Reserva de la Biosfer	Mexico	30-Jun-06	20.040	-87.470	Cameron, Andy	1.8		0.0%			0	0
2815	Sea Aquarium	West Coast	NethAnt - Curacao	19-Sep-05	12.084	-68.895	Weil, Ernesto	20.5		0.5%			0	2.55
2819	East coast	Saba	NethAnt - Saba	16-Aug-05	17.623	-63.220	Williams, Ernest	14	0.0%	0.0%	0.0%	0.0%	4.05	17.2
2820	East coast	Saba	NethAnt - Saba	2-Sep-05	17.623	-63.220	Williams, Ernest	14	7.5%	7.5%	0.0%	0.0%	7.55	17.2
2821	East coast	Saba	NethAnt - Saba	3-Sep-05	17.623	-63.220	Williams, Ernest	14	7.5%	7.5%	0.0%	0.0%	7.55	17.2
2822	East coast	Saba	NethAnt - Saba	7-Sep-05	17.623	-63.220	Williams, Ernest	14	7.5%	7.5%	0.0%	0.0%	8.05	17.2
2823	East coast	Saba	NethAnt - Saba	15-Sep-05	17.623	-63.220	Williams, Ernest	14	60.0%	60.0%	0.0%	0.0%	9.8	17.2
2824	Core Gut	Saba	NethAnt - Saba	16-Sep-05	17.623	-63.220	Williams, Ernest	24	100.0%	100.0%	1.0%	0.0%	10.55	17.2
2825	East coast	Saba	NethAnt - Saba	29-Sep-05	17.623	-63.220	Williams, Ernest	14	77.5%	77.5%	0.0%	0.0%	13.65	17.2
2826	Babylon	Saba	NethAnt - Saba	11-Oct-05	17.620	-63.258	Williams, Ernest	18	100.0%	100.0%	1.0%	0.0%	15.9	17.2
2827	East coast	Saba	NethAnt - Saba	11-Oct-05	17.623	-63.220	Williams, Ernest	14	97.5%	97.5%	1.0%	0.0%	15.9	17.2
2828	Custom’s House	Saba	NethAnt - Saba	12-Oct-05	17.624	-63.258	Williams, Ernest	20	98.0%	98.0%	1.0%	0.0%	15.9	17.2
2829	Tent Reef	Saba	NethAnt - Saba	12-Oct-05	17.640	-63.256	Williams, Ernest	16	90.0%	90.0%	1.0%	0.0%	15.9	17.2
2830	Tent Reef (shallow)	Saba	NethAnt - Saba	12-Oct-05	17.640	-63.256	Williams, Ernest	3.5	46.0%	46.3%	1.0%	0.0%	15.9	17.2
2831	50/50	Saba	NethAnt - Saba	13-Oct-05	17.618	-63.260	Williams, Ernest	18.5	100.0%	100.0%	1.0%	0.0%	15.95	17.2
2832	Third Encounter	Saba	NethAnt - Saba	13-Oct-05	17.624	-63.272	Williams, Ernest	30.3	100.0%	100.0%	1.0%	0.0%	15.95	17.2
2833	Third Encounter (deep)	Saba	NethAnt - Saba	13-Oct-05	17.624	-63.272	Williams, Ernest	42.7	100.0%	100.0%	1.0%	0.0%	15.95	17.2
2834	Torren’s Point	Saba	NethAnt - Saba	14-Oct-05	17.634	-63.252	Williams, Ernest	12.5	80.0%	80.0%	1.0%	0.0%	15.95	17.2
2835	Twilight Zone	Saba	NethAnt - Saba	14-Oct-05	17.624	-63.272	Williams, Ernest	28	100.0%	100.0%	1.0%	0.0%	15.95	17.2
2836	Man o’ War Shoal	Saba	NethAnt - Saba	15-Oct-05	17.637	-63.255	Williams, Ernest	13.5	95.0%	95.0%	1.0%	0.0%	15.95	17.2
2837	Outer Limits	Saba	NethAnt - Saba	15-Oct-05	17.625	-63.270	Williams, Ernest	28.5	100.0%	100.0%	1.0%	0.0%	15.95	17.2
2838	Torren’s Point (shallow)	Saba	NethAnt - Saba	15-Oct-05	17.634	-63.252	Williams, Ernest	9	80.0%	80.0%	1.0%	0.0%	15.95	17.2
2839	East coast	Saba	NethAnt - Saba	18-Oct-05	17.623	-63.220	Williams, Ernest	14	85.0%	85.0%	2.0%	0.0%	16.55	17.2

Observation Number	Reef or Site Name	Location	Country	Date (DD-MMM-YY)	Latitude (decimal degrees)	Longitude (decimal degrees)	Primary Contributor	Depth (m)	Cover bleached (%)	Colonies bleached (%)	Cover dead (%)	Colonies dead (%)	Observed DHW (°C-weeks)	Maximum DHW (°C-weeks)
2840	East coast	Saba	NethAnt - Saba	8-Nov-05	17.623	-63.220	Williams, Ernest	14	40.0%	47.5%	2.0%	0.0%	14.25	17.2
1053	15, Galeta	Central Coast	Panama	29-Aug-05	9.405	-79.860	Guzman, Hector	8	20.0%		0.0%		8.65	9.8
1054	16, Largo Remo	Central Coast	Panama	30-Aug-05	9.397	-79.832	Guzman, Hector	8	20.0%		0.0%		8.65	9.8
1055	17, Punta Muerto	Central Coast	Panama	30-Aug-05	9.405	-79.819	Guzman, Hector	8	20.0%		0.0%		8.65	9.8
1056	18, Doncella	Central Coast	Panama	1-Sep-05	9.580	-79.670	Guzman, Hector	4.5	20.0%		0.0%		10.1	11.3
1057	19, Palina West	Central Coast	Panama	1-Sep-05	9.614	-79.595	Guzman, Hector	10.5	20.0%		0.0%		10.1	11.3
1058	11, Colon	Bocas del Toro	Panama	8-Sep-05	9.256	-82.235	Guzman, Hector	10.5	70.0%		5.8%		7.55	10.2
1059	10, Cayo Roldan	Bocas del Toro	Panama	9-Sep-05	9.349	-82.263	Guzman, Hector	10.5	10.0%		0.0%		5.95	8.15
1060	14, Cayo Agua	Bocas del Toro	Panama	10-Sep-05	9.133	-82.040	Guzman, Hector	8	20.0%		3.1%		7.55	10.2
1061	13, Bastimentos	Bocas del Toro	Panama	11-Sep-05	9.267	-82.120	Guzman, Hector	5.5	20.0%		5.0%		6.75	10.2
1062	9, Cristóbal	Bocas del Toro	Panama	12-Sep-05	9.220	-82.325	Guzman, Hector	8	30.0%		5.2%		6.75	10.2
1063	12, Buttonwood (Solarte)	Bocas del Toro	Panama	13-Sep-05	9.308	-82.207	Guzman, Hector	8	30.0%		1.4%		5.15	8.15
1429	Punta Caracol	Bocas del Toro	Panama	30-Sep-05	9.227	-82.182	Kline, David	6	70.0%	80.0%	20.0%	10.0%	3.35	10.2
1064	11, Colon	Bocas del Toro	Panama	7-Nov-05	9.256	-82.235	Guzman, Hector	10.5		25.4%		2.6%	1.5	10.2
1065	10, Cayo Roldan	Bocas del Toro	Panama	9-Nov-05	9.349	-82.263	Guzman, Hector	10.5		17.1%		0.4%	0	8.15
1066	13, Bastimentos	Bocas del Toro	Panama	11-Nov-05	9.267	-82.120	Guzman, Hector	5.5		3.6%		1.7%	1.5	10.2
1067	14, Cayo Agua	Bocas del Toro	Panama	12-Nov-05	9.133	-82.040	Guzman, Hector	8		6.4%		16.1%	1.5	10.2
1068	9, Cristóbal	Bocas del Toro	Panama	12-Nov-05	9.220	-82.325	Guzman, Hector	8		7.8%		4.4%	1.5	10.2
1069	12, Buttonwood (Solarte)	Bocas del Toro	Panama	13-Nov-05	9.308	-82.207	Guzman, Hector	8		15.8%		0.1%	0	8.15
1430	Crawl Cay	Bocas del Toro	Panama	21-Nov-05	9.153	-82.075	Kline, David	8.5	70.0%	70.0%	10.0%	10.0%	1.5	10.2
1431	Punta Caracol	Bocas del Toro	Panama	21-Nov-05	9.227	-82.182	Kline, David	6	70.0%	80.0%	20.0%	10.0%	1.5	10.2
1070	10, Cayo Roldan	Bocas del Toro	Panama	24-Aug-06	9.349	-82.263	Guzman, Hector	10.5		2.7%		0.0%	0	8.15
1071	11, Colon	Bocas del Toro	Panama	24-Aug-06	9.256	-82.235	Guzman, Hector	10.5		6.5%		6.4%	0	10.2
1072	9, Cristóbal	Bocas del Toro	Panama	24-Aug-06	9.220	-82.325	Guzman, Hector	8		1.0%		0.0%	0	10.2
1073	12, Buttonwood (Solarte)	Bocas del Toro	Panama	25-Aug-06	9.308	-82.207	Guzman, Hector	8		3.1%		0.1%	0	8.15
1074	13, Bastimentos	Bocas del Toro	Panama	27-Aug-06	9.267	-82.120	Guzman, Hector	5.5		0.0%		0.2%	0	10.2
1075	14, Cayo Agua	Bocas del Toro	Panama	27-Aug-06	9.133	-82.040	Guzman, Hector	8		0.2%		6.2%	0	10.2
2092	Malgretoute	Soufriere	St. Lucia	29-Dec-05	13.843	-61.065	Reef Check	3	22.0%	33.0%			2.7	12.2
2093	Malgretoute	Soufriere	St. Lucia	29-Dec-05	13.843	-61.065	Reef Check	10	44.5%	55.0%			2.7	12.2
2085	Plaintain Mustique	Mustique	St. Vincent	12-Nov-05	12.870	-61.195	Reef Check	9	75.0%	75.0%			10	11.85
2124	Caribbean coast of Tobago	Caribbean coast of Tobago	Trin&Tob -Tobago	14-Oct-05	11.180	-60.877	ReefBase	5	80.0%				9.25	9.75
891	Mt Irvine Reef	Tobago	Trin&Tob -Tobago	29-Oct-05	11.199	-60.800	Day, Owen	12		56.7%			9.75	9.75
892	Mt Irvine Reef	Tobago	Trin&Tob -Tobago	29-Oct-05	11.198	-60.800	Day, Owen	7		63.2%			9.75	9.75
893	Mt Irvine Reef	Tobago	Trin&Tob -Tobago	29-Oct-05	11.200	-60.800	Day, Owen	12		85.7%			9.75	9.75
894	Mt Irvine Reef	Tobago	Trin&Tob -Tobago	29-Oct-05	11.199	-60.799	Day, Owen	7		100.0%			9.75	9.75
895	Plymouth Back Bay	Tobago	Trin&Tob -Tobago	29-Oct-05	11.227	-60.774	Day, Owen	12		100.0%			9.75	9.75
896	Plymouth Reef West	Tobago	Trin&Tob -Tobago	29-Oct-05	11.222	-60.784	Day, Owen	12		78.9%			9.75	9.75
897	Plymouth Reef West	Tobago	Trin&Tob -Tobago	29-Oct-05	11.222	-60.784	Day, Owen	7		52.6%			9.75	9.75
898	Plymouth Reef West	Tobago	Trin&Tob -Tobago	29-Oct-05	11.223	-60.784	Day, Owen	12		88.9%			9.75	9.75
899	Plymouth Reef West	Tobago	Trin&Tob -Tobago	29-Oct-05	11.222	-60.784	Day, Owen	7		63.6%			9.75	9.75
900	Castara Point North	Tobago	Trin&Tob -Tobago	31-Oct-05	11.284	-60.699	Day, Owen	12		100.0%			9.75	9.75
901	Castara Point North	Tobago	Trin&Tob -Tobago	31-Oct-05	11.284	-60.699	Day, Owen	7		92.9%			9.75	9.75
902	Castara Point South	Tobago	Trin&Tob -Tobago	31-Oct-05	11.282	-60.698	Day, Owen	12		100.0%			9.75	9.75
903	Castara Point South	Tobago	Trin&Tob -Tobago	31-Oct-05	11.282	-60.698	Day, Owen	7		86.7%			9.75	9.75
904	Culloden Reef North	Tobago	Trin&Tob -Tobago	31-Oct-05	11.250	-60.747	Day, Owen	12		100.0%			9.75	9.75
905	Culloden Reef North	Tobago	Trin&Tob -Tobago	31-Oct-05	11.250	-60.747	Day, Owen	7		100.0%			9.75	9.75
906	Culloden Reef South	Tobago	Trin&Tob -Tobago	31-Oct-05	11.247	-60.751	Day, Owen	12		62.8%			9.75	9.75
907	Culloden Reef South	Tobago	Trin&Tob -Tobago	31-Oct-05	11.247	-60.750	Day, Owen	7		66.7%			9.75	9.75
908	Plymouth Back Bay	Tobago	Trin&Tob -Tobago	31-Oct-05	11.228	-60.774	Day, Owen	12		26.3%			9.75	9.75
909	Plymouth Back Bay	Tobago	Trin&Tob -Tobago	31-Oct-05	11.227	-60.774	Day, Owen	7		27.3%			9.75	9.75
910	Plymouth Back Bay	Tobago	Trin&Tob -Tobago	31-Oct-05	11.227	-60.773	Day, Owen	12		89.5%			9.75	9.75
911	Plymouth Back Bay	Tobago	Trin&Tob -Tobago	31-Oct-05	11.227	-60.773	Day, Owen	7		30.0%			9.75	9.75
912	Bloody Bay North	Tobago	Trin&Tob -Tobago	1-Nov-05	11.307	-60.632	Day, Owen	12		55.6%			9.75	9.75
913	Bloody Bay North	Tobago	Trin&Tob -Tobago	1-Nov-05	11.306	-60.632	Day, Owen	12		100.0%			9.75	9.75
914	Bloody Bay South	Tobago	Trin&Tob -Tobago	1-Nov-05	11.306	-60.631	Day, Owen	7		100.0%			9.75	9.75
915	Englishman's Bay North	Tobago	Trin&Tob -Tobago	1-Nov-05	11.295	-60.672	Day, Owen	7		70.0%			9.75	9.75
916	Little Englishman's Bay	Tobago	Trin&Tob -Tobago	1-Nov-05	11.291	-60.679	Day, Owen	12		88.9%			9.75	9.75
917	Little Englishman's Bay	Tobago	Trin&Tob -Tobago	1-Nov-05	11.291	-60.679	Day, Owen	7		50.0%			9.75	9.75

Observation Number	Reef or Site Name	Location	Country	Date (DD-MMM-YY)	Latitude (decimal degrees)	Longitude (decimal degrees)	Primary Contributor	Depth (m)	Cover bleached (%)	Colonies bleached (%)	Cover dead (%)	Colonies dead (%)	Observed DHW (°C-weeks)	Maximum DHW (°C-weeks)
918	Sister's Rocks Central	Tobago	Trin&Tob -Tobago	1-Nov-05	11.332	-60.645	Day, Owen	12		80.8%			9.75	9.75
919	Sister's Rocks Central	Tobago	Trin&Tob -Tobago	1-Nov-05	11.332	-60.645	Day, Owen	7		10.5%			9.75	9.75
920	Sister's Rocks West	Tobago	Trin&Tob -Tobago	1-Nov-05	11.332	-60.646	Day, Owen	12		54.2%			9.75	9.75
921	Buccoo Reef Central N	Tobago	Trin&Tob -Tobago	2-Nov-05	11.183	-60.836	Day, Owen	12		75.0%			9.75	9.75
922	Buccoo Reef Central N	Tobago	Trin&Tob -Tobago	2-Nov-05	11.183	-60.836	Day, Owen	7		77.8%			9.75	9.75
923	Buccoo Reef Central S	Tobago	Trin&Tob -Tobago	2-Nov-05	11.180	-60.838	Day, Owen	12		100.0%			9.75	9.75
924	Buccoo Reef Central S	Tobago	Trin&Tob -Tobago	2-Nov-05	11.180	-60.838	Day, Owen	7		75.0%			9.75	9.75
925	Buccoo Reef Coral Gardens	Tobago	Trin&Tob -Tobago	2-Nov-05	11.187	-60.830	Day, Owen	12		97.0%			9.75	9.75
926	Buccoo Reef Coral Gardens	Tobago	Trin&Tob -Tobago	2-Nov-05	11.187	-60.830	Day, Owen	7		6.5%			9.75	9.75
927	Kariwak reef	Tobago	Trin&Tob -Tobago	2-Nov-05	11.155	-60.850	Day, Owen	12		50.0%			9.75	9.75
928	Kariwak reef	Tobago	Trin&Tob -Tobago	2-Nov-05	11.155	-60.850	Day, Owen	12		72.7%			9.75	9.75
929	Kariwak reef	Tobago	Trin&Tob -Tobago	2-Nov-05	11.156	-60.850	Day, Owen	12		83.3%			9.75	9.75
930	Kariwak reef	Tobago	Trin&Tob -Tobago	2-Nov-05	11.156	-60.850	Day, Owen	7		85.0%			9.75	9.75
931	Ketchup reef	Tobago	Trin&Tob -Tobago	2-Nov-05	11.164	-60.850	Day, Owen	7		16.7%			9.75	9.75
932	Bucoo Reef North East	Tobago	Trin&Tob -Tobago	3-Nov-05	11.193	-60.826	Day, Owen	12		56.0%			9.75	9.75
933	Bucoo Reef North East	Tobago	Trin&Tob -Tobago	3-Nov-05	11.192	-60.826	Day, Owen	7		75.0%			9.75	9.75
934	Bucoo Reef North East	Tobago	Trin&Tob -Tobago	3-Nov-05	11.193	-60.827	Day, Owen	12		62.5%			9.75	9.75
935	Bucoo Reef North East	Tobago	Trin&Tob -Tobago	3-Nov-05	11.193	-60.828	Day, Owen	7		100.0%			9.75	9.75
936	Cove Reef	Tobago	Trin&Tob -Tobago	3-Nov-05	11.132	-60.790	Day, Owen	12		78.3%			9.75	9.75
937	Cove Reef	Tobago	Trin&Tob -Tobago	3-Nov-05	11.133	-60.790	Day, Owen	7		22.2%			9.75	9.75
938	Cove Reef	Tobago	Trin&Tob -Tobago	3-Nov-05	11.132	-60.790	Day, Owen	12		100.0%			9.75	9.75
939	Cove Reef	Tobago	Trin&Tob -Tobago	3-Nov-05	11.132	-60.790	Day, Owen	7		83.3%			9.75	9.75
940	Flying Reef	Tobago	Trin&Tob -Tobago	3-Nov-05	11.137	-60.840	Day, Owen	12		73.7%			9.75	9.75
941	Flying Reef	Tobago	Trin&Tob -Tobago	3-Nov-05	11.138	-60.840	Day, Owen	7		77.8%			9.75	9.75
942	Flying Reef	Tobago	Trin&Tob -Tobago	3-Nov-05	11.137	-60.839	Day, Owen	12		100.0%			9.75	9.75
943	Flying Reef	Tobago	Trin&Tob -Tobago	3-Nov-05	11.138	-60.838	Day, Owen	7		100.0%			9.75	9.75
944	King Peter's Bay	Tobago	Trin&Tob -Tobago	4-Nov-05	11.259	-60.725	Day, Owen	12		87.9%			9.15	9.75
945	King Peter's Bay	Tobago	Trin&Tob -Tobago	4-Nov-05	11.259	-60.725	Day, Owen	7		88.2%			9.15	9.75
946	Buccoo Reef North	Tobago	Trin&Tob -Tobago	5-Nov-05	11.194	-60.836	Day, Owen	12		57.9%			9.15	9.75
947	Buccoo Reef North	Tobago	Trin&Tob -Tobago	5-Nov-05	11.193	-60.836	Day, Owen	7		75.0%			9.15	9.75
948	Buccoo West	Tobago	Trin&Tob -Tobago	5-Nov-05	11.186	-60.847	Day, Owen	12		38.9%			9.15	9.75
949	Buccoo West	Tobago	Trin&Tob -Tobago	5-Nov-05	11.185	-60.847	Day, Owen	7		45.5%			9.15	9.75
950	Brother's Rocks East	Tobago	Trin&Tob -Tobago	7-Nov-05	11.324	-60.616	Day, Owen	12		85.7%			8.55	9.75
951	Brother's Rocks East	Tobago	Trin&Tob -Tobago	7-Nov-05	11.325	-60.616	Day, Owen	7		75.0%			8.55	9.75
952	Brother's Rocks West	Tobago	Trin&Tob -Tobago	7-Nov-05	11.324	-60.616	Day, Owen	12		52.0%			8.55	9.75
953	Brother's Rocks West	Tobago	Trin&Tob -Tobago	7-Nov-05	11.325	-60.617	Day, Owen	7		0.0%			8.55	9.75
954	Corvo Point	Tobago	Trin&Tob -Tobago	7-Nov-05	11.330	-60.586	Day, Owen	12		33.3%			8.55	9.75
955	Corvo Point	Tobago	Trin&Tob -Tobago	7-Nov-05	11.329	-60.586	Day, Owen	7		72.7%			8.55	9.75
956	Corvo Point	Tobago	Trin&Tob -Tobago	7-Nov-05	11.330	-60.586	Day, Owen	12		89.5%			8.55	9.75
957	Corvo Point	Tobago	Trin&Tob -Tobago	7-Nov-05	11.329	-60.586	Day, Owen	7		75.0%			8.55	9.75
958	Pirates Bay East	Tobago	Trin&Tob -Tobago	7-Nov-05	11.331	-60.554	Day, Owen	12		90.9%			8.55	9.75
959	Pirates Bay East	Tobago	Trin&Tob -Tobago	7-Nov-05	11.331	-60.553	Day, Owen	7		94.4%			8.55	9.75
960	Pirates Bay West	Tobago	Trin&Tob -Tobago	7-Nov-05	11.331	-60.555	Day, Owen	12		64.3%			8.55	9.75
961	Pirates Bay West	Tobago	Trin&Tob -Tobago	7-Nov-05	11.332	-60.555	Day, Owen	7		64.6%			8.55	9.75
962	Angel Reef North	Tobago	Trin&Tob -Tobago	8-Nov-05	11.302	-60.519	Day, Owen	7		17.4%			8.55	9.75
963	Angel Reef North	Tobago	Trin&Tob -Tobago	8-Nov-05	11.302	-60.520	Day, Owen	12		0.0%			8.55	9.75
964	Angel Reef South	Tobago	Trin&Tob -Tobago	8-Nov-05	11.302	-60.520	Day, Owen	12		39.4%			8.55	9.75
965	Angel Reef South	Tobago	Trin&Tob -Tobago	8-Nov-05	11.302	-60.520	Day, Owen	7		30.6%			8.55	9.75
966	Japanese Gardens East	Tobago	Trin&Tob -Tobago	8-Nov-05	11.297	-60.520	Day, Owen	12		50.0%			8.55	9.75
967	Japanese Gardens East	Tobago	Trin&Tob -Tobago	8-Nov-05	11.297	-60.520	Day, Owen	7		26.3%			8.55	9.75
968	Japanese Gardens West	Tobago	Trin&Tob -Tobago	8-Nov-05	11.297	-60.520	Day, Owen	12		28.6%			8.55	9.75
969	Japanese Gardens West	Tobago	Trin&Tob -Tobago	8-Nov-05	11.297	-60.520	Day, Owen	7		20.0%			8.55	9.75
970	Spiny Colony North	Tobago	Trin&Tob -Tobago	8-Nov-05	11.290	-60.523	Day, Owen	7		100.0%			8.55	9.75
971	Spiny Colony North	Tobago	Trin&Tob -Tobago	8-Nov-05	11.290	-60.523	Day, Owen	12		100.0%			8.55	9.75
972	Spiny Colony South	Tobago	Trin&Tob -Tobago	8-Nov-05	11.290	-60.523	Day, Owen	12		16.7%			8.55	9.75
973	Spiny Colony South	Tobago	Trin&Tob -Tobago	8-Nov-05	11.290	-60.523	Day, Owen	7		12.5%			8.55	9.75
974	Englishman's Bay North	Tobago	Trin&Tob -Tobago	11-Nov-05	11.294	-60.672	Day, Owen	12		100.0%			7.9	9.75
1718	Fishbowl	South Caicos	Turks&Caic - Caicos	11-Nov-05	21.485	-71.508	Mallela, Jennie	10		88.9%			5.05	5.75

Observation Number	Reef or Site Name	Location	Country	Date (DD-MMM-YY)	Latitude (decimal degrees)	Longitude (decimal degrees)	Primary Contributor	Depth (m)	Cover bleached (%)	Colonies bleached (%)	Cover dead (%)	Colonies dead (%)	Observed DHW (°C-weeks)	Maximum DHW (°C-weeks)
1719	Tuckers Reef	South Caicos	Turks&Caic - Caicos	11-Nov-05	21.485	-71.527	Mallela, Jennie	5		41.2%			5.05	5.75
1720	War Head	South Caicos	Turks&Caic - Caicos	11-Nov-05	21.481	-71.534	Mallela, Jennie	15		31.8%			5.05	5.75
805	Low-relief spur and groove at Sa	Florida Keys	US Florida - Keys	1-Aug-05	24.451	-81.878	Chiappone, Mark	9.6		26.5%			0	1.7
806	Offshore patch reef north of San	Florida Keys	US Florida - Keys	1-Aug-05	24.466	-81.878	Chiappone, Mark	8.7		5.0%			0	1.7
807	Offshore patch reef north of San	Florida Keys	US Florida - Keys	1-Aug-05	24.467	-81.882	Chiappone, Mark	9.45		3.1%			0	1.7
808	Low-relief spur and groove at Sa	Florida Keys	US Florida - Keys	2-Aug-05	24.452	-81.874	Chiappone, Mark	10.65		28.8%			0	1.7
809	Mid-channel patch reef north of	Florida Keys	US Florida - Keys	2-Aug-05	24.485	-81.856	Chiappone, Mark	6.75		5.1%			0	1.7
810	Mid-channel patch reef SE of M	Florida Keys	US Florida - Keys	2-Aug-05	24.480	-81.877	Chiappone, Mark	8.85		7.0%			0	1.7
811	Low-relief hard-bottom at Vesta	Florida Keys	US Florida - Keys	3-Aug-05	24.451	-81.983	Chiappone, Mark	9.45		7.6%			0	1.7
812	Mid-channel patch reef north of	Florida Keys	US Florida - Keys	3-Aug-05	24.483	-81.956	Chiappone, Mark	5.1		10.1%			0	1.7
813	Mid-channel patch reef north of	Florida Keys	US Florida - Keys	3-Aug-05	24.476	-81.952	Chiappone, Mark	4.65		6.4%			0	1.7
814	Mid-channel patch reef north of	Florida Keys	US Florida - Keys	4-Aug-05	24.474	-81.899	Chiappone, Mark	6.6		8.6%			0	1.7
815	Mid-channel patch reef north of	Florida Keys	US Florida - Keys	4-Aug-05	24.482	-81.895	Chiappone, Mark	4.05		6.0%			0	1.7
816	Patchy hard-bottom at Satan Sho	Florida Keys	US Florida - Keys	4-Aug-05	24.446	-81.863	Chiappone, Mark	9.3		10.3%			0	1.7
817	Mid-channel patch reef north of	Florida Keys	US Florida - Keys	5-Aug-05	24.460	-81.914	Chiappone, Mark	8.25		6.6%			0	1.7
818	Offshore patch reef west of Key	Florida Keys	US Florida - Keys	5-Aug-05	24.476	-81.814	Chiappone, Mark	8.55		7.9%			0	1.7
819	Patchy hard-bottom north of San	Florida Keys	US Florida - Keys	5-Aug-05	24.455	-81.919	Chiappone, Mark	9.75		16.7%			0	1.7
820	Mid-channel patch reef north of	Florida Keys	US Florida - Keys	6-Aug-05	24.521	-81.662	Chiappone, Mark	4.8		11.2%			0	3.4
821	Mid-channel patch reef north of	Florida Keys	US Florida - Keys	6-Aug-05	24.523	-81.659	Chiappone, Mark	4.8		4.7%			0	3.4
822	Low-relief hard-bottom east of F	Florida Keys	US Florida - Keys	8-Aug-05	24.496	-81.645	Chiappone, Mark	5.55		14.8%			0	1.15
823	Mid-channel patch reef north of	Florida Keys	US Florida - Keys	8-Aug-05	24.528	-81.645	Chiappone, Mark	4.65		5.6%			0.5	3.4
824	Patchy hard-bottom west of Peli	Florida Keys	US Florida - Keys	8-Aug-05	24.497	-81.651	Chiappone, Mark	8.7		12.5%			0	1.15
58	French Reef	Florida Keys	US Florida - Keys	9-Aug-05	25.034	-80.350	Brandt, Marilyn	6.8		9.5%	0.0%	0.0%	1.85	4.5
59	Little Grecian	Florida Keys	US Florida - Keys	9-Aug-05	25.119	-80.300	Brandt, Marilyn	3.2		1.7%	0.0%	0.0%	1.85	4.5
825	Mid-channel patch reef north of	Florida Keys	US Florida - Keys	9-Aug-05	24.539	-81.626	Chiappone, Mark	6		11.2%			0.5	3.4
826	Mid-channel patch reef north of	Florida Keys	US Florida - Keys	9-Aug-05	24.525	-81.625	Chiappone, Mark	6.6		12.4%			0.5	3.4
827	Patchy hard-bottom east of Rock	Florida Keys	US Florida - Keys	9-Aug-05	24.469	-81.822	Chiappone, Mark	8.55		14.9%			0	1.7
828	Deep reef slope offshore of Peli	Florida Keys	US Florida - Keys	10-Aug-05	24.504	-81.582	Chiappone, Mark	24.45		11.7%			0.5	3.4
829	Mid-channel patch reef north of	Florida Keys	US Florida - Keys	10-Aug-05	24.537	-81.615	Chiappone, Mark	4.95		13.4%			0.5	3.4
830	Mid-channel patch reef north of	Florida Keys	US Florida - Keys	10-Aug-05	24.541	-81.595	Chiappone, Mark	5.25		8.3%			0.5	3.4
60	Cheeca	Florida Keys	US Florida - Keys	11-Aug-05	24.897	-80.616	Brandt, Marilyn	2.8		3.5%	0.0%	0.0%	0.5	3.4
61	Coral Gardens	Florida Keys	US Florida - Keys	11-Aug-05	24.836	-80.728	Brandt, Marilyn	3.2		5.2%	0.0%	0.0%	0.5	3.4
831	High-relief spur and groove at M	Florida Keys	US Florida - Keys	11-Aug-05	24.489	-81.675	Chiappone, Mark	3.3		14.9%			0	1.15
832	High-relief spur and groove at P	Florida Keys	US Florida - Keys	11-Aug-05	24.501	-81.629	Chiappone, Mark	2.7		31.3%			0.5	3.4
833	Low-relief hard-bottom at Mary	Florida Keys	US Florida - Keys	11-Aug-05	24.524	-81.574	Chiappone, Mark	5.4		10.8%			0.5	3.4
834	Mid-channel patch reef at West	Florida Keys	US Florida - Keys	11-Aug-05	24.556	-81.583	Chiappone, Mark	5.7		17.9%			0.5	3.4
835	Mid-channel patch reef inshore o	Florida Keys	US Florida - Keys	12-Aug-05	24.564	-81.471	Chiappone, Mark	7.2		16.1%			1.1	3.4
836	High-relief spur and groove at A	Florida Keys	US Florida - Keys	13-Aug-05	24.522	-81.522	Chiappone, Mark	5.85		41.1%			1.1	3.4
837	High-relief spur and groove at A	Florida Keys	US Florida - Keys	13-Aug-05	24.523	-81.521	Chiappone, Mark	5.25		20.7%			1.1	3.4
838	Mid-channel patch reef inshore o	Florida Keys	US Florida - Keys	13-Aug-05	24.548	-81.533	Chiappone, Mark	5.1		10.0%			1.1	3.4
839	Low-relief hard-bottom at Carys	Florida Keys	US Florida - Keys	15-Aug-05	25.221	-80.209	Chiappone, Mark	8.55		31.9%			2.4	4.5
840	Mid-channel patch reef at Basin	Florida Keys	US Florida - Keys	15-Aug-05	25.240	-80.256	Chiappone, Mark	2.85		34.0%			2.4	4.5
62	Cheeca	Florida Keys	US Florida - Keys	16-Aug-05	24.897	-80.616	Brandt, Marilyn	2.8		8.6%	0.0%	0.0%	1.1	3.4
63	Coral Gardens	Florida Keys	US Florida - Keys	16-Aug-05	24.836	-80.728	Brandt, Marilyn	3.2		9.7%	0.0%	0.0%	1.1	3.4
841	Low-relief hard-bottom near SW	Florida Keys	US Florida - Keys	16-Aug-05	25.304	-80.167	Chiappone, Mark	8.55		27.3%			2.4	4.5
842	Low-relief hard-bottom near SW	Florida Keys	US Florida - Keys	16-Aug-05	25.309	-80.159	Chiappone, Mark	11.4		36.8%			2.4	4.5
843	Offshore patch reef SW of Pacif	Florida Keys	US Florida - Keys	16-Aug-05	25.345	-80.163	Chiappone, Mark	4.35		20.4%			2.4	4.5
64	French Reef	Florida Keys	US Florida - Keys	17-Aug-05	25.034	-80.350	Brandt, Marilyn	6.8		16.9%	0.0%	0.0%	2.4	4.5
65	Little Grecian	Florida Keys	US Florida - Keys	17-Aug-05	25.119	-80.300	Brandt, Marilyn	3.2		2.2%	0.0%	0.0%	2.4	4.5
844	Low-relief hard-bottom near SW	Florida Keys	US Florida - Keys	17-Aug-05	25.321	-80.155	Chiappone, Mark	10.8		40.0%			2.4	4.5
845	Mid-channel patch reef SW of P	Florida Keys	US Florida - Keys	17-Aug-05	25.349	-80.181	Chiappone, Mark	3.3		33.7%			2.4	4.5
846	Low-relief hard-bottom inshore	Florida Keys	US Florida - Keys	18-Aug-05	25.362	-80.154	Chiappone, Mark	5.85		62.3%			2.4	4.5
847	Low-relief spur and groove near	Florida Keys	US Florida - Keys	18-Aug-05	25.321	-80.153	Chiappone, Mark	17.1		46.8%			2.4	4.5
848	Mid-channel patch reef inshore o	Florida Keys	US Florida - Keys	18-Aug-05	25.351	-80.165	Chiappone, Mark	6.15		36.0%			2.4	4.5
1432	A1037	Offshore Patch Reef, Lower Key	US Florida - Keys	18-Aug-05	24.555	-81.435	Kramer, Phil	5.6		36.4%	0.1%		1.1	3.4
1433	A1053	Inshore, Lower Keys	US Florida - Keys	18-Aug-05	24.620	-81.370	Kramer, Phil	2.7		70.0%	0.0%		1.1	3.4
849	Low-relief hard-bottom SW of P	Florida Keys	US Florida - Keys	19-Aug-05	25.332	-80.181	Chiappone, Mark	3.75		52.0%			2.95	4.5
850	Low-relief spur and groove nort	Florida Keys	US Florida - Keys	19-Aug-05	25.495	-80.104	Chiappone, Mark	16.95		46.0%			2.95	4.5

Observation Number	Reef or Site Name	Location	Country	Date (DD-MMM-YY)	Latitude (decimal degrees)	Longitude (decimal degrees)	Primary Contributor	Depth (m)	Cover bleached (%)	Colonies bleached (%)	Cover dead (%)	Colonies dead (%)	Observed DHW (°C-weeks)	Maximum DHW (°C-weeks)
851	Offshore patch reef SW of Triun	Florida Keys	US Florida - Keys	19-Aug-05	25.462	-80.133	Chiappone, Mark	5.85		55.0%			2.95	4.5
852	Offshore patch reef SW of Triun	Florida Keys	US Florida - Keys	19-Aug-05	25.459	-80.137	Chiappone, Mark	3.75		65.0%			2.95	4.5
1434	A1041	Reef Margin, Lower Keys	US Florida - Keys	19-Aug-05	24.562	-81.340	Kramer, Phil	8.5		39.6%	0.4%		1.85	3.4
1435	A1043	Reef Margin, Lower Keys	US Florida - Keys	19-Aug-05	24.557	-81.350	Kramer, Phil	14.7		37.5%	0.0%		1.85	3.4
1436	A1046	Offshore Patch Reef, Lower Key	US Florida - Keys	19-Aug-05	24.548	-81.426	Kramer, Phil	10.2		29.6%	0.2%		1.85	3.4
853	High-relief spur and groove at F	Florida Keys	US Florida - Keys	21-Aug-05	25.593	-80.096	Chiappone, Mark	6.45		79.4%			4.1	5.7
854	Low-relief spur and groove at Fo	Florida Keys	US Florida - Keys	21-Aug-05	25.598	-80.094	Chiappone, Mark	9.6		54.3%			4.1	5.7
855	Low-relief spur and groove at Fo	Florida Keys	US Florida - Keys	21-Aug-05	25.589	-80.096	Chiappone, Mark	3.3		49.0%			4.1	5.7
856	Mid-channel patch reef inshore o	Florida Keys	US Florida - Keys	22-Aug-05	25.435	-80.136	Chiappone, Mark	4.05		59.5%			3.5	4.5
857	Midchannel patch reef on northe	Florida Keys	US Florida - Keys	22-Aug-05	25.523	-80.135	Chiappone, Mark	6.75		70.7%			3.5	4.5
858	Offshore patch reef inshore of S	Florida Keys	US Florida - Keys	22-Aug-05	25.507	-80.120	Chiappone, Mark	5.55		72.6%			3.5	4.5
1446	A1146	Reef Margin, Upper Keys Trans	US Florida - Keys	22-Aug-05	25.402	-80.132	Kramer, Phil	2.5		17.7%	0.0%		3.5	4.5
1447	A1151	Reef Margin, Upper Keys Trans	US Florida - Keys	22-Aug-05	25.388	-80.133	Kramer, Phil	9		29.0%	3.2%		3.5	4.5
1448	A1154	Reef Margin, Upper Keys Trans	US Florida - Keys	22-Aug-05	25.579	-80.092	Kramer, Phil	14		34.0%	0.0%		4.1	5.7
1449	A1157	Reef Margin, Upper Keys Trans	US Florida - Keys	22-Aug-05	25.376	-80.139	Kramer, Phil	10		80.0%	0.0%		3.5	4.5
1450	A1158	Reef Margin, Upper Keys Trans	US Florida - Keys	22-Aug-05	25.604	-80.094	Kramer, Phil	6.7		63.3%	0.0%		4.1	5.7
1452	A4005	Reef Margin, Upper Keys Trans	US Florida - Keys	22-Aug-05	25.458	-80.131	Kramer, Phil	6		71.7%	0.0%		3.5	4.5
1453	A4008	Mid Channel, Upper Keys Trans	US Florida - Keys	22-Aug-05	25.399	-80.161	Kramer, Phil	3		69.4%	0.1%		3.5	4.5
1714	3162	Florida Keys	US Florida - Keys	22-Aug-05	25.640	-80.333	Lirman, Diego	3		69.4%			4.1	5.7
1715	3165	Florida Keys	US Florida - Keys	22-Aug-05	25.583	-80.360	Lirman, Diego	6		71.7%			4.1	5.7
859	Low-relief hard-bottom at Star R	Florida Keys	US Florida - Keys	23-Aug-05	25.517	-80.112	Chiappone, Mark	6.15		78.0%			3.5	4.5
860	Low-relief spur and groove at Tr	Florida Keys	US Florida - Keys	23-Aug-05	25.477	-80.111	Chiappone, Mark	4.95		71.1%			3.5	4.5
861	Mid-channel patch reef at Marg	Florida Keys	US Florida - Keys	23-Aug-05	25.445	-80.154	Chiappone, Mark	3.6		65.6%			3.5	4.5
862	Patchy hard-bottom inshore of S	Florida Keys	US Florida - Keys	23-Aug-05	25.511	-80.116	Chiappone, Mark	7.35		95.8%			3.5	4.5
1716	3147	Florida Keys	US Florida - Keys	23-Aug-05	25.700	-80.097	Lirman, Diego	8.5		43.8%			4.1	5.7
1717	3150	Florida Keys	US Florida - Keys	23-Aug-05	25.804	-80.335	Lirman, Diego	10		35.4%			4.1	5.7
863	Low-relief hard-bottom at Long	Florida Keys	US Florida - Keys	24-Aug-05	25.424	-80.126	Chiappone, Mark	3.3		25.0%			3.5	4.5
864	Mid-channel patch reef southeas	Florida Keys	US Florida - Keys	24-Aug-05	25.423	-80.151	Chiappone, Mark	2.55		60.4%			3.5	4.5
1469	A1048	Mid Channel, Lower Keys	US Florida - Keys	24-Aug-05	24.492	-81.888	Kramer, Phil	6.8		50.0%	1.2%		1.7	1.7
1470	A1050	Offshore Patch Reef, Lower Key	US Florida - Keys	24-Aug-05	24.477	-81.893	Kramer, Phil	5		23.2%	0.6%		1.7	1.7
1471	A1052	Mid Channel, Lower Keys	US Florida - Keys	24-Aug-05	24.478	-81.957	Kramer, Phil	8.6		29.2%	0.1%		1.7	1.7
1472	A1097	Offshore Patch Reef, Middle Ke	US Florida - Keys	24-Aug-05	24.618	-81.190	Kramer, Phil	7.6		61.9%	0.6%		2.4	3.4
1473	A1099	Reef Margin, Middle Keys	US Florida - Keys	24-Aug-05	24.630	-81.083	Kramer, Phil	15.4		66.7%	0.0%		2.4	3.4
1474	A1104	Mid Channel, Middle Keys	US Florida - Keys	24-Aug-05	24.625	-81.235	Kramer, Phil	3.9		75.9%	3.8%		2.4	3.4
1475	A1135	Mid Channel, Upper Keys	US Florida - Keys	31-Aug-05	25.036	-80.403	Kramer, Phil	3		59.4%	0.0%		4.5	4.5
1476	A1137	Inshore, Upper Keys	US Florida - Keys	31-Aug-05	25.109	-80.338	Kramer, Phil	2.8		62.7%	0.0%		4.5	4.5
1477	A1145	Reef Margin, Upper Keys Trans	US Florida - Keys	31-Aug-05	25.423	-80.125	Kramer, Phil	3.7		40.0%	0.0%		4.5	4.5
1478	A2159	Mid Channel, Upper Keys Trans	US Florida - Keys	31-Aug-05	25.410	-80.157	Kramer, Phil	2.4		58.6%	2.8%		4.5	4.5
66	Cheeca	Florida Keys	US Florida - Keys	1-Sep-05	24.897	-80.616	Brandt, Marilyn	2.8		76.1%	0.0%	0.0%	3.4	3.4
67	Coral Gardens	Florida Keys	US Florida - Keys	1-Sep-05	24.836	-80.728	Brandt, Marilyn	3.2		79.0%	0.0%	0.0%	3.4	3.4
1485	A1149	Reef Margin, Upper Keys Trans	US Florida - Keys	1-Sep-05	25.577	-80.101	Kramer, Phil	6.1		83.9%	0.0%		5.7	5.7
1486	A1153	Reef Margin, Upper Keys Trans	US Florida - Keys	1-Sep-05	25.540	-80.096	Kramer, Phil	21.3		33.3%	0.0%		4.5	4.5
68	French Reef	Florida Keys	US Florida - Keys	2-Sep-05	25.034	-80.350	Brandt, Marilyn	6.8		48.3%	0.0%	0.0%	4.5	4.5
69	Little Grecian	Florida Keys	US Florida - Keys	2-Sep-05	25.119	-80.300	Brandt, Marilyn	3.2		11.7%	0.0%	0.0%	4.5	4.5
2426	Marker 9 Reef	N. Florida Keys	US Florida - Keys	5-Sep-05	25.497	-80.147	Smith, Tyler	3		81.8%		0.0%	4.5	4.5
2427	Marker 9 Reef	N. Florida Keys	US Florida - Keys	5-Sep-05	25.497	-80.147	Smith, Tyler	3		80.0%		0.0%	4.5	4.5
2428	Marker 9 Reef	N. Florida Keys	US Florida - Keys	5-Sep-05	25.497	-80.147	Smith, Tyler	3		100.0%		0.0%	4.5	4.5
2429	Palythoa Reef	N. Florida Keys	US Florida - Keys	5-Sep-05	25.495	-80.122	Smith, Tyler	3		100.0%		0.0%	4.5	4.5
2430	Palythoa Reef	N. Florida Keys	US Florida - Keys	5-Sep-05	25.495	-80.122	Smith, Tyler	3		75.0%		0.0%	4.5	4.5
2431	Site 3	N. Florida Keys	US Florida - Keys	5-Sep-05	25.468	-80.142	Smith, Tyler	3		100.0%		0.0%	4.5	4.5
2432	Site 3	N. Florida Keys	US Florida - Keys	5-Sep-05	25.468	-80.142	Smith, Tyler	3		100.0%		0.0%	4.5	4.5
2433	Site 3	N. Florida Keys	US Florida - Keys	5-Sep-05	25.468	-80.142	Smith, Tyler	3		88.9%		0.0%	4.5	4.5
70	Cheeca	Florida Keys	US Florida - Keys	6-Sep-05	24.897	-80.616	Brandt, Marilyn	2.8		82.3%	0.0%	0.0%	3.4	3.4
71	Coral Gardens	Florida Keys	US Florida - Keys	6-Sep-05	24.836	-80.728	Brandt, Marilyn	3.2		92.0%	0.0%	0.0%	3.4	3.4
1490	A1138	Offshore Patch Reef, Upper Key	US Florida - Keys	6-Sep-05	25.009	-80.447	Kramer, Phil	3		59.5%	0.0%		4.5	4.5
1491	A1141	Offshore Patch Reef, Upper Key	US Florida - Keys	6-Sep-05	25.034	-80.380	Kramer, Phil	5.5		34.6%	0.3%		4.5	4.5
72	French Reef	Florida Keys	US Florida - Keys	7-Sep-05	25.034	-80.350	Brandt, Marilyn	6.8		72.0%	0.0%	0.0%	4.5	4.5
73	Little Grecian	Florida Keys	US Florida - Keys	7-Sep-05	25.119	-80.300	Brandt, Marilyn	3.2		19.5%	0.0%	0.0%	4.5	4.5

Observation Number	Reef or Site Name	Location	Country	Date (DD-MMM-YY)	Latitude (decimal degrees)	Longitude (decimal degrees)	Primary Contributor	Depth (m)	Cover bleached (%)	Colonies bleached (%)	Cover dead (%)	Colonies dead (%)	Observed DHW (°C-weeks)	Maximum DHW (°C-weeks)
1492	A1142	Offshore Patch Reef, Upper Key	US Florida - Keys	7-Sep-05	25.008	-80.424	Kramer, Phil	4		56.3%	0.3%		4.5	4.5
1493	A1029	Reef Margin, Lower Keys	US Florida - Keys	8-Sep-05	24.521	-81.521	Kramer, Phil	9.2		40.0%	0.8%		3.4	3.4
1494	A1035	Offshore Patch Reef, Lower Key	US Florida - Keys	8-Sep-05	24.550	-81.440	Kramer, Phil	7.6		49.2%	1.1%		3.4	3.4
1495	A1044	Reef Margin, Lower Keys	US Florida - Keys	8-Sep-05	24.536	-81.457	Kramer, Phil	12.5		57.6%	0.3%		3.4	3.4
1496	A1045	Reef Margin, Lower Keys	US Florida - Keys	8-Sep-05	24.532	-81.479	Kramer, Phil	10.3		18.4%	0.6%		3.4	3.4
865	High-relief spur and groove at A	Florida Keys	US Florida - Keys	9-Sep-05	25.410	-80.129	Chiappone, Mark	4.65		65.6%			4.5	4.5
866	Mid-channel patch reef northeas	Florida Keys	US Florida - Keys	9-Sep-05	25.408	-80.166	Chiappone, Mark	4.65		66.1%			4.5	4.5
867	Mid-channel patch reef south of	Florida Keys	US Florida - Keys	9-Sep-05	25.425	-80.161	Chiappone, Mark	4.05		51.4%			4.5	4.5
868	Patchy hard-bottom inshore of L	Florida Keys	US Florida - Keys	9-Sep-05	25.449	-80.121	Chiappone, Mark	7.35		88.2%			4.5	4.5
2128	Looe Key Reef	Lower Florida Keys	US Florida - Keys	9-Sep-05	24.623	-81.400	Ritchie, Kim	15	35.0%				3.4	3.4
869	Mid-channel patch reef inshore o	Florida Keys	US Florida - Keys	10-Sep-05	25.403	-80.152	Chiappone, Mark	6.2		78.2%			4.5	4.5
870	Mid-channel patch reef southeas	Florida Keys	US Florida - Keys	10-Sep-05	25.365	-80.189	Chiappone, Mark	4.05		61.3%			4.5	4.5
2129	Molasses Reef	Upper Keys regions	US Florida - Keys	10-Sep-05	25.007	-80.373	Ritchie, Kim	10	35.0%				4.5	4.5
2130	White Banks	Upper Keys	US Florida - Keys	10-Sep-05	25.040	-80.382	Ritchie, Kim	15	35.0%				4.5	4.5
1498	A1034	Reef Margin, Lower Keys	US Florida - Keys	12-Sep-05	24.452	-81.883	Kramer, Phil	10.2		34.1%	0.2%		1.7	1.7
1499	A1120	Reef Margin, Upper Keys	US Florida - Keys	12-Sep-05	25.205	-80.223	Kramer, Phil	5.5		43.9%	0.4%		4.5	4.5
1500	A1030	Reef Margin, Lower Keys	US Florida - Keys	13-Sep-05	24.501	-81.632	Kramer, Phil	2.2		57.1%	0.0%		3.4	3.4
1501	A1036	Offshore Patch Reef, Lower Key	US Florida - Keys	13-Sep-05	24.514	-81.608	Kramer, Phil	6.7		31.6%	0.4%		3.4	3.4
1502	A1038	Offshore Patch Reef, Lower Key	US Florida - Keys	13-Sep-05	24.514	-81.619	Kramer, Phil	2.8		33.3%	0.0%		3.4	3.4
1503	A1049	Offshore Patch Reef, Lower Key	US Florida - Keys	13-Sep-05	24.505	-81.721	Kramer, Phil	5.7		35.9%	0.0%		3.4	3.4
1504	A3015	Mid Channel, Lower Keys	US Florida - Keys	13-Sep-05	24.548	-81.586	Kramer, Phil	3.9		47.6%	0.1%		3.4	3.4
1505	A3018	Reef Margin, Lower Keys	US Florida - Keys	13-Sep-05	24.479	-81.717	Kramer, Phil	8.3		43.6%	0.9%		1.15	1.15
1506	A1096	Offshore Patch Reef, Middle Ke	US Florida - Keys	16-Sep-05	24.652	-81.019	Kramer, Phil	10.3		29.5%	0.0%		3.4	3.4
1507	A1098	Reef Margin, Middle Keys	US Florida - Keys	16-Sep-05	24.631	-81.085	Kramer, Phil	13.8		44.2%	0.1%		3.4	3.4
1508	A1101	Reef Margin, Middle Keys	US Florida - Keys	16-Sep-05	24.649	-81.031	Kramer, Phil	9.7		46.4%	0.0%		3.4	3.4
1509	A1130	Reef Margin, Upper Keys	US Florida - Keys	16-Sep-05	25.133	-80.262	Kramer, Phil	17.5		19.5%	0.3%		4.5	4.5
1510	A1132.1	Reef Margin, Upper Keys	US Florida - Keys	16-Sep-05	25.005	-80.387	Kramer, Phil	12		27.3%	1.8%		4.5	4.5
1511	A3012	Mid Channel, Middle Keys	US Florida - Keys	16-Sep-05	24.690	-81.030	Kramer, Phil	3.1		74.1%	0.2%		3.4	3.4
1512	A3013	Reef Margin, Middle Keys	US Florida - Keys	16-Sep-05	24.623	-81.111	Kramer, Phil	14.2		50.0%	0.2%		3.4	3.4
871	High-relief spur and groove at L	Florida Keys	US Florida - Keys	17-Sep-05	24.546	-81.406	Chiappone, Mark	4.65		48.2%			3.4	3.4
872	Low-relief hard-bottom west of	Florida Keys	US Florida - Keys	17-Sep-05	24.540	-81.443	Chiappone, Mark	7.95		45.1%			3.4	3.4
2434	Anniversary Reef	N. Florida Keys	US Florida - Keys	17-Sep-05	25.398	-80.161	Smith, Tyler	3		47.4%		0.0%	4.5	4.5
2435	Anniversary Reef	N. Florida Keys	US Florida - Keys	17-Sep-05	25.398	-80.161	Smith, Tyler	3		63.6%		0.0%	4.5	4.5
2436	Bache Shoal	N. Florida Keys	US Florida - Keys	17-Sep-05	25.486	-80.149	Smith, Tyler	3		88.9%		0.0%	4.5	4.5
2437	Bache Shoal	N. Florida Keys	US Florida - Keys	17-Sep-05	25.486	-80.149	Smith, Tyler	3		100.0%		0.0%	4.5	4.5
2438	Dome Reef	N. Florida Keys	US Florida - Keys	17-Sep-05	25.447	-80.160	Smith, Tyler	3		100.0%		0.0%	4.5	4.5
2439	Dome Reef	N. Florida Keys	US Florida - Keys	17-Sep-05	25.447	-80.160	Smith, Tyler	3		85.7%		0.0%	4.5	4.5
2440	Dome Reef	N. Florida Keys	US Florida - Keys	17-Sep-05	25.447	-80.160	Smith, Tyler	3		33.3%		0.0%	4.5	4.5
2441	Fowey Rocks	N. Florida Keys	US Florida - Keys	17-Sep-05	25.587	-80.096	Smith, Tyler	3		50.0%		0.0%	5.7	5.7
2442	Fowey Rocks	N. Florida Keys	US Florida - Keys	17-Sep-05	25.587	-80.096	Smith, Tyler	3		80.0%		0.0%	5.7	5.7
2443	Fowey Rocks	N. Florida Keys	US Florida - Keys	17-Sep-05	25.587	-80.096	Smith, Tyler	3		66.7%		0.0%	5.7	5.7
873	Mid-channel patch reef inshore o	Florida Keys	US Florida - Keys	18-Sep-05	24.584	-81.394	Chiappone, Mark	9.15		29.9%			3.4	3.4
1513	A1031	Reef Margin, Lower Keys	US Florida - Keys	27-Sep-05	24.445	-81.923	Kramer, Phil	8.8		30.6%	0.0%		1.7	1.7
1514	A1032	Reef Margin, Lower Keys	US Florida - Keys	27-Sep-05	24.445	-81.923	Kramer, Phil	6.5		23.8%	0.3%		1.7	1.7
1515	A1042	Reef Margin, Lower Keys	US Florida - Keys	27-Sep-05	24.438	-81.903	Kramer, Phil	14.8		18.8%	0.4%		1.7	1.7
1516	A1047	Reef Margin, Lower Keys	US Florida - Keys	27-Sep-05	24.447	-81.903	Kramer, Phil	13.3		32.7%	0.4%		1.7	1.7
1517	A1055	Offshore Patch Reef, Lower Key	US Florida - Keys	27-Sep-05	24.458	-81.895	Kramer, Phil	10.3		36.6%	0.5%		1.7	1.7
1518	A1057	Offshore Patch Reef, Lower Key	US Florida - Keys	27-Sep-05	24.460	-81.907	Kramer, Phil	9.9		21.9%	0.2%		1.7	1.7
1519	A1121	Reef Margin, Upper Keys	US Florida - Keys	28-Sep-05	24.991	-80.411	Kramer, Phil	6.1		64.3%	0.0%		4.5	4.5
1520	A1122	Reef Margin, Upper Keys	US Florida - Keys	28-Sep-05	24.986	-80.415	Kramer, Phil	6.2		74.4%	0.0%		4.5	4.5
1521	A1132.2	Reef Margin, Upper Keys	US Florida - Keys	28-Sep-05	25.005	-80.387	Kramer, Phil	10.6		66.7%	0.0%		4.5	4.5
1522	A1143	Offshore Patch Reef, Upper Key	US Florida - Keys	28-Sep-05	25.053	-80.362	Kramer, Phil	3.6		63.9%	0.0%		4.5	4.5
1523	A1088	Reef Margin, Upper Keys	US Florida - Keys	29-Sep-05	24.851	-80.618	Kramer, Phil	3.6		42.9%	0.0%		3.4	3.4
1524	A1090	Reef Margin, Upper Keys	US Florida - Keys	29-Sep-05	24.823	-80.657	Kramer, Phil	8.5		68.8%	0.0%		3.4	3.4
1525	A1091	Offshore Patch Reef, Upper Key	US Florida - Keys	29-Sep-05	24.917	-80.545	Kramer, Phil	6		50.0%	0.0%		3.4	3.4
1526	A1093	Reef Margin, Upper Keys	US Florida - Keys	29-Sep-05	24.899	-80.550	Kramer, Phil	8.7		29.4%	8.3%		3.4	3.4
1527	A1106	Inshore, Upper Keys	US Florida - Keys	29-Sep-05	24.906	-80.613	Kramer, Phil	3.9		100.0%	0.0%		3.4	3.4
874	Low-relief hard-bottom at Tenn	Florida Keys	US Florida - Keys	30-Sep-05	24.768	-80.750	Chiappone, Mark	7.2		13.0%			3.4	3.4

Observation Number	Reef or Site Name	Location	Country	Date (DD-MMM-YY)	Latitude (decimal degrees)	Longitude (decimal degrees)	Primary Contributor	Depth (m)	Cover bleached (%)	Colonies bleached (%)	Cover dead (%)	Colonies dead (%)	Observed DHW (°C-weeks)	Maximum DHW (°C-weeks)
875	Low-relief hard-bottom at Tenn	Florida Keys	US Florida - Keys	30-Sep-05	24.766	-80.754	Chiappone, Mark	7.5		16.7%			3.4	3.4
876	Low-relief spur and groove east	Florida Keys	US Florida - Keys	30-Sep-05	24.860	-80.597	Chiappone, Mark	11.25		29.0%			3.4	3.4
877	Low-relief spur and groove at C	Florida Keys	US Florida - Keys	1-Oct-05	24.696	-80.936	Chiappone, Mark	4.8		15.0%			3.4	3.4
878	Low-relief spur and groove offs	Florida Keys	US Florida - Keys	1-Oct-05	24.623	-81.108	Chiappone, Mark	15.45		30.0%			3.4	3.4
879	Mid-channel patch reef at West	Florida Keys	US Florida - Keys	1-Oct-05	24.702	-80.965	Chiappone, Mark	4.35		23.4%			3.4	3.4
880	Low-relief hard-bottom southwe	Florida Keys	US Florida - Keys	2-Oct-05	24.722	-80.839	Chiappone, Mark	7.2		26.5%			3.4	3.4
881	Low-relief spur and groove sout	Florida Keys	US Florida - Keys	2-Oct-05	24.726	-80.825	Chiappone, Mark	10.2		100.0%			3.4	3.4
74	French Reef	Florida Keys	US Florida - Keys	8-Nov-05	25.034	-80.350	Brandt, Marilyn	6.1		11.7%	0.0%	0.0%	2.1	4.5
75	French Reef	Florida Keys	US Florida - Keys	8-Nov-05	25.034	-80.350	Brandt, Marilyn	6.8		32.3%	0.0%	0.0%	2.1	4.5
76	Little Grecian	Florida Keys	US Florida - Keys	8-Nov-05	25.119	-80.300	Brandt, Marilyn	3.5		0.0%	0.0%	0.0%	2.1	4.5
77	Little Grecian	Florida Keys	US Florida - Keys	8-Nov-05	25.119	-80.300	Brandt, Marilyn	3.2		6.1%	0.0%	0.0%	2.1	4.5
78	Cheeca	Florida Keys	US Florida - Keys	9-Nov-05	24.897	-80.616	Brandt, Marilyn	2.8		32.0%	0.0%	0.0%	2.3	3.4
79	Cheeca	Florida Keys	US Florida - Keys	9-Nov-05	24.897	-80.616	Brandt, Marilyn	2.8		35.7%	0.0%	0.0%	2.3	3.4
80	Coral Gardens	Florida Keys	US Florida - Keys	2-Dec-05	24.836	-80.728	Brandt, Marilyn	3.5		19.0%	0.0%	0.0%	0	3.4
81	Coral Gardens	Florida Keys	US Florida - Keys	2-Dec-05	24.836	-80.728	Brandt, Marilyn	3.2		32.5%	0.0%	0.0%	0	3.4
1528	B2045	Reef Margin, Lower Keys	US Florida - Keys	23-Jan-06	24.438	-81.893	Kramer, Phil	16.2		2.9%	0.2%		0	1.7
1529	B2030	Reef Margin, Lower Keys	US Florida - Keys	24-Jan-06	24.492	-81.659	Kramer, Phil	7.7		3.7%	0.0%		0	1.15
1530	B2032	Reef Margin, Lower Keys	US Florida - Keys	24-Jan-06	24.494	-81.659	Kramer, Phil	4.4		0.0%	0.0%		0	1.15
1531	B2042	Reef Margin, Lower Keys	US Florida - Keys	24-Jan-06	24.499	-81.634	Kramer, Phil	5.5		0.0%	4.2%		0	1.15
1532	B2043	Reef Margin, Lower Keys	US Florida - Keys	24-Jan-06	24.492	-81.652	Kramer, Phil	14.8		3.9%	0.0%		0	1.15
1533	B2053	Inshore, Lower Keys	US Florida - Keys	24-Jan-06	24.586	-81.580	Kramer, Phil	4.6		6.5%	0.2%		0	3.4
1534	B2054	Inshore, Lower Keys	US Florida - Keys	24-Jan-06	24.564	-81.661	Kramer, Phil	3.4		0.0%	0.0%		0	3.4
1535	B2029	Reef Margin, Lower Keys	US Florida - Keys	31-Jan-06	24.510	-81.564	Kramer, Phil	10.4		0.0%	0.2%		0	3.4
1536	B2041.1	Reef Margin, Lower Keys	US Florida - Keys	31-Jan-06	24.512	-81.560	Kramer, Phil	10.7		6.3%	0.2%		0	3.4
1540	B1152	Reef Margin, Upper Keys Trans	US Florida - Keys	21-Feb-06	25.375	-80.139	Kramer, Phil	13.4		0.0%	0.1%		0	4.5
1541	B1154	Reef Margin, Upper Keys Trans	US Florida - Keys	21-Feb-06	25.582	-80.096	Kramer, Phil	7.6		1.6%	0.0%		0	5.7
1542	B2147	Reef Margin, Upper Keys Trans	US Florida - Keys	21-Feb-06	25.559	-80.106	Kramer, Phil	4.5		0.0%	0.2%		0	4.5
1543	B1145	Reef Margin, Upper Keys Trans	US Florida - Keys	22-Feb-06	25.422	-80.126	Kramer, Phil	3		0.0%	0.0%		0	4.5
1545	B1159	Mid Channel, Upper Keys Trans	US Florida - Keys	22-Feb-06	25.422	-80.154	Kramer, Phil	3.7		0.0%	0.0%		0	4.5
1546	B1165	Reef Margin, Upper Keys Trans	US Florida - Keys	22-Feb-06	25.490	-80.127	Kramer, Phil	7.3		1.9%	0.3%		0	4.5
1547	B2162	Mid Channel, Upper Keys Trans	US Florida - Keys	22-Feb-06	25.386	-80.163	Kramer, Phil	4.6		3.5%	0.1%		0	4.5
1548	B2165	Reef Margin, Upper Keys Trans	US Florida - Keys	22-Feb-06	25.459	-80.130	Kramer, Phil	4.6		4.2%	0.1%		0	4.5
1549	B1034	Reef Margin, Lower Keys	US Florida - Keys	23-Feb-06	24.452	-81.883	Kramer, Phil	9.5		0.0%	0.2%		0	1.7
1550	B2036	Offshore Patch Reef, Lower Key	US Florida - Keys	23-Feb-06	24.457	-81.913	Kramer, Phil	8.5		3.3%	0.2%		0	1.7
1551	B2046	Reef Margin, Lower Keys	US Florida - Keys	23-Feb-06	24.445	-81.943	Kramer, Phil	10.4		3.2%	0.8%		0	1.7
1552	B2051	Mid Channel, Lower Keys	US Florida - Keys	23-Feb-06	24.488	-81.883	Kramer, Phil	8.7		1.6%	0.1%		0	1.7
1553	B2160	Inshore, Upper Keys Trans	US Florida - Keys	23-Feb-06	25.456	-80.172	Kramer, Phil	3.7		8.1%	0.6%		0	4.5
1554	B2163	Inshore, Upper Keys Trans	US Florida - Keys	23-Feb-06	25.434	-80.193	Kramer, Phil	3		5.3%	0.1%		0	4.5
1555	B3160	Inshore, Upper Keys Trans	US Florida - Keys	23-Feb-06	25.464	-80.169	Kramer, Phil	3.7		1.4%	0.1%		0	4.5
1556	B3163	Mid Channel, Upper Keys Trans	US Florida - Keys	23-Feb-06	25.405	-80.169	Kramer, Phil	4.6		9.2%	0.4%		0	4.5
1557	B1038	Offshore Patch Reef, Lower Key	US Florida - Keys	24-Feb-06	24.514	-81.619	Kramer, Phil	3.7		0.0%	0.0%		0	3.4
1558	B1049	Offshore Patch Reef, Lower Key	US Florida - Keys	24-Feb-06	24.505	-81.721	Kramer, Phil	6.1		0.0%	0.0%		0	3.4
1559	B2034	Offshore Patch Reef, Lower Key	US Florida - Keys	24-Feb-06	24.478	-81.832	Kramer, Phil	4.8		7.7%	0.0%		0	1.7
1560	B2035	Offshore Patch Reef, Lower Key	US Florida - Keys	24-Feb-06	24.460	-81.856	Kramer, Phil	6.7		0.0%	0.0%		0	1.7
1561	B2040	Offshore Patch Reef, Lower Key	US Florida - Keys	24-Feb-06	24.476	-81.834	Kramer, Phil	5		0.0%	0.0%		0	1.7
1562	B2039	Offshore Patch Reef, Lower Key	US Florida - Keys	6-Mar-06	24.526	-81.566	Kramer, Phil	8.1		1.6%	0.2%		0	3.4
1563	B2041.2	Reef Margin, Lower Keys	US Florida - Keys	6-Mar-06	24.512	-81.560	Kramer, Phil	10.3		1.5%	0.1%		0	3.4
1564	B3015	Mid Channel, Lower Keys	US Florida - Keys	6-Mar-06	24.547	-81.587	Kramer, Phil	4.9		2.3%	1.1%		0	3.4
1565	B3016	Inshore, Lower Keys	US Florida - Keys	6-Mar-06	24.586	-81.583	Kramer, Phil	3		2.3%	2.8%		0	3.4
1578	C1042	Reef Margin, Lower Keys	US Florida - Keys	22-Aug-06	24.546	-81.402	Kramer, Phil	6.2		9.1%	0.0%		0	3.4
1579	C4107	Offshore Patch Reef, Lower Key	US Florida - Keys	22-Aug-06	24.563	-81.401	Kramer, Phil	6.4		5.3%	0.9%		0	3.4
1580	C4108	Offshore Patch Reef, Lower Key	US Florida - Keys	22-Aug-06	24.553	-81.379	Kramer, Phil	5		15.6%	0.0%		0	3.4
1581	C1035	Mid Channel, Lower Keys	US Florida - Keys	23-Aug-06	24.579	-81.438	Kramer, Phil	7.2		3.8%	0.0%		0	3.4
1582	C1036	Mid Channel, Lower Keys	US Florida - Keys	23-Aug-06	24.582	-81.441	Kramer, Phil	6.1		9.5%	0.0%		0	3.4
1583	C1041	Reef Margin, Lower Keys	US Florida - Keys	23-Aug-06	24.521	-81.525	Kramer, Phil	10		2.9%	0.2%		0	3.4
1584	C1049	Reef Margin, Lower Keys	US Florida - Keys	23-Aug-06	24.515	-81.555	Kramer, Phil	6.8		18.9%	0.5%		0	3.4
1585	C2268	Reef Margin, Lower Keys	US Florida - Keys	23-Aug-06	24.537	-81.437	Kramer, Phil	17.9		12.0%	0.4%		0	3.4
1586	C1015	Reef Margin, Middle Keys	US Florida - Keys	24-Aug-06	24.602	-81.213	Kramer, Phil	7.2		28.6%	0.0%		0	3.4

Observation Number	Reef or Site Name	Location	Country	Date (DD-MMM-YY)	Latitude (decimal degrees)	Longitude (decimal degrees)	Primary Contributor	Depth (m)	Cover bleached (%)	Colonies bleached (%)	Cover dead (%)	Colonies dead (%)	Observed DHW (°C-weeks)	Maximum DHW (°C-weeks)
1587	C1021	Reef Margin, Middle Keys	US Florida - Keys	24-Aug-06	24.595	-81.211	Kramer, Phil	13.7		0.0%	0.0%		0	3.4
1588	C1045	Offshore Patch Reef, Middle Ke	US Florida - Keys	24-Aug-06	24.577	-81.305	Kramer, Phil	8.1		6.3%	0.0%		0	3.4
1589	C1046	Reef Margin, Middle Keys	US Florida - Keys	24-Aug-06	24.591	-81.247	Kramer, Phil	7.5		18.9%	1.0%		0	3.4
1590	C1050	Offshore Patch Reef, Middle Ke	US Florida - Keys	24-Aug-06	24.569	-81.315	Kramer, Phil	10.1		21.2%	0.2%		0	3.4
1592	C1024	Inshore, Lower Keys	US Florida - Keys	25-Aug-06	24.550	-81.703	Kramer, Phil	3.9		0.0%	0.0%		0	3.4
1593	C1030	Mid Channel, Lower Keys	US Florida - Keys	25-Aug-06	24.524	-81.638	Kramer, Phil	6.9		8.2%	0.5%		0	3.4
1594	C1031	Mid Channel, Lower Keys	US Florida - Keys	25-Aug-06	24.527	-81.674	Kramer, Phil	7.5		13.7%	1.0%		0	3.4
1595	C1033	Mid Channel, Lower Keys	US Florida - Keys	25-Aug-06	24.535	-81.605	Kramer, Phil	6.8		8.6%	0.5%		0	3.4
1596	C1039	Offshore Patch Reef, Lower Key	US Florida - Keys	25-Aug-06	24.502	-81.660	Kramer, Phil	6.2		18.9%	0.4%		0	3.4
1597	C1086	Reef Margin, Upper Keys Trans	US Florida - Keys	25-Aug-06	25.588	-80.113	Kramer, Phil	4.5		8.7%	1.1%		0	5.7
1598	C1076	Reef Margin, Upper Keys Trans	US Florida - Keys	28-Aug-06	25.621	-80.096	Kramer, Phil	7		2.6%	0.3%		0	5.7
1599	C1080	Reef Margin, Upper Keys Trans	US Florida - Keys	28-Aug-06	25.643	-80.097	Kramer, Phil	8.9		11.6%	0.2%		0	5.7
1600	C1082	Reef Margin, Upper Keys Trans	US Florida - Keys	28-Aug-06	25.574	-80.113	Kramer, Phil	4.1		2.0%	0.0%		0	4.5
1601	C2168	Reef Margin, Upper Keys Trans	US Florida - Keys	28-Aug-06	25.543	-80.102	Kramer, Phil	6		22.9%	0.7%		0	4.5
1602	C2174	Reef Margin, Upper Keys Trans	US Florida - Keys	28-Aug-06	25.592	-80.106	Kramer, Phil	4.5		12.5%	0.0%		0	5.7
1603	C1018	Reef Margin, Middle Keys	US Florida - Keys	1-Sep-06	24.623	-81.107	Kramer, Phil	15.7		15.0%	0.0%		0	3.4
1604	C1020	Offshore Patch Reef, Middle Ke	US Florida - Keys	1-Sep-06	24.672	-80.964	Kramer, Phil	9.8		0.0%	0.0%		0	3.4
1605	C2141	Offshore Patch Reef, Middle Ke	US Florida - Keys	1-Sep-06	24.705	-80.936	Kramer, Phil	7.1		15.9%	0.5%		0	3.4
1606	C2226	Offshore Patch Reef, Middle Ke	US Florida - Keys	1-Sep-06	24.698	-80.958	Kramer, Phil	4.6		15.4%	0.1%		0	3.4
1607	C1023	Inshore, Lower Keys	US Florida - Keys	5-Sep-06	24.517	-82.001	Kramer, Phil	6		12.5%	0.0%		0.5	3.4
1608	C1027	Mid Channel, Lower Keys	US Florida - Keys	5-Sep-06	24.477	-81.954	Kramer, Phil	6.7		16.1%	0.2%		0	1.7
1609	C1028	Mid Channel, Lower Keys	US Florida - Keys	5-Sep-06	24.480	-81.933	Kramer, Phil	5.1		7.8%	0.0%		0	1.7
1610	C1037	Offshore Patch Reef, Lower Key	US Florida - Keys	5-Sep-06	24.448	-81.950	Kramer, Phil	7.4		11.9%	0.0%		0	1.7
1611	C1043	Offshore Patch Reef, Lower Key	US Florida - Keys	5-Sep-06	24.456	-81.913	Kramer, Phil	9.6		13.6%	0.2%		0	1.7
1612	C2240	Inshore, Lower Keys	US Florida - Keys	5-Sep-06	24.533	-81.871	Kramer, Phil	2		0.0%	0.0%		0.5	3.4
1613	C1078	Reef Margin, Upper Keys Trans	US Florida - Keys	6-Sep-06	25.488	-80.109	Kramer, Phil	6		13.6%	0.9%		0	4.5
1614	C1081	Reef Margin, Upper Keys Trans	US Florida - Keys	6-Sep-06	25.469	-80.122	Kramer, Phil	7.5		4.3%	0.1%		0	4.5
1617	C2175	Reef Margin, Upper Keys Trans	US Florida - Keys	6-Sep-06	25.423	-80.128	Kramer, Phil	6		7.1%	0.1%		0	4.5
1618	C2179	Mid Channel, Upper Keys Trans	US Florida - Keys	6-Sep-06	25.461	-80.142	Kramer, Phil	3.7		1.5%	0.8%		0	4.5
1619	C1011	Reef Margin, Middle Keys	US Florida - Keys	7-Sep-06	24.627	-81.109	Kramer, Phil	3.6		21.4%	0.0%		0.5	3.4
1633	C1064.2	Inshore, Upper Keys Trans	US Florida - Keys	12-Sep-06	25.320	-80.207	Kramer, Phil	3.9		4.9%	0.0%		0	4.5
1634	C1069.2	Inshore, Upper Keys	US Florida - Keys	13-Sep-06	25.073	-80.379	Kramer, Phil	1.2		29.5%	0.0%		0	4.5
1635	C1070.2	Inshore, Upper Keys	US Florida - Keys	13-Sep-06	25.063	-80.395	Kramer, Phil	2.4		6.7%	0.0%		0	4.5
1636	C1071.2	Inshore, Upper Keys	US Florida - Keys	13-Sep-06	25.061	-80.387	Kramer, Phil	3		11.1%	0.0%		0	4.5
1637	C4109	Inshore, Upper Keys	US Florida - Keys	13-Sep-06	25.207	-80.288	Kramer, Phil	2.4		17.0%	0.0%		0	4.5
1638	C4110	Inshore, Upper Keys	US Florida - Keys	13-Sep-06	25.112	-80.342	Kramer, Phil	1.7		13.9%	0.0%		0	4.5
1645	C1075	Reef Margin, Upper Keys Trans	US Florida - Keys	18-Sep-06	25.500	-80.103	Kramer, Phil	13		13.5%	0.1%		0.5	4.5
1646	C1079	Reef Margin, Upper Keys Trans	US Florida - Keys	18-Sep-06	25.446	-80.120	Kramer, Phil	6		9.3%	0.8%		0.5	4.5
1647	C1083	Reef Margin, Upper Keys Trans	US Florida - Keys	18-Sep-06	25.498	-80.123	Kramer, Phil	2.6		1.5%	0.1%		0.5	4.5
1648	C1088	Mid Channel, Upper Keys Trans	US Florida - Keys	18-Sep-06	25.504	-80.132	Kramer, Phil	4		1.0%	0.2%		0.5	4.5
1649	C1093	Mid Channel, Upper Keys Trans	US Florida - Keys	18-Sep-06	25.394	-80.161	Kramer, Phil	3.7		4.5%	1.6%		0.5	4.5
1650	C1094	Mid Channel, Upper Keys Trans	US Florida - Keys	18-Sep-06	25.390	-80.149	Kramer, Phil	7.9		20.6%	6.2%		0.5	4.5
1651	C2181	Mid Channel, Upper Keys Trans	US Florida - Keys	18-Sep-06	25.405	-80.165	Kramer, Phil	2		7.2%	1.0%		0.5	4.5
1652	C1052	Reef Margin, Lower Keys	US Florida - Keys	19-Sep-06	24.467	-81.806	Kramer, Phil	17.6		10.7%	1.4%		0	1.7
1653	C1084	Reef Margin, Upper Keys Trans	US Florida - Keys	19-Sep-06	25.403	-80.131	Kramer, Phil	1.5		2.1%	0.0%		0.5	4.5
1654	C1054	Offshore Patch Reef, Upper Key	US Florida - Keys	20-Sep-06	25.104	-80.310	Kramer, Phil	6.7		13.6%	0.3%		0.5	4.5
1655	C1057	Offshore Patch Reef, Upper Key	US Florida - Keys	20-Sep-06	25.083	-80.323	Kramer, Phil	4.8		7.7%	0.0%		0.5	4.5
1656	C1062	Offshore Patch Reef, Upper Key	US Florida - Keys	20-Sep-06	25.118	-80.302	Kramer, Phil	5.5		7.0%	2.5%		0.5	4.5
1657	C1069.1	Inshore, Upper Keys	US Florida - Keys	20-Sep-06	25.073	-80.378	Kramer, Phil	3		2.4%	0.0%		0.5	4.5
1658	C1070.1	Inshore, Upper Keys	US Florida - Keys	20-Sep-06	25.062	-80.395	Kramer, Phil	3		7.9%	0.0%		0.5	4.5
1659	C1071.1	Inshore, Upper Keys	US Florida - Keys	20-Sep-06	25.060	-80.387	Kramer, Phil	3		5.5%	0.6%		0.5	4.5
1660	C1113	Reef Margin, Upper Keys	US Florida - Keys	20-Sep-06	25.111	-80.284	Kramer, Phil	12.8		12.9%	0.0%		0.5	4.5
1661	C1000	Reef Margin, Upper Keys	US Florida - Keys	21-Sep-06	25.214	-80.211	Kramer, Phil	7.1		23.7%	1.1%		0.5	4.5
1662	C1055	Offshore Patch Reef, Upper Key	US Florida - Keys	21-Sep-06	25.242	-80.224	Kramer, Phil	2.1		14.3%	0.0%		0.5	4.5
1663	C1056	Offshore Patch Reef, Upper Key	US Florida - Keys	21-Sep-06	25.225	-80.214	Kramer, Phil	4.1		14.8%	0.0%		0.5	4.5
1664	C1058	Reef Margin, Upper Keys	US Florida - Keys	21-Sep-06	25.230	-80.206	Kramer, Phil	4.3		29.7%	0.5%		0.5	4.5
1665	C1059	Reef Margin, Upper Keys	US Florida - Keys	21-Sep-06	25.145	-80.254	Kramer, Phil	9.8		32.1%	3.4%		0.5	4.5
1666	C1060	Offshore Patch Reef, Upper Key	US Florida - Keys	21-Sep-06	25.244	-80.213	Kramer, Phil	4.6		19.4%	0.3%		0.5	4.5

Observation Number	Reef or Site Name	Location	Country	Date (DD-MMM-YY)	Latitude (decimal degrees)	Longitude (decimal degrees)	Primary Contributor	Depth (m)	Cover bleached (%)	Colonies bleached (%)	Cover dead (%)	Colonies dead (%)	Observed DHW (°C-weeks)	Maximum DHW (°C-weeks)
1667	C1066	Mid Channel, Upper Keys	US Florida - Keys	21-Sep-06	25.291	-80.213	Kramer, Phil	10		11.4%	0.0%		0.5	4.5
1668	C1067	Mid Channel, Upper Keys	US Florida - Keys	21-Sep-06	25.288	-80.223	Kramer, Phil	3.5		7.8%	0.1%		0.5	4.5
1669	C1068	Mid Channel, Upper Keys	US Florida - Keys	21-Sep-06	25.277	-80.227	Kramer, Phil	1.6		12.3%	0.0%		0.5	4.5
1673	C1008	Inshore, Upper Keys	US Florida - Keys	22-Sep-06	24.969	-80.539	Kramer, Phil	3.1		50.0%	0.0%		0.5	4.5
1674	C1010	Mid Channel, Upper Keys	US Florida - Keys	22-Sep-06	24.955	-80.504	Kramer, Phil	3.5		12.7%	0.0%		1	3.4
1675	C1014	Offshore Patch Reef, Upper Key	US Florida - Keys	22-Sep-06	24.922	-80.522	Kramer, Phil	3		20.0%	0.0%		1	3.4
1676	C1017	Reef Margin, Upper Keys	US Florida - Keys	22-Sep-06	24.895	-80.556	Kramer, Phil	9		37.5%	0.0%		1	3.4
1677	C1063	Offshore Patch Reef, Upper Key	US Florida - Keys	22-Sep-06	25.003	-80.438	Kramer, Phil	3		13.9%	0.0%		0.5	4.5
1684	C1085	Reef Margin, Upper Keys Trans	US Florida - Keys	26-Sep-06	25.363	-80.141	Kramer, Phil	6.3		18.5%	0.0%		0.5	4.5
1685	C1095	Inshore, Upper Keys Trans	US Florida - Keys	26-Sep-06	25.367	-80.187	Kramer, Phil	2.9		1.4%	0.2%		0.5	4.5
1686	C1012	Reef Margin, Upper Keys	US Florida - Keys	27-Sep-06	24.847	-80.623	Kramer, Phil	7.6		24.1%	0.5%		1	3.4
1687	C1016	Reef Margin, Upper Keys	US Florida - Keys	27-Sep-06	24.843	-80.631	Kramer, Phil	6.2		25.0%	0.0%		1	3.4
1688	C1089	Mid Channel, Upper Keys Trans	US Florida - Keys	27-Sep-06	25.491	-80.131	Kramer, Phil	3.5		6.1%	0.2%		0.5	4.5
1689	C1097	Inshore, Upper Keys Trans	US Florida - Keys	27-Sep-06	25.428	-80.191	Kramer, Phil	4		0.0%	0.0%		0.5	4.5
1690	C1098	Inshore, Upper Keys Trans	US Florida - Keys	27-Sep-06	25.414	-80.193	Kramer, Phil	4		0.8%	0.1%		0.5	4.5
1691	C2142	Inshore, Upper Keys	US Florida - Keys	27-Sep-06	24.904	-80.617	Kramer, Phil	2.8		8.3%	0.2%		1	3.4
1694	C1064.1	Inshore, Upper Keys Trans	US Florida - Keys	30-Sep-06	25.320	-80.207	Kramer, Phil	2.6		3.6%	0.5%		0.5	4.5
1695	C1065	Mid Channel, Upper Keys Trans	US Florida - Keys	30-Sep-06	25.309	-80.213	Kramer, Phil	5.2		4.8%	2.8%		0.5	4.5
1696	C1096	Mid Channel, Upper Keys Trans	US Florida - Keys	30-Sep-06	25.347	-80.187	Kramer, Phil	5.3		0.0%	2.4%		0.5	4.5
1697	C2178	Offshore Patch Reef, Upper Key	US Florida - Keys	30-Sep-06	25.329	-80.173	Kramer, Phil	10		9.5%	0.4%		0.5	4.5
1698	C1125	Reef Ridge Complex, Palm Beac	US Florida - Keys	11-Oct-06	26.649	-80.020	Kramer, Phil	22		12.9%	2.8%		0	5.7
1699	C1130	Reef Ridge Complex, Palm Beac	US Florida - Keys	12-Oct-06	26.435	-80.049	Kramer, Phil	8.8		37.3%	0.0%		0	5.7
1700	C2220	Reef Ridge Complex, Palm Beac	US Florida - Keys	12-Oct-06	26.481	-80.042	Kramer, Phil	17		31.0%	0.0%		0	5.7
1437	A1112	Inshore, Palm Beach	US Florida - Mainland	20-Aug-05	26.707	-80.030	Kramer, Phil	2.9		28.6%	0.0%		3.55	6
1438	A2111	Inshore, Palm Beach	US Florida - Mainland	20-Aug-05	26.723	-80.029	Kramer, Phil	7.9		4.5%	0.1%		3.55	6
1439	A3003	Inshore, Palm Beach	US Florida - Mainland	20-Aug-05	26.709	-80.028	Kramer, Phil	7		0.0%	0.9%		3.55	6
1440	A4000	Undetermined, Northern Trans	US Florida - Mainland	21-Aug-05	27.108	-80.123	Kramer, Phil	4.2		75.0%	0.0%		4.6	5.2
1441	A4001	Undetermined, Northern Trans	US Florida - Mainland	21-Aug-05	27.112	-80.125	Kramer, Phil	4.9		25.0%	0.0%		4.6	5.2
1442	A4002	Undetermined, Northern Trans	US Florida - Mainland	21-Aug-05	27.132	-80.134	Kramer, Phil	3.7		23.1%	0.1%		4.6	5.2
1443	A1001	Inner Reef, Broward	US Florida - Mainland	22-Aug-05	25.800	-80.096	Kramer, Phil	14		33.3%	0.8%		3.55	4.7
1444	A1002	Inner Reef, Broward	US Florida - Mainland	22-Aug-05	25.798	-80.098	Kramer, Phil	10.4		23.5%	0.2%		3.55	4.7
1445	A1023	Outer Reef, Broward	US Florida - Mainland	22-Aug-05	25.778	-80.090	Kramer, Phil	12.2		46.2%	2.3%		3.55	4.7
1451	A2000	Inner Reef, Broward	US Florida - Mainland	22-Aug-05	25.825	-80.091	Kramer, Phil	16.4		22.6%	0.8%		3.55	4.7
1454	A1003	Outer Reef, Broward	US Florida - Mainland	23-Aug-05	26.305	-80.068	Kramer, Phil	11.8		22.0%	0.4%		4.25	6
1455	A1004	Inner Reef, Broward	US Florida - Mainland	23-Aug-05	26.286	-80.070	Kramer, Phil	13.7		25.0%	0.4%		4.25	6
1456	A1005	Inner Reef, Broward	US Florida - Mainland	23-Aug-05	26.203	-80.078	Kramer, Phil	19		33.3%	1.7%		3.55	4.7
1457	A1006	Outer Reef, Broward	US Florida - Mainland	23-Aug-05	26.185	-80.076	Kramer, Phil	14		36.0%	0.5%		3.55	4.7
1458	A1014	Inshore, Broward	US Florida - Mainland	23-Aug-05	25.942	-80.101	Kramer, Phil	11.3		42.3%	0.3%		3.55	4.7
1459	A1015	Inshore, Broward	US Florida - Mainland	23-Aug-05	26.193	-80.091	Kramer, Phil	16		27.3%	0.6%		3.55	4.7
1460	A1018	Inshore, Broward	US Florida - Mainland	23-Aug-05	25.887	-80.111	Kramer, Phil	8.5		29.4%	0.0%		3.55	4.7
1461	A1022	Outer Reef, Broward	US Florida - Mainland	23-Aug-05	25.862	-80.087	Kramer, Phil	23.2		29.2%	0.1%		3.55	4.7
1462	A1025	Outer Reef, Broward	US Florida - Mainland	23-Aug-05	25.902	-80.088	Kramer, Phil	19.8		9.7%	0.2%		3.55	4.7
1463	A1026	Outer Reef, Broward	US Florida - Mainland	23-Aug-05	26.309	-80.060	Kramer, Phil	19.1		20.0%	0.0%		4.25	6
1464	A1028	Outer Reef, Broward	US Florida - Mainland	23-Aug-05	25.841	-80.088	Kramer, Phil	14.3		33.3%	0.1%		3.55	4.7
1465	A1150	Outer Reef, Broward	US Florida - Mainland	23-Aug-05	25.678	-80.090	Kramer, Phil	12		20.0%	0.2%		4.1	5.7
1466	A3002	Outer Reef, Broward	US Florida - Mainland	23-Aug-05	25.842	-80.088	Kramer, Phil	14.7		7.1%	0.0%		3.55	4.7
1467	A4006	Inner Reef, Broward	US Florida - Mainland	23-Aug-05	25.700	-80.097	Kramer, Phil	8.5		37.8%	0.0%		4.1	5.7
1468	A4007	Inner Reef, Broward	US Florida - Mainland	23-Aug-05	25.675	-80.098	Kramer, Phil	10		50.0%	0.0%		4.1	5.7
1479	A3001	Outer Reef, Broward	US Florida - Mainland	31-Aug-05	25.842	-80.104	Kramer, Phil	17.3		50.0%	0.0%		4.7	4.7
1480	A4003	Inner Reef, Broward	US Florida - Mainland	31-Aug-05	26.160	-80.083	Kramer, Phil	13.6		22.2%	4.1%		4.7	4.7
1481	A4004	Outer Reef, Broward	US Florida - Mainland	31-Aug-05	26.159	-80.078	Kramer, Phil	17.3		50.0%	0.0%		4.7	4.7
1482	A4009	Inshore, Broward	US Florida - Mainland	31-Aug-05	26.148	-80.096	Kramer, Phil	6.4		35.7%	0.0%		4.7	4.7
1001	POMP3	Broward County, FL- 3rdReef	US Florida - Mainland	1-Sep-05	26.187	-80.073	Gilliam, Dave	15	10.0%	12.9%	0.0%	0.0%	4.7	4.7
1483	A1011	Inshore, Broward	US Florida - Mainland	1-Sep-05	25.986	-80.105	Kramer, Phil	9.1		50.0%	2.9%		4.7	4.7
1484	A1013	Inshore, Broward	US Florida - Mainland	1-Sep-05	25.998	-80.102	Kramer, Phil	8.3		33.3%	1.0%		4.7	4.7
1487	A1008	Inshore, Broward	US Florida - Mainland	2-Sep-05	25.966	-80.114	Kramer, Phil	5.6		52.2%	0.0%		4.7	4.7
1488	A1010	Inshore, Broward	US Florida - Mainland	2-Sep-05	25.975	-80.105	Kramer, Phil	9.9		60.0%	0.0%		4.7	4.7
1489	A1024	Outer Reef, Broward	US Florida - Mainland	2-Sep-05	25.970	-80.090	Kramer, Phil	17.8		53.3%	0.0%		4.7	4.7

Observation Number	Reef or Site Name	Location	Country	Date (DD-MMM-YY)	Latitude (decimal degrees)	Longitude (decimal degrees)	Primary Contributor	Depth (m)	Cover bleached (%)	Colonies bleached (%)	Cover dead (%)	Colonies dead (%)	Observed DHW (°C-weeks)	Maximum DHW (°C-weeks)
1000	Marker 3 Reef	Miami	US Florida - Mainland	9-Sep-05	25.373	-80.160	DiResta, Dan	3		100.0%	0.0%	0.0%	5.7	5.7
2109	Biscayne National Park Inshore	Biscayne National Park Inshore	US Florida - Mainland	10-Sep-05	25.497	-80.195	ReefBase	5	89.0%				5.7	5.7
1497	A1012	Inshore, Broward	US Florida - Mainland	11-Sep-05	26.014	-80.102	Kramer, Phil	7.8		42.1%	1.1%		4.7	4.7
1002	BOCA1	Broward County, FL- 2nd Reef	US Florida - Mainland	15-Sep-05	26.347	-80.065	Gilliam, Dave	8	17.5%	17.6%	0.0%	0.0%	6	6
1003	DB2	Broward County, FL- 2nd Reef	US Florida - Mainland	15-Sep-05	26.310	-80.067	Gilliam, Dave	12	11.3%	11.4%	0.0%	0.0%	6	6
2115	Boynton Beach	Boynton Beach	US Florida - Mainland	1-Oct-05	26.547	-80.037	ReefBase	5	3.0%				6	6
1004	HB1	Broward County, FL- 1st Reef	US Florida - Mainland	12-Oct-05	26.281	-80.076	Gilliam, Dave	6	1.0%	0.7%	0.0%	0.0%	6	6
1005	FTL1	Broward County, FL- 1st Reef	US Florida - Mainland	14-Oct-05	26.159	-80.096	Gilliam, Dave	6	0.0%	0.0%	0.0%	0.0%	4.7	4.7
1006	POMP6	Broward County, FL- 3rdReef	US Florida - Mainland	9-Dec-05	26.243	-80.073	Gilliam, Dave	15	15.0%	16.0%	0.0%	0.0%	0	4.7
1007	POMP4	Broward County, FL- 1st Reef	US Florida - Mainland	20-Dec-05	26.212	-80.087	Gilliam, Dave	6	3.4%	3.4%	0.0%	0.0%	0	4.7
1008	JUL7	Broward County, FL- 2nd Reef	US Florida - Mainland	8-Feb-06	26.083	-80.096	Gilliam, Dave	10	12.8%	12.8%	0.0%	0.0%	0	4.7
1009	JUL8	Broward County, FL- 3rdReef	US Florida - Mainland	8-Feb-06	26.083	-80.085	Gilliam, Dave	15	4.0%	8.2%	0.0%	0.0%	0	4.7
1537	B1000	Inner Reef, Broward	US Florida - Mainland	21-Feb-06	25.751	-80.098	Kramer, Phil	7.6		3.9%	0.2%		0	4.7
1538	B1147	Inner Reef, Broward	US Florida - Mainland	21-Feb-06	25.696	-80.097	Kramer, Phil	8.5		7.7%	0.2%		0	5.7
1539	B1150	Outer Reef, Broward	US Florida - Mainland	21-Feb-06	25.678	-80.091	Kramer, Phil	12.5		0.0%	0.2%		0	5.7
1544	B1157	Outer Reef, Broward	US Florida - Mainland	22-Feb-06	25.696	-80.090	Kramer, Phil	6.1		7.1%	0.4%		0	5.7
1566	B1007	Inner Reef, Broward	US Florida - Mainland	17-Mar-06	26.122	-80.087	Kramer, Phil	14.3		13.0%	0.0%		0	4.7
1567	B1009	Inshore, Broward	US Florida - Mainland	17-Mar-06	26.018	-80.112	Kramer, Phil	5.6		4.0%	0.2%		0	4.7
1568	B1010	Inshore, Broward	US Florida - Mainland	17-Mar-06	25.975	-80.105	Kramer, Phil	9.9		6.3%	0.0%		0	4.7
1569	B2006	Inner Reef, Broward	US Florida - Mainland	17-Mar-06	26.007	-80.097	Kramer, Phil	11.9		15.6%	0.9%		0	4.7
1570	B2010	Inshore, Broward	US Florida - Mainland	17-Mar-06	26.065	-80.106	Kramer, Phil	6.6		20.0%	2.9%		0	4.7
1571	B2021	Outer Reef, Broward	US Florida - Mainland	17-Mar-06	26.032	-80.087	Kramer, Phil	14.2		12.1%	0.7%		0	4.7
1572	B2013	Inshore, Broward	US Florida - Mainland	23-Mar-06	26.164	-80.095	Kramer, Phil	5.6		0.0%	0.0%		0	4.7
1573	B1005	Inner Reef, Broward	US Florida - Mainland	24-Mar-06	26.203	-80.078	Kramer, Phil	17.3		10.8%	0.2%		0	4.7
1574	B1006	Inner Reef, Broward	US Florida - Mainland	24-Mar-06	26.185	-80.081	Kramer, Phil	13.6		8.3%	0.0%		0	4.7
1575	B1003	Inner Reef, Broward	US Florida - Mainland	4-Apr-06	26.306	-80.068	Kramer, Phil	10.8		22.7%	0.6%		0	6
1576	B1026	Outer Reef, Broward	US Florida - Mainland	4-Apr-06	26.259	-80.059	Kramer, Phil	20.1		12.0%	2.4%		0	6
1577	B2023	Outer Reef, Broward	US Florida - Mainland	4-Apr-06	26.217	-80.070	Kramer, Phil	15.1		37.3%	0.0%		0	4.7
1591	C2173	Inner Reef, Broward	US Florida - Mainland	24-Aug-06	25.702	-80.102	Kramer, Phil	8.4		11.1%	0.4%		0	5.7
1615	C1116	Inshore, Broward	US Florida - Mainland	6-Sep-06	25.854	-80.110	Kramer, Phil	5.2		6.9%	0.9%		0	4.7
1616	C1117	Inshore, Broward	US Florida - Mainland	6-Sep-06	25.870	-80.113	Kramer, Phil	5.2		6.1%	0.8%		0	4.7
1620	C1099	Outer Reef, Broward	US Florida - Mainland	7-Sep-06	25.778	-80.086	Kramer, Phil	23.9		8.3%	2.8%		0	4.7
1621	C1101	Outer Reef, Broward	US Florida - Mainland	7-Sep-06	26.205	-80.073	Kramer, Phil	18.9		14.3%	4.5%		0	4.7
1622	C1103	Inner Reef, Broward	US Florida - Mainland	7-Sep-06	25.845	-80.094	Kramer, Phil	15		20.0%	1.2%		0	4.7
1623	C1105	Inner Reef, Broward	US Florida - Mainland	7-Sep-06	25.783	-80.096	Kramer, Phil	10.7		5.0%	0.1%		0	4.7
1624	C1114	Inshore, Broward	US Florida - Mainland	7-Sep-06	25.838	-80.099	Kramer, Phil	11.3		5.3%	0.4%		0	4.7
1625	C1115	Inshore, Broward	US Florida - Mainland	7-Sep-06	25.854	-80.102	Kramer, Phil	9.8		7.7%	0.9%		0	4.7
1626	C1118	Inshore, Broward	US Florida - Mainland	7-Sep-06	26.245	-80.080	Kramer, Phil	6.8		21.4%	0.3%		0	4.7
1627	C1119	Inshore, Broward	US Florida - Mainland	7-Sep-06	26.140	-80.090	Kramer, Phil	8.3		21.3%	1.8%		0	4.7
1628	C1121	Inshore, Broward	US Florida - Mainland	7-Sep-06	26.160	-80.097	Kramer, Phil	5		26.7%	0.0%		0	4.7
1629	C1123	Inshore, Broward	US Florida - Mainland	7-Sep-06	26.085	-80.106	Kramer, Phil	4.2		0.0%	0.0%		0	4.7
1630	C2202	Inner Reef, Broward	US Florida - Mainland	7-Sep-06	26.090	-80.089	Kramer, Phil	18.4		5.4%	1.5%		0	4.7
1631	C1104	Outer Reef, Broward	US Florida - Mainland	8-Sep-06	26.371	-80.055	Kramer, Phil	17.2		7.7%	0.0%		0	6
1632	C1108	Inner Reef, Broward	US Florida - Mainland	8-Sep-06	26.282	-80.070	Kramer, Phil	10		15.4%	1.6%		0	6
1639	C1111	Inner Reef, Broward	US Florida - Mainland	14-Sep-06	26.058	-80.095	Kramer, Phil	10.7		15.4%	0.2%		0	4.7
1640	C1122	Inshore, Broward	US Florida - Mainland	14-Sep-06	26.038	-80.105	Kramer, Phil	7.3		6.5%	0.3%		0	4.7
1641	C2203	Inner Reef, Broward	US Florida - Mainland	14-Sep-06	26.027	-80.096	Kramer, Phil	11.4		0.0%	0.0%		0	4.7
1642	C2208	Inshore, Broward	US Florida - Mainland	14-Sep-06	26.144	-80.094	Kramer, Phil	7.3		16.7%	0.0%		0	4.7
1643	C2209	Inshore, Broward	US Florida - Mainland	14-Sep-06	26.112	-80.095	Kramer, Phil	6.4		4.2%	0.0%		0	4.7
1644	C2211	Inshore, Broward	US Florida - Mainland	14-Sep-06	26.069	-80.104	Kramer, Phil	6.4		9.1%	0.6%		0	4.7
1670	C4101	Undetermined, Northern Trans	US Florida - Mainland	21-Sep-06	27.132	-80.134	Kramer, Phil	3.5		33.3%	1.2%		0	5.2
1671	C4102	Undetermined, Northern Trans	US Florida - Mainland	21-Sep-06	27.112	-80.126	Kramer, Phil	5		29.4%	2.5%		0	5.2
1672	C4103	Undetermined, Northern Trans	US Florida - Mainland	21-Sep-06	27.108	-80.123	Kramer, Phil	2.4		18.4%	8.8%		0	5.2
1678	C4104	Undetermined, Northern Trans	US Florida - Mainland	22-Sep-06	27.106	-80.122	Kramer, Phil	4.9		18.2%	3.0%		0	5.2
1679	C4105	Undetermined, Northern Trans	US Florida - Mainland	22-Sep-06	27.104	-80.121	Kramer, Phil	7.2		13.3%	4.3%		0	5.2
1680	C4106	Undetermined, Northern Trans	US Florida - Mainland	22-Sep-06	27.121	-80.127	Kramer, Phil	6		12.5%	4.8%		0	5.2
1681	C1102	Outer Reef, Broward	US Florida - Mainland	25-Sep-06	25.967	-80.090	Kramer, Phil	18.9		11.8%	0.0%		0	4.7
1682	C2210	Inshore, Broward	US Florida - Mainland	25-Sep-06	25.985	-80.099	Kramer, Phil	10.7		0.0%	0.0%		0	4.7

Observation Number	Reef or Site Name	Location	Country	Date (DD-MMM-YY)	Latitude (decimal degrees)	Longitude (decimal degrees)	Primary Contributor	Depth (m)	Cover bleached (%)	Colonies bleached (%)	Cover dead (%)	Colonies dead (%)	Observed DHW (°C-weeks)	Maximum DHW (°C-weeks)
1683	C2213	Inshore, Broward	US Florida - Mainland	25-Sep-06	25.948	-80.108	Kramer, Phil	5.1		9.7%	0.2%		0	4.7
1692	C2190	Outer Reef, Broward	US Florida - Mainland	27-Sep-06	25.927	-80.087	Kramer, Phil	16.9		12.0%	0.0%		0	4.7
1693	C2212	Inshore, Broward	US Florida - Mainland	27-Sep-06	26.006	-80.106	Kramer, Phil	8.5		13.3%	0.0%		0	4.7
2110	Cayo Largo (off Fajardo)	Cayo Largo (off Fajardo)	US Puerto Rico - East	10-Sep-05	18.309	-65.655	ReefBase	5	62.5%				1.75	6.5
2111	Isla Piñero (off Ceiba)	Isla Piñero (off Ceiba)	US Puerto Rico - East	10-Sep-05	18.256	-65.592	ReefBase	5	62.5%				1.75	6.5
1076	Palominitos Island	Fajardo	US Puerto Rico - East	15-Sep-05	18.354	-65.566	Hernandez, Edwin	9.5	81.0%	81.6%	0.3%		2.4	6.5
1077	Cayo Diablo	Fajardo	US Puerto Rico - East	16-Sep-05	18.360	-65.529	Hernandez, Edwin	4.25	82.0%	71.2%	0.0%		3.85	10.25
1078	Cayo Largo	Fajardo	US Puerto Rico - East	16-Sep-05	18.326	-65.581	Hernandez, Edwin	3	93.0%	93.5%	20.0%		3.15	6.5
1079	Palominitos Island	Fajardo	US Puerto Rico - East	16-Sep-05	18.336	-65.566	Hernandez, Edwin	16.5	68.0%	68.8%	0.0%		3.15	6.5
1080	Sur de Pando	Fajardo	US Puerto Rico - East	16-Sep-05	18.366	-65.548	Hernandez, Edwin	4.75	34.0%	44.7%	0.0%		3.85	10.25
1081	Punta Soldado	Culebra Island	US Puerto Rico - East	22-Sep-05	18.279	-65.288	Hernandez, Edwin	4.5	86.0%	84.6%	0.0%		5.3	10.25
1082	Carlos Rosario Beach	Culebra Island	US Puerto Rico - East	23-Sep-05	18.325	-65.332	Hernandez, Edwin	7.25	95.0%	94.2%	1.0%		5.3	10.25
1083	Peninsula Flamenco	Culebra Island	US Puerto Rico - East	24-Sep-05	18.331	-65.333	Hernandez, Edwin	8	93.0%	89.8%	1.0%		5.3	10.25
1084	Playa Larga	Culebra Island	US Puerto Rico - East	30-Sep-05	18.317	-65.251	Hernandez, Edwin	3.5	67.0%	72.5%	0.0%		6.7	10.25
1085	Culebrita Island	Culebra Island	US Puerto Rico - East	2-Nov-05	18.386	-65.627	Hernandez, Edwin	11	92.0%	91.5%	2.5%		6.5	6.5
2818	Culebrita	East Coast	US Puerto Rico - East	2-Nov-05	18.317	-65.225	Weil, Ernesto	8		34.2%			10.25	10.25
1087	Ensenada Yegua	Fajardo	US Puerto Rico - East	15-Nov-05	18.386	-65.627	Hernandez, Edwin	3	58.0%	63.0%	2.0%		5.95	6.5
1088	Isla Verde	Carolina	US Puerto Rico - East	9-Dec-05	18.468	-66.098	Hernandez, Edwin	14.5	23.0%	23.0%	1.0%		3.75	6.85
2814	Guanica	South Coast	US Puerto Rico - West	14-Sep-05	17.956	-66.788	Weil, Ernesto	9.5		23.7%			2.25	9.65
1086	Shacks	Isabela	US Puerto Rico - West	11-Nov-05	18.517	-67.098	Hernandez, Edwin	6	28.0%	33.3%	0.0%		6.7	7.2
82	Bajo Gullardo	Boqueron	US Puerto Rico - West	5-Dec-05	18.003	-67.332	Bruckner, Andy	4.5	3.8%	22.7%			4.75	7.55
83	Bajo Gullardo	Boqueron	US Puerto Rico - West	5-Dec-05	18.003	-67.332	Bruckner, Andy	16	53.9%	75.0%			4.75	7.55
84	Cable Reef	Desecheo Island	US Puerto Rico - West	6-Dec-05	18.382	-67.488	Bruckner, Andy	15	40.0%	31.6%			3.85	7.2
85	Candyland	Desecheo Island	US Puerto Rico - West	6-Dec-05	18.378	-67.485	Bruckner, Andy	22	98.3%	45.7%			3.85	7.2
86	Puerto De Botes	Desecheo Island	US Puerto Rico - West	6-Dec-05	18.381	-67.487	Bruckner, Andy	12	87.0%	31.8%			3.85	7.2
87	Cayo Ron	Mayaguez	US Puerto Rico - West	7-Dec-05	18.096	-67.287	Bruckner, Andy	10	20.8%	34.1%			4.75	7.55
88	El Negro norte	Mayaguez	US Puerto Rico - West	7-Dec-05	18.181	-67.237	Bruckner, Andy	10	40.6%	46.1%			4.75	7.55
89	Tourmaline	Mayaguez	US Puerto Rico - West	7-Dec-05	18.163	-67.274	Bruckner, Andy	15	41.2%	44.4%			4.75	7.55
90	San Cristobal	La Parguera	US Puerto Rico - West	8-Dec-05	17.941	-67.077	Bruckner, Andy	12.5	44.8%	35.0%			6.8	9.65
91	Turrumote	La Parguera	US Puerto Rico - West	8-Dec-05	17.935	-67.019	Bruckner, Andy	16.5	52.0%	67.2%			6.8	9.65
92	Turrumote	La Parguera	US Puerto Rico - West	8-Dec-05	17.935	-67.019	Bruckner, Andy	9	49.2%	22.5%			6.8	9.65
93	Carmelita middle	Mona island	US Puerto Rico - West	9-Dec-05	18.099	-67.938	Bruckner, Andy	10	63.8%	15.3%			4.25	7.55
94	Carmelita outer	Mona island	US Puerto Rico - West	9-Dec-05	18.105	-67.942	Bruckner, Andy	17	28.7%	15.8%			4.25	7.55
95	Carmelita south	Mona island	US Puerto Rico - West	9-Dec-05	18.099	-67.942	Bruckner, Andy	13	74.3%	48.3%			4.25	7.55
96	Mujeres West	Mona island	US Puerto Rico - West	9-Dec-05	18.073	-67.942	Bruckner, Andy	17	98.5%	67.2%			4.25	7.55
97	Sardinera	Mona island	US Puerto Rico - West	9-Dec-05	18.089	-67.944	Bruckner, Andy	8	19.6%	17.5%			4.25	7.55
98	Carabinero	Mona island	US Puerto Rico - West	10-Dec-05	18.060	-67.920	Bruckner, Andy	16	13.4%	32.6%			4.25	7.55
99	Mujeres east	Mona island	US Puerto Rico - West	10-Dec-05	18.072	-67.938	Bruckner, Andy	14	59.2%	22.8%			4.25	7.55
100	Mujeres Wall	Mona island	US Puerto Rico - West	10-Dec-05	18.072	-67.940	Bruckner, Andy	17	76.3%	47.8%			4.25	7.55
101	Uvero west	Mona island	US Puerto Rico - West	10-Dec-05	18.047	-67.903	Bruckner, Andy	13	37.2%	31.1%			4.25	7.55
102	Coco Beach	Mona island	US Puerto Rico - West	11-Dec-05	18.045	-67.884	Bruckner, Andy	15	83.0%	38.5%			4.25	7.55
103	Pajaros	Mona island	US Puerto Rico - West	11-Dec-05	18.055	-67.860	Bruckner, Andy	18	52.8%	13.8%			4.25	7.55
104	Pajaros middle shelf	Mona island	US Puerto Rico - West	11-Dec-05	18.056	-67.869	Bruckner, Andy	10	42.2%	30.1%			4.25	7.55
105	Uvero east	Mona island	US Puerto Rico - West	11-Dec-05	18.054	-67.912	Bruckner, Andy	16.5	42.9%	35.7%			4.25	7.55
106	Atravesado	Parguera	US Puerto Rico - West	12-Dec-05	17.939	-67.086	Bruckner, Andy	2	68.0%	60.3%			6.1	9.65
107	El Palo forereef	Parguera	US Puerto Rico - West	12-Dec-05	17.930	-67.096	Bruckner, Andy	11	43.8%	46.5%			6.1	9.65
108	Laurel east	Parguera	US Puerto Rico - West	12-Dec-05	17.939	-67.060	Bruckner, Andy	16.5	48.1%	39.2%			6.1	9.65
109	Laurel east	Parguera	US Puerto Rico - West	12-Dec-05	17.939	-67.060	Bruckner, Andy	10.5	48.1%	39.2%			6.1	9.65
110	grounding	Guayanilla	US Puerto Rico - West	13-Dec-05	17.960	-66.769	Bruckner, Andy	12	72.0%	71.9%			6.1	9.65
111	Old Buoy	Parguera	US Puerto Rico - West	13-Dec-05	17.888	-66.998	Bruckner, Andy	21.5	57.7%	36.4%			6.1	9.65
2421	East Flower Garden Bank	Flower Garden Banks NMS	US Texas	18-Oct-05	27.908	-93.596	Schmahl, George	21.35		47.0%	0.0%	0.0%	5.55	5.55
2422	West Flower Garden Bank	Flower Garden Banks NMS	US Texas	19-Oct-05	27.875	-93.817	Schmahl, George	23		33.2%	0.0%	0.0%	7.05	7.05
2423	East Flower Garden Bank	Flower Garden Banks NMS	US Texas	14-Nov-05	27.908	-93.596	Schmahl, George	19.8		46.0%	0.0%	0.0%	3	5.55
2424	East Flower Garden Bank	Flower Garden Banks NMS	US Texas	3-Jan-06	27.908	-93.596	Schmahl, George	19.8		10.3%	0.0%	0.0%	0	5.55
2425	West Flower Garden Bank	Flower Garden Banks NMS	US Texas	4-Jan-06	27.875	-93.817	Schmahl, George	21.9		4.5%	0.0%	0.0%	0	7.05
2644	Sprat Hall	Frederiksted	US Virgin Islands - St. Croix	1-Sep-05	17.734	-64.895	Taylor, Marcia	10	81.1%				3.8	13.35
2645	Sprat Hall	Frederiksted	US Virgin Islands - St. Croix	1-Sep-05	17.734	-64.895	Taylor, Marcia	10	83.0%				3.8	13.35
2646	Sprat Hall	Frederiksted	US Virgin Islands - St. Croix	1-Sep-05	17.734	-64.895	Taylor, Marcia	10	70.0%				3.8	13.35

Observation Number	Reef or Site Name	Location	Country	Date (DD-MMM-YY)	Latitude (decimal degrees)	Longitude (decimal degrees)	Primary Contributor	Depth (m)	Cover bleached (%)	Colonies bleached (%)	Cover dead (%)	Colonies dead (%)	Observed DHW (°C-weeks)	Maximum DHW (°C-weeks)
2647	Sprat Hall	Frederiksted	US Virgin Islands - St. Croix	1-Sep-05	17.734	-64.895	Taylor, Marcia	10	73.0%				3.8	13.35
2648	Sprat Hall	Frederiksted	US Virgin Islands - St. Croix	1-Sep-05	17.734	-64.895	Taylor, Marcia	10	90.0%				3.8	13.35
2649	Sprat Hall	Frederiksted	US Virgin Islands - St. Croix	1-Sep-05	17.734	-64.895	Taylor, Marcia	10	99.0%				3.8	13.35
2116	North shore of St. Croix	North shore of St. Croix	US Virgin Islands - St. Croix	1-Oct-05	17.764	-64.660	ReefBase	5	90.0%				9	13.35
2119	Buck Island, St. Croix	Buck Island, St. Croix	US Virgin Islands - St. Croix	7-Oct-05	17.790	-64.630	ReefBase	5	70.0%				10.45	13.35
2120	Cane Bay, St. Croix	Cane Bay, St. Croix	US Virgin Islands - St. Croix	7-Oct-05	17.703	-64.760	ReefBase	5	75.0%				8.15	10.25
758	Salt River	Salt River	US Virgin Islands - St. Croix	10-Oct-05	17.785	-64.759	Carr, Liam	13		100.0%			8.85	10.25
759	Salt River	Salt River	US Virgin Islands - St. Croix	10-Oct-05	17.785	-64.759	Carr, Liam	13		33.3%			8.85	10.25
760	Salt River	Salt River	US Virgin Islands - St. Croix	10-Oct-05	17.785	-64.759	Carr, Liam	13		100.0%			8.85	10.25
761	Salt River	Salt River	US Virgin Islands - St. Croix	10-Oct-05	17.785	-64.759	Carr, Liam	13		66.7%			8.85	10.25
762	Salt River	Salt River	US Virgin Islands - St. Croix	10-Oct-05	17.785	-64.759	Carr, Liam	13		75.0%			8.85	10.25
763	Salt River	Salt River	US Virgin Islands - St. Croix	10-Oct-05	17.785	-64.759	Carr, Liam	13		100.0%			8.85	10.25
2650	Salt River	No. shore	US Virgin Islands - St. Croix	13-Oct-05	17.785	-64.759	Taylor, Marcia	6	80.0%				9.55	10.25
2651	Salt River	No. shore	US Virgin Islands - St. Croix	13-Oct-05	17.785	-64.759	Taylor, Marcia	6	94.0%				9.55	10.25
2652	Salt River	No. shore	US Virgin Islands - St. Croix	13-Oct-05	17.785	-64.759	Taylor, Marcia	6	83.0%				9.55	10.25
2653	Salt River	No. shore	US Virgin Islands - St. Croix	13-Oct-05	17.785	-64.759	Taylor, Marcia	6	68.0%				9.55	10.25
2654	Salt River	No. shore	US Virgin Islands - St. Croix	13-Oct-05	17.785	-64.759	Taylor, Marcia	6	76.0%				9.55	10.25
2655	Salt River	No. shore	US Virgin Islands - St. Croix	13-Oct-05	17.785	-64.759	Taylor, Marcia	6	93.0%				9.55	10.25
764	Cane Bay	Cane Bay	US Virgin Islands - St. Croix	17-Oct-05	17.774	-64.814	Carr, Liam	12		66.7%			10.25	10.25
765	Cane Bay	Cane Bay	US Virgin Islands - St. Croix	17-Oct-05	17.774	-64.814	Carr, Liam	12		93.8%			10.25	10.25
766	Cane Bay	Cane Bay	US Virgin Islands - St. Croix	17-Oct-05	17.774	-64.814	Carr, Liam	12		88.2%			10.25	10.25
767	Cane Bay	Cane Bay	US Virgin Islands - St. Croix	17-Oct-05	17.774	-64.814	Carr, Liam	12		73.3%			10.25	10.25
768	Cane Bay	Cane Bay	US Virgin Islands - St. Croix	17-Oct-05	17.774	-64.814	Carr, Liam	12		100.0%			10.25	10.25
769	Cane Bay	Cane Bay	US Virgin Islands - St. Croix	17-Oct-05	17.774	-64.814	Carr, Liam	12		83.3%			10.25	10.25
1207	H107	St. Croix	US Virgin Islands - St. Croix	17-Oct-05	17.772	-64.565	Jeffrey, Chris	24.1	8.8%				12.3	13.35
1208	H74	St. Croix	US Virgin Islands - St. Croix	17-Oct-05	17.768	-64.657	Jeffrey, Chris	7.6	25.5%				12.3	13.35
1209	H87	St. Croix	US Virgin Islands - St. Croix	17-Oct-05	17.773	-64.566	Jeffrey, Chris	23.8	0.0%				12.3	13.35
2656	Cane Bay	No. shore	US Virgin Islands - St. Croix	17-Oct-05	17.774	-64.814	Taylor, Marcia	9	68.0%				10.25	10.25
2657	Cane Bay	No. shore	US Virgin Islands - St. Croix	17-Oct-05	17.774	-64.814	Taylor, Marcia	9	79.0%				10.25	10.25
2658	Cane Bay	No. shore	US Virgin Islands - St. Croix	17-Oct-05	17.774	-64.814	Taylor, Marcia	9	93.0%				10.25	10.25
2659	Cane Bay	No. shore	US Virgin Islands - St. Croix	17-Oct-05	17.774	-64.814	Taylor, Marcia	9	96.0%				10.25	10.25
2660	Cane Bay	No. shore	US Virgin Islands - St. Croix	17-Oct-05	17.774	-64.814	Taylor, Marcia	9	94.0%				10.25	10.25
2661	Cane Bay	No. shore	US Virgin Islands - St. Croix	17-Oct-05	17.774	-64.814	Taylor, Marcia	9	94.0%				10.25	10.25
1210	H3	St. Croix	US Virgin Islands - St. Croix	18-Oct-05	17.779	-64.643	Jeffrey, Chris	11.6	0.0%				12.3	13.35
1211	H47	St. Croix	US Virgin Islands - St. Croix	18-Oct-05	17.781	-64.645	Jeffrey, Chris	9.4	27.3%				12.3	13.35
1212	H73	St. Croix	US Virgin Islands - St. Croix	18-Oct-05	17.779	-64.663	Jeffrey, Chris	8.2	19.1%				12.3	13.35
1213	H75	St. Croix	US Virgin Islands - St. Croix	18-Oct-05	17.784	-64.569	Jeffrey, Chris	23.2	27.8%				12.3	13.35
1214	H76	St. Croix	US Virgin Islands - St. Croix	18-Oct-05	17.779	-64.666	Jeffrey, Chris	9.4	53.0%				12.3	13.35
1215	H77	St. Croix	US Virgin Islands - St. Croix	18-Oct-05	17.780	-64.572	Jeffrey, Chris	19.2	80.1%				12.3	13.35
1216	H89	St. Croix	US Virgin Islands - St. Croix	18-Oct-05	17.779	-64.655	Jeffrey, Chris	9.1	0.0%				12.3	13.35
1217	H91	St. Croix	US Virgin Islands - St. Croix	18-Oct-05	17.790	-64.573	Jeffrey, Chris	18.3	19.5%				12.3	13.35
1218	H97	St. Croix	US Virgin Islands - St. Croix	18-Oct-05	17.789	-64.569	Jeffrey, Chris	20.7	8.8%				12.3	13.35
1219	H11	St. Croix	US Virgin Islands - St. Croix	19-Oct-05	17.811	-64.630	Jeffrey, Chris	27.7	51.3%				12.3	13.35
1220	H2	St. Croix	US Virgin Islands - St. Croix	19-Oct-05	17.791	-64.585	Jeffrey, Chris	10.7	37.0%				12.3	13.35
1221	H22	St. Croix	US Virgin Islands - St. Croix	19-Oct-05	17.797	-64.572	Jeffrey, Chris	21	10.0%				12.3	13.35
1222	H36	St. Croix	US Virgin Islands - St. Croix	19-Oct-05	17.791	-64.645	Jeffrey, Chris	14.3	2.9%				12.3	13.35
1223	H39	St. Croix	US Virgin Islands - St. Croix	19-Oct-05	17.797	-64.576	Jeffrey, Chris	17.4	26.5%				12.3	13.35
1224	H4	St. Croix	US Virgin Islands - St. Croix	19-Oct-05	17.792	-64.593	Jeffrey, Chris	8.5	66.1%				12.3	13.35
1225	H51	St. Croix	US Virgin Islands - St. Croix	19-Oct-05	17.787	-64.642	Jeffrey, Chris	10.1	33.3%				12.3	13.35
1226	H95	St. Croix	US Virgin Islands - St. Croix	19-Oct-05	17.778	-64.647	Jeffrey, Chris	11	23.1%				12.3	13.35
770	Mutton Snapper	Southwest Bank	US Virgin Islands - St. Croix	20-Oct-05	17.637	-64.862	Carr, Liam	23		100.0%			12.3	13.35
771	Mutton Snapper	Southwest Bank	US Virgin Islands - St. Croix	20-Oct-05	17.637	-64.862	Carr, Liam	23		100.0%			12.3	13.35
772	Mutton Snapper	Southwest Bank	US Virgin Islands - St. Croix	20-Oct-05	17.637	-64.862	Carr, Liam	23		81.8%			12.3	13.35
773	Mutton Snapper	Southwest Bank	US Virgin Islands - St. Croix	20-Oct-05	17.637	-64.862	Carr, Liam	23		100.0%			12.3	13.35
774	Mutton Snapper	Southwest Bank	US Virgin Islands - St. Croix	20-Oct-05	17.637	-64.862	Carr, Liam	23		88.9%			12.3	13.35
775	Mutton Snapper	Southwest Bank	US Virgin Islands - St. Croix	20-Oct-05	17.637	-64.862	Carr, Liam	23		100.0%			12.3	13.35
1227	H10	St. Croix	US Virgin Islands - St. Croix	20-Oct-05	17.786	-64.595	Jeffrey, Chris	7.6	14.1%				12.3	13.35
1228	H102	St. Croix	US Virgin Islands - St. Croix	20-Oct-05	17.766	-64.618	Jeffrey, Chris	10.7	66.1%				12.3	13.35

Observation Number	Reef or Site Name	Location	Country	Date (DD-MMM-YY)	Latitude (decimal degrees)	Longitude (decimal degrees)	Primary Contributor	Depth (m)	Cover bleached (%)	Colonies bleached (%)	Cover dead (%)	Colonies dead (%)	Observed DHW (°C-weeks)	Maximum DHW (°C-weeks)
1229	H116	St. Croix	US Virgin Islands - St. Croix	20-Oct-05	17.767	-64.621	Jeffrey, Chris	9.8	51.7%				12.3	13.35
1230	H117	St. Croix	US Virgin Islands - St. Croix	20-Oct-05	17.782	-64.572	Jeffrey, Chris	19.5	17.8%				12.3	13.35
1231	H14	St. Croix	US Virgin Islands - St. Croix	20-Oct-05	17.785	-64.588	Jeffrey, Chris	8.2	57.1%				12.3	13.35
1232	H48	St. Croix	US Virgin Islands - St. Croix	20-Oct-05	17.786	-64.586	Jeffrey, Chris	11.6	9.1%				12.3	13.35
1233	H48	St. Croix	US Virgin Islands - St. Croix	20-Oct-05	17.786	-64.586	Jeffrey, Chris	10.7	55.6%				12.3	13.35
1234	H69	St. Croix	US Virgin Islands - St. Croix	20-Oct-05	17.766	-64.573	Jeffrey, Chris	9.1	40.4%				12.3	13.35
1235	H71	St. Croix	US Virgin Islands - St. Croix	20-Oct-05	17.782	-64.570	Jeffrey, Chris	21.3	45.0%				12.3	13.35
1236	H83	St. Croix	US Virgin Islands - St. Croix	20-Oct-05	17.767	-64.569	Jeffrey, Chris	21.3	8.3%				12.3	13.35
2662	Mutton Snapper aggregation site		US Virgin Islands - St. Croix	20-Oct-05	17.637	-64.862	Taylor, Marcia	23	71.0%				12.3	13.35
2663	Mutton Snapper aggregation site		US Virgin Islands - St. Croix	20-Oct-05	17.637	-64.862	Taylor, Marcia	23	97.0%				12.3	13.35
2664	Mutton Snapper aggregation site		US Virgin Islands - St. Croix	20-Oct-05	17.637	-64.862	Taylor, Marcia	23	91.0%				12.3	13.35
2665	Mutton Snapper aggregation site		US Virgin Islands - St. Croix	20-Oct-05	17.637	-64.862	Taylor, Marcia	23	84.0%				12.3	13.35
2666	Mutton Snapper aggregation site		US Virgin Islands - St. Croix	20-Oct-05	17.637	-64.862	Taylor, Marcia	23	97.0%				12.3	13.35
2667	Mutton Snapper aggregation site		US Virgin Islands - St. Croix	20-Oct-05	17.637	-64.862	Taylor, Marcia	23	87.0%				12.3	13.35
1237	H002	St. Croix	US Virgin Islands - St. Croix	21-Oct-05	17.763	-64.578	Jeffrey, Chris	2.7	98.8%				12.85	13.35
1238	H103	St. Croix	US Virgin Islands - St. Croix	21-Oct-05	17.777	-64.670	Jeffrey, Chris	6.4	47.5%				12.85	13.35
1239	H111	St. Croix	US Virgin Islands - St. Croix	21-Oct-05	17.767	-64.619	Jeffrey, Chris	13.4	64.0%				12.85	13.35
1240	H115	St. Croix	US Virgin Islands - St. Croix	21-Oct-05	17.768	-64.620	Jeffrey, Chris	13.4	0.0%				12.85	13.35
1241	H16	St. Croix	US Virgin Islands - St. Croix	22-Oct-05	17.802	-64.639	Jeffrey, Chris	19.2	77.5%				12.85	13.35
1242	H25	St. Croix	US Virgin Islands - St. Croix	22-Oct-05	17.802	-64.639	Jeffrey, Chris	19.5	84.1%				12.85	13.35
1243	H110	St. Croix	US Virgin Islands - St. Croix	24-Oct-05	17.764	-64.576	Jeffrey, Chris	3.7	29.6%				13.35	13.35
1244	H113	St. Croix	US Virgin Islands - St. Croix	24-Oct-05	17.767	-64.579	Jeffrey, Chris	7.9	18.5%				13.35	13.35
1245	H114	St. Croix	US Virgin Islands - St. Croix	24-Oct-05	17.769	-64.582	Jeffrey, Chris	19.2	100.0%				13.35	13.35
1246	H118	St. Croix	US Virgin Islands - St. Croix	24-Oct-05	17.764	-64.572	Jeffrey, Chris	7.6	35.2%				13.35	13.35
1247	H26	St. Croix	US Virgin Islands - St. Croix	24-Oct-05	17.794	-64.632	Jeffrey, Chris	3	0.0%				13.35	13.35
1248	H29	St. Croix	US Virgin Islands - St. Croix	24-Oct-05	17.795	-64.631	Jeffrey, Chris	4.6	41.4%				13.35	13.35
1249	H35	St. Croix	US Virgin Islands - St. Croix	24-Oct-05	17.795	-64.628	Jeffrey, Chris	6.7	89.2%				13.35	13.35
1250	H44	St. Croix	US Virgin Islands - St. Croix	24-Oct-05	17.793	-64.628	Jeffrey, Chris	6.4	96.2%				13.35	13.35
1251	H52	St. Croix	US Virgin Islands - St. Croix	24-Oct-05	17.793	-64.628	Jeffrey, Chris	5.8	28.2%				13.35	13.35
1252	H88	St. Croix	US Virgin Islands - St. Croix	24-Oct-05	17.764	-64.570	Jeffrey, Chris	8.5	50.0%				13.35	13.35
1253	H92	St. Croix	US Virgin Islands - St. Croix	24-Oct-05	17.772	-64.574	Jeffrey, Chris	17.4	45.9%				13.35	13.35
1254	H94	St. Croix	US Virgin Islands - St. Croix	24-Oct-05	17.765	-64.574	Jeffrey, Chris	6.4	50.0%				13.35	13.35
1255	H98	St. Croix	US Virgin Islands - St. Croix	24-Oct-05	17.774	-64.578	Jeffrey, Chris	16.8	26.3%				13.35	13.35
1256	H99	St. Croix	US Virgin Islands - St. Croix	24-Oct-05	17.775	-64.587	Jeffrey, Chris	17.4	35.3%				13.35	13.35
1257	H100	St. Croix	US Virgin Islands - St. Croix	25-Oct-05	17.781	-64.577	Jeffrey, Chris	11.9	74.2%				13.35	13.35
1258	H106	St. Croix	US Virgin Islands - St. Croix	25-Oct-05	17.766	-64.581	Jeffrey, Chris	5.2	59.3%				13.35	13.35
1259	H112	St. Croix	US Virgin Islands - St. Croix	25-Oct-05	17.767	-64.583	Jeffrey, Chris	11	58.8%				13.35	13.35
1260	H12	St. Croix	US Virgin Islands - St. Croix	25-Oct-05	17.792	-64.610	Jeffrey, Chris	13.4	95.6%				13.35	13.35
1261	H13	St. Croix	US Virgin Islands - St. Croix	25-Oct-05	17.793	-64.610	Jeffrey, Chris	11.9	86.8%				13.35	13.35
1262	H19	St. Croix	US Virgin Islands - St. Croix	25-Oct-05	17.794	-64.576	Jeffrey, Chris	19.5	7.4%				13.35	13.35
1263	H40	St. Croix	US Virgin Islands - St. Croix	25-Oct-05	17.787	-64.605	Jeffrey, Chris	11.3	82.5%				13.35	13.35
1264	H41	St. Croix	US Virgin Islands - St. Croix	25-Oct-05	17.789	-64.611	Jeffrey, Chris	3.4	72.0%				13.35	13.35
1265	H45	St. Croix	US Virgin Islands - St. Croix	25-Oct-05	17.794	-64.601	Jeffrey, Chris	8.5	39.8%				13.35	13.35
1266	H6	St. Croix	US Virgin Islands - St. Croix	25-Oct-05	17.797	-64.607	Jeffrey, Chris	5.8	23.1%				13.35	13.35
1267	H81	St. Croix	US Virgin Islands - St. Croix	25-Oct-05	17.782	-64.579	Jeffrey, Chris	11.3	52.4%				13.35	13.35
1268	H84	St. Croix	US Virgin Islands - St. Croix	25-Oct-05	17.795	-64.568	Jeffrey, Chris	20.4	33.3%				13.35	13.35
1269	H96	St. Croix	US Virgin Islands - St. Croix	25-Oct-05	17.784	-64.581	Jeffrey, Chris	12.5	15.0%				13.35	13.35
1270	H1	St. Croix	US Virgin Islands - St. Croix	26-Oct-05	17.806	-64.611	Jeffrey, Chris	11.9	36.9%				13.35	13.35
1271	H104	St. Croix	US Virgin Islands - St. Croix	26-Oct-05	17.762	-64.566	Jeffrey, Chris	9.4	56.4%				13.35	13.35
1272	H105	St. Croix	US Virgin Islands - St. Croix	26-Oct-05	17.759	-64.565	Jeffrey, Chris	7.9	58.7%				13.35	13.35
1273	H119	St. Croix	US Virgin Islands - St. Croix	26-Oct-05	17.761	-64.565	Jeffrey, Chris	11	69.1%				13.35	13.35
1274	H15	St. Croix	US Virgin Islands - St. Croix	26-Oct-05	17.799	-64.618	Jeffrey, Chris	11.3	91.5%				13.35	13.35
1275	H17	St. Croix	US Virgin Islands - St. Croix	26-Oct-05	17.780	-64.593	Jeffrey, Chris	11	70.0%				13.35	13.35
1276	H20	St. Croix	US Virgin Islands - St. Croix	26-Oct-05	17.802	-64.624	Jeffrey, Chris	4	19.4%				13.35	13.35
1277	H21	St. Croix	US Virgin Islands - St. Croix	26-Oct-05	17.786	-64.645	Jeffrey, Chris	10.1	0.0%				13.35	13.35
1278	H30	St. Croix	US Virgin Islands - St. Croix	26-Oct-05	17.802	-64.612	Jeffrey, Chris	7.3	54.6%				13.35	13.35
1279	H32	St. Croix	US Virgin Islands - St. Croix	26-Oct-05	17.804	-64.613	Jeffrey, Chris	9.1	84.3%				13.35	13.35
1280	H34	St. Croix	US Virgin Islands - St. Croix	26-Oct-05	17.805	-64.619	Jeffrey, Chris	7.9	47.9%				13.35	13.35

Observation Number	Reef or Site Name	Location	Country	Date (DD-MMM-YY)	Latitude (decimal degrees)	Longitude (decimal degrees)	Primary Contributor	Depth (m)	Cover bleached (%)	Colonies bleached (%)	Cover dead (%)	Colonies dead (%)	Observed DHW (°C-weeks)	Maximum DHW (°C-weeks)
1281	H38	St. Croix	US Virgin Islands - St. Croix	26-Oct-05	17.780	-64.594	Jeffrey, Chris	8.8	52.1%				13.35	13.35
1282	H43	St. Croix	US Virgin Islands - St. Croix	26-Oct-05	17.798	-64.625	Jeffrey, Chris	10.7	25.6%				13.35	13.35
1283	H46	St. Croix	US Virgin Islands - St. Croix	26-Oct-05	17.780	-64.592	Jeffrey, Chris	12.2	63.4%				13.35	13.35
1284	H53	St. Croix	US Virgin Islands - St. Croix	26-Oct-05	17.800	-64.630	Jeffrey, Chris	3.7	62.6%				13.35	13.35
1285	H70	St. Croix	US Virgin Islands - St. Croix	26-Oct-05	17.758	-64.568	Jeffrey, Chris	6.1	13.9%				13.35	13.35
1286	H200	St. Croix	US Virgin Islands - St. Croix	27-Oct-05	17.804	-64.631	Jeffrey, Chris	6.7	14.6%				12.85	13.35
1287	H23	St. Croix	US Virgin Islands - St. Croix	27-Oct-05	17.799	-64.586	Jeffrey, Chris	15.5	73.0%				12.85	13.35
1288	H24	St. Croix	US Virgin Islands - St. Croix	27-Oct-05	17.783	-64.649	Jeffrey, Chris	11.3	62.5%				12.85	13.35
1289	H27	St. Croix	US Virgin Islands - St. Croix	27-Oct-05	17.804	-64.628	Jeffrey, Chris	5.5	0.0%				12.85	13.35
1290	H28	St. Croix	US Virgin Islands - St. Croix	27-Oct-05	17.803	-64.603	Jeffrey, Chris	13.1	88.7%				12.85	13.35
1291	H31	St. Croix	US Virgin Islands - St. Croix	27-Oct-05	17.794	-64.583	Jeffrey, Chris	13.7	65.1%				12.85	13.35
1292	H33	St. Croix	US Virgin Islands - St. Croix	27-Oct-05	17.802	-64.595	Jeffrey, Chris	14.9	45.3%				12.85	13.35
1293	H37	St. Croix	US Virgin Islands - St. Croix	27-Oct-05	17.802	-64.599	Jeffrey, Chris	12.8	63.0%				12.85	13.35
1294	H42	St. Croix	US Virgin Islands - St. Croix	27-Oct-05	17.785	-64.588	Jeffrey, Chris	8.2	34.6%				12.85	13.35
1295	H5	St. Croix	US Virgin Islands - St. Croix	27-Oct-05	17.801	-64.600	Jeffrey, Chris	12.5	28.1%				12.85	13.35
1296	H79	St. Croix	US Virgin Islands - St. Croix	27-Oct-05	17.767	-64.639	Jeffrey, Chris	4.6	40.1%				12.85	13.35
1297	H93	St. Croix	US Virgin Islands - St. Croix	27-Oct-05	17.766	-64.628	Jeffrey, Chris	4.9	57.2%				12.85	13.35
776	Eagle Ray	Christiansted Harbor	US Virgin Islands - St. Croix	2-Nov-05	17.761	-64.699	Carr, Liam	10		66.7%			12.85	13.35
777	Eagle Ray	Christiansted Harbor	US Virgin Islands - St. Croix	2-Nov-05	17.761	-64.699	Carr, Liam	10		85.7%			12.85	13.35
778	Eagle Ray	Christiansted Harbor	US Virgin Islands - St. Croix	2-Nov-05	17.761	-64.699	Carr, Liam	10		87.5%			12.85	13.35
779	Eagle Ray	Christiansted Harbor	US Virgin Islands - St. Croix	2-Nov-05	17.761	-64.699	Carr, Liam	10		100.0%			12.85	13.35
780	Eagle Ray	Christiansted Harbor	US Virgin Islands - St. Croix	2-Nov-05	17.761	-64.699	Carr, Liam	10		66.7%			12.85	13.35
781	Eagle Ray	Christiansted Harbor	US Virgin Islands - St. Croix	2-Nov-05	17.761	-64.699	Carr, Liam	10		100.0%			12.85	13.35
2668	Eagle Ray	No. shore	US Virgin Islands - St. Croix	2-Nov-05	17.762	-64.699	Taylor, Marcia	9	56.0%				12.85	13.35
2669	Eagle Ray	No. shore	US Virgin Islands - St. Croix	2-Nov-05	17.762	-64.699	Taylor, Marcia	9	36.0%				12.85	13.35
2670	Eagle Ray	No. shore	US Virgin Islands - St. Croix	2-Nov-05	17.762	-64.699	Taylor, Marcia	9	61.0%				12.85	13.35
2671	Eagle Ray	No. shore	US Virgin Islands - St. Croix	2-Nov-05	17.762	-64.699	Taylor, Marcia	9	73.0%				12.85	13.35
2672	Eagle Ray	No. shore	US Virgin Islands - St. Croix	2-Nov-05	17.762	-64.699	Taylor, Marcia	9	61.0%				12.85	13.35
2673	Eagle Ray	No. shore	US Virgin Islands - St. Croix	2-Nov-05	17.762	-64.699	Taylor, Marcia	9	55.0%				12.85	13.35
1750	S. Fore Reef	Buck Island	US Virgin Islands - St. Croix	9-Nov-05	17.785	-64.610	Miller, Jeff	14.05	96.0%				12.3	13.35
782	Lang Bank	Lang Bank	US Virgin Islands - St. Croix	21-Nov-05	17.824	-64.449	Carr, Liam	30	91.7%	77.8%			10.1	13.35
783	Lang Bank	Lang Bank	US Virgin Islands - St. Croix	21-Nov-05	17.824	-64.449	Carr, Liam	30	83.3%	75.0%			10.1	13.35
784	Lang Bank	Lang Bank	US Virgin Islands - St. Croix	21-Nov-05	17.824	-64.449	Carr, Liam	30	100.0%	100.0%			10.1	13.35
785	Lang Bank	Lang Bank	US Virgin Islands - St. Croix	21-Nov-05	17.824	-64.449	Carr, Liam	30	83.3%	100.0%			10.1	13.35
786	Lang Bank	Lang Bank	US Virgin Islands - St. Croix	21-Nov-05	17.824	-64.449	Carr, Liam	30		76.9%			10.1	13.35
787	Lang Bank	Lang Bank	US Virgin Islands - St. Croix	21-Nov-05	17.824	-64.449	Carr, Liam	30		80.0%			10.1	13.35
788	Great Pond	Great Pond	US Virgin Islands - St. Croix	22-Nov-05	17.711	-64.652	Carr, Liam	5		85.7%			10.1	13.35
789	Great Pond	Great Pond	US Virgin Islands - St. Croix	22-Nov-05	17.711	-64.652	Carr, Liam	5		100.0%		20.0%	10.1	13.35
790	Great Pond	Great Pond	US Virgin Islands - St. Croix	22-Nov-05	17.711	-64.652	Carr, Liam	5		66.7%			10.1	13.35
791	Great Pond	Great Pond	US Virgin Islands - St. Croix	22-Nov-05	17.711	-64.652	Carr, Liam	5		33.3%		16.7%	10.1	13.35
792	Great Pond	Great Pond	US Virgin Islands - St. Croix	22-Nov-05	17.711	-64.652	Carr, Liam	5		80.0%		20.0%	10.1	13.35
793	Great Pond	Great Pond	US Virgin Islands - St. Croix	22-Nov-05	17.711	-64.652	Carr, Liam	5		37.5%			10.1	13.35
794	Jacks Bay	Jacks Bay	US Virgin Islands - St. Croix	22-Nov-05	17.743	-64.572	Carr, Liam	13		83.3%			10.1	13.35
795	Jacks Bay	Jacks Bay	US Virgin Islands - St. Croix	22-Nov-05	17.743	-64.572	Carr, Liam	13		75.0%			10.1	13.35
796	Jacks Bay	Jacks Bay	US Virgin Islands - St. Croix	22-Nov-05	17.743	-64.572	Carr, Liam	13		66.7%			10.1	13.35
797	Jacks Bay	Jacks Bay	US Virgin Islands - St. Croix	22-Nov-05	17.743	-64.572	Carr, Liam	13		66.7%			10.1	13.35
798	Jacks Bay	Jacks Bay	US Virgin Islands - St. Croix	22-Nov-05	17.743	-64.572	Carr, Liam	13		100.0%			10.1	13.35
799	Jacks Bay	Jacks Bay	US Virgin Islands - St. Croix	22-Nov-05	17.743	-64.572	Carr, Liam	13		100.0%			10.1	13.35
2541	Cane Bay	Cane Bay	US Virgin Islands - St. Croix	23-Nov-05	17.774	-64.814	Smith, Tyler	40		76.9%		0.0%	8.75	10.25
2542	Cane Bay	Cane Bay	US Virgin Islands - St. Croix	23-Nov-05	17.774	-64.814	Smith, Tyler	40		81.8%		0.0%	8.75	10.25
2543	Cane Bay	Cane Bay	US Virgin Islands - St. Croix	23-Nov-05	17.774	-64.814	Smith, Tyler	27.5		100.0%		0.0%	8.75	10.25
2544	Cane Bay	Cane Bay	US Virgin Islands - St. Croix	23-Nov-05	17.774	-64.814	Smith, Tyler	40		69.6%		0.0%	8.75	10.25
2545	Cane Bay	Cane Bay	US Virgin Islands - St. Croix	23-Nov-05	17.774	-64.814	Smith, Tyler	27.5		78.6%		0.0%	8.75	10.25
2546	Cane Bay	Cane Bay	US Virgin Islands - St. Croix	23-Nov-05	17.774	-64.814	Smith, Tyler	21.5		90.0%		0.0%	8.75	10.25
2547	Cane Bay	Cane Bay	US Virgin Islands - St. Croix	23-Nov-05	17.774	-64.814	Smith, Tyler	21.5		78.6%		0.0%	8.75	10.25
2548	Cane Bay	Cane Bay	US Virgin Islands - St. Croix	23-Nov-05	17.774	-64.814	Smith, Tyler	21.5		91.7%		0.0%	8.75	10.25
2549	Cane Bay	Cane Bay	US Virgin Islands - St. Croix	23-Nov-05	17.774	-64.814	Smith, Tyler	39		100.0%		0.0%	8.75	10.25
2550	Cane Bay	Cane Bay	US Virgin Islands - St. Croix	23-Nov-05	17.774	-64.814	Smith, Tyler	39		100.0%		0.0%	8.75	10.25

Observation Number	Reef or Site Name	Location	Country	Date (DD-MMM-YY)	Latitude (decimal degrees)	Longitude (decimal degrees)	Primary Contributor	Depth (m)	Cover bleached (%)	Colonies bleached (%)	Cover dead (%)	Colonies dead (%)	Observed DHW (°C-weeks)	Maximum DHW (°C-weeks)
2551	Cane Bay	Cane Bay	US Virgin Islands - St. Croix	23-Nov-05	17.774	-64.814	Smith, Tyler	29		100.0%		0.0%	8.75	10.25
2552	Cane Bay	Cane Bay	US Virgin Islands - St. Croix	23-Nov-05	17.774	-64.814	Smith, Tyler	29		100.0%		0.0%	8.75	10.25
2553	Cane Bay	Cane Bay	US Virgin Islands - St. Croix	23-Nov-05	17.774	-64.814	Smith, Tyler	20.5		80.0%		0.0%	8.75	10.25
2554	Cane Bay	Cane Bay	US Virgin Islands - St. Croix	23-Nov-05	17.774	-64.814	Smith, Tyler	21.5		44.4%		0.0%	8.75	10.25
2841	PI11	St. Croix	US Virgin Islands - St. Croix	1-Dec-05	17.787	-64.604	Woody, Kimberly	12.192	60.0%				9.55	13.35
2842	PI12	St. Croix	US Virgin Islands - St. Croix	1-Dec-05	17.780	-64.590	Woody, Kimberly	7.3152	0.0%				9.55	13.35
2843	PI14	St. Croix	US Virgin Islands - St. Croix	1-Dec-05	17.795	-64.627	Woody, Kimberly	7.3152	21.0%				9.55	13.35
2844	PI15	St. Croix	US Virgin Islands - St. Croix	1-Dec-05	17.793	-64.620	Woody, Kimberly	7.9248	4.0%				9.55	13.35
2845	PI16	St. Croix	US Virgin Islands - St. Croix	1-Dec-05	17.798	-64.617	Woody, Kimberly	11.5824	28.0%				9.55	13.35
2846	PI4	St. Croix	US Virgin Islands - St. Croix	1-Dec-05	17.804	-64.618	Woody, Kimberly	5.7912	10.0%				9.55	13.35
2847	PI5	St. Croix	US Virgin Islands - St. Croix	1-Dec-05	17.782	-64.643	Woody, Kimberly	10.668	10.0%				9.55	13.35
2848	PI7	St. Croix	US Virgin Islands - St. Croix	1-Dec-05	17.783	-64.584	Woody, Kimberly	13.4112	22.0%				9.55	13.35
2849	PI8	St. Croix	US Virgin Islands - St. Croix	1-Dec-05	17.803	-64.628	Woody, Kimberly	5.7912	78.0%				9.55	13.35
2850	PO32	St. Croix	US Virgin Islands - St. Croix	1-Dec-05	17.767	-64.597	Woody, Kimberly	5.7912	4.0%				9.55	13.35
2851	PO33	St. Croix	US Virgin Islands - St. Croix	1-Dec-05	17.783	-64.571	Woody, Kimberly	20.1168	26.0%				9.55	13.35
2852	PO34	St. Croix	US Virgin Islands - St. Croix	1-Dec-05	17.780	-64.582	Woody, Kimberly	10.9728	10.0%				9.55	13.35
2853	PO35	St. Croix	US Virgin Islands - St. Croix	1-Dec-05	17.777	-64.660	Woody, Kimberly	7.62	23.0%				9.55	13.35
2854	PO41	St. Croix	US Virgin Islands - St. Croix	1-Dec-05	17.771	-64.585	Woody, Kimberly	17.6784	34.0%				9.55	13.35
2855	PO44	St. Croix	US Virgin Islands - St. Croix	1-Dec-05	17.769	-64.585	Woody, Kimberly	7.3152	45.0%				9.55	13.35
2856	PO46	St. Croix	US Virgin Islands - St. Croix	1-Dec-05	17.767	-64.567	Woody, Kimberly	18.8976	29.0%				9.55	13.35
2857	PO47	St. Croix	US Virgin Islands - St. Croix	1-Dec-05	17.769	-64.590	Woody, Kimberly	6.096	47.0%				9.55	13.35
2858	PO49	St. Croix	US Virgin Islands - St. Croix	1-Dec-05	17.773	-64.610	Woody, Kimberly	13.4112	3.0%				9.55	13.35
2557	Lang Bank	St. Croix (USVI)	US Virgin Islands - St. Croix	12-Jan-06	17.824	-64.449	Smith, Tyler	30		84.9%		0.0%	2.25	13.35
800	Buck Island	Buck Island	US Virgin Islands - St. Croix	13-Jan-06	17.785	-64.609	Carr, Liam	12		16.7%			0.5	13.35
801	Buck Island	Buck Island	US Virgin Islands - St. Croix	13-Jan-06	17.785	-64.609	Carr, Liam	12		57.1%			0.5	13.35
802	Buck Island	Buck Island	US Virgin Islands - St. Croix	13-Jan-06	17.785	-64.609	Carr, Liam	12		71.4%			0.5	13.35
803	Buck Island	Buck Island	US Virgin Islands - St. Croix	13-Jan-06	17.785	-64.609	Carr, Liam	12		58.3%			0.5	13.35
804	Buck Island	Buck Island	US Virgin Islands - St. Croix	13-Jan-06	17.785	-64.609	Carr, Liam	12		62.5%			0.5	13.35
2558	Lang Bank	St. Croix (USVI)	US Virgin Islands - St. Croix	14-Jan-06	17.824	-64.449	Smith, Tyler	30		72.7%		4.5%	0.5	13.35
2561	Cane Bay	St. Croix (USVI)	US Virgin Islands - St. Croix	7-Feb-06	17.774	-64.814	Smith, Tyler	20.5		31.5%		0.0%	0	10.25
2562	Lang Bank	St. Croix (USVI)	US Virgin Islands - St. Croix	10-Feb-06	17.824	-64.449	Smith, Tyler	30		47.0%		0.0%	0	13.35
2564	Salt River	St. Croix (USVI)	US Virgin Islands - St. Croix	26-Feb-06	17.785	-64.759	Smith, Tyler	6		12.5%		0.0%	0	10.25
2565	Eagle ray	St. Croix (USVI)	US Virgin Islands - St. Croix	28-Feb-06	17.761	-64.699	Smith, Tyler	10		18.4%		0.0%	0	13.35
2566	Buck Island, STX	St. Croix (USVI)	US Virgin Islands - St. Croix	3-Mar-06	17.785	-64.609	Smith, Tyler	12	0.6%	27.3%	4.2%	0.0%	0	13.35
2567	Mutton	St. Croix (USVI)	US Virgin Islands - St. Croix	13-Mar-06	17.637	-64.862	Smith, Tyler	23		29.0%		0.0%	0	13.35
2568	Kings	St. Croix (USVI)	US Virgin Islands - St. Croix	15-Mar-06	17.743	-64.572	Smith, Tyler	13		15.4%		0.0%	0	13.35
2053	Cane Bay	St Croix	US Virgin Islands - St. Croix	2-Apr-06	17.767	-64.828	Quinn, Norman	13	59.0%	59.0%	13.0%	13.0%	0	10.25
2859	3194	St. Croix	US Virgin Islands - St. Croix	10-Apr-06	17.769	-64.583	Woody, Kimberly	12.4968	50.9%				0	13.35
2860	3206	St. Croix	US Virgin Islands - St. Croix	11-Apr-06	17.779	-64.655	Woody, Kimberly	8.41248	0.0%				0	13.35
2861	3209	St. Croix	US Virgin Islands - St. Croix	11-Apr-06	17.768	-64.614	Woody, Kimberly	13.22832	0.0%				0	13.35
2862	3214	St. Croix	US Virgin Islands - St. Croix	11-Apr-06	17.777	-64.587	Woody, Kimberly	10.668	0.0%				0	13.35
2863	3254	St. Croix	US Virgin Islands - St. Croix	11-Apr-06	17.786	-64.612	Woody, Kimberly	1.76784	9.8%				0	13.35
2864	3257	St. Croix	US Virgin Islands - St. Croix	11-Apr-06	17.789	-64.616	Woody, Kimberly	2.1336	5.2%				0	13.35
2865	3258	St. Croix	US Virgin Islands - St. Croix	11-Apr-06	17.790	-64.615	Woody, Kimberly	3.77952	0.0%				0	13.35
2866	3144	St. Croix	US Virgin Islands - St. Croix	12-Apr-06	17.784	-64.609	Woody, Kimberly	14.50848	51.8%				0	13.35
2867	3160	St. Croix	US Virgin Islands - St. Croix	12-Apr-06	17.783	-64.599	Woody, Kimberly	10.48512	0.0%				0	13.35
2868	3164	St. Croix	US Virgin Islands - St. Croix	12-Apr-06	17.782	-64.601	Woody, Kimberly	5.97408	28.6%				0	13.35
2869	3200	St. Croix	US Virgin Islands - St. Croix	12-Apr-06	17.776	-64.659	Woody, Kimberly	7.80288	0.0%				0	13.35
2870	3204	St. Croix	US Virgin Islands - St. Croix	12-Apr-06	17.781	-64.584	Woody, Kimberly	7.9248	0.0%				0	13.35
2871	3217	St. Croix	US Virgin Islands - St. Croix	12-Apr-06	17.779	-64.586	Woody, Kimberly	7.62	0.0%				0	13.35
2872	3218	St. Croix	US Virgin Islands - St. Croix	12-Apr-06	17.776	-64.670	Woody, Kimberly	6.096	10.4%				0	13.35
2873	3232	St. Croix	US Virgin Islands - St. Croix	12-Apr-06	17.778	-64.669	Woody, Kimberly	6.58368	14.1%				0	13.35
2874	3240	St. Croix	US Virgin Islands - St. Croix	12-Apr-06	17.774	-64.659	Woody, Kimberly	9.08304	0.0%				0	13.35
2875	3143	St. Croix	US Virgin Islands - St. Croix	13-Apr-06	17.807	-64.625	Woody, Kimberly	12.8016	16.7%				0	13.35
2876	3149	St. Croix	US Virgin Islands - St. Croix	13-Apr-06	17.806	-64.633	Woody, Kimberly	8.59536	37.5%				0	13.35
2877	3152	St. Croix	US Virgin Islands - St. Croix	13-Apr-06	17.794	-64.626	Woody, Kimberly	4.08432	11.9%				0	13.35
2878	3156	St. Croix	US Virgin Islands - St. Croix	13-Apr-06	17.794	-64.629	Woody, Kimberly	3.77952	0.0%				0	13.35
2879	3157	St. Croix	US Virgin Islands - St. Croix	13-Apr-06	17.795	-64.622	Woody, Kimberly	11.21664	1.3%				0	13.35

Observation Number	Reef or Site Name	Location	Country	Date (DD-MMM-YY)	Latitude (decimal degrees)	Longitude (decimal degrees)	Primary Contributor	Depth (m)	Cover bleached (%)	Colonies bleached (%)	Cover dead (%)	Colonies dead (%)	Observed DHW (°C-weeks)	Maximum DHW (°C-weeks)
2880	3167	St. Croix	US Virgin Islands - St. Croix	13-Apr-06	17.780	-64.587	Woody, Kimberly	6.88848	1.0%				0	13.35
2881	3173	St. Croix	US Virgin Islands - St. Croix	13-Apr-06	17.801	-64.624	Woody, Kimberly	2.86512	1.1%				0	13.35
2882	3177	St. Croix	US Virgin Islands - St. Croix	13-Apr-06	17.792	-64.629	Woody, Kimberly	4.63296	3.8%				0	13.35
2883	3193	St. Croix	US Virgin Islands - St. Croix	13-Apr-06	17.776	-64.586	Woody, Kimberly	14.81328	0.0%				0	13.35
2884	3210	St. Croix	US Virgin Islands - St. Croix	13-Apr-06	17.778	-64.574	Woody, Kimberly	14.87424	3.2%				0	13.35
2885	3216	St. Croix	US Virgin Islands - St. Croix	13-Apr-06	17.777	-64.652	Woody, Kimberly	8.59536	0.0%				0	13.35
2886	3142	St. Croix	US Virgin Islands - St. Croix	14-Apr-06	17.794	-64.618	Woody, Kimberly	9.81456	8.9%				0	13.35
2887	3145	St. Croix	US Virgin Islands - St. Croix	14-Apr-06	17.794	-64.616	Woody, Kimberly	11.2776	26.7%				0	13.35
2888	3147	St. Croix	US Virgin Islands - St. Croix	14-Apr-06	17.796	-64.618	Woody, Kimberly	11.09472	35.8%				0	13.35
2889	3153	St. Croix	US Virgin Islands - St. Croix	14-Apr-06	17.787	-64.587	Woody, Kimberly	9.144	0.0%				0	13.35
2890	3154	St. Croix	US Virgin Islands - St. Croix	14-Apr-06	17.792	-64.585	Woody, Kimberly	10.48512	15.0%				0	13.35
2891	3166	St. Croix	US Virgin Islands - St. Croix	14-Apr-06	17.802	-64.618	Woody, Kimberly	4.572	35.3%				0	13.35
2892	3170	St. Croix	US Virgin Islands - St. Croix	14-Apr-06	17.796	-64.579	Woody, Kimberly	18.47088	0.0%				0	13.35
2893	3172	St. Croix	US Virgin Islands - St. Croix	14-Apr-06	17.800	-64.629	Woody, Kimberly	3.41376	11.4%				0	13.35
2894	3175	St. Croix	US Virgin Islands - St. Croix	14-Apr-06	17.796	-64.614	Woody, Kimberly	11.8872	29.3%				0	13.35
2895	3179	St. Croix	US Virgin Islands - St. Croix	14-Apr-06	17.789	-64.579	Woody, Kimberly	14.69136	20.4%				0	13.35
2896	3190	St. Croix	US Virgin Islands - St. Croix	15-Apr-06	17.764	-64.613	Woody, Kimberly	0.9144	0.0%				0	13.35
2897	3222	St. Croix	US Virgin Islands - St. Croix	15-Apr-06	17.765	-64.655	Woody, Kimberly	3.29184	0.0%				0	13.35
2898	3146	St. Croix	US Virgin Islands - St. Croix	17-Apr-06	17.798	-64.599	Woody, Kimberly	7.37616	0.0%				0	13.35
2899	3158	St. Croix	US Virgin Islands - St. Croix	17-Apr-06	17.793	-64.592	Woody, Kimberly	8.8392	3.7%				0	13.35
2900	3161	St. Croix	US Virgin Islands - St. Croix	17-Apr-06	17.794	-64.597	Woody, Kimberly	7.86384	11.2%				0	13.35
2901	3165	St. Croix	US Virgin Islands - St. Croix	17-Apr-06	17.799	-64.600	Woody, Kimberly	7.3152	0.0%				0	13.35
2902	3174	St. Croix	US Virgin Islands - St. Croix	17-Apr-06	17.798	-64.607	Woody, Kimberly	6.03504	10.3%				0	13.35
2903	3176	St. Croix	US Virgin Islands - St. Croix	17-Apr-06	17.804	-64.602	Woody, Kimberly	19.32432	1.7%				0	13.35
2904	3148	St. Croix	US Virgin Islands - St. Croix	18-Apr-06	17.790	-64.584	Woody, Kimberly	10.54608	16.1%				0	13.35
2905	3150	St. Croix	US Virgin Islands - St. Croix	18-Apr-06	17.806	-64.607	Woody, Kimberly	16.70304	13.8%				0	13.35
2906	3151	St. Croix	US Virgin Islands - St. Croix	18-Apr-06	17.806	-64.612	Woody, Kimberly	13.28928	12.8%				0	13.35
2907	3163	St. Croix	US Virgin Islands - St. Croix	18-Apr-06	17.803	-64.595	Woody, Kimberly	28.3464	12.4%				0	13.35
2908	3201	St. Croix	US Virgin Islands - St. Croix	18-Apr-06	17.765	-64.583	Woody, Kimberly	3.59664	0.0%				0	13.35
2909	3203	St. Croix	US Virgin Islands - St. Croix	18-Apr-06	17.761	-64.569	Woody, Kimberly	9.69264	0.0%				0	13.35
2910	3215	St. Croix	US Virgin Islands - St. Croix	18-Apr-06	17.777	-64.671	Woody, Kimberly	6.4008	4.9%				0	13.35
2911	3219	St. Croix	US Virgin Islands - St. Croix	18-Apr-06	17.760	-64.568	Woody, Kimberly	9.02208	50.9%				0	13.35
2912	3224	St. Croix	US Virgin Islands - St. Croix	18-Apr-06	17.776	-64.670	Woody, Kimberly	6.4008	0.0%				0	13.35
2913	3225	St. Croix	US Virgin Islands - St. Croix	18-Apr-06	17.763	-64.572	Woody, Kimberly	6.4008	8.6%				0	13.35
2914	3226	St. Croix	US Virgin Islands - St. Croix	18-Apr-06	17.778	-64.567	Woody, Kimberly	23.53056	0.0%				0	13.35
2915	3228	St. Croix	US Virgin Islands - St. Croix	18-Apr-06	17.771	-64.664	Woody, Kimberly	7.19328	0.0%				0	13.35
2916	3229	St. Croix	US Virgin Islands - St. Croix	18-Apr-06	17.762	-64.571	Woody, Kimberly	8.5344	0.0%				0	13.35
2917	3231	St. Croix	US Virgin Islands - St. Croix	18-Apr-06	17.774	-64.565	Woody, Kimberly	23.89632	0.0%				0	13.35
2918	3141	St. Croix	US Virgin Islands - St. Croix	19-Apr-06	17.797	-64.612	Woody, Kimberly	10.60704	53.9%				0	13.35
2919	3159	St. Croix	US Virgin Islands - St. Croix	19-Apr-06	17.799	-64.634	Woody, Kimberly	6.94944	4.2%				0	13.35
2920	3168	St. Croix	US Virgin Islands - St. Croix	19-Apr-06	17.801	-64.617	Woody, Kimberly	8.04672	83.2%				0	13.35
2921	3169	St. Croix	US Virgin Islands - St. Croix	19-Apr-06	17.789	-64.606	Woody, Kimberly	13.4112	5.8%				0	13.35
2922	3171	St. Croix	US Virgin Islands - St. Croix	19-Apr-06	17.798	-64.571	Woody, Kimberly	26.02992	0.0%				0	13.35
2923	3178	St. Croix	US Virgin Islands - St. Croix	19-Apr-06	17.791	-64.633	Woody, Kimberly	4.2672	3.6%				0	13.35
2924	3191	St. Croix	US Virgin Islands - St. Croix	19-Apr-06	17.766	-64.583	Woody, Kimberly	10.3632	0.0%				0	13.35
2925	3192	St. Croix	US Virgin Islands - St. Croix	19-Apr-06	17.769	-64.566	Woody, Kimberly	22.5552	0.0%				0	13.35
2926	3196	St. Croix	US Virgin Islands - St. Croix	19-Apr-06	17.767	-64.583	Woody, Kimberly	10.0584	0.0%				0	13.35
2927	3205	St. Croix	US Virgin Islands - St. Croix	19-Apr-06	17.789	-64.570	Woody, Kimberly	20.7264	21.3%				0	13.35
2928	3207	St. Croix	US Virgin Islands - St. Croix	19-Apr-06	17.766	-64.588	Woody, Kimberly	8.47344	1.6%				0	13.35
2929	3223	St. Croix	US Virgin Islands - St. Croix	19-Apr-06	17.769	-64.564	Woody, Kimberly	23.40864	0.0%				0	13.35
2930	3227	St. Croix	US Virgin Islands - St. Croix	19-Apr-06	17.779	-64.574	Woody, Kimberly	12.43584	5.8%				0	13.35
2931	3155	St. Croix	US Virgin Islands - St. Croix	20-Apr-06	17.797	-64.583	Woody, Kimberly	16.64208	30.0%				0	13.35
2932	3162	St. Croix	US Virgin Islands - St. Croix	20-Apr-06	17.792	-64.580	Woody, Kimberly	17.6784	48.8%				0	13.35
2933	3180	St. Croix	US Virgin Islands - St. Croix	20-Apr-06	17.792	-64.577	Woody, Kimberly	18.288	28.2%				0	13.35
2934	3198	St. Croix	US Virgin Islands - St. Croix	20-Apr-06	17.770	-64.664	Woody, Kimberly	5.66928	0.0%				0	13.35
2935	3202	St. Croix	US Virgin Islands - St. Croix	20-Apr-06	17.762	-64.597	Woody, Kimberly	1.03632	0.0%				0	13.35
2936	3220	St. Croix	US Virgin Islands - St. Croix	20-Apr-06	17.788	-64.565	Woody, Kimberly	21.82368	26.3%				0	13.35
2937	3221	St. Croix	US Virgin Islands - St. Croix	20-Apr-06	17.775	-64.656	Woody, Kimberly	8.59536	7.0%				0	13.35

Observation Number	Reef or Site Name	Location	Country	Date (DD-MMM-YY)	Latitude (decimal degrees)	Longitude (decimal degrees)	Primary Contributor	Depth (m)	Cover bleached (%)	Colonies bleached (%)	Cover dead (%)	Colonies dead (%)	Observed DHW (°C-weeks)	Maximum DHW (°C-weeks)
2938	3243	St. Croix	US Virgin Islands - St. Croix	20-Apr-06	17.764	-64.617	Woody, Kimberly	1.88976	0.0%				0	13.35
2939	3261	St. Croix	US Virgin Islands - St. Croix	20-Apr-06	17.778	-64.657	Woody, Kimberly	8.10768	0.0%				0	13.35
2940	3262	St. Croix	US Virgin Islands - St. Croix	20-Apr-06	17.767	-64.666	Woody, Kimberly	2.1336	0.0%				0	13.35
2941	3251	St. Croix	US Virgin Islands - St. Croix	21-Apr-06	17.791	-64.622	Woody, Kimberly	2.92608	0.0%				0	13.35
2942	3253	St. Croix	US Virgin Islands - St. Croix	21-Apr-06	17.792	-64.624	Woody, Kimberly	3.048	0.0%				0	13.35
2943	3255	St. Croix	US Virgin Islands - St. Croix	21-Apr-06	17.793	-64.628	Woody, Kimberly	4.81584	51.7%				0	13.35
2944	3259	St. Croix	US Virgin Islands - St. Croix	21-Apr-06	17.791	-64.625	Woody, Kimberly	1.40208	0.0%				0	13.35
2945	3263	St. Croix	US Virgin Islands - St. Croix	21-Apr-06	17.767	-64.638	Woody, Kimberly	6.33984	0.0%				0	13.35
2600	Lang Bank	St. Croix (USVI)	US Virgin Islands - St. Croix	7-Sep-06	17.824	-64.449	Smith, Tyler	30	2.6%	24.3%	0.1%	0.0%	0	13.35
2601	Eagle ray	St. Croix (USVI)	US Virgin Islands - St. Croix	5-Dec-06	17.761	-64.699	Smith, Tyler	10	0.0%	17.0%	1.8%	0.0%	2.6	13.35
2602	Jacks Bay	St. Croix (USVI)	US Virgin Islands - St. Croix	6-Dec-06	17.743	-64.572	Smith, Tyler	13	0.0%	3.7%	1.9%	0.0%	2.6	13.35
2603	Cane Bay	St. Croix (USVI)	US Virgin Islands - St. Croix	7-Dec-06	17.774	-64.814	Smith, Tyler	20.5	9.3%	18.8%	13.5%	0.0%	2.7	10.25
2604	Salt River	St. Croix (USVI)	US Virgin Islands - St. Croix	7-Dec-06	17.785	-64.759	Smith, Tyler	6	0.0%	15.8%	1.4%	0.0%	2.7	10.25
2605	Great Pond	St. Croix (USVI)	US Virgin Islands - St. Croix	9-Dec-06	17.711	-64.652	Smith, Tyler	5	0.0%	3.1%	7.2%	0.0%	2.1	13.35
2606	Mutton	St. Croix (USVI)	US Virgin Islands - St. Croix	9-Dec-06	17.637	-64.862	Smith, Tyler	23	0.0%	4.2%	31.7%	0.0%	2.1	13.35
2607	Buck Island, STX	St. Croix (USVI)	US Virgin Islands - St. Croix	10-Dec-06	17.785	-64.609	Smith, Tyler	12	0.0%	0.0%	11.1%	0.0%	2.1	13.35
2608	Sprat	St. Croix (USVI)	US Virgin Islands - St. Croix	20-Jan-07	17.734	-64.895	Smith, Tyler	10	0.0%	65.4%	16.0%	0.0%	99.99	13.35
2104	Tektite Reef	Tektite Reef	US Virgin Islands - St. John	1-Sep-05	18.308	-64.722	ReefBase	5	90.2%				2	10.25
1747	Yawzi Reef	St. John	US Virgin Islands - St. John	19-Sep-05	18.320	-64.730	Miller, Jeff	12.85	70.9%				4.65	10.25
1748	Tektite Reef	St. John	US Virgin Islands - St. John	29-Sep-05	18.310	-64.720	Miller, Jeff	12.65	97.0%				6.7	10.25
1758	Haulover Bay	St. John	US Virgin Islands - St. John	29-Sep-05	18.351	-64.680	Muller, Erinn	3	96.0%	60.8%		6.3%	6.7	10.25
1759	Hawksnest Bay	St. John	US Virgin Islands - St. John	30-Sep-05	18.348	-64.781	Muller, Erinn	3		51.8%		16.1%	6.7	10.25
1760	Salt Pond	St. John	US Virgin Islands - St. John	5-Oct-05	18.307	-64.708	Muller, Erinn	3		39.9%		7.0%	7.45	10.25
2504	Fish Bay	Fish Bay	US Virgin Islands - St. John	6-Oct-05	18.314	-64.764	Smith, Tyler	6	43.8%	80.0%		0.0%	7.45	10.25
2505	Fish Bay	Fish Bay	US Virgin Islands - St. John	6-Oct-05	18.314	-64.764	Smith, Tyler	6	66.7%	100.0%		0.0%	7.45	10.25
2506	Fish Bay	Fish Bay	US Virgin Islands - St. John	6-Oct-05	18.314	-64.764	Smith, Tyler	6	96.7%	100.0%		0.0%	7.45	10.25
2507	Fish Bay	Fish Bay	US Virgin Islands - St. John	6-Oct-05	18.314	-64.764	Smith, Tyler	6	92.6%	100.0%		0.0%	7.45	10.25
2508	Fish Bay	Fish Bay	US Virgin Islands - St. John	6-Oct-05	18.314	-64.764	Smith, Tyler	6	81.8%	100.0%		0.0%	7.45	10.25
2509	Fish Bay	Fish Bay	US Virgin Islands - St. John	6-Oct-05	18.314	-64.764	Smith, Tyler	6	90.5%	100.0%		0.0%	7.45	10.25
2510	S. Fish	S. Fish	US Virgin Islands - St. John	6-Oct-05	18.244	-64.758	Smith, Tyler	33	30.1%	67.7%			7.45	10.25
2511	S. Fish	S. Fish	US Virgin Islands - St. John	6-Oct-05	18.244	-64.758	Smith, Tyler	33	31.7%	0.0%			7.45	10.25
2512	S. Fish	S. Fish	US Virgin Islands - St. John	6-Oct-05	18.244	-64.758	Smith, Tyler	33	10.1%	43.5%			7.45	10.25
2513	S. Fish	S. Fish	US Virgin Islands - St. John	6-Oct-05	18.244	-64.758	Smith, Tyler	33	10.5%	79.2%			7.45	10.25
2514	S. Fish	S. Fish	US Virgin Islands - St. John	6-Oct-05	18.244	-64.758	Smith, Tyler	33	27.2%	80.0%			7.45	10.25
2515	S. Fish	S. Fish	US Virgin Islands - St. John	6-Oct-05	18.244	-64.758	Smith, Tyler	33	42.4%	72.7%			7.45	10.25
1761	Trunk Bay	St. John	US Virgin Islands - St. John	12-Oct-05	18.354	-64.769	Muller, Erinn	4		34.1%		5.9%	8.85	10.25
1749	Newfound Reef	St. John	US Virgin Islands - St. John	1-Nov-05	18.350	-64.670	Miller, Jeff	9.15	91.8%				10.25	10.25
2580	Fish Bay	St. Johns (USVI)	US Virgin Islands - St. John	28-Apr-06	18.314	-64.764	Smith, Tyler	6		38.2%		0.0%	0	10.25
2581	S. Fish	St. Johns (USVI)	US Virgin Islands - St. John	28-Apr-06	18.244	-64.758	Smith, Tyler	33		47.4%		0.0%	0	10.25
2584	Fish Bay	St. Johns (USVI)	US Virgin Islands - St. John	1-Jun-06	18.314	-64.764	Smith, Tyler	6		0.0%		0.0%	0	10.25
2590	Fish Bay	St. Johns (USVI)	US Virgin Islands - St. John	3-Jul-06	18.314	-64.764	Smith, Tyler	6	1.6%	28.2%	1.6%	0.0%	0	10.25
2591	S. Fish	St. Johns (USVI)	US Virgin Islands - St. John	3-Jul-06	18.244	-64.758	Smith, Tyler	33		33.3%		0.0%	0	10.25
2025	Saba Is	St Thomas	US Virgin Islands - St. Thomas	6-Aug-05	18.328	-65.004	Quinn, Norman	10	0.0%	0.0%	0.0%	0.0%	0	10.25
2026	Coki Beach	St Thomas	US Virgin Islands - St. Thomas	8-Aug-05	18.350	-64.868	Quinn, Norman	5	0.0%	0.0%	0.0%	0.0%	0	10.25
2321	Flat Cay	St. Thomas	US Virgin Islands - St. Thomas	16-Sep-05	18.317	-64.993	Romano, Sandra	12	86.0%		0.0%		3.85	10.25
2322	Flat Cay	St. Thomas	US Virgin Islands - St. Thomas	16-Sep-05	18.317	-64.993	Romano, Sandra	2.5	71.0%		0.0%		3.85	10.25
2444	Black Pt.	Black Pt.	US Virgin Islands - St. Thomas	26-Sep-05	18.345	-64.986	Smith, Tyler	10	88.6%	100.0%		0.0%	5.95	10.25
2445	Black Pt.	Black Pt.	US Virgin Islands - St. Thomas	26-Sep-05	18.345	-64.986	Smith, Tyler	10	65.5%	81.8%		0.0%	5.95	10.25
2446	Black Pt.	Black Pt.	US Virgin Islands - St. Thomas	26-Sep-05	18.345	-64.986	Smith, Tyler	10	79.6%	100.0%		0.0%	5.95	10.25
2447	Black Pt.	Black Pt.	US Virgin Islands - St. Thomas	26-Sep-05	18.345	-64.986	Smith, Tyler	10	37.8%	100.0%		0.0%	5.95	10.25
2448	Black Pt.	Black Pt.	US Virgin Islands - St. Thomas	26-Sep-05	18.345	-64.986	Smith, Tyler	10	78.1%	100.0%		0.0%	5.95	10.25
2449	Black Pt.	Black Pt.	US Virgin Islands - St. Thomas	26-Sep-05	18.345	-64.986	Smith, Tyler	10	53.8%	100.0%		0.0%	5.95	10.25
2450	Buck	Buck	US Virgin Islands - St. Thomas	27-Sep-05	18.279	-64.898	Smith, Tyler	15	59.1%	100.0%		0.0%	5.95	10.25
2451	Buck	Buck	US Virgin Islands - St. Thomas	27-Sep-05	18.279	-64.898	Smith, Tyler	15	81.8%	85.7%		0.0%	5.95	10.25
2452	Buck	Buck	US Virgin Islands - St. Thomas	27-Sep-05	18.279	-64.898	Smith, Tyler	15	95.5%	50.0%		0.0%	5.95	10.25
2453	Buck	Buck	US Virgin Islands - St. Thomas	27-Sep-05	18.279	-64.898	Smith, Tyler	15	66.7%	100.0%		0.0%	5.95	10.25
2454	Buck	Buck	US Virgin Islands - St. Thomas	27-Sep-05	18.279	-64.898	Smith, Tyler	15	71.4%	100.0%		0.0%	5.95	10.25
2455	Buck	Buck	US Virgin Islands - St. Thomas	27-Sep-05	18.279	-64.898	Smith, Tyler	15	86.7%	0.0%		0.0%	5.95	10.25

Observation Number	Reef or Site Name	Location	Country	Date (DD-MMM-YY)	Latitude (decimal degrees)	Longitude (decimal degrees)	Primary Contributor	Depth (m)	Cover bleached (%)	Colonies bleached (%)	Cover dead (%)	Colonies dead (%)	Observed DHW (°C-weeks)	Maximum DHW (°C-weeks)
2456	Flat	Flat	US Virgin Islands - St. Thomas	27-Sep-05	18.318	-64.991	Smith, Tyler	14	57.8%	100.0%			5.95	10.25
2457	Flat	Flat	US Virgin Islands - St. Thomas	27-Sep-05	18.318	-64.991	Smith, Tyler	14	69.4%	94.1%			5.95	10.25
2458	Flat	Flat	US Virgin Islands - St. Thomas	27-Sep-05	18.318	-64.991	Smith, Tyler	14	85.9%	78.9%			5.95	10.25
2459	Flat	Flat	US Virgin Islands - St. Thomas	27-Sep-05	18.318	-64.991	Smith, Tyler	14	68.1%	86.4%			5.95	10.25
2460	Flat	Flat	US Virgin Islands - St. Thomas	27-Sep-05	18.318	-64.991	Smith, Tyler	14	79.2%	90.0%			5.95	10.25
2461	Flat	Flat	US Virgin Islands - St. Thomas	27-Sep-05	18.318	-64.991	Smith, Tyler	14	68.9%	95.0%			5.95	10.25
2462	Benner	Benner	US Virgin Islands - St. Thomas	28-Sep-05	18.313	-64.861	Smith, Tyler	7	66.7%	0.0%		0.0%	5.95	10.25
2463	Benner	Benner	US Virgin Islands - St. Thomas	28-Sep-05	18.313	-64.861	Smith, Tyler	7	78.3%	78.6%		0.0%	5.95	10.25
2464	Benner	Benner	US Virgin Islands - St. Thomas	28-Sep-05	18.313	-64.861	Smith, Tyler	7	42.1%	78.6%		7.1%	5.95	10.25
2465	Benner	Benner	US Virgin Islands - St. Thomas	28-Sep-05	18.313	-64.861	Smith, Tyler	7	80.0%	75.0%		0.0%	5.95	10.25
2466	Benner	Benner	US Virgin Islands - St. Thomas	28-Sep-05	18.313	-64.861	Smith, Tyler	7	60.9%	75.0%		0.0%	5.95	10.25
2467	Benner	Benner	US Virgin Islands - St. Thomas	28-Sep-05	18.313	-64.861	Smith, Tyler	7	31.6%	80.0%		0.0%	5.95	10.25
2468	S. Capella	S. Capella	US Virgin Islands - St. Thomas	28-Sep-05	18.263	-64.872	Smith, Tyler	22	58.0%	90.9%			5.95	10.25
2469	S. Capella	S. Capella	US Virgin Islands - St. Thomas	28-Sep-05	18.263	-64.872	Smith, Tyler	22	56.8%	92.9%			5.95	10.25
2470	S. Capella	S. Capella	US Virgin Islands - St. Thomas	28-Sep-05	18.263	-64.872	Smith, Tyler	22	18.4%	55.6%			5.95	10.25
2471	S. Capella	S. Capella	US Virgin Islands - St. Thomas	28-Sep-05	18.263	-64.872	Smith, Tyler	22	60.0%	62.5%			5.95	10.25
2472	S. Capella	S. Capella	US Virgin Islands - St. Thomas	28-Sep-05	18.263	-64.872	Smith, Tyler	22	31.7%	64.0%			5.95	10.25
2473	S. Capella	S. Capella	US Virgin Islands - St. Thomas	28-Sep-05	18.263	-64.872	Smith, Tyler	22	40.5%	84.2%			5.95	10.25
2474	Botony	Botony	US Virgin Islands - St. Thomas	30-Sep-05	18.358	-65.033	Smith, Tyler	14	73.5%	90.0%		0.0%	6.7	10.25
2475	Botony	Botony	US Virgin Islands - St. Thomas	30-Sep-05	18.358	-65.033	Smith, Tyler	14	63.0%	100.0%		0.0%	6.7	10.25
2476	Botony	Botony	US Virgin Islands - St. Thomas	30-Sep-05	18.358	-65.033	Smith, Tyler	14	71.4%	100.0%		0.0%	6.7	10.25
2477	Botony	Botony	US Virgin Islands - St. Thomas	30-Sep-05	18.358	-65.033	Smith, Tyler	14	68.9%	100.0%		0.0%	6.7	10.25
2478	Botony	Botony	US Virgin Islands - St. Thomas	30-Sep-05	18.358	-65.033	Smith, Tyler	14	93.6%	100.0%		0.0%	6.7	10.25
2479	Botony	Botony	US Virgin Islands - St. Thomas	30-Sep-05	18.358	-65.033	Smith, Tyler	14	98.4%	100.0%		0.0%	6.7	10.25
2480	Grammanik	Grammanik	US Virgin Islands - St. Thomas	4-Oct-05	18.191	-64.950	Smith, Tyler	36	0.0%	27.8%		0.0%	7.45	10.25
2481	Grammanik	Grammanik	US Virgin Islands - St. Thomas	4-Oct-05	18.191	-64.950	Smith, Tyler	36	0.0%	23.5%		0.0%	7.45	10.25
2482	Grammanik	Grammanik	US Virgin Islands - St. Thomas	4-Oct-05	18.191	-64.950	Smith, Tyler	36	0.8%	16.7%		0.0%	7.45	10.25
2483	Grammanik	Grammanik	US Virgin Islands - St. Thomas	4-Oct-05	18.191	-64.950	Smith, Tyler	36	0.0%	0.0%		0.0%	7.45	10.25
2484	Grammanik	Grammanik	US Virgin Islands - St. Thomas	4-Oct-05	18.191	-64.950	Smith, Tyler	36	1.7%	0.0%		0.0%	7.45	10.25
2485	Grammanik	Grammanik	US Virgin Islands - St. Thomas	4-Oct-05	18.191	-64.950	Smith, Tyler	36	0.0%	10.0%		0.0%	7.45	10.25
2486	S. Water	S. Water	US Virgin Islands - St. Thomas	4-Oct-05	18.281	-64.946	Smith, Tyler	20	0.0%	80.0%			7.45	10.25
2487	S. Water	S. Water	US Virgin Islands - St. Thomas	4-Oct-05	18.281	-64.946	Smith, Tyler	20	80.0%	100.0%			7.45	10.25
2488	S. Water	S. Water	US Virgin Islands - St. Thomas	4-Oct-05	18.281	-64.946	Smith, Tyler	20	91.7%	100.0%			7.45	10.25
2489	S. Water	S. Water	US Virgin Islands - St. Thomas	4-Oct-05	18.281	-64.946	Smith, Tyler	20	88.9%	60.0%			7.45	10.25
2490	S. Water	S. Water	US Virgin Islands - St. Thomas	4-Oct-05	18.281	-64.946	Smith, Tyler	20	87.5%	85.7%			7.45	10.25
2491	S. Water	S. Water	US Virgin Islands - St. Thomas	4-Oct-05	18.281	-64.946	Smith, Tyler	20	100.0%	75.0%			7.45	10.25
2492	Hind	Hind	US Virgin Islands - St. Thomas	5-Oct-05	18.202	-65.002	Smith, Tyler	39	6.8%	12.5%		0.0%	7.45	10.25
2493	Hind	Hind	US Virgin Islands - St. Thomas	5-Oct-05	18.202	-65.002	Smith, Tyler	39	0.9%	0.0%		0.0%	7.45	10.25
2494	Hind	Hind	US Virgin Islands - St. Thomas	5-Oct-05	18.202	-65.002	Smith, Tyler	39	5.5%	15.4%		0.0%	7.45	10.25
2495	Hind	Hind	US Virgin Islands - St. Thomas	5-Oct-05	18.202	-65.002	Smith, Tyler	39	4.5%	5.9%		0.0%	7.45	10.25
2496	Hind	Hind	US Virgin Islands - St. Thomas	5-Oct-05	18.202	-65.002	Smith, Tyler	39	1.8%	0.0%		0.0%	7.45	10.25
2497	Hind	Hind	US Virgin Islands - St. Thomas	5-Oct-05	18.202	-65.002	Smith, Tyler	39	4.2%	0.0%		0.0%	7.45	10.25
2498	Savana	Savana	US Virgin Islands - St. Thomas	5-Oct-05	18.341	-65.082	Smith, Tyler	8	10.7%	75.0%			7.45	10.25
2499	Savana	Savana	US Virgin Islands - St. Thomas	5-Oct-05	18.341	-65.082	Smith, Tyler	8	18.8%	61.5%			7.45	10.25
2500	Savana	Savana	US Virgin Islands - St. Thomas	5-Oct-05	18.341	-65.082	Smith, Tyler	8	66.0%	78.9%			7.45	10.25
2501	Savana	Savana	US Virgin Islands - St. Thomas	5-Oct-05	18.341	-65.082	Smith, Tyler	8	56.8%	93.3%			7.45	10.25
2502	Savana	Savana	US Virgin Islands - St. Thomas	5-Oct-05	18.341	-65.082	Smith, Tyler	8	92.0%	90.9%			7.45	10.25
2503	Savana	Savana	US Virgin Islands - St. Thomas	5-Oct-05	18.341	-65.082	Smith, Tyler	8	81.0%	85.0%			7.45	10.25
2516	Botany Bay	St. Thomas (USVI)	US Virgin Islands - St. Thomas	12-Oct-05	18.358	-65.033	Smith, Tyler	5		98.0%		0.0%	8.85	10.25
2517	Seahorse	Seahorse	US Virgin Islands - St. Thomas	13-Oct-05	18.295	-64.868	Smith, Tyler	18	76.2%	100.0%			9.55	10.25
2518	Seahorse	Seahorse	US Virgin Islands - St. Thomas	13-Oct-05	18.295	-64.868	Smith, Tyler	18	92.1%	100.0%			9.55	10.25
2519	Seahorse	Seahorse	US Virgin Islands - St. Thomas	13-Oct-05	18.295	-64.868	Smith, Tyler	18	96.2%	100.0%			9.55	10.25
2520	Seahorse	Seahorse	US Virgin Islands - St. Thomas	13-Oct-05	18.295	-64.868	Smith, Tyler	18	88.8%	94.4%			9.55	10.25
2521	Seahorse	Seahorse	US Virgin Islands - St. Thomas	13-Oct-05	18.295	-64.868	Smith, Tyler	18	93.2%	100.0%			9.55	10.25
2522	Seahorse	Seahorse	US Virgin Islands - St. Thomas	13-Oct-05	18.295	-64.868	Smith, Tyler	18	83.9%	95.5%			9.55	10.25
2523	St. James	St. James	US Virgin Islands - St. Thomas	13-Oct-05	18.295	-64.832	Smith, Tyler	17	72.2%	100.0%			9.55	10.25
2524	St. James	St. James	US Virgin Islands - St. Thomas	13-Oct-05	18.295	-64.832	Smith, Tyler	17		100.0%			9.55	10.25
2525	St. James	St. James	US Virgin Islands - St. Thomas	13-Oct-05	18.295	-64.832	Smith, Tyler	17	75.0%	100.0%			9.55	10.25

Observation Number	Reef or Site Name	Location	Country	Date (DD-MMM-YY)	Latitude (decimal degrees)	Longitude (decimal degrees)	Primary Contributor	Depth (m)	Cover bleached (%)	Colonies bleached (%)	Cover dead (%)	Colonies dead (%)	Observed DHW (°C-weeks)	Maximum DHW (°C-weeks)
2526	St. James	St. James	US Virgin Islands - St. Thomas	13-Oct-05	18.295	-64.832	Smith, Tyler	17	72.7%	100.0%			9.55	10.25
2527	St. James	St. James	US Virgin Islands - St. Thomas	13-Oct-05	18.295	-64.832	Smith, Tyler	17	93.8%	100.0%			9.55	10.25
2528	St. James	St. James	US Virgin Islands - St. Thomas	13-Oct-05	18.295	-64.832	Smith, Tyler	17	83.3%	100.0%			9.55	10.25
2529	College	College	US Virgin Islands - St. Thomas	18-Oct-05	18.186	-65.077	Smith, Tyler	30	5.7%	54.5%		0.0%	10.25	10.25
2530	College	College	US Virgin Islands - St. Thomas	18-Oct-05	18.186	-65.077	Smith, Tyler	30	8.8%	64.7%		0.0%	10.25	10.25
2531	College	College	US Virgin Islands - St. Thomas	18-Oct-05	18.186	-65.077	Smith, Tyler	30	11.5%	66.7%		0.0%	10.25	10.25
2532	College	College	US Virgin Islands - St. Thomas	18-Oct-05	18.186	-65.077	Smith, Tyler	30	8.1%	52.9%		0.0%	10.25	10.25
2533	College	College	US Virgin Islands - St. Thomas	18-Oct-05	18.186	-65.077	Smith, Tyler	30	9.6%	33.3%		0.0%	10.25	10.25
2534	College	College	US Virgin Islands - St. Thomas	18-Oct-05	18.186	-65.077	Smith, Tyler	30	19.2%	36.4%		0.0%	10.25	10.25
2535	Megans	Megans	US Virgin Islands - St. Thomas	19-Oct-05	18.374	-64.934	Smith, Tyler	11	40.0%	0.0%			10.25	10.25
2536	Megans	Megans	US Virgin Islands - St. Thomas	19-Oct-05	18.374	-64.934	Smith, Tyler	11	95.7%	100.0%			10.25	10.25
2537	Megans	Megans	US Virgin Islands - St. Thomas	19-Oct-05	18.374	-64.934	Smith, Tyler	11	81.8%	100.0%			10.25	10.25
2538	Megans	Megans	US Virgin Islands - St. Thomas	19-Oct-05	18.374	-64.934	Smith, Tyler	11	60.0%	100.0%			10.25	10.25
2539	Megans	Megans	US Virgin Islands - St. Thomas	19-Oct-05	18.374	-64.934	Smith, Tyler	11	80.0%	100.0%			10.25	10.25
2540	Megans	Megans	US Virgin Islands - St. Thomas	19-Oct-05	18.374	-64.934	Smith, Tyler	11	75.0%	100.0%			10.25	10.25
2031	Coki Beach	St Thomas	US Virgin Islands - St. Thomas	23-Oct-05	18.350	-64.868	Quinn, Norman	10	100.0%	100.0%	0.0%	0.0%	10.25	10.25
2323	Black Point	St. Thomas	US Virgin Islands - St. Thomas	28-Oct-05	18.347	-64.988	Romano, Sandra	2.5	50.0%		0.0%		10.25	10.25
2324	Black Point	St. Thomas	US Virgin Islands - St. Thomas	28-Oct-05	18.347	-64.988	Romano, Sandra	15	32.0%		0.0%		10.25	10.25
2325	Hull Bay	St. Thomas	US Virgin Islands - St. Thomas	4-Nov-05	18.372	-64.955	Romano, Sandra	2.5	18.0%		0.0%		10.25	10.25
2326	Hull Bay	St. Thomas	US Virgin Islands - St. Thomas	4-Nov-05	18.372	-64.955	Romano, Sandra	12	40.0%		0.0%		10.25	10.25
2327	Botany Bay	St. Thomas	US Virgin Islands - St. Thomas	18-Nov-05	18.358	-65.043	Romano, Sandra	2.5	4.0%		0.0%		9.25	10.25
2328	Botany Bay	St. Thomas	US Virgin Islands - St. Thomas	18-Nov-05	18.358	-65.043	Romano, Sandra	13	49.0%		0.0%		9.25	10.25
2555	Frenchman's Reef	St. Thomas (USVI)	US Virgin Islands - St. Thomas	20-Dec-05	18.318	-64.991	Smith, Tyler	14		32.6%		0.0%	4.3	10.25
2556	Rupert's Rock	St. Thomas (USVI)	US Virgin Islands - St. Thomas	20-Dec-05	18.263	-64.872	Smith, Tyler	3.5		60.5%		2.3%	4.3	10.25
2045	Coki Beach	St Thomas	US Virgin Islands - St. Thomas	27-Jan-06	18.350	-64.868	Quinn, Norman	10	0.0%	0.0%	14.0%	7.0%	0	10.25
2559	Botany Bay	St. Thomas (USVI)	US Virgin Islands - St. Thomas	3-Feb-06	18.358	-65.033	Smith, Tyler	14		48.8%		1.2%	0	10.25
2560	Megans	St. Thomas (USVI)	US Virgin Islands - St. Thomas	3-Feb-06	18.374	-64.934	Smith, Tyler	11		42.9%		0.0%	0	10.25
2046	Buck Is Bay	St Thomas	US Virgin Islands - St. Thomas	4-Feb-06	18.276	-64.898	Quinn, Norman	10	10.0%	10.0%	1.0%	6.7%	0	10.25
2563	Hind E.	St. Thomas (USVI)	US Virgin Islands - St. Thomas	13-Feb-06	18.202	-65.002	Smith, Tyler	39		18.1%		0.0%	0	10.25
2050	Buck Is Slope	St Thomas	US Virgin Islands - St. Thomas	19-Mar-06	18.279	-64.897	Quinn, Norman	10	82.0%	82.0%	13.0%	14.0%	0	10.25
2051	Congo Cay	St Thomas	US Virgin Islands - St. Thomas	25-Mar-06	18.366	-64.805	Quinn, Norman	10	58.0%	57.5%	6.0%	7.5%	0	10.25
2569	Botany Bay	St. Thomas (USVI)	US Virgin Islands - St. Thomas	28-Mar-06	18.358	-65.033	Smith, Tyler	14		20.3%		4.1%	0	10.25
2570	Megans	St. Thomas (USVI)	US Virgin Islands - St. Thomas	28-Mar-06	18.374	-64.934	Smith, Tyler	11		10.0%		0.0%	0	10.25
2571	Benner	St. Thomas (USVI)	US Virgin Islands - St. Thomas	29-Mar-06	18.313	-64.861	Smith, Tyler	7		20.4%		0.0%	0	10.25
2572	Buck Island, STT	St. Thomas (USVI)	US Virgin Islands - St. Thomas	29-Mar-06	18.279	-64.898	Smith, Tyler	15		57.4%		2.1%	0	10.25
2573	College East	St. Thomas (USVI)	US Virgin Islands - St. Thomas	31-Mar-06	18.186	-65.077	Smith, Tyler	30		12.6%		0.0%	0	10.25
2052	Great Bay West Reef	St Thomas	US Virgin Islands - St. Thomas	1-Apr-06	18.319	-64.836	Quinn, Norman	10	60.0%	60.0%	6.0%	15.0%	0	10.25
2574	S. Capella	St. Thomas (USVI)	US Virgin Islands - St. Thomas	4-Apr-06	18.263	-64.872	Smith, Tyler	22		48.6%		0.0%	0	10.25
2575	St. James	St. Thomas (USVI)	US Virgin Islands - St. Thomas	4-Apr-06	18.295	-64.832	Smith, Tyler	17		19.0%		2.4%	0	10.25
2576	S. Water	St. Thomas (USVI)	US Virgin Islands - St. Thomas	5-Apr-06	18.281	-64.946	Smith, Tyler	20		46.8%		0.0%	0	10.25
2577	Hind E.	St. Thomas (USVI)	US Virgin Islands - St. Thomas	7-Apr-06	18.202	-65.002	Smith, Tyler	39		4.3%		0.0%	0	10.25
2054	Great St James	St Thomas	US Virgin Islands - St. Thomas	9-Apr-06	18.295	-64.832	Quinn, Norman	10	74.0%	74.0%	13.0%	26.0%	0	10.25
2578	Black Point	St. Thomas (USVI)	US Virgin Islands - St. Thomas	10-Apr-06	18.345	-64.986	Smith, Tyler	10		17.1%		13.7%	0	10.25
2579	Savanna	St. Thomas (USVI)	US Virgin Islands - St. Thomas	10-Apr-06	18.341	-65.082	Smith, Tyler	8		38.4%		1.0%	0	10.25
2582	Seahorse	St. Thomas (USVI)	US Virgin Islands - St. Thomas	29-Apr-06	18.295	-64.868	Smith, Tyler	18		47.4%		5.2%	0	10.25
2583	Grammanik	St. Thomas (USVI)	US Virgin Islands - St. Thomas	3-May-06	18.191	-64.950	Smith, Tyler	36		14.9%		1.1%	0	10.25
2055	Coki Beach	St Thomas	US Virgin Islands - St. Thomas	15-May-06	18.350	-64.868	Quinn, Norman	10	0.0%	0.0%	15.0%	8.0%	0	10.25
2585	Botany Bay	St. Thomas (USVI)	US Virgin Islands - St. Thomas	21-Jun-06	18.358	-65.033	Smith, Tyler	14	1.9%	7.5%	5.0%	1.9%	0	10.25
2586	Megans	St. Thomas (USVI)	US Virgin Islands - St. Thomas	21-Jun-06	18.374	-64.934	Smith, Tyler	11	0.8%	41.4%	0.0%	0.0%	0	10.25
2587	Benner	St. Thomas (USVI)	US Virgin Islands - St. Thomas	23-Jun-06	18.313	-64.861	Smith, Tyler	7	4.0%	22.4%		0.0%	0	10.25
2588	Seahorse	St. Thomas (USVI)	US Virgin Islands - St. Thomas	23-Jun-06	18.295	-64.868	Smith, Tyler	18	0.0%	61.9%	11.7%	0.0%	0	10.25
2589	Buck Island, STT	St. Thomas (USVI)	US Virgin Islands - St. Thomas	28-Jun-06	18.279	-64.898	Smith, Tyler	15	0.0%	8.2%	2.4%	3.3%	0	10.25
2592	Savanna	St. Thomas (USVI)	US Virgin Islands - St. Thomas	13-Jul-06	18.341	-65.082	Smith, Tyler	8	0.0%	72.5%	8.8%	0.0%	0	10.25
2593	Black Point	St. Thomas (USVI)	US Virgin Islands - St. Thomas	14-Jul-06	18.345	-64.986	Smith, Tyler	10	1.1%	12.5%		0.0%	0	10.25
2594	S. Capella	St. Thomas (USVI)	US Virgin Islands - St. Thomas	19-Jul-06	18.263	-64.872	Smith, Tyler	22	0.0%	34.3%	23.5%	0.0%	0	10.25
2595	St. James	St. Thomas (USVI)	US Virgin Islands - St. Thomas	19-Jul-06	18.295	-64.832	Smith, Tyler	17	0.0%	12.0%	2.6%	0.0%	0	10.25
2596	Hind E.	St. Thomas (USVI)	US Virgin Islands - St. Thomas	21-Jul-06	18.202	-65.002	Smith, Tyler	39	0.2%	18.9%	7.3%	0.0%	0	10.25
2597	S. Water	St. Thomas (USVI)	US Virgin Islands - St. Thomas	24-Jul-06	18.281	-64.946	Smith, Tyler	20	1.9%	28.0%	0.3%	0.0%	0	10.25

Observation Number	Reef or Site Name	Location	Country	Date (DD-MMM-YY)	Latitude (decimal degrees)	Longitude (decimal degrees)	Primary Contributor	Depth (m)	Cover bleached (%)	Colonies bleached (%)	Cover dead (%)	Colonies dead (%)	Observed DHW (°C-weeks)	Maximum DHW (°C-weeks)
2598	Grammanik	St. Thomas (USVI)	US Virgin Islands - St. Thomas	26-Jul-06	18.191	-64.950	Smith, Tyler	36	1.1%	3.6%	7.6%	0.0%	0	10.25
2599	College East	St. Thomas (USVI)	US Virgin Islands - St. Thomas	31-Jul-06	18.186	-65.077	Smith, Tyler	30	0.8%	8.0%	10.6%	0.0%	0	10.25
2280	Tres Playas	La Blanquilla	Venezuela - Antilles	25-Aug-05	11.888	-64.650	Rodriguez, Sebastian	6		3.3%			0.5	5.65
2281	Tres Playas	La Blanquilla	Venezuela - Antilles	25-Aug-05	11.888	-64.650	Rodriguez, Sebastian	6		0.4%			0.5	5.65
2285	Dos Mosquises Sur	Archipielago Los Roques	Venezuela - Antilles	1-Oct-05	11.793	-66.895	Rodriguez, Sebastian	10	0.4%	0.7%			0	4
2286	Madrisqui	Archipielago Los Roques	Venezuela - Antilles	1-Oct-05	11.935	-66.655	Rodriguez, Sebastian	8	0.8%	0.8%			0	4
2674	Boca Cote	Los Roques	Venezuela - Antilles	29-Oct-05	11.764	-66.703	Villamizar, Estrella	11.5		0.3%			4	4
2675	Dos Mosquises	Los Roques	Venezuela - Antilles	29-Oct-05	11.793	-66.895	Villamizar, Estrella	10.5		0.4%			4	4
2676	Boca del Medio	Los Roques	Venezuela - Antilles	30-Oct-05	11.897	-66.583	Villamizar, Estrella	6.5		0.9%			4	4
2677	Cayo Sal	Los Roques	Venezuela - Antilles	30-Oct-05	11.736	-66.844	Villamizar, Estrella	10.5		0.4%			4	4
2319	Dos Mosquises Sur	Archipielago Los Roques	Venezuela - Antilles	12-Dec-06	11.793	-66.895	Rodriguez, Sebastian	10	0.4%	0.0%	0.0%	0.0%	0.5	4
2320	Madrisqui	Archipielago Los Roques	Venezuela - Antilles	13-Dec-06	11.935	-66.655	Rodriguez, Sebastian	8	0.0%	0.0%	0.0%	0.0%	0.5	4
2282	Playa Caiman	Morrocoy	Venezuela - Mainland	25-Sep-05	10.857	-68.236	Rodriguez, Sebastian	4	0.0%	0.0%			0	3.65
2283	Cayo Norte	Cuare	Venezuela - Mainland	27-Sep-05	10.830	-68.360	Rodriguez, Sebastian	7	0.0%	0.0%			0.6	3.65
2284	Bajo de Cayo Sombrero	Morrocoy	Venezuela - Mainland	28-Sep-05	10.882	-68.218	Rodriguez, Sebastian	8	0.2%	0.7%			0.6	3.65
2114	Cayo Sombrero (Morrocoy Nati	Cayo Sombrero (Morrocoy Nati	Venezuela - Mainland	30-Sep-05	10.887	-68.221	ReefBase	8.5	21.0%				0.6	3.65
2287	Bajo de Cayo Sombrero	Morrocoy	Venezuela - Mainland	28-Oct-05	10.882	-68.218	Rodriguez, Sebastian	8		16.2%			3.65	3.65
2288	Bajo de Cayo Sombrero	Morrocoy	Venezuela - Mainland	28-Oct-05	10.882	-68.218	Rodriguez, Sebastian	8		2.0%			3.65	3.65
2289	Bajo de Cayo Sombrero	Morrocoy	Venezuela - Mainland	28-Oct-05	10.882	-68.218	Rodriguez, Sebastian	8		2.0%			3.65	3.65
2290	Suanchez	Morrocoy	Venezuela - Mainland	17-Dec-05	10.805	-68.285	Rodriguez, Sebastian	5	0.3%	1.3%			3.65	3.65
2291	Suanchez	Morrocoy	Venezuela - Mainland	17-Dec-05	10.805	-68.285	Rodriguez, Sebastian	5	20.5%	14.1%			3.65	3.65
2292	Suanchez	Morrocoy	Venezuela - Mainland	17-Dec-05	10.805	-68.285	Rodriguez, Sebastian	5	8.9%	2.6%			3.65	3.65
2293	Mero	Morrocoy	Venezuela - Mainland	18-Dec-05	10.833	-68.250	Rodriguez, Sebastian	9	4.6%	1.4%			3.05	3.65
2294	Mero	Morrocoy	Venezuela - Mainland	18-Dec-05	10.833	-68.250	Rodriguez, Sebastian	9	4.6%	5.7%			3.05	3.65
2295	Mero	Morrocoy	Venezuela - Mainland	18-Dec-05	10.833	-68.250	Rodriguez, Sebastian	9	0.5%	1.4%			3.05	3.65
2296	Pescadores	Morrocoy	Venezuela - Mainland	18-Dec-05	10.869	-68.214	Rodriguez, Sebastian	9.5	6.5%	2.9%			3.05	3.65
2297	Pescadores	Morrocoy	Venezuela - Mainland	18-Dec-05	10.869	-68.214	Rodriguez, Sebastian	9.5	5.1%	5.1%			3.05	3.65
2298	Pescadores	Morrocoy	Venezuela - Mainland	18-Dec-05	10.869	-68.214	Rodriguez, Sebastian	9.5	17.4%	7.3%			3.05	3.65
2299	Bajo de Cayo Sombrero	Morrocoy	Venezuela - Mainland	19-Dec-05	10.882	-68.218	Rodriguez, Sebastian	8		7.2%		1.0%	3.05	3.65
2300	Bajo de Cayo Sombrero	Morrocoy	Venezuela - Mainland	19-Dec-05	10.882	-68.218	Rodriguez, Sebastian	8		10.3%			3.05	3.65
2301	Bajo de Cayo Sombrero	Morrocoy	Venezuela - Mainland	19-Dec-05	10.882	-68.218	Rodriguez, Sebastian	8		13.4%			3.05	3.65
2302	Cayo Sombrero	Morrocoy	Venezuela - Mainland	19-Dec-05	10.883	-68.212	Rodriguez, Sebastian	12.5	2.1%	4.2%			3.05	3.65
2303	Cayo Sombrero	Morrocoy	Venezuela - Mainland	19-Dec-05	10.883	-68.212	Rodriguez, Sebastian	12.5	13.2%	7.3%			3.05	3.65
2304	Cayo Sombrero	Morrocoy	Venezuela - Mainland	19-Dec-05	10.883	-68.212	Rodriguez, Sebastian	12.5	3.8%	2.1%			3.05	3.65
2305	Bajo de Cayo Sombrero	Morrocoy	Venezuela - Mainland	25-Jan-06	10.882	-68.218	Rodriguez, Sebastian	12.5		3.3%		0.0%	0	3.65
2306	Bajo de Cayo Sombrero	Morrocoy	Venezuela - Mainland	25-Jan-06	10.882	-68.218	Rodriguez, Sebastian	12.5		11.0%		0.0%	0	3.65
2307	Bajo de Cayo Sombrero	Morrocoy	Venezuela - Mainland	25-Jan-06	10.882	-68.218	Rodriguez, Sebastian	12.5		3.3%		0.0%	0	3.65
2308	Playa Blanca Norte	Mochima	Venezuela - Mainland	29-Jan-06	10.394	-64.335	Rodriguez, Sebastian	4	0.0%	0.0%	0.0%	0.0%	0	3.05
2309	Playa Blanca Sur	Mochima	Venezuela - Mainland	30-Jan-06	10.388	-64.340	Rodriguez, Sebastian	4	0.0%	0.0%	0.0%	0.0%	0	3.05
2310	Punta La Virgen	Mochima	Venezuela - Mainland	31-Jan-06	10.399	-64.337	Rodriguez, Sebastian	4	0.0%	0.0%		0.0%	0	3.05
2311	Bajo de Cayo Sombrero	Morrocoy	Venezuela - Mainland	8-Feb-06	10.882	-68.218	Rodriguez, Sebastian	12.5		5.6%			0	3.65
2312	Bajo de Cayo Sombrero	Morrocoy	Venezuela - Mainland	8-Feb-06	10.882	-68.218	Rodriguez, Sebastian	12.5		4.4%			0	3.65
2313	Bajo de Cayo Sombrero	Morrocoy	Venezuela - Mainland	6-May-06	10.882	-68.218	Rodriguez, Sebastian	12.5	1.0%	1.2%	0.0%	0.0%	0	3.65
2314	Bajo de Cayo Sombrero	Morrocoy	Venezuela - Mainland	6-May-06	10.882	-68.218	Rodriguez, Sebastian	12.5	0.0%	0.4%	0.0%	0.0%	0	3.65
2315	Bajo de Cayo Sombrero	Morrocoy	Venezuela - Mainland	6-May-06	10.882	-68.218	Rodriguez, Sebastian	12.5	0.0%	1.2%	0.0%	0.0%	0	3.65
2316	Bajo de Cayo Sombrero	Morrocoy	Venezuela - Mainland	19-Oct-06	10.882	-68.218	Rodriguez, Sebastian	12.5	0.0%	1.2%	0.0%	0.0%	0	3.65
2317	Playa Caiman	Morrocoy	Venezuela - Mainland	20-Oct-06	10.857	-68.236	Rodriguez, Sebastian	4	0.0%	0.0%	0.0%	0.0%	0	3.65
2318	Cayo Norte	Cuare	Venezuela - Mainland	21-Oct-06	10.830	-68.360	Rodriguez, Sebastian	7	0.0%	0.0%	0.0%	0.0%	0	3.65
